# Hydrogen Isotope Exchange Catalyzed by Ru Nanocatalysts: Labelling of Complex Molecules Containing *N*‐Heterocycles and Reaction Mechanism Insights

**DOI:** 10.1002/chem.201905651

**Published:** 2020-03-09

**Authors:** Viktor Pfeifer, Marie Certiat, Donia Bouzouita, Alberto Palazzolo, Sébastien Garcia‐Argote, Elodie Marcon, David‐Alexandre Buisson, Philippe Lesot, Laurent Maron, Bruno Chaudret, Simon Tricard, Iker del Rosal, Romuald Poteau, Sophie Feuillastre, Grégory Pieters

**Affiliations:** ^1^ SCBM, JOLIOT Institute, CEA Université Paris-Saclay 91191 Gif-sur-Yvette France; ^2^ LPCNO, Laboratoire de Physique et Chimie de Nano-Objets, UMR 5215 INSA-CNRS-UPS Institut National des Sciences Appliquées 135, Avenue de Rangueil 31077 Toulouse France; ^3^ RMN en Milieu Orienté, ICMMO, UMR CNRS 8182 UFR d'Orsay, Université Paris-Saclay Bât. 410 91405 Orsay cedex France

**Keywords:** C−H activation, deuterium, hydrogen isotopic exchange, nanocatalysis, tritium

## Abstract

Ruthenium nanocatalysis can provide effective deuteration and tritiation of oxazole, imidazole, triazole and carbazole substructures in complex molecules using D_2_ or T_2_ gas as isotopic sources. Depending on the substructure considered, this approach does not only represent a significant step forward in practice, with notably higher isotope uptakes, a broader substrate scope and a higher solvent applicability compared to existing procedures, but also the unique way to label important heterocycles using hydrogen isotope exchange. In terms of applications, the high incorporation of deuterium atoms, allows the synthesis of internal standards for LC‐MS quantification. Moreover, the efficacy of the catalyst permits, even under subatmospheric pressure of T_2_ gas, the preparation of complex radiolabeled drugs owning high molar activities. From a fundamental point of view, a detailed DFT‐based mechanistic study identifying undisclosed key intermediates, allowed a deeper understanding of C−H (and N−H) activation processes occurring at the surface of metallic nanoclusters.

## Graphical abstract

**Ruthenium nanocatalysis** can provide effective deuteration and tritiation of oxazole, imidazole, triazole and carbazole substructures in complex molecules using D_2_ or T_2_ gas as isotopic sources. In terms of application, the high incorporation of deuterium atoms allows the synthesis of deuterated internal standards for LC/MS quantification and the efficacy of the catalytic process permits the preparation of complex radiolabelled drugs owning high specific activities by using a subatmospheric pressure of T_2_ gas.

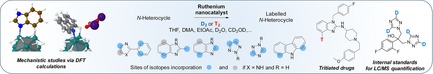

## Introduction

**Figure 1:**
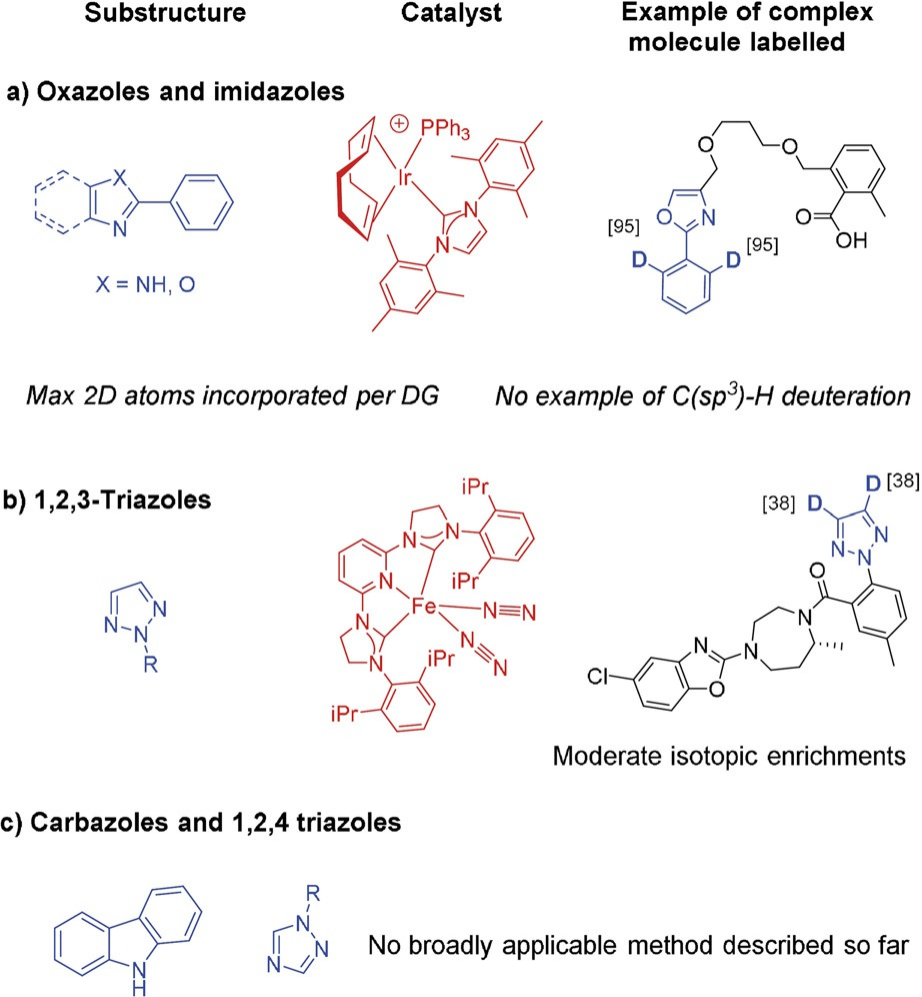
Main previous works on the deuterium/tritium labelling of oxazole, imidazole, triazole and carbazole substructures using HIE procedures.

Nitrogen‐based heterocycles such as imidazoles, benzimidazoles and triazoles rank on the top of the most frequently approved small molecule drugs by US FDA.^1^ For example, 1,2,4‐triazoles are prominent structural motifs in many commercial antifungal drugs,^2^ anti‐tumor‐,^3^ anti‐migraine agents^4^ and many others.^5^ Other heterocycles such as oxazoles and carbazoles are also commonly encountered substructures in drug development,^6^ biologically active natural products^7^ and in material science as fluorescent molecules where deuterium incorporation can be interesting for the enhancement of fluorescence properties.^8^ Due to the occurrence of *N*‐heterocycles in many types of useful molecules, the development of efficient methods for isotopic labelling, particularly with hydrogen isotopes, is of paramount importance. Indeed, tritiated analogues of drug candidates are considered as essential tools for studying the in vivo fate of drug candidates during absorption, distribution, metabolism and excretion (ADME) studies.^9^ In this context, the development of robust methods allowing the incorporation of at least 0.5 T (corresponding to a molar activity of 15 Ci mmol^−1^) in complex molecules using mild reaction conditions (ideally with a subatmospheric pressure of T_2_ gas) is of particular interest. Deuterated compounds are also widely employed in various life‐science applications, such as metabolomics and proteomics as internal standards for quantitative LC‐ and GC‐MS analyses.^10^ To facilitate the synthetic access to these valuable isotopically labelled compounds, the discovery of new approaches enabling the selective incorporation of at least three deuterium atoms in small complex molecules, is appealing.^11^ In the case of molecules containing oxazole and imidazole substructures, high isotopic enrichments can be obtained using Ir^I^ catalysts, but only for compounds owning an aromatic ring in position 2 (see Figure 1 a). In addition, only C(*sp* ^2^)−H bonds in the γ‐position relative to the directing atom (DA) can be activated efficiently, and as a consequence only two deuterium atoms can be incorporated (per DA).^12^ Concerning the deuterium and tritium labelling of some other *N*‐heterocyclic cores such as 1,2,3‐triazole, remarkable results have been described recently by Chirik and co‐workers employing an air sensitive bis(arylimidazol‐2‐ylidene)pyridine iron bis(dinitrogen)^13^ (see Figure 1 b) and a dimeric nickel hydride complex.^14^

**Figure 2:**
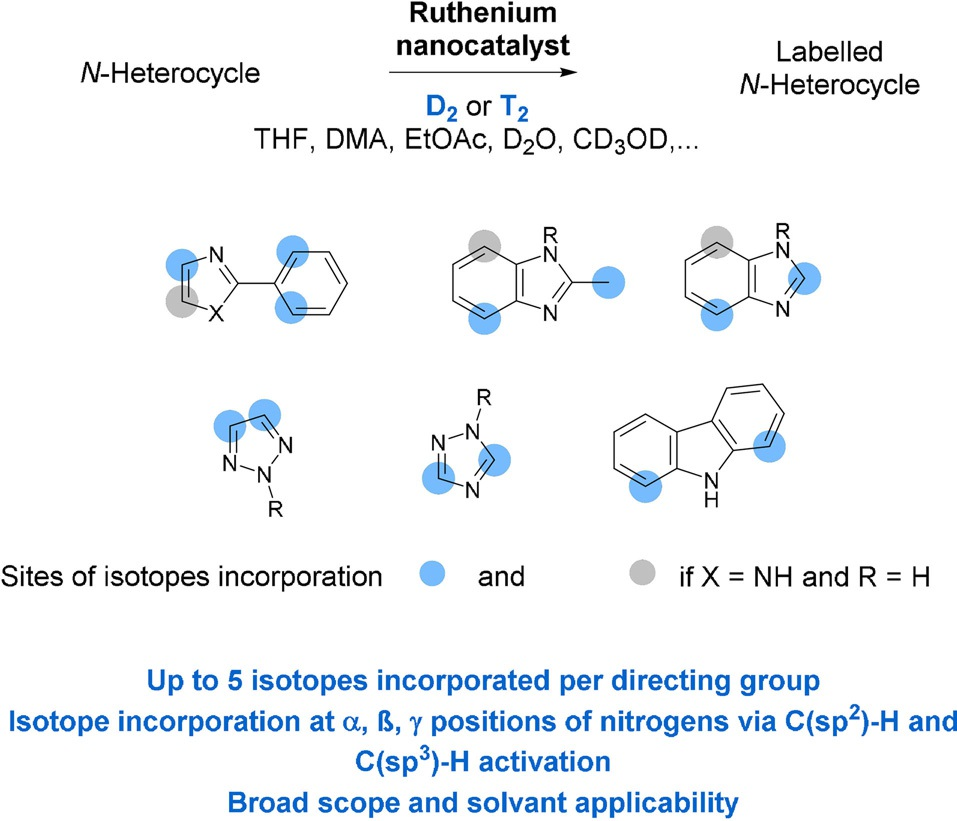
Scope and regioselectivity of HIE reactions catalyzed by a Ru nanocatalyst.

Even if the iron catalyzed hydrogen isotope exchange (HIE) procedure possesses the advantage to work at low pressure of D_2_ (1 bar), it afforded moderate deuterium incorporation on the 1,2,3‐triazole moiety of suvorexant (0.8D incorporated). For 1,2,4‐triazole‐ and carbazole substructures (see Figure 1 c), no broadly applicable HIE methods have been described so far. In this paper, we demonstrate that the selective deuteration and tritiation of the oxazole, (benz)imidazole, triazole and carbazole pattern in complex molecules can be achieved using Ru nanoparticles (RuNps) as catalyst (see Figure 2). For the oxazole and imidazole substructures, this approach possesses a broader scope of applications, a higher solvent applicability, and allows, in most cases, higher isotope incorporations under mild reactions conditions compared to previously described methods.

On top of that, this work describes also the first general method for the selective deuterium and tritium labelling of the 1,2,4‐triazole and carbazole scaffold by HIE reactions. From an application point of view, these reactions can be used for the late stage radiolabelling of complex pharmaceuticals (applying a low pressure of tritium gas) and the preparation of internal standards for LC‐MS quantification. On a more fundamental aspect, a deeper understanding of the reaction mechanisms involved in C−H and N−H activation processes at the surface of the Ru nanocatalyst was provided by DFT calculations. These studies notably revealed the formation of different types of key intermediates, explaining the isotope incorporation at α, β and γ positions of the directing nitrogen atom.

## Results and Discussion

### Labelling of oxazole derivatives

**Figure 3:**
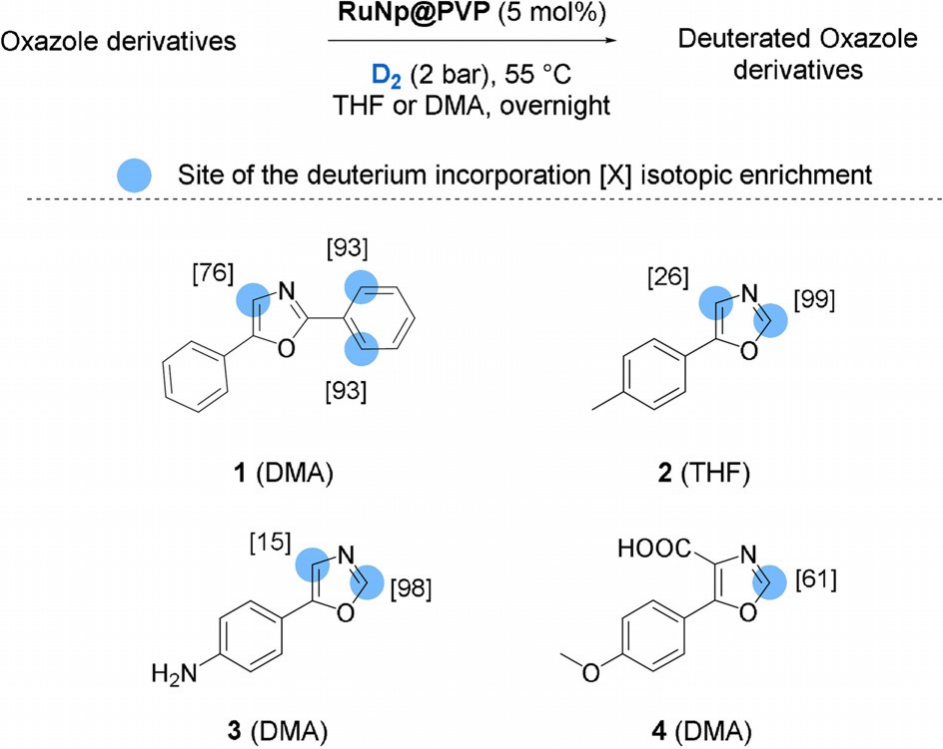
Labelling of oxazole derivatives using RuNp catalyzed HIE.

We decided to initiate our studies with the deuteration of oxazole derivatives because effective methods to label this substructure are still scarce. Based on our previous results, we envisioned that Ru nanoparticles might be used as a catalyst for the deuteration of such nitrogen containing heterocycles.^15^ Different oxazole derivatives **1**–**4** were successfully deuterated at 50 °C in THF or DMA as solvent using a catalytic amount of ruthenium nanoparticles (5 mol %) stabilized in a polyvinylpyrrolidone matrix (RuNp@PVP) under 2 bar of D_2_ gas (see Figure 3). Under these reaction conditions, diphenyloxazole **1** was labelled with a total uptake of 2.6 deuterium atoms, incorporated at the oxazole core and in the *ortho* positions of the phenyl group. The possibility to selectively activate C−H bonds at α and γ positions to the nitrogen atom of **1** permitted to obtain higher deuterium incorporation than the one achievable over homogeneous Ir^I^ catalysis (where only two deuteriums (2D) can be theoretically introduced).^16^ The substrates **2**–**4** were used to demonstrate the broader substrate scope of RuNp‐catalyzed HIE, as they cannot be labeled by other existing approaches such as Ir^I^‐catalysis due to their substitution pattern. Indeed, compounds **2** and **3** were successfully deuterated at the C_2_ and C_4_ position of the oxazole ring (both α‐positions relative to the nitrogen atom). Furthermore, regio‐ and chemoselective labelling at C_2_ of carboxylic compound **4** was achieved by using DMA as solvent.

**Figure 4:**
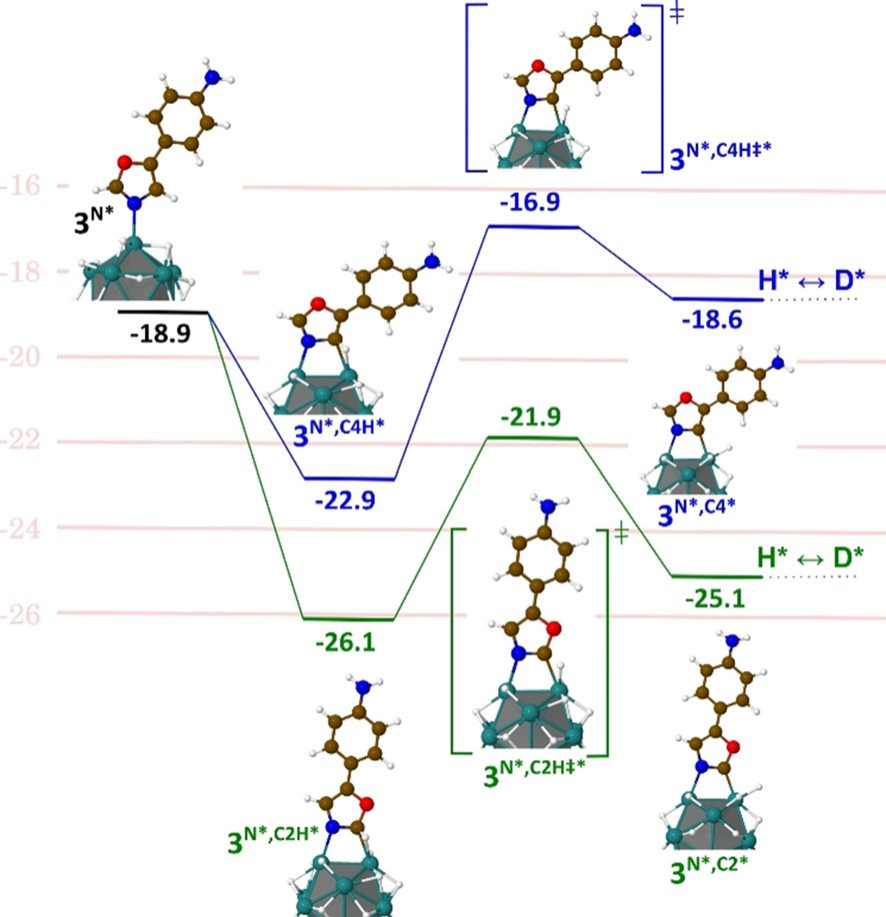
Energy diagram for the first steps of the Langmuir‐Hinshelwood‐type H/D exchange on the C_2_ (green pathway) and C_4_ (blue pathway) position of the oxazole ring of compound **3**; energie values are given in kcal mol^−1^. The full pathway, that is, with the D incorporation, is given in Figure S1.

Theoretical calculations at the DFT‐PBE level of theory were conducted in order to study the reaction pathway leading to the C(sp^2^)−H activation at the α positions of the nitrogen atom. To achieve this study, a 0.5 nm ruthenium cluster with 1.4 H atoms *per* Ru surface atom (Ru_13_H_17_) was used as RuNp model.^17^ As we can see in Figure 4, the coordination of **3** to the RuNp model through the lone pair of the nitrogen atom is an exothermic process (3^N*^: ca. −19 kcal mol^−1^; the labelling is explained in the SI). From this intermediate, a stabilizing agostic interaction can be established between the C_2_−H (3^N*,C2H*^, green pathway) or C_4_‐H (3^N*,C4H*^, blue pathway) group and one of the first‐neighbored ruthenium atoms to the one that interacts with the nitrogen atom. The formation of this three‐center, two‐electron bond between a C−H bonding orbital and an empty metal orbital is evidenced by the slight carbon pyramidalization (accompanied by the lifting of the hydrogen atom out of the plane of the oxazole ring). From both 4‐membered dimetallacycle intermediates 3^N*,C4H*^ and 3^N*,C2H*^, the C−H bond activation is a kinetically accessible process with an activation barrier of 6.0 kcal mol^−1^ on C_4_ position (3^N*,C4H≠*^) and of 4.2 kcal mol^−1^ on C_2_ position (3^N*,C2H≠*^). However, from a thermodynamic point of view, the C−H bond breaking is an almost athermic process at the C_2_ position (3^N*,C2*^: +1 kcal mol^−1^ with respect to 3^N*,C2H*^) whereas it is clearly endothermic at the C_4_ position (3^N*,C4*^: +4.3 kcal mol^−1^ w.r.t. 3^N*,C4H*^). The H* atom can then easily exchange its position with one of the numerous deuterides already present at the surface of the RuNps (H*↔D* step in Figure 4), as previously demonstrated by NMR experiments.9a One available deuteride in the vicinity of the active site can then recombine with the C_X_ atom, provided that kinetics and thermodynamics do not impede it (the full pathway is given in Figure S1). In this mechanism, the first key parameter is the formation of a 4‐membered dimetallacyle in both pathways (3^N*,CXH*^, **X**=2 or 4). But owing to the small barrier heights, a second key parameter is the competition between the (C−H)*→(C)*(H)* reaction (i.e., 3^N*,CXH*^→(3^N*,CX*^)(H*)) and the (C)*(H)*→(C−H)* back reaction. The lower deuterium incorporation at the C_4_ position (15 %) versus the C_2_ position (98 %), experimentally observed for compound **3**, can therefore be explained by the small barrier (only 1.7 kcal mol^−1^) for the back reaction (from 3^N*,C4*^ to 3^N*,C4H*^), thereby reducing the efficiency of the global process (see also Figure S2 for a fully detailed pathway, that is, up to the final D‐incorporation). A similar explanation can probably be invoked for compound **2**, where the isotopic labelling at the C_2_ position (99 %) was found to be higher than that measured at the C_4_ position (26 %).

### Labelling of imidazole derivatives

**Figure 5:**
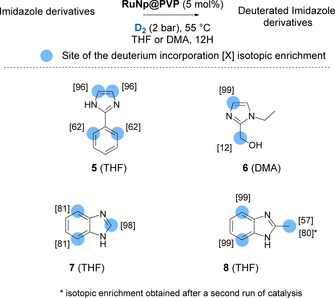
Examples of deuterated imidazole derivatives.

Further, RuNp@PVP catalyzed deuterations were also conducted on various imidazole derivatives (see Figure 5). 2‐Phenylimidazole **5** showed high deuterium incorporation in the *ortho*‐positions of the phenyl and at both α‐positions relative to the imidazole nitrogen atoms. This result represented another example where the use of RuNps as catalyst yielded a higher deuterium incorporation (3.3D) than Ir^I^‐based catalysts, which would lead to the incorporation of only two deuterium atoms at a maximum.

**Figure 6:**
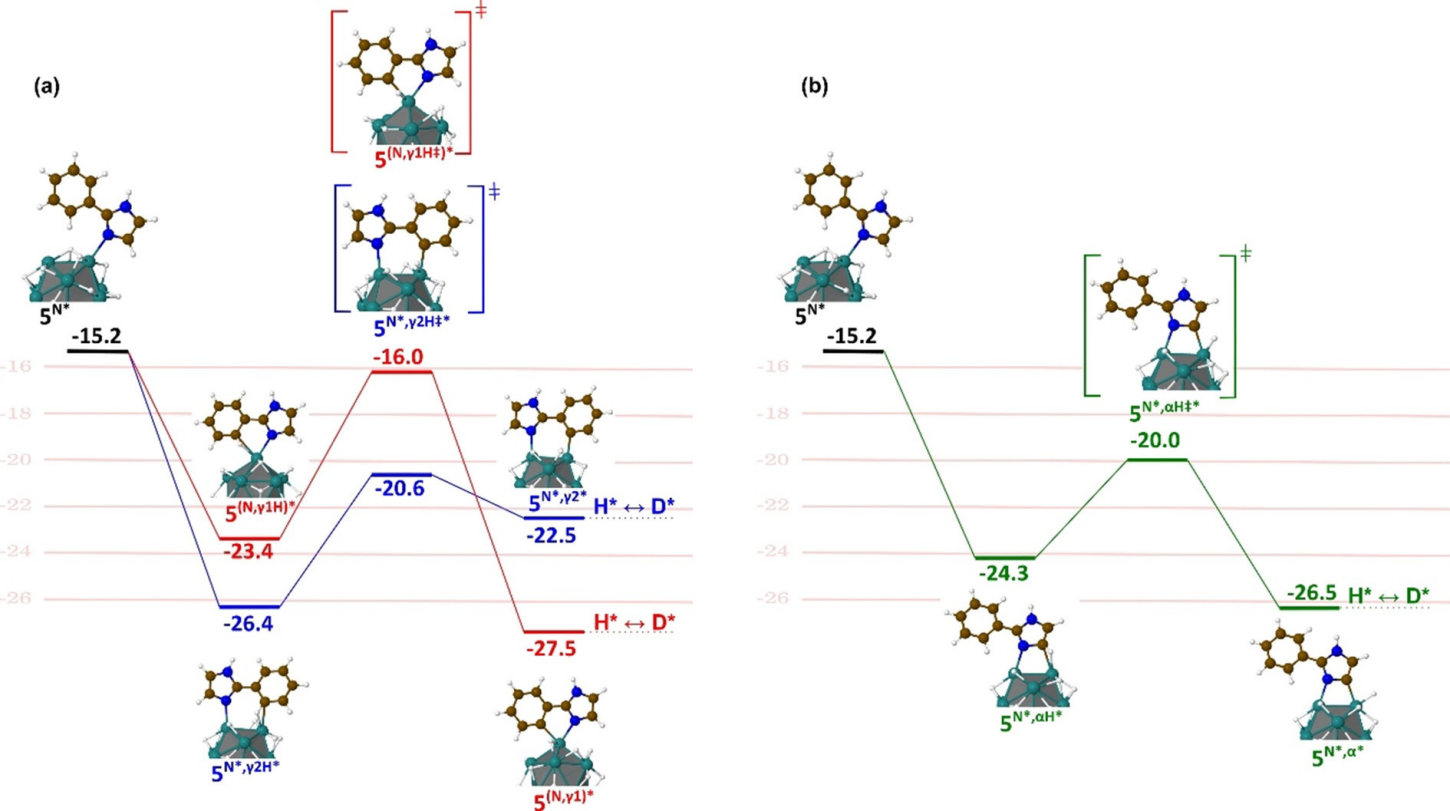
a) Energy diagram for the Langmuir‐Hinshelwood‐type H/D exchange on **5** in the *ortho*‐position of the phenyl (blue and red pathways). b) at α‐positions relative to the imidazole nitrogen atoms (green pathway); energies are given in kcal mol^−1^. See also Figure S2 for a fully detailed pathway, that is, up to the final D‐incorporation.

The deuterium incorporation on both sites of **5** has also been considered from a theoretical point of view in order to identify notably, the key intermediate leading to the labelling at the γ‐positions of the coordinating nitrogen. In order to answer this question, two competitive pathways were investigated (**γ1** in red and **γ2** in blue, Figure 6 a). Independent of the considered pathway, compound **5** is initially adsorbed at the RuNp surface through the lone pair of the N_3_ nitrogen atom to give 5^N*^, followed by the formation of a stabilizing C−H agostic interaction. It is noteworthy that two different adducts can be formed, either with the same ruthenium atom (5^N*,γ1H*^) or with two neighboring ruthenium atoms (5^N*,γ2H*^). They lead, respectively to a five‐membered metallacyle (a key intermediate analogue to the one proposed in homogeneous catalysis) or a six‐membered dimetallacycle adduct. The pathway that involves a six‐membered dimetallacycle is both endothermic and kinetically accessible (blue pathway in Figure 6 a). This mechanism cannot be excluded for the deuterium incorporation but it is probably inefficient due to the small barrier (1.9 kcal mol^−1^) and the exothermicity (ca.‐4 kcal mol^−1^) of the (C)*(H)* ↔ (C−H)* back reaction (i.e., 5^N*,γ2*^↔5^N*,γ2H*^). The C−H activation involving a five‐membered metallacycle (red pathway in Figure 6 a) is also kinetically accessible, with an activation barrier of 7.4 kcal mol^−1^, but thermodynamically more favorable (−4.1 kcal mol^−1^) on the contrary to the **γ_1_** case.

**Figure 7:**
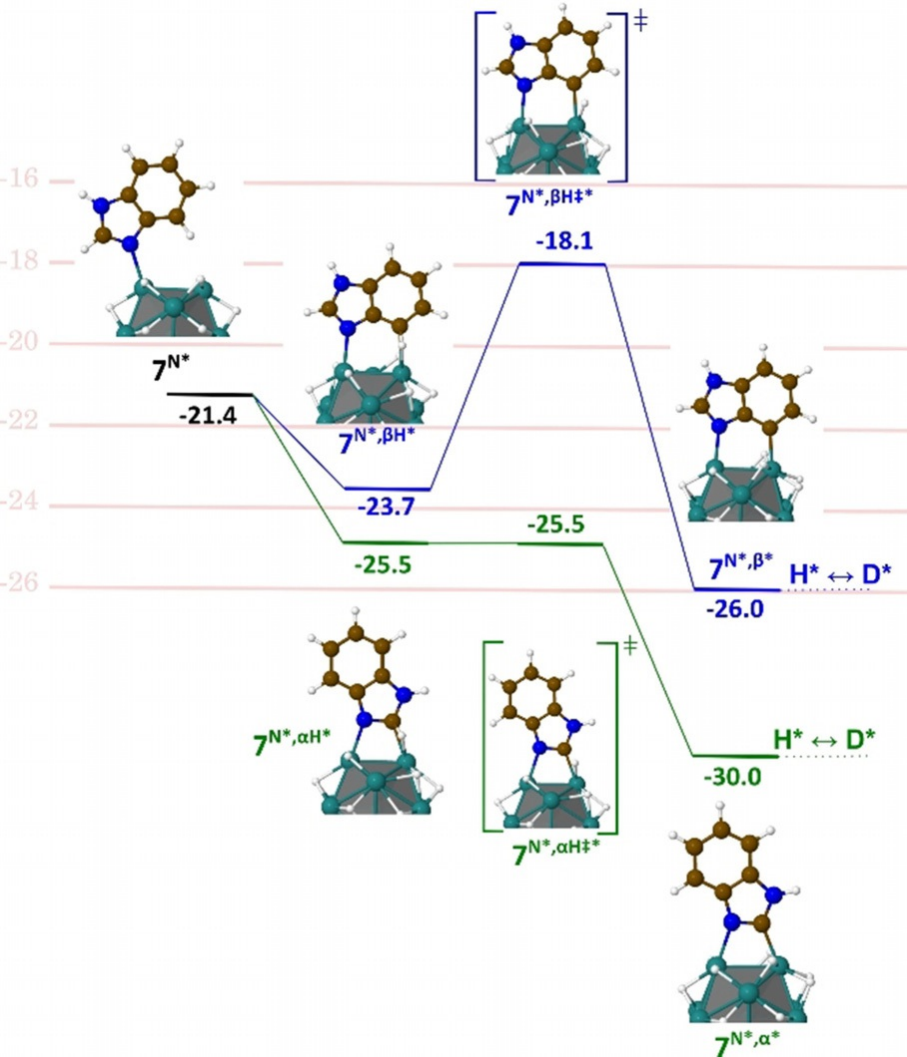
Energy diagram for the Langmuir‐Hinshelwood‐type H/D exchange on **7** in α (green pathway) and β (blue pathway) positions of the nitrogen atoms; energies are given in kcal mol^−1^_._ See also Figure S3 for a fully detailed pathway, up to the final D‐incorporation.

Thus, the deuterium incorporation at the γ‐position of the nitrogen is most probably due to a process that goes through a five‐membered metallacyle intermediate such as in homogeneous catalysis. The very efficient HIE in α to nitrogen atoms (96 %) corresponds to a thermodynamically favorable mechanism (−2.1 kcal mol^−1^ w.r.t. (C−H)*, green pathway in Figure 6 b) involving a 4‐membered dimetallacycle as key intermediate and a low activation barrier for the C−H bond breaking step (4.3 kcal mol^−1^). Thus, similar to the C_2_H activation pathway in compound **3** (see Figure 4, green pathway) the competition between the (C)*(H)*↔(C−H)* back reaction (i.e., (5^N*,α*^)(H*) ↔5^N*,αH*^) and the (C)*(D*)*↔(C−D)* (i.e., (5^N*,α*^)(D*)↔5^N*,αD*^) isotopic exchange is in favor of the latter. An explanation of the difference between the isotopic enrichments of 62 and 96 % is somewhat beyond the chemical accuracy of DFT, even though, interestingly, the barrier that leads to the (C^γ^‐D)* turns out to be higher than its (C^α^−D)* counterpart (11.5 vs. 6.5 kcal mol^−1^), in agreement with the observed lower isotopic enrichment at this position. Compound **6** was deuterated at the α‐position of the unsubstituted nitrogen atom with 99 % of isotopic enrichment accompanied by a slight deuterium incorporation on the hydroxymethyl group. The coordination of the nitrogen and the oxygen atom to the surface of the catalyst probably immobilizes the substrate in a certain conformation. This constraint would increase the activation barrier for C−H activation giving rise to a lowered deuterium incorporation at the C(sp^3^) center. Very high incorporations of deuterium were obtained for benzimidazoles **7** and **8**, which cannot be labelled by other HIE procedures to the best of our knowledge. Indeed, carbon centers in α and β positions of the nitrogen atoms of **7** were deuterated with 98 % and, respectively 81 % of isotopic enrichment. A DFT‐based investigation was also achieved for compound **7** in order to identify the key intermediate involved in the labelling of the β‐positions of the nitrogens (see Figure 7). Again, the reaction starts with a favorable σ‐donation of the nitrogen lone pair (7^N*^) combined with a further stabilization of the adduct as a result of a the C^α^−H (7^N*,αH*^) or C^β^−H (7^N*,βH*^) agostic interaction on a neighboring Ru atom. The C^β^−H HIE reaction involves a 5‐membered dimetallacycle and is favored by an exothermic (C−H)*↔(C)*(H)* reaction (7^N*,β*^ is more stable than 7^N*,βH*^ by −2.3 kcal mol^−1^) leading to the H/D exchange (see the discussion for compound **3**) and a relatively low C−H activation barrier (7^N*,βH≠*^: 5.6 kcal mol^−1^). As shown in Figure 7, the C^α^−H HIE reaction is characterized by a profile (in green), very similar to the profiles calculated for the two other H/D exchanges in α, described above for oxazole and imidazole substructures. It is noteworthy that the most efficient labelling process (98 %) goes first through an almost barrierless (C)*(H)* pathway and is then followed by the formation of the new (C‐D)* bond which also requires to overcome the lowest barrier (4.5 vs. 7.9 kcal mol^−1^, see Figure S3). Overall, the postulated C−H activation processes excelled in three key factors which characterize an efficient HIE at C(sp^2^) centers, that is, the formation of metallacycle intermediates, low barriers and their exothermicities.

Regarding **8**, all C−H bonds situated at the β‐positions of nitrogen atoms (C(sp^3^)‐H and C(sp^2^)‐H) were activated, leading to the incorporation of 3.7 deuterium atoms. Interestingly, the reaction conducted in deuterated THF did not lead to an increase of the isotopic enrichment whereas running the reaction twice resulted in a higher isotopic enrichment on the methyl [80].

### Labelling of triazole derivatives

**Figure 8:**
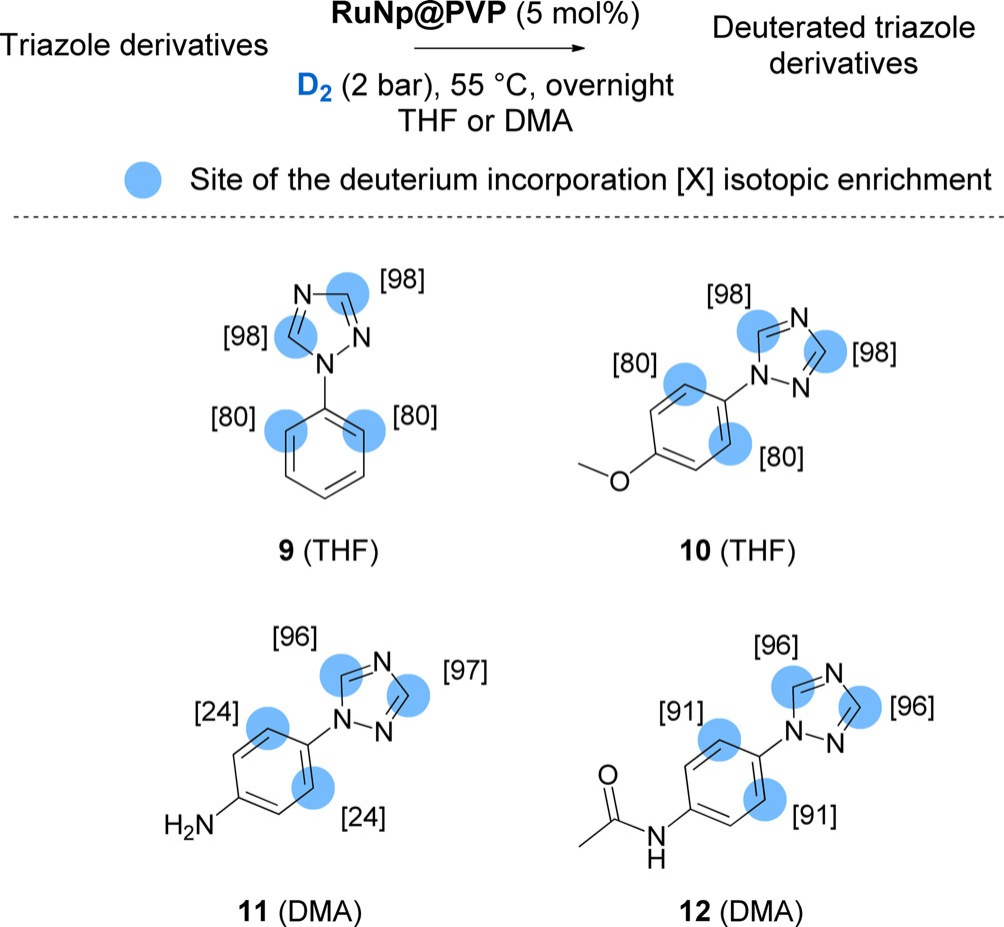
Deuteration of triazole containing molecules.

To the best of our knowledge, no general approach has been described so far for the labelling of the 1,2,4‐triazole scaffold using direct HIE. To our delight, our Ru nanocatalyst also promoted effective HIE on these heterocycles as demonstrated by the labelling of the differently functionalized compounds **9**–**12** (see Figure 8). Both, α and γ positions of coordinating nitrogen atoms were labelled leading to high deuterium uptakes per substructure unit (up 3.8D in a single run of catalysis). Similar to previous findings, it appeared very likely, that the underlying C−H activations for the labelling at the α positions of the triazolic scaffold pass through 4‐membered dimetallacyclic key intermediates (see Figure 4). In contrast to the targeted C_2_‐H and C_4_‐H of oxazoles, the C_3_−H and C_5_−H of the 1,2,4‐triazole unit display almost the same reactivities in most cases, which was reflected in identical isotopic enrichments on C_3_ and C_5_ of compounds **9**–**12** (see Figure 8). Logically, C−H activations in *ortho* of adjacent phenyl substituents must also proceed through 5‐membered metallacyclic key intermediates as in the case of 2‐phenylimidazole (see Figure 6). The efficient deuterium incorporation and the functional group tolerance for the methoxy unit in **10**, the amino unit in **11** and the acetamide unit in **12** supported the potential of this method to prepare stable isotopically labelled internal standards for LC/MS quantification. Indeed, in a later section, the applicability of RuNp@PVP was also confirmed for the deuterium and tritium labelling of more complex triazole based drugs and one agrochemical (see Figures 12 and 13).

### Labelling of carbazole derivatives

**Figure 9:**
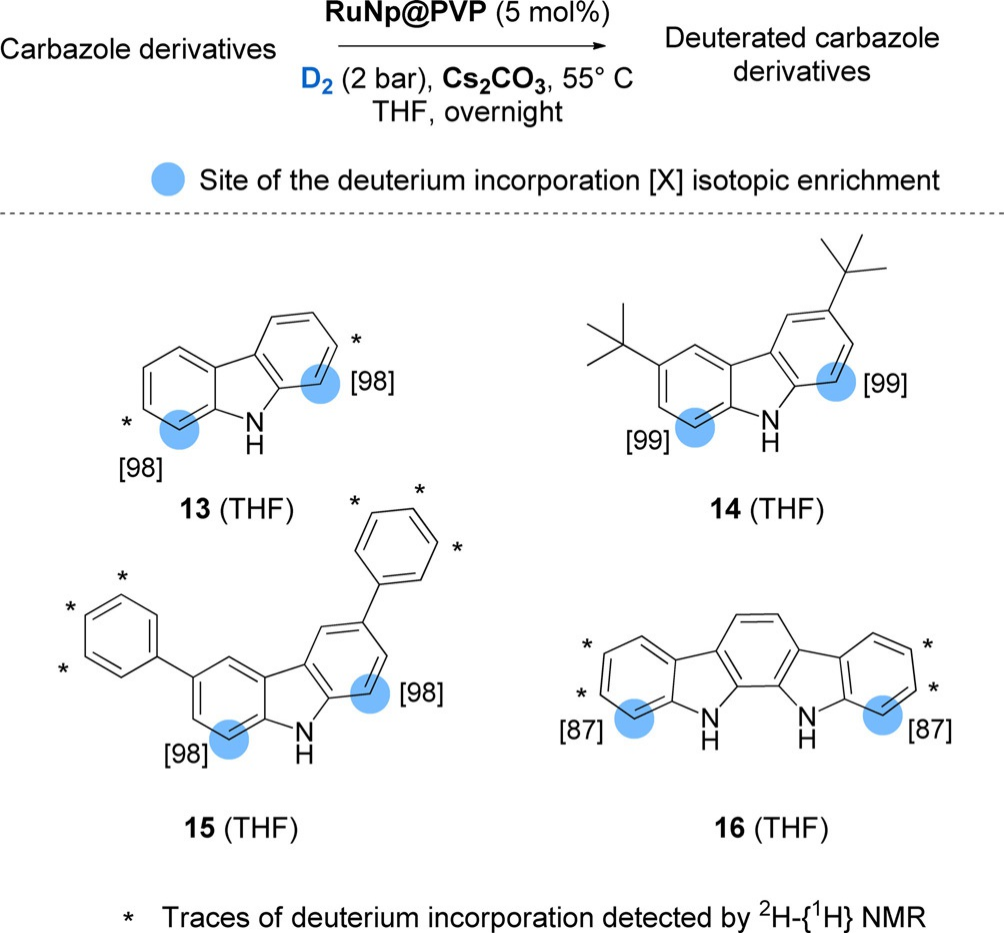
Deuteration of carbazole containing molecule.

**Figure 10:**
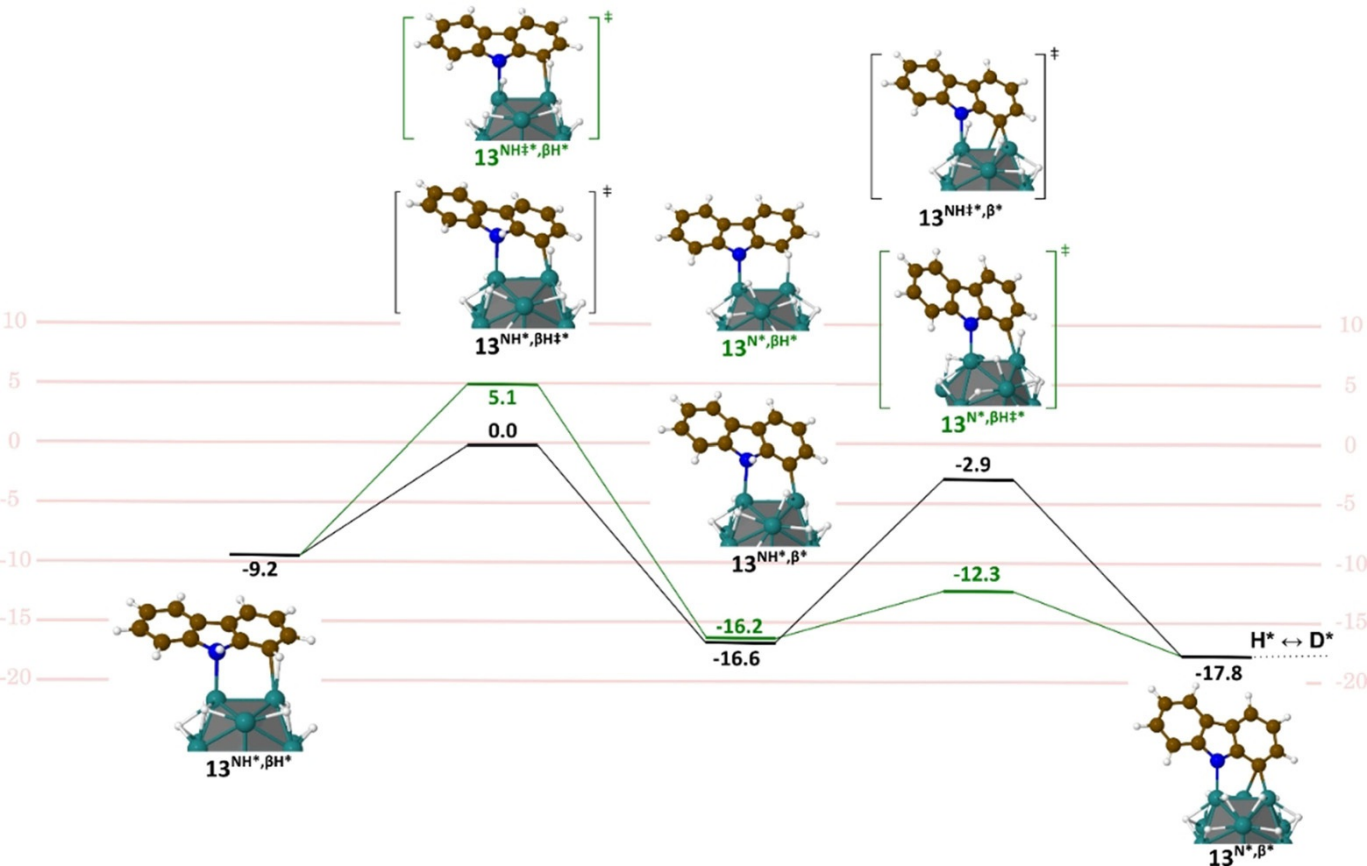
Energy diagram for the first steps of the Langmuir‐Hinshelwood‐type H/D exchange on **13**: on N and then in β (green pathway); in β and then on N (black pathway). Energies are given in kcal mol^−1^. See also Figure S4, where are given: the H/D exchange pathways directly on N; the π adsorption energy in β.

**Figure 11:**
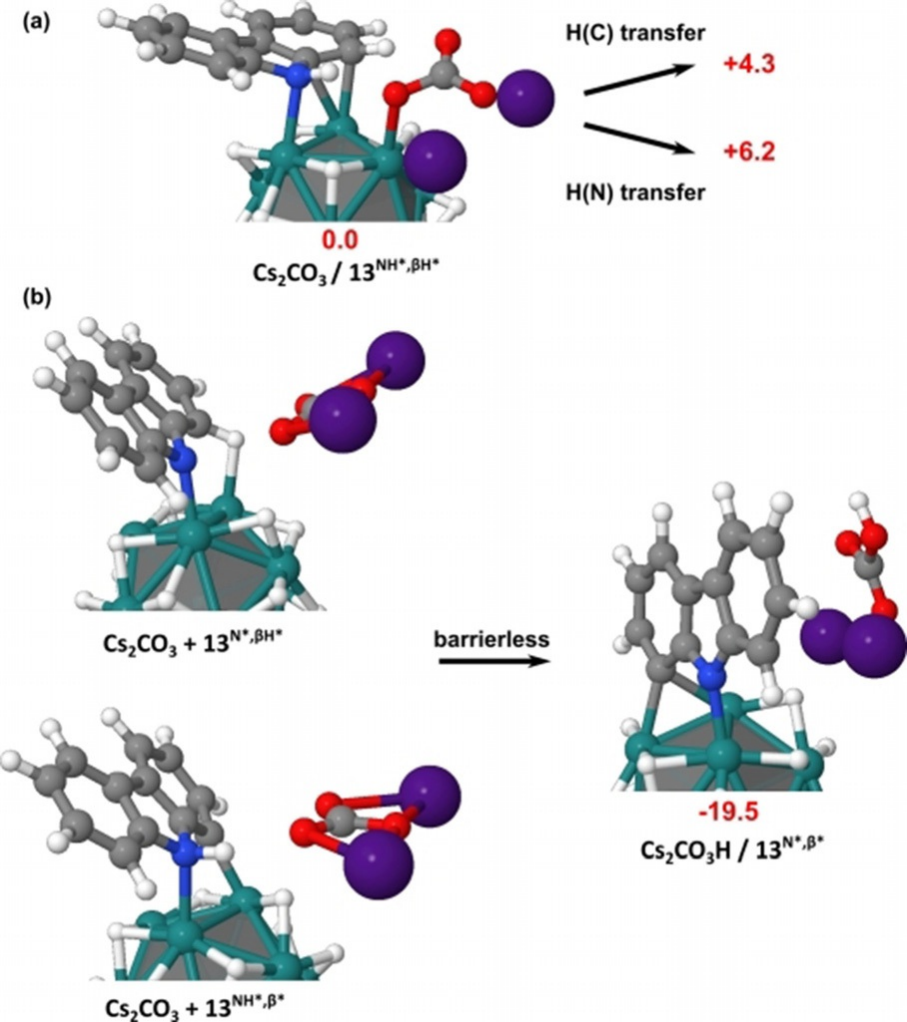
Ability of Cs_2_CO_3_ to coordinate to the catalyst surface and to adopt the role of a proton acceptor in the N−H and C−H activation step. a) H transfer from **13** ^NH*,βH*^ to cesium carbonate, b) H transfer to cesium carbonate after a preliminary C−H or N−H activation.

Up to now, only few methods for the deuteration of carbazoles have been reported using HIE. All of them employed harsh reaction conditions and led to unselective isotope incorporation.^18^ Through Ru nanocatalysis, compounds **13**–**16**, were deuterated with high isotopic enrichments at the β‐positions relative to the nitrogen using mild reaction conditions (see Figure 9). Interestingly, we found that the addition of a base enhanced the chemoselectivity and efficacy of the deuterium incorporation (see Supporting Information for results obtained without base). Indeed, without base, considerable amounts of reduced side‐products were formed, reducing the overall yield of isolated products. In contrast, performing the reaction with 1 equiv. of Cs_2_CO_3_, led to the recovery of deuterated compounds **13**–**16** in nearly quantitative yields. The higher chemoselectivity (i.e., the non‐formation of reduced side‐products) and efficacy (higher isotopic enrichments), observed for this transformation in the presence of a base, might be explained by the fostering of reaction intermediates leading to the isotope incorporation in β position of the nitrogen. To test this hypothesis, a DFT‐based investigation was first performed without base using carbazole as model compound. Different coordination modes of the substrate were considered (see Figure S4). The most favored involved the coordination of N and the simultaneous agostic interaction of C_1_‐H to give 13^NH*,βH*^ (see Figure 10). In this case, **13** is weakly coordinated to the surface, just by −9 kcal mol^−1^ (compared to the ca. −25 kcal mol^−1^ in the previous cases). This is consistent with a π‐coordination of the nitrogen atom instead of a σ‐coordination. Starting from 13^NH*,βH*^ two pathways could be stated: the N−H activation (13^N*,βH*^, green line) followed by the C_1_−H activation (13^N*,β*^) and the C_1_−H activation (13^NH*,β*^, black line) followed by the N−H activation (13^N*,β*^). These both exothermic reactions involve moderate barrier heights, but the C−H activation seems easier, whatever the pathway is. Interestingly, if the N−H activation takes place before (green line), the barrier of the C−H activation is significantly lowered by 5.3 kcal mol^−1^ (with an activation energy of 9.2 for TS 13^NH*,βH≠*^ vs. 3.9 kcal mol^−1^ for TS 13^N*,βH≠*^). In summary, the optimal reaction consists in the coordination of a 5‐membered dimetallacycle that partially breaks the conjugation. This preludes a possible HIE both on N and on C_1_ through two pathways that require to overcome a 17.8 kcal mol^−1^ apparent barrier and going on to 13^ND*,βD*^. Prompted by this data, we were eager to investigate the role of cesium carbonate (Cs_2_CO_3_) on these activation processes. The exploration of these pathways in the presence of a base is not an easy task for transition‐state search algorithms. It is, however, possible to give some energetic and structural clues regarding the role of Cs_2_CO_3_ on the C−H and N−H activation in compound **13**. As shown in Figure 11 a, Cs_2_CO_3_ can favorably interact with both the surface and 13^NH*,βH*^. Interestingly, this Cs_2_CO_3_/13^NH*,βH*^ complex is more stable by ca. 20 kcal mol^−1^ than the two species lying far away from each other on the surface. However, this is an energy minimum, that is, geometry optimizations do not involve a barrierless H transfer from C_1_ or N toward the base. Given that the species resulting from this transfer are thermodynamically less stable by ≈4 and ≈6 kcal mol^−1^, such pathways would not facilitate the deuterium incorporation. However, the possible role of the base after a first C−H or N−H activation by the metal surface of the RuNp should be also considered to clarify the situation. As shown in Figure 11 b, a Cs_2_CO_3_ molecule in the vicinity of the 13^NH*,β*^ or 13^N*,βH*^ intermediates spontaneously—and hence efficiently—abstracts the hydrogen of the N−H and C_1_−H bonds, respectively. The reaction is exothermic by ≈19.5 kcal mol^−1^. Given the C and N coordination on the surface, the H/D exchange can then occur from the resulting 13^N*,β*^ compound. To sum up, these mechanistic investigations have shown that after a prior C−H or N−H activation on the surface, a base could potentially facilitate the second rate‐determining steps (C−H and N−H activation) of the reactions leading to higher isotope incorporation.^19^

### Labelling of pharmaceuticals and other bioactive molecules

#### a) Deuterium labelling

**Figure 12:**
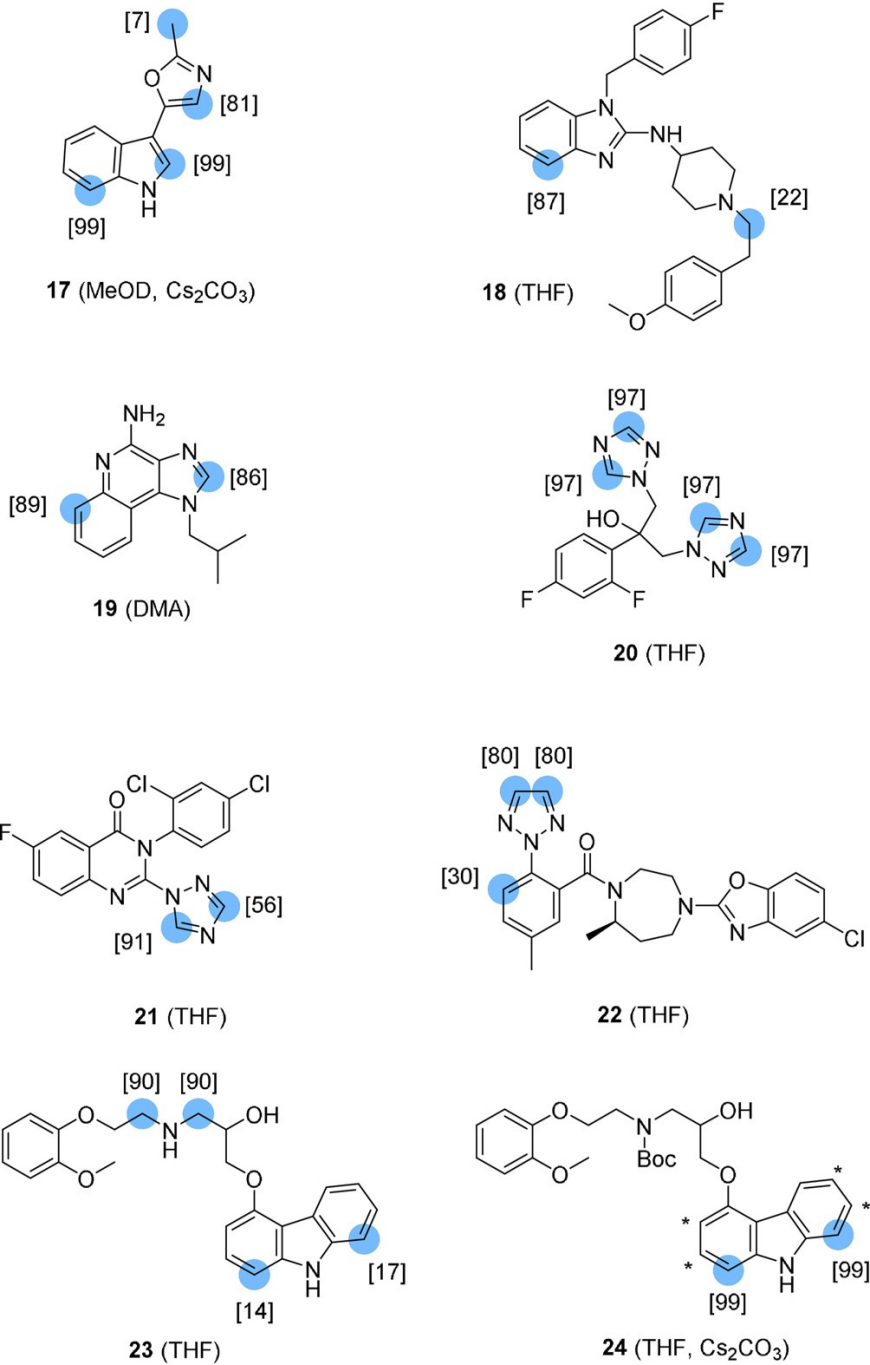
Deuterium labelling of complex pharmaceuticals (at 55 °C, overnight).

To illustrate the usefulness and the broad applicability of our RuNps catalyzed HIE, deuterium and tritium labelling of *N*‐heterocycle containing molecules of medical relevance and higher molecular complexity, was then considered (see Figure 12). As a first example, we have chosen the oxazole containing alkaloid, pimprinine **17** possessing anticonvulsant^20^ and antiviral activities.^21^ The use of RuNp@PVP (20 mol %) in CD_3_OD with 1 equiv of Cs_2_CO_3_, allowed a much higher deuteration of this sensitive molecule on its indole moiety than without Cs_2_CO_3_, analogous to the findings made with carbazoles **13**–**16**. Very good isotopic enrichment also occurred on the oxazole core (for the results without Cs_2_CO_3_, see Supporting Information). The benzimidazole containing drug astemizole **18** was used as a drug in the treatment of allergies.^22^ In the case of this complex molecule, deuteration occurs at the β‐position of the unsubstituted nitrogen of the benzimidazole moiety with an isotopic enrichment of 87 %. Furthermore, the analysis of ^2^H NMR data (@14T) also supported slight H/D exchange on one α methylene of the tertiary amine (22 %). Imiquimod **19** has a convincing efficacy against malignant melanoma.^23^ This poorly soluble molecule was successfully deuterated using DMA as solvent, highlighting the broad solvent compatibility of our method. The human antifungal drug fluconazole **20**, was selectively labelled on the 1,2,4‐triazole units leading to a very high isotope incorporation (almost 4D incorporated). This result repeatedly manifests the potential of our method for the preparation of stable isotopically labelled internal standards of a commercial drug for LC‐MS quantifications. This rapid deuterium labelling is insofar beneficiary as we consider that stable isotopically labelled internal standards of fluconazole were originally synthesized from deuterated precursors over four steps.^24^ The efficient and selective deuteration of the triazole substructure of the complex agricultural fungicide fluquinconazole **21** is representative for the functional group tolerance of the described catalytic transformation. Despite the presence of different carbon‐halogen bonds (1 C‐F and 2 C‐Cl), the highly deuterated product was obtained in high yield after a simple filtration through a C18 cartridge. The 1,2,3‐triazole drug suvorexant **22**, administered for insomnia treatment, is known to be labelled by hydrogen isotopes within other methods. Hence, H/D exchange occurs either on the C−H bonds of the 1,2,3‐triazole with a homogeneous Fe^0^ catalyst (leading to the incorporation of 0.6D) or in the *ortho* position of the adjacent phenyl with Crabtree's catalyst (theoretically limited to the incorporation of 1D).^13^ Here, the use of RuNps as catalyst allowed the deuterium labelling of both, the 1,2,3‐triazole group (1.6D) and the adjacent phenyl ring (0.3D), which gave a considerably higher deuterium incorporation as a whole, compared with the ones obtained using the previously described HIE procedures. Carvedilol **23** is a nonselective beta/alpha‐1 blocker used for treating congestive heart failure, left ventricular dysfunction and high blood pressure.^25^ Using carvedilol **23** as a substrate led to the deuteration of the secondary amine with a high isotopic enrichment due to the higher affinity of the alkylamine nitrogen compared to the one embedded in the carbazole moiety. By simply protecting the aliphatic amine with a *Boc*‐group and adding one equivalent of Cs_2_CO_3_, we were able to exclusively label the carbazole moiety (molecule **24**, see Figure 12) with a high isotopic enrichment (99 %). The possibility to modify the regioselectivity of the isotope incorporation on a such complex structure using simple protecting group strategies, highlights the versatility of our RuNps catalyzed HIE reactions for the synthesis of labelled drug compounds for metabolic studies.

#### b) Tritium labelling of pharmaceuticals

**Figure 13:**
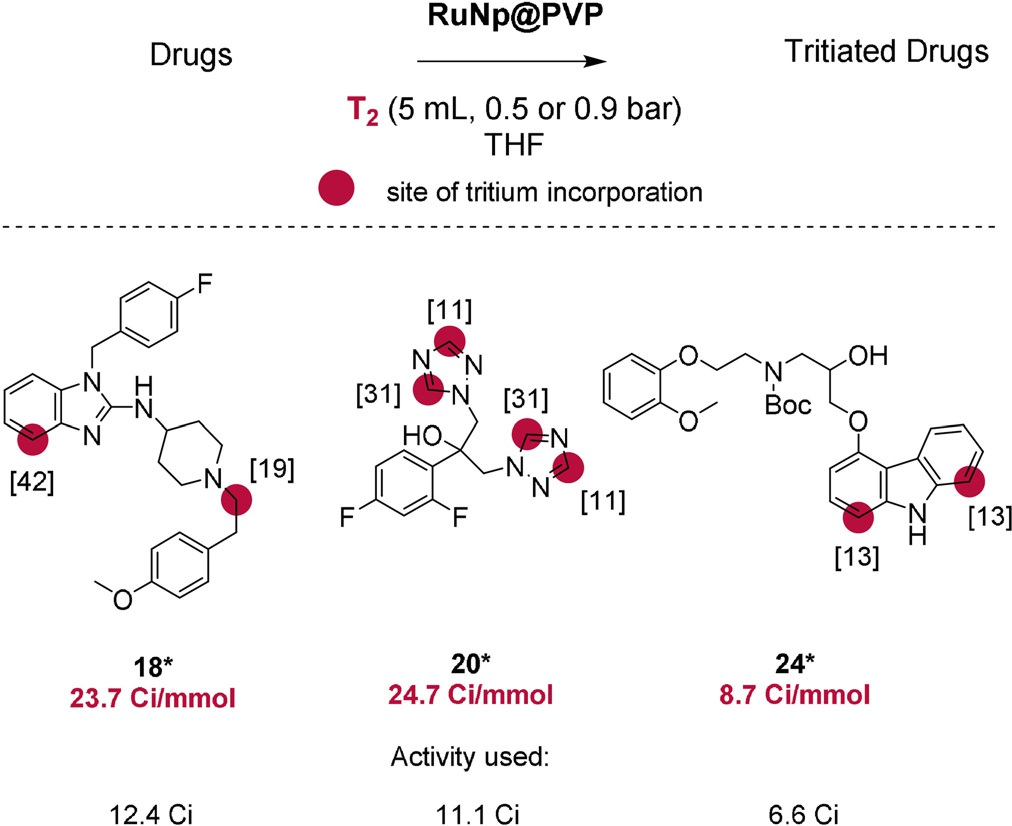
Tritium labelling of complex pharmaceuticals.

As a next step, we demonstrated that the late‐stage tritiation of complex pharmaceuticals can succeed through our catalytic method (see Figure 13). In this context, the use of T_2_ gas as isotopic source is a great advantage because it is the easiest raw material to handle for tritium labelling. Typically, reactions involving gaseous tritium were conducted using a subatmospheric pressure of T_2_ in order to minimize the risk of leakage and radioactive releases.

Up to date, the simplest way to label the *N*‐heterocyclic substructures of astemizole and carvedilol with tritium was the transformation of halogenated precursors with Pd/C and T_2_, a procedure that consisted of several other reaction steps.^26^ With our method, radioactive analogues of astemizole 18*, fluconazole 20*, and *N*‐Boc‐protected carvedilol 24* were obtained with satisfying molar activities and in high yields using subatmospheric tritium gas pressures. It is noteworthy that in every case (18*, 20*, 24*) tritium labelling took place on positions which are not major metabolism sites.^27^ The obtained molar activities of 18* and 20* (24 Ci mmol^−1^), under a tritium gas pressure of ≈900 mbar, clearly outperformed the prerequisites for ADME studies (10–20 Ci mmol^−1^). Performing the radiolabelling under a lower tritium gas pressure of ≈500 mbar, tritiated 24* could be obtained with a tritium incorporation of ≈9 Ci mmol^−1^, which was still within an acceptable range.

## Conclusion

In this paper, we demonstrated that the application of Ru nanocatalysts can provide efficient deuteration and tritiation of different classes of heterocycles in complex molecules using D_2_ or T_2_ gas as isotopic sources. For the labelling of oxazole and imidazole substructures, this work represents a significant advance in practicality compared to previously described methods, with notably higher isotope uptakes, a broader substrate scope and a higher solvent compatibility. This method represents also the unique approach allowing the selective deuterium and tritium incorporation on 1,2,4‐triazole and carbazole substructures using direct hydrogen isotope exchange. In terms of application, the high incorporation of deuterium atoms allows the synthesis of deuterated internal standards for LC/MS quantification and the efficacy of the catalytic process permits the preparation of complex radiolabelled drugs owning high specific activities by using a subatmospheric pressure of T_2_ gas. From a more fundamental point of view, the detailed theoretical calculations have deciphered the processes of C−H bond activation at α, β‐ and γ‐positions of coordinating nitrogen atoms occurring at the surface of Ru clusters. All the key intermediates involved in such reactions were disclosed: four‐membered dimetallacycle for the C−H activation in α, five‐membered dimetallacycle for the C−H activation in β and five‐membered metallacycle for the C−H activation in γ. Energy profiles qualitatively account for the experimental isotopic enrichment rates. C−H activation equilibria or C−D recombination equilibria exhibit characteristic patterns that drive the overall reaction. In addition to the fact that this study paves the way for an easier access to valuable isotopically labelled complex molecules, the theoretical aspects addressed will be useful for future developments of more efficient and selective nanocatalysts for C−H activations.

## Conflict of interest

The authors declare no conflict of interest.

## Supporting information

As a service to our authors and readers, this journal provides supporting information supplied by the authors. Such materials are peer reviewed and may be re‐organized for online delivery, but are not copy‐edited or typeset. Technical support issues arising from supporting information (other than missing files) should be addressed to the authors.

Supplementary
