# Supplementary material for: Hydrogen Isotope Exchange Catalyzed by Ru Nanocatalysts: Labelling of Complex Molecules Containing N‐Heterocycles and Reaction Mechanism Insights
Source: Chemistry. 2020 Mar 9;26(22):4988–96. doi: 10.1002/chem.201905651 (PMC7187376; doi:10.1002/chem.201905651)
Supplement: Supplementary file 1 — Supplementary [file CHEM-26-4988-s001.pdf]

Chemistry–A European Journal

Supporting Information

Hydrogen Isotope Exchange Catalyzed by Ru Nanocatalysts: Labelling of Complex Molecules Containing *N*-Heterocycles and Reaction Mechanism Insights

Viktor Pfeifer,[a] Marie Certiat,[b] Donia Bouzouita,[b] Alberto Palazzolo,[a] Sébastien Garcia-Argote,[a] Elodie Marcon,[a] David-Alexandre Buisson,[a] Philippe Lesot,[c] Laurent Maron,[b] Bruno Chaudret,[b] Simon Tricard,[b] Iker del Rosal,[b] Romuald Poteau,*[b] Sophie Feuillastre,[a] and Grégory Pieters*[a]

Table of Content

Table of Content 1
General Procedures 2
Syntheses of compounds 3
Synthesis of Pimprinine 3
Synthesis of 4'-(1,2,4-Triazol-1-yl)acetanilide 6
Synthesis of *N*-Boc-carvedilol 8
H/D exchange reactions 10
2,5-Diphenyloxazole **1** 10
5-(4-Methylphenyl)-oxazole **2** 14
4-(Oxazol-5-yl)aniline **3** 18
5-(4-Methoxyphenyl)-oxazole-4-carboxylic acid **4** 21
2-Phenylimidazole **5** 25
(1-Ethyl-imidazol-2-yl)methanol **6** 29
Benzimidazole **7** 32
2-Methyl-benzimidazole **8** (first deuteration run) 35
2-Methyl-benzimidazole **8** (second deuteration run) 38
1-Phenyl-1*H*-1,2,4-triazole **9** 41
1-(4-Methoxyphenyl)-1*H*-1,2,4-triazole **10** 44
4-(1*H*-1,2,4-Triazol-1-yl)aniline **11** 48
4'-(1,2,4-Triazol-1-yl)acetanilide **12** 52
Deuteration of carbazoles with RuNp@PVP and Cs_2CO_3 55
Carbazole **13** 55
3,6-Di-*tert*-butylcarbazole **14** 59
3,6-Diphenylcarbazole **15** 62
11,12-Dihydroindolo[2,3-a]carbazole **16** 66
Deuteration of carbazoles without Cs_2CO_3 69
Carbazole **13’** 69
3,6-Di-*tert*-butylcarbazole **14’** 72
3,6-Diphenylcarbazole **15’** 77
11,12-Dihydroindolo[2,3-a]carbazole **16’** 81
Deuterations of Drugs and other bioactive molecules 85
Pimprinine **17I** (without base) 85
Pimprinine **17II** (with base) 89

Astemizole **18**....92
Imiquimod **19**....96
Fluconazole **20**....99
Fluquinconazole **21**....103
Suvorexant **22**....106
Carvedilol **23**....110
N-Boc-carvedilol **24**....114
Tritiations of Drugs....118
Tritiation of astemizole **18***....118
Tritiation of fluconazole **20***....120
Tritiation of *N*-boc-carvedilol **24***....122
Reaction pathways and syntax of the labels....125

General Procedures

Reagents and instrumentation. Ruthenium nanoparticles in a polyvinylpyrolidone matrix (RuNp@PVP, 7wt% metallic ruthenium) were synthesized as described elsewhere(1) and stored in a glove box under an argon atmosphere (O_2 < 0.1 ppm). Nanoparticle synthesis and catalysis were carried out in Fischer-Porter glassware under argon. Commercially available substrates were used without further purification. THF was dried over sodium and benzophenone and distilled before use. DMA was stored in a Schlenck tube over molecular sieves (4Å, previously activated by microwave treatment). CD_3OD was used without further purification. ^1H NMR (400 MHz), ^{13}C NMR (100 MHz) & ^3H NMR (427 MHz) spectra were recorded on a (400 MHz) Bruker Avance spectrometer. Proton-decoupled deuterium (^2H-{^1H}) 1D NMR (92 MHz) spectra were recorded on a 14.1 T (600 MHz) Bruker Avance II NMR spectrometer equipped with a 5-mm selective ^2H observe cryogenic probe. Proton signals were eliminated using the WALTZ-16 CPD sequence. Chemical shifts are reported in parts per million (ppm) downfield from residual solvent peaks and coupling constants are reported in Hertz (Hz). Splitting patterns are designated as singlets (s), doublets (d) or triplets (t). Splitting patterns that could not be interpreted or easily visualized are designated as multiplets (m). Electrospray (ESI) mass spectra were recorded using a Waters ZQ 2000 LCMS System. GC-MS analysis was carried out using a Waters GCT Premier TOF Mass spectrometer equipped with a DCI probe tip.

H/D exchange quantification. Deuterium incorporation was quantified by the decrease of ^1H-NMR integral intensities at the specified positions compared to the starting material. Integral intensities were calibrated against hydrogen signals that did not undergo H/D-exchange. Incorporations of deuterium and tritium could be further determined by ^2H- and ^3H-NMR. Isotopic labeling of complex molecular structures as drugs in particular requires robust NMR analyses for properly determining both the degree of regioselectivity, the isotopic enrichment rate but also the final product purity. Variations observed on the ^1H NMR spectra of compounds with and without isotope provides first indications on the reaction efficiency, but the overlapping of fine structures when the isotopic incorporation is not total can obscure the analysis of results. Consequently, all deuterated compounds of this study have been analyzed by proton-decoupled deuterium (^2H-{^1H}) NMR. The simplicity of ^2H-{^1H} 1D spectra (no J(^2H-^1H) couplings and J(^2H-^2H) couplings smaller than the linewidths) guarantees a simple analysis of results (sum of singulets). Besides as ^2H-{^1H} spectra were recorded on a 14.1 T NMR (600 MHz) spectrometer equipped with a ^2H cryogenic probe, we benefit a confortable dispersion of ^2H signals (in Hz), as well as a significant sensitivity able to easily detect the major and minor sites of incorporation and any traces of deuterated impurities in the sample, even with small amount of analyte(2).

Mass spectrometry quantification was performed by subtraction of the mean molecular masses of the product and substrate isotopologue clusters in order to eliminate the contribution of the natural isotope abundance to the total mass.

General Procedure for H/D exchanges. A 100 mL Fischer–Porter bottle was equipped with a magnetic stir bar and charged with RuNp@PVP in a glove box. The substrate was dissolved in the appropriate solvent; the solution was then degassed and added to the catalyst in the Fischer–Porter bottle under an argon atmosphere. Argon was removed under reduced pressure and the Fischer-Porter glassware was flushed with D_2 gas under stirring. The degassing-flushing cycle was repeated twice. The reaction mixture was then stirred at 50°C (sand bath) under D_2 (2 bar) for 24 hours. Amounts of substrates, catalyst, used solvents, work-up and purification procedures are individually indicated in all cases.

DFT calculations. The Ru_{13} model has previously been published and detailed elsewhere.[0] It has been successfully applied to rationalize the enantiospecific C-H activation using ruthenium nanocatalysts in terms of reaction pathways. It was shown that similar thermodynamic and activation energies are found on this model and on a larger hydrogenated 1nm Ru_{55} model. The surface of this Ru_{13} model is covered with 17 hydrides per surface ruthenium atom, *i.e.* 1.4 H/surface Ru atom, a usually measured coverage value on RuNPs.[0] DFT calculations were done with the Vienna ab initio simulation package, VASP.[0] spin polarized DFT; exchange-correlation potential approximated by the generalized gradient approach proposed by Perdew, Burke, and Ernzerhof (PBE);[0] projector augmented waves (PAW) full-potential reconstruction;[0] PAW data sets for Ru atoms treating the 4*p*, 4*d* and 5*s* states (14 valence electrons); kinetic energy cutoff: 500 eV; Γ-centered calculations;[0] Gaussian smearing of 0.02 eV width; geometry optimization threshold: residual forces on any direction less than 0.02 eV/Å; supercell size set to ensure a vacuum space of *ca.* 14 Å between periodic images of metal clusters, 30.5Å x 30.5Å x 31Å).

Reaction barriers were estimated by the climbing image nudge elastic band (CINEB) method;[0] spring force between images: 5 eV; force tolerance of 0.02 eV/Å. The harmonic vibrational modes were systematically calculated for in order to distinguish minima and saddle points by using the dynamical matrix code implemented in VASP as well as the VASPTST tools also developed by Henkelman's group.

REFERENCES

(1) C. Pan, K. Pelzer, K. Philippot, B. Chaudret, F. Dassenoy, P. Lecante, M.-J. Casanove, *J. Am. Chem. Soc.* **2001**, *123*, 7584-7593

(2) H. Kovacs, D. Moskau, M. Spraul, Cryogenically cooled probes, a leap in NMR technology, *Prog. Nucl. Magn. Reson. Spectrosc.* **2005**, 55, 131–155

(3) C. Taglang; L. M. Martínez-Prieto; I. del Rosal; L. Maron; R. Poteau; K. Philippot; B. Chaudret; S. Perato; A. Sam Lone; C. Puente; C. Dugave; B. Rousseau & G. Pieters, Enantiospecific C-H Activation Using Ruthenium Nanocatalysts, *Angew. Chem., Int. ed. Eng*. **2015**, *54*, 10474-10477.

(4) García-Antón, J.; Axet, M. R.; Jansat, S.; Philippot, K.; Chaudret, B.; Pery, T.; Buntkowsky, G.; Limbach, H. H. Reactions of Olefins with Ruthenium Hydride Nanoparticles: NMR Characterization, Hydride Titration, and Room-Temperature C-C Bond Activation. *Angew. Chem. Int. Ed.* **2008**, *47*, 2074-2078.

(5) (a) Kresse, G.; Fürthmuller, J. Efficient Iterative Schemes for Ab Initio Total-Energy Calculations Using a Plane-Wave Basis Set. *Phys. Rev. B* **1996**, *54*, 11169–11186; (b) Kresse, G.; Fürthmuller, J. Efficiency of Ab Initio Total Energy Calculations for Metals and Semiconductors Using a Plane-Wave Basis Set. *Comput. Mater. Sci*., **1996**, *6*, 15–50.

(6) Perdew, J. P.; Burke, K.; Ernzerhof, M. Generalized Gradient Approximation Made Simple. *Phys. Rev. Lett*. **1996**, *77*, 3865–3868.

(7) (a) Blöchl, P. E. Projector Augmented-Wave Method. *Phys. Rev. B*. **1994**, *50*, 17953–17979; (b) Kresse, G.; Joubert, D. From Ultrasoft Pseudopotentials to the Projector Augmented-Wave Method. *Phys. Rev. B*. **1999**, *59*, 1758–1775.

(8) Monkhorst, J. D.; Pack. H. J. Special Points for Brillouin-Zone Integrations. *Phys. Rev. B*. **1976**, *13*, 5188–5192.

(9)(a) G. Henkelman; B. P. Uberuaga; H. Jonsson; A climbing image nudged elastic band method for finding saddle points and minimum energy paths; *J. Chem. Phys*. **2000**; *113*; 9901-9904; (b) G. Henkelman; H. Jonsson; Improved tangent estimate in the nudged elastic band method for finding minimum energy paths and saddle points; *J. Chem. Phys.* **2000**; *113*; 9978-9985; (c) D. Sheppard; R. Terrell; G. Henkelman; Optimization Methods for Finding Minimum Energy Paths; *J. Chem. Phys*. **2008**; *128*; 134106-1-10.

Syntheses of compounds

Synthesis of Pimprinine

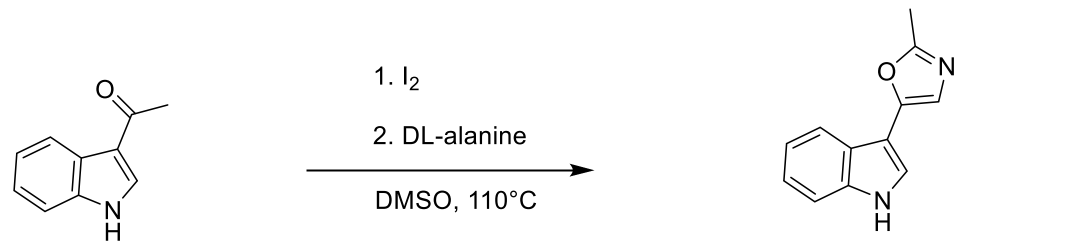

Chemical Formula: $C_{12}H_{10}N_2O$

Pimprinine was synthesized after a literature procedure.[9] 3-Acetylindole (1.6g, 10mmol) and I_2 (5.1g, 20mmol) were dissolved in DMSO (60mL) and stirred for 45min at 110°C. DL-alanine (1.8g, 20mmol) was added to the reaction mixture and the reaction was stirred for another 15min at 110°C. After cooling down to room temperature the reaction mixture was poured into a Na_2SO_3 solution (100mL, 10% in H_2O dist.). The aqueous phase was extracted three times with EtOAc (3 x 50mL). The combined organic phases were dried over $MgSO_4$ and the solvent was removed under vacuum. The crude product was purified over C18 functionalized SiO_2. Pimprinine was eluted with 1:1 MeOH : H_2O (0.1% TFA).

Yield: 160mg, 8%, light yellow solid

^{1}H NMR (400 MHz, Methanol-*d₄*): δ 7.80 – 7.75 (m, 1H), 7.59 (s, 1H), 7.46 – 7.40 (m, 1H), 7.23 – 7.12 (m, 3H), 2.51 (s, 3H).

^{13}C-{^{1}H}NMR (100 MHz, Methanol-*d₄*): δ 160.8, 150.1, 138.2, 125.3, 123.7, 123.4, 121.3, 120.4, 119.2, 112.8, 105.5, 13.5.

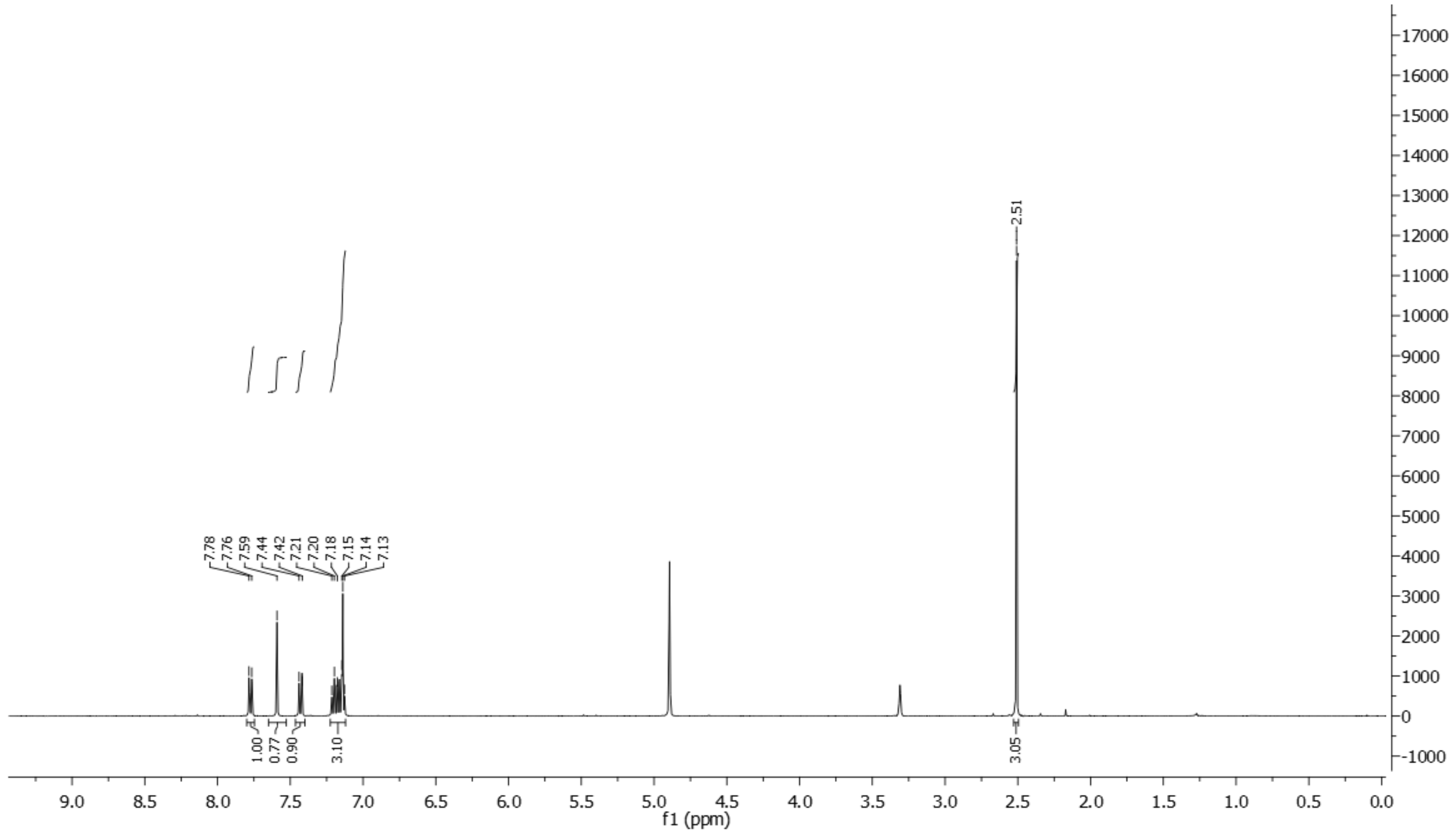

^{1}H-NMR spectrum of Pimprinine

9 Xiang, J.; Wang, J.; Wang, M.; Meng, X.; Wu, A. *Tetrahedron* **2014**, *70*, 7470-7475.

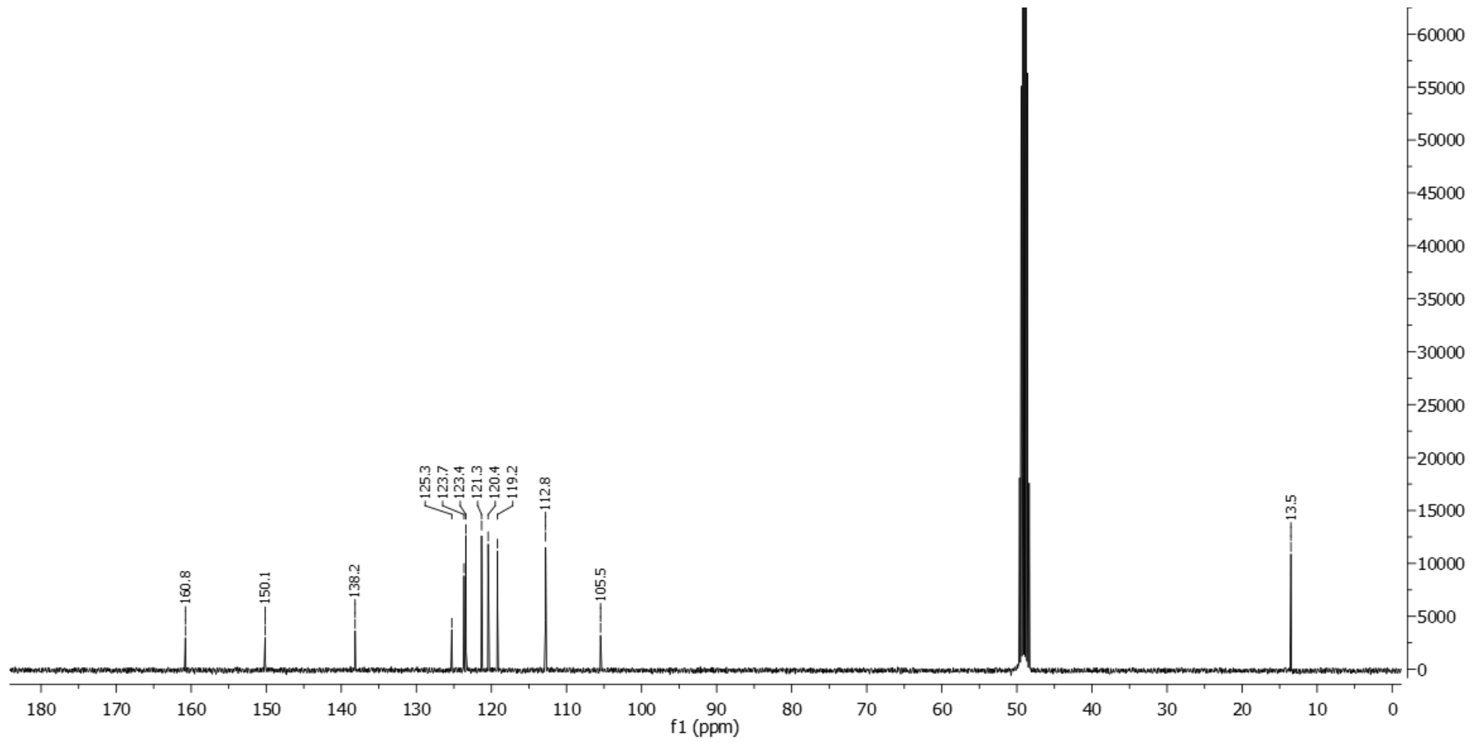

^{13}C-NMR spectrum of Pimprinine

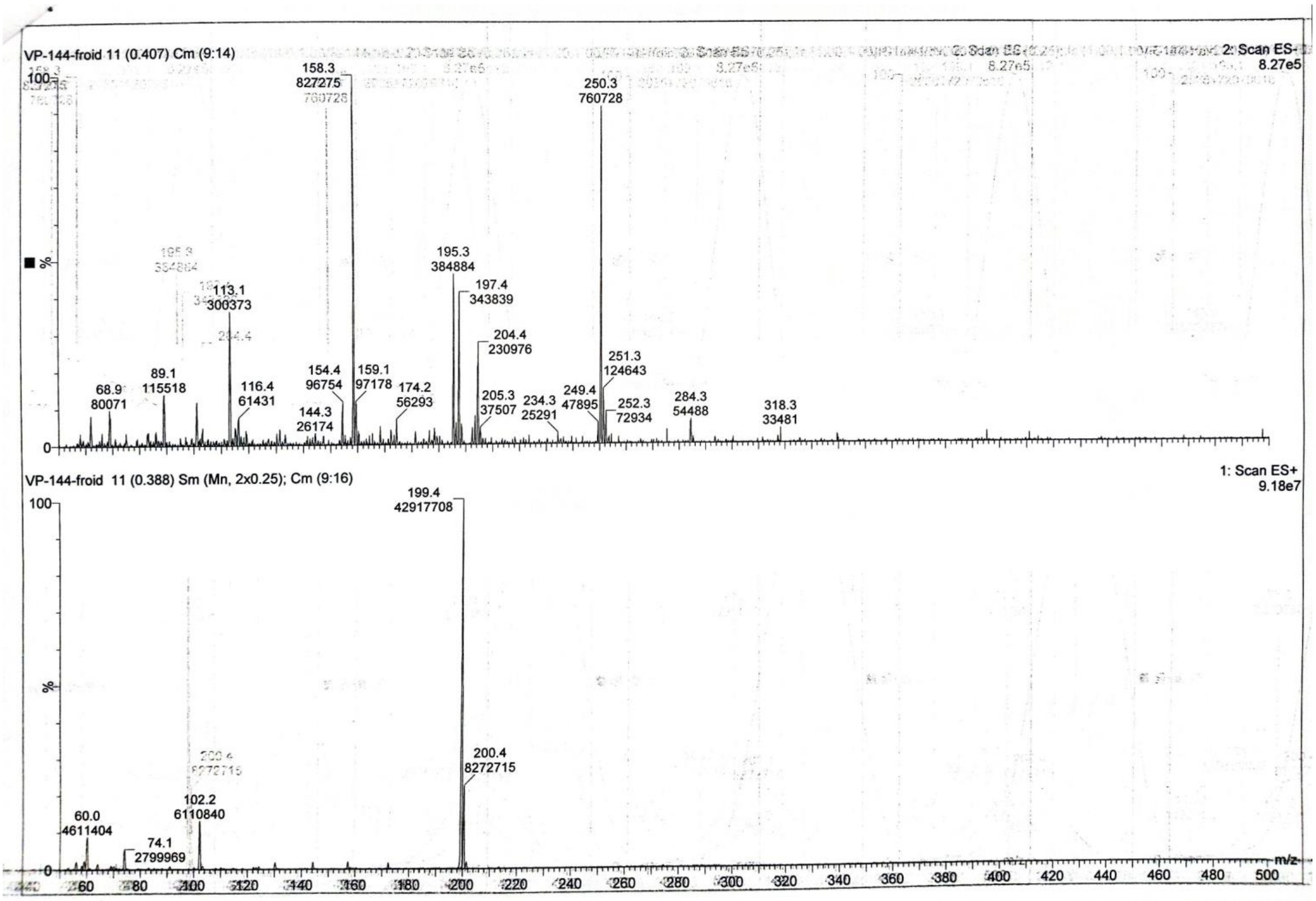

ESI spectrum of Pimprinine

Synthesis of 4'-(1,2,4-Triazol-1-yl)acetanilide

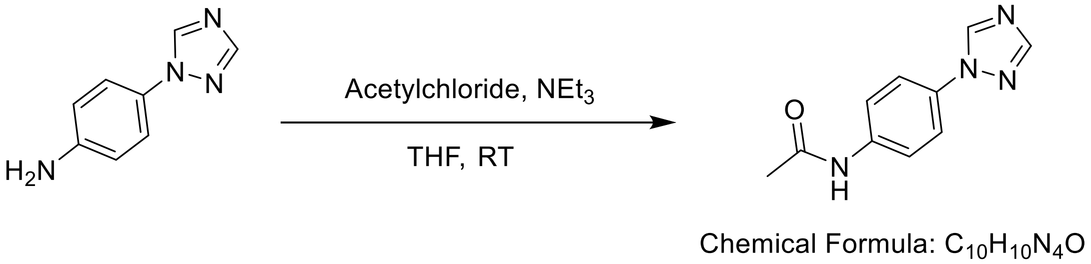

4-(1*H*-1,2,4-Triazol-1-yl)aniline (150mg, 937µmol) was dissolved in THF (5mL) and NEt_3 (130µL, 937µmol) was added under stirring at RT. Acetylchloride (65µL, 937µmol) was added and the reaction mixture was stirred for 1h at room temperature. The reaction mixture was poured into H_2O dist. (100mL). The aqueous phase was extracted three times with DCM (3 x 50mL). The solvent was removed under vacuum and the crude product was recrystallized from DCM/MeOH (3:1).

Yield: 65.0mg, 34%, white solid

^{1}H NMR (400 MHz, Methanol-*d₄*): δ 9.02 (s, 1H), 8.14 (s, 1H), 7.77 – 7.71 (m, 4H), 2.15 (s, 3H).

^{13}C-{^{1}H}NMR (100 MHz, Methanol-*d₄*): δ 171.8, 152.7, 142.8, 140.2, 134.0, 121.8, 121.6, 23.9.

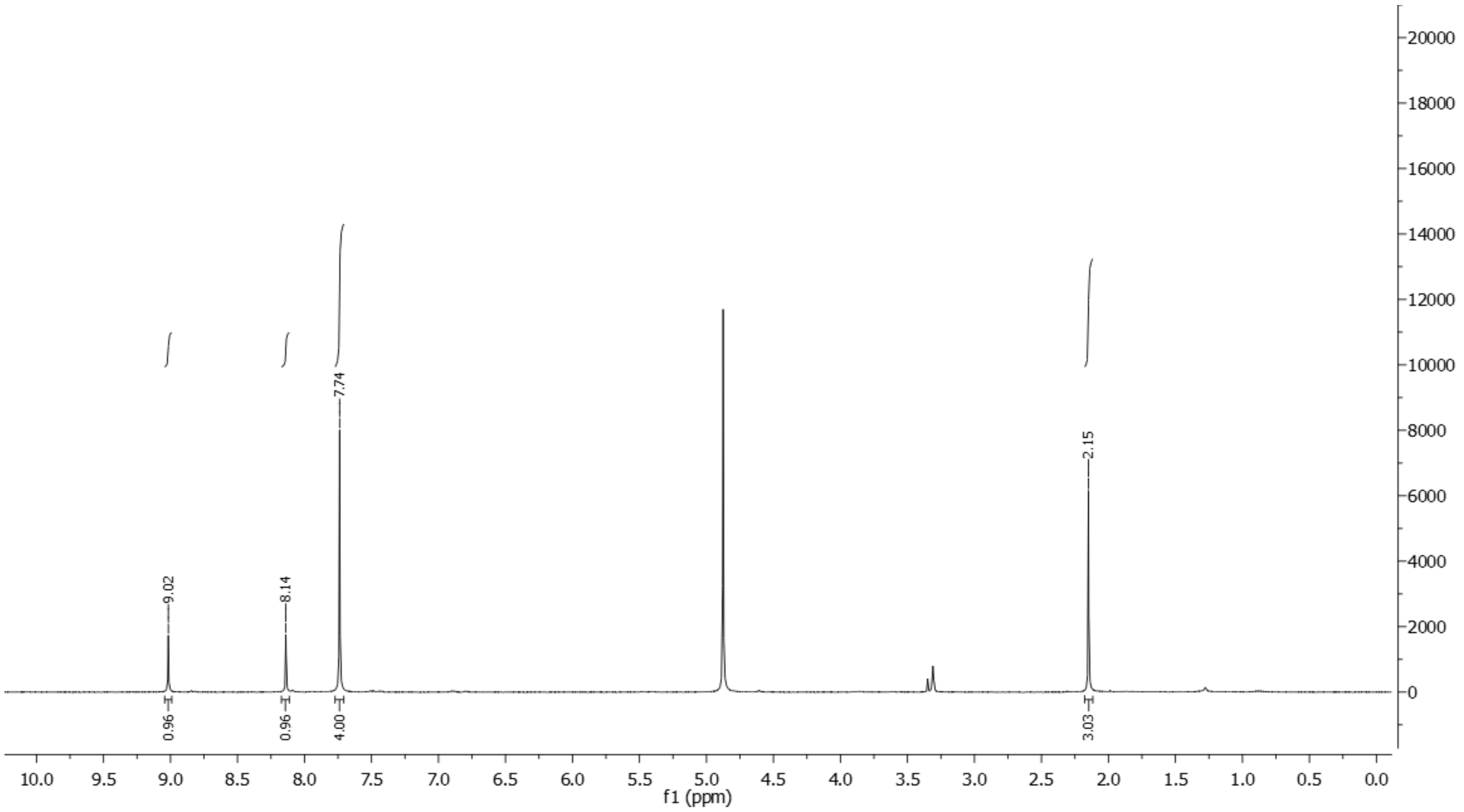

^{1}H-NMR spectrum of 4'-(1,2,4-Triazol-1-yl)acetanilide

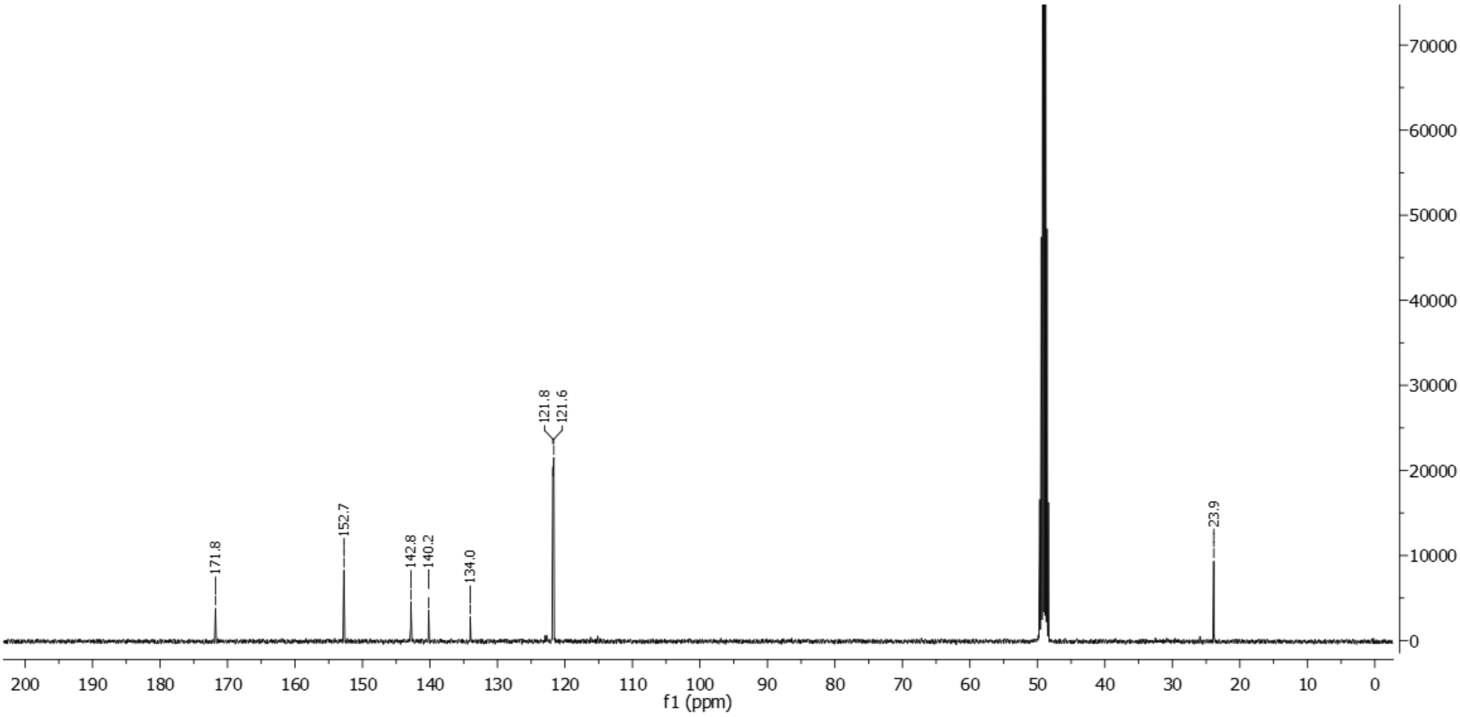

^{13}C-NMR spectrum of 4'-(1,2,4-Triazol-1-yl)acetanilide

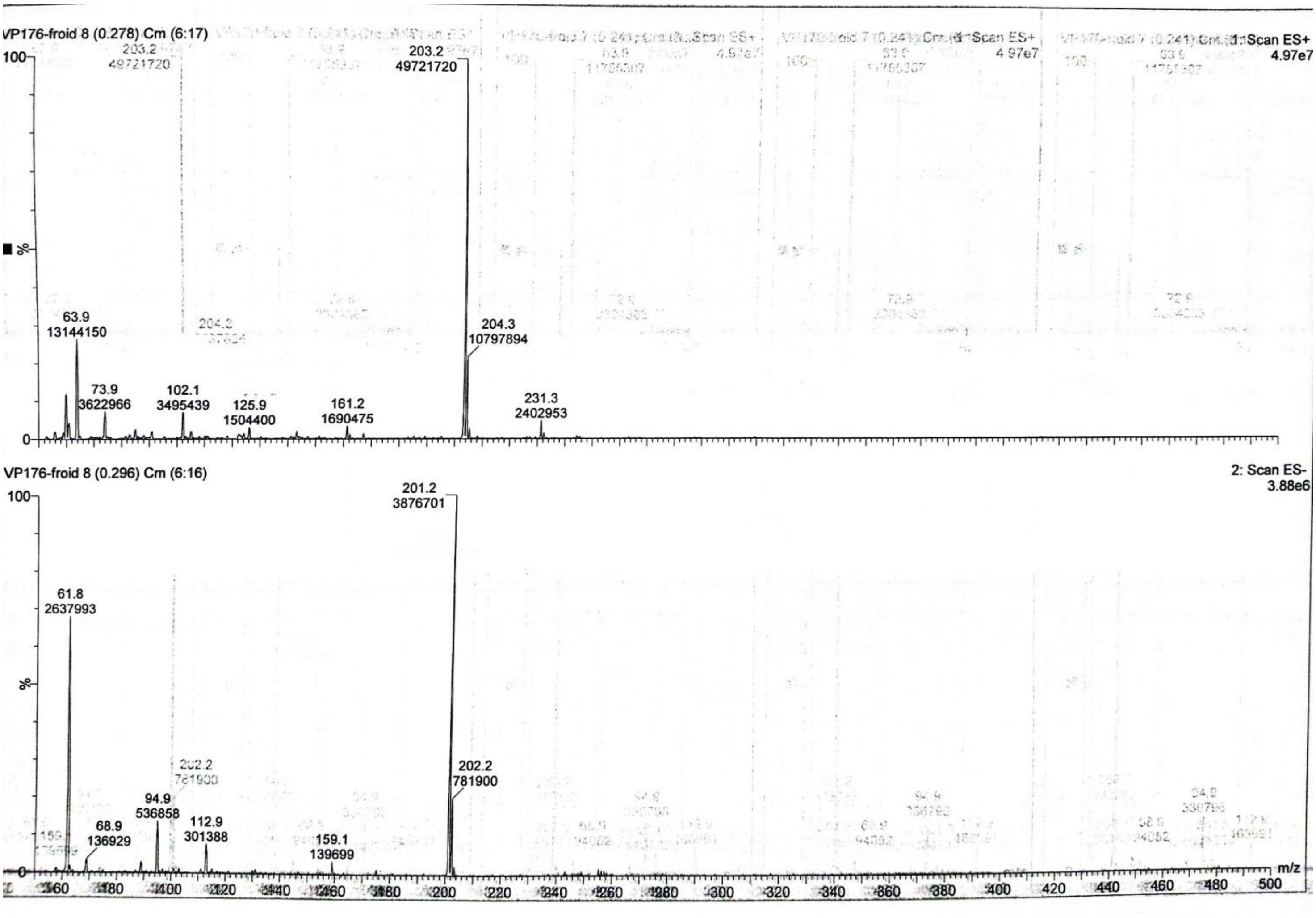

ESI-spectrum of 4'-(1,2,4-Triazol-1-yl)acetanilide

Synthesis of *N*-Boc-carvedilol

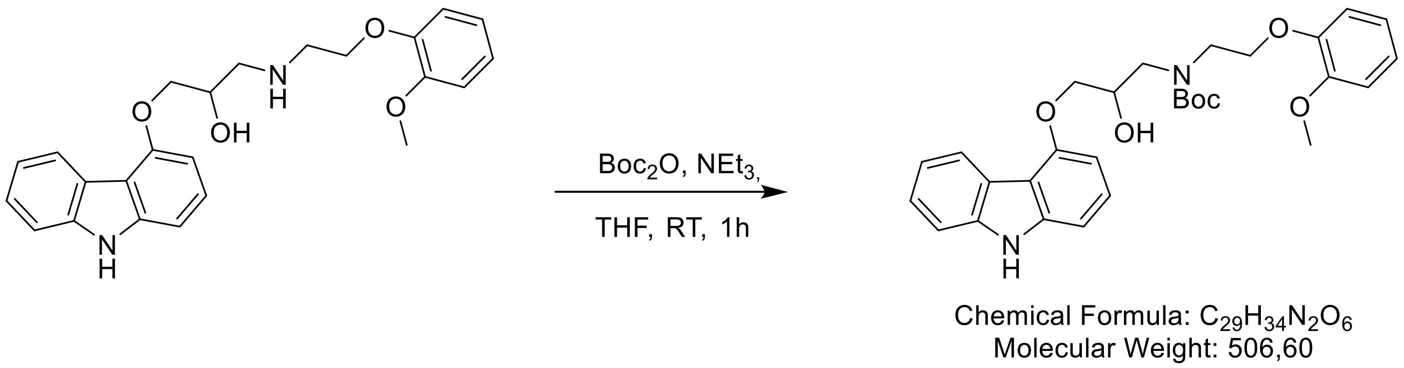

Carvedilol (1.0g, 2.5mmol) was dissolved in THF (20mL) and NEt_3 (377µL, 2.70mmol, 1.1eq) was added under stirring. A solution of di-*tert*-butyl dicarbonate (540mg, 2.50mmol, 1eq) in THF (15mL) was added slowly under stirring to the reaction mixture at room temperature. The reaction mixture was stirred 1h at room temperature and poured on a SiO_2 column. Elution was carried out with EtOAc/MeOH (50:3) and the solvent was removed under vacuum. The crude product was dissolved in THF (5mL) and precipitated in *n*-pentane (400mL).

Yield: 1.2g, 96%, white solid

^{1}H NMR (400 MHz, Acetone-*d$_6$*): δ 10.31 (bs, NH), 8.55 – 8.40 (m, 1H), 7.54 – 7.46 (m, 1H), 7.40 – 7.28 (m, 2H), 7.22 – 7.11 (m, 2H), 6.99 – 6.80 (m, 4H), 6.77 – 6.68 (m, 1H), 4.80 – 4.49 (m, 2H), 4.38 – 4.15 (m, 4H), 4.00 – 3.92 (m, 1H), 3.88 – 3.79 (m, 2H), 3.78 – 3.66 (m, 4H), 1.54 – 1.43 (m, 9H).

^{13}C-{^{1}H}NMR (100 MHz, Acetone-*d$_6$*): δ 157.1, 156.2, 150.7, 149.3, 142.4, 140.1, 127.3, 125.4, 124.1, 123.3, 122.2, 121.6, 119.7, 114.6, 113.3, 110.9, 104.7, 101.4, 80.3, 71.2, 70.4, 68.3, 56.0, 53.1, 49.2, 28.6.

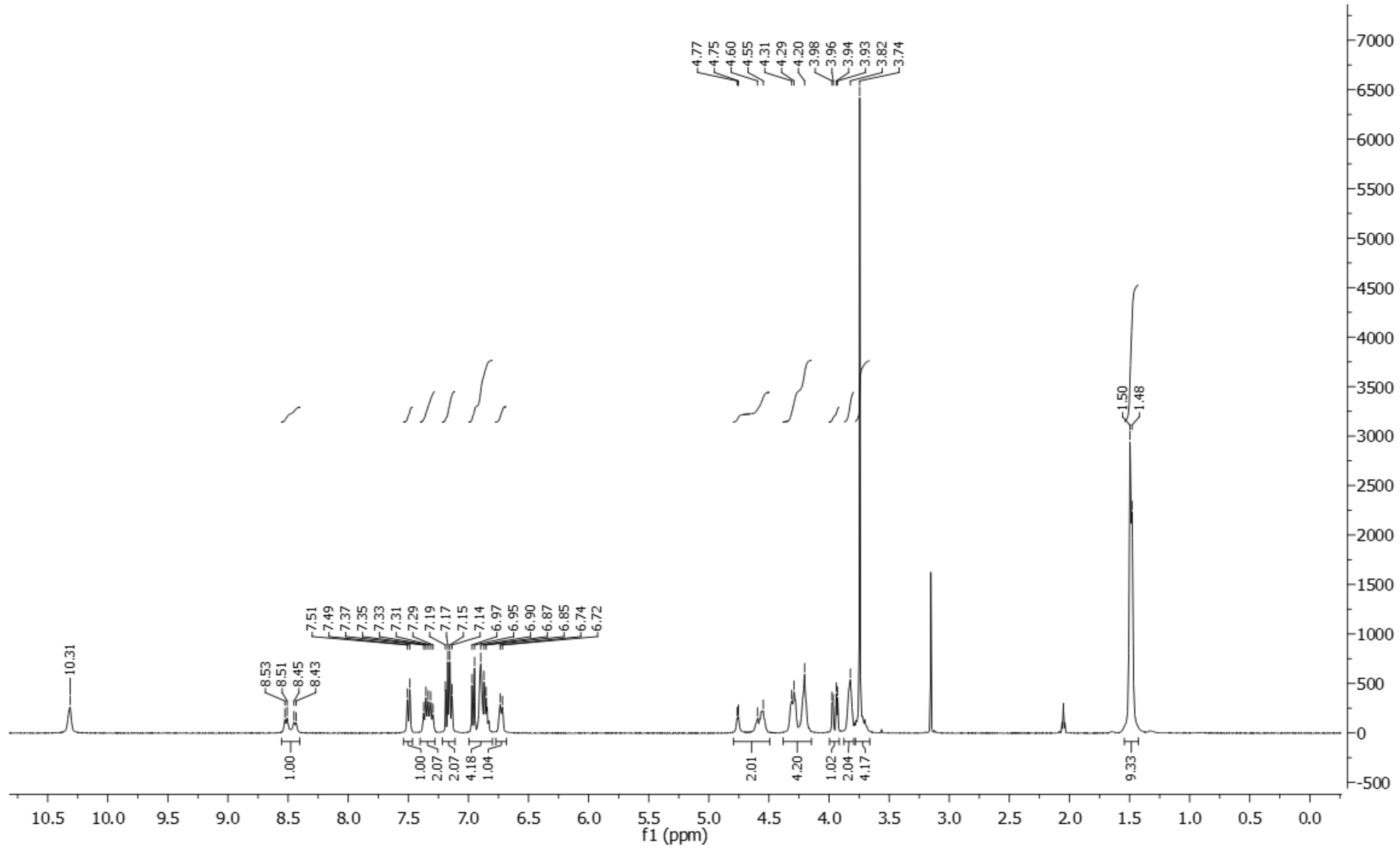

^{1}H-NMR spectrum of *N*-Boc-carvedilol

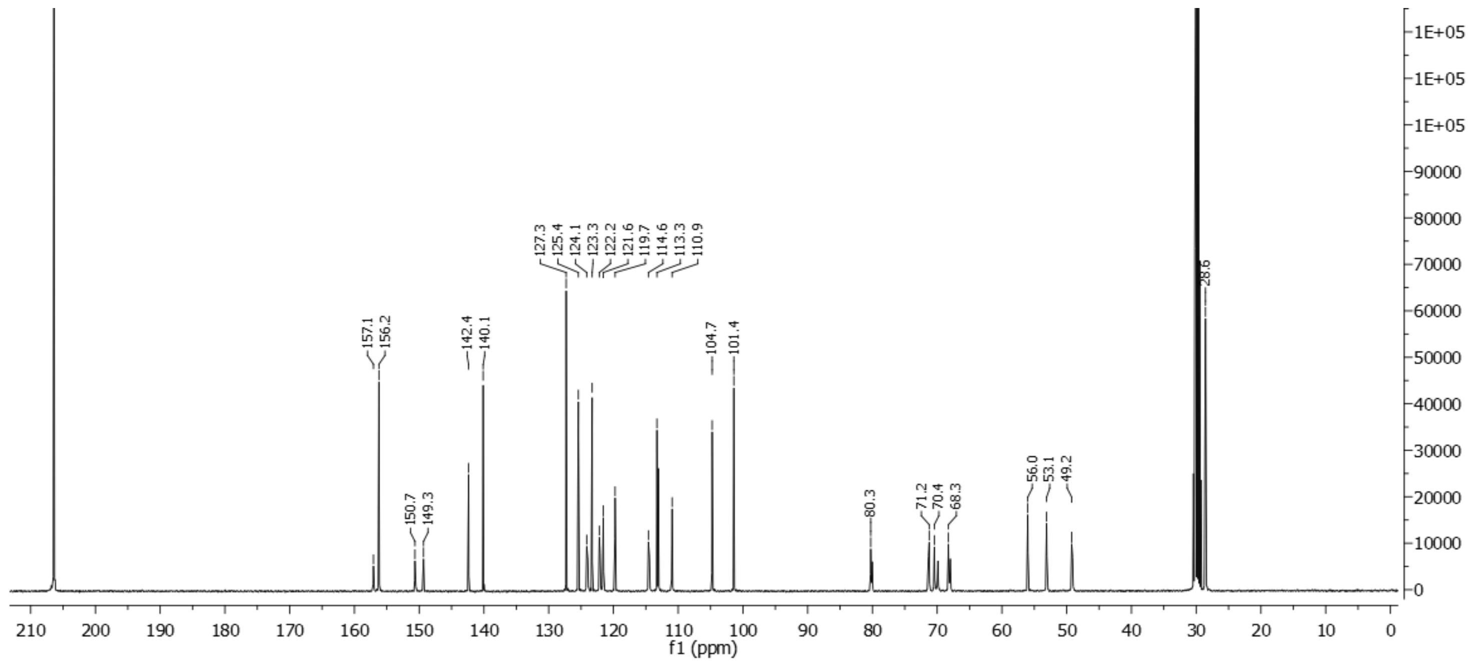

^{13}C-NMR spectrum of *N*-Boc-carvedilol

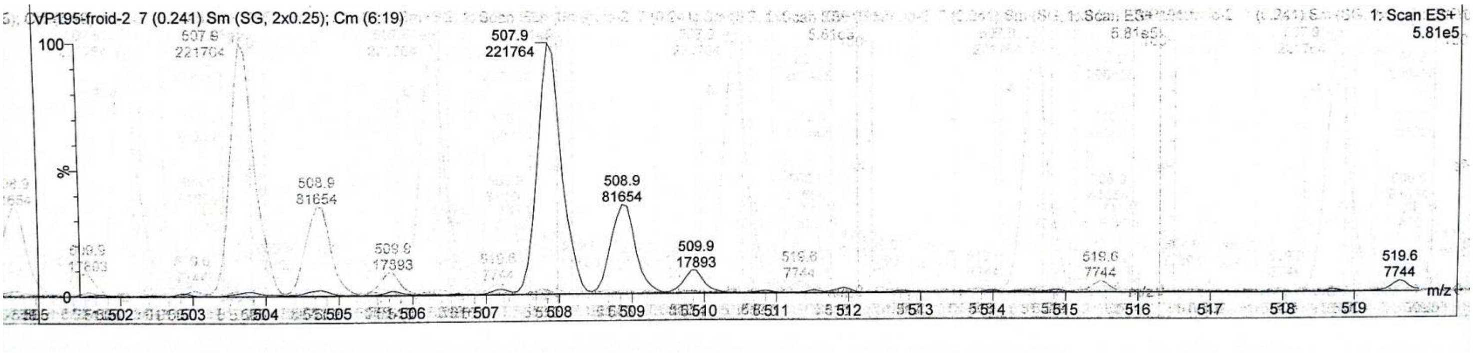

ESI-spectrum of *N*-Boc-carvedilol

H/D exchange reactions

2,5-Diphenyloxazole **1**

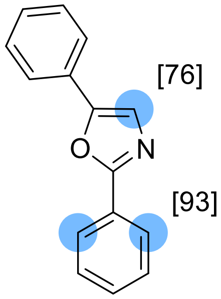

Chemical Formula: $C_{15}H_{11}NO$

Substrate	Solvent (Volume)	RuNp@PVP cat.
44.3mg, 0.2mmol	DMA (1mL)	14.4mg, 5mol%

Workup and purification:

After cooling down to room temperature ethylacetate : cyclohexane (1:1, 3mL) was added to the reaction mixture and stirred for 10min to let precipitate RuNp@PVP. The suspension was passed through a SiO_2 pad and the crude product was eluted with ethylacetate (5mL). The solvent was removed under vacuum and the crude product was purified by HPLC on an Interchim utisphere C18-HDO 5UM 150x21.2mm P REP-LC column. Condition: 1.7mL/min, UV & mass detection, 25°C, Solvents & gradients: Solvent A : H_2O + 0.1% HCOOH; Solvent B : ACN + 0.1% HCOOH

t (0)	95%A 5%B
t(24min)	50%A 50%B

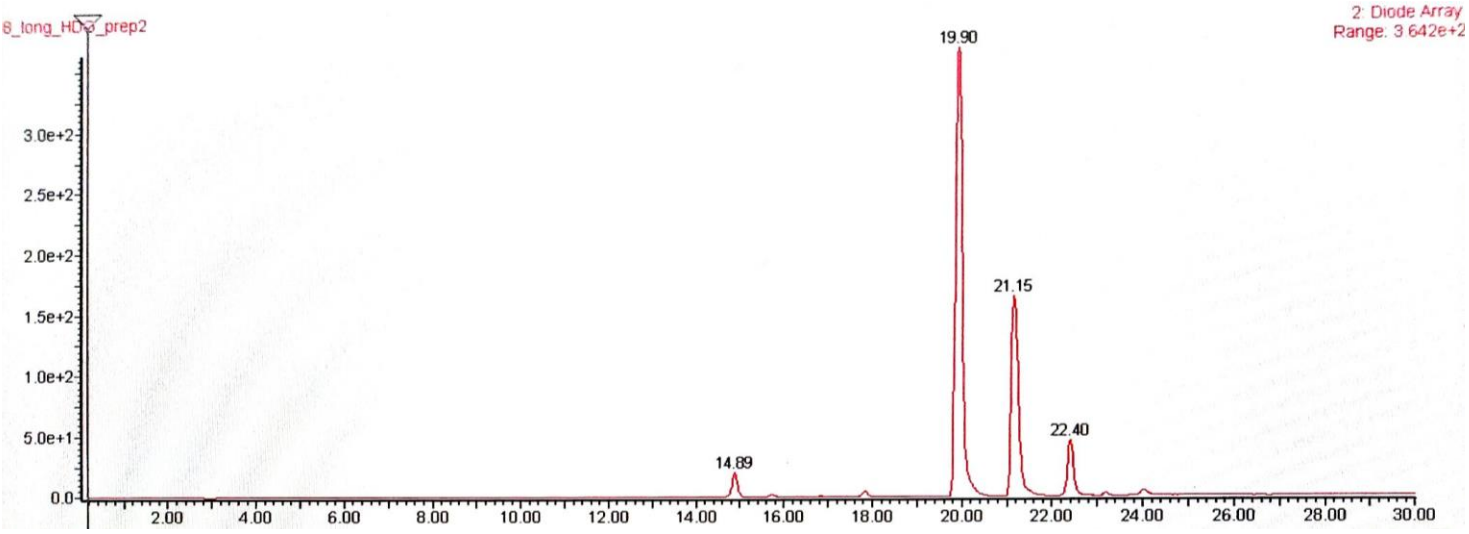

HPLC chromatogram

Yield: 11.0mg, 25%, white solid

^{1}H NMR (400 MHz, Acetone-d_6): δ 8.19 – 8.10 (m, 0.15H), 7.90 – 7.83 (m, 2H), 7.70 (s, 0.24H), 7.60 – 7.47 (m, 5H), 7.42 – 7.36 (m, 1H).

Deuterium incorporation was expected at *δ* 8.19 – 8.10 and at *δ* 7.70. Isotopic enrichment values were determined against the integral at *δ* 7.42 – 7.36.

^{2}H-{^{1}H}NMR (92 MHz, Acetone): δ 8.19 (s, 1.86D), 7.72 (s, 0.76D).

^{13}C-{^{1}H}NMR (100 MHz, Acetone-d_6): δ 161.6, 152.1 (m), 131.3, 129.9, 129.7, 129.3, 128.9, 128.3, 126.9 (m), 125.0, 124.7.

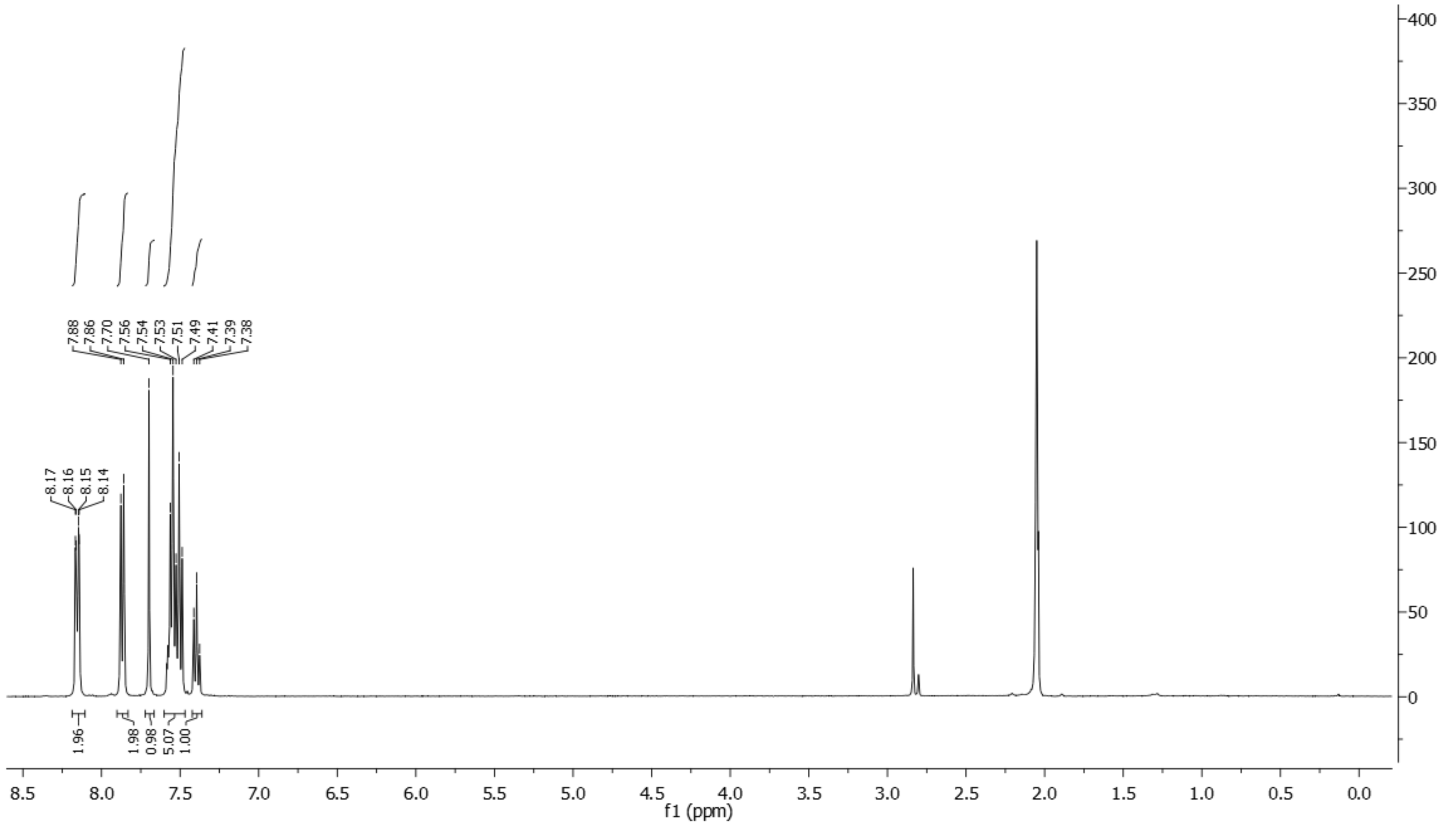

^{1}H-NMR spectrum of the non-deuterated starting material

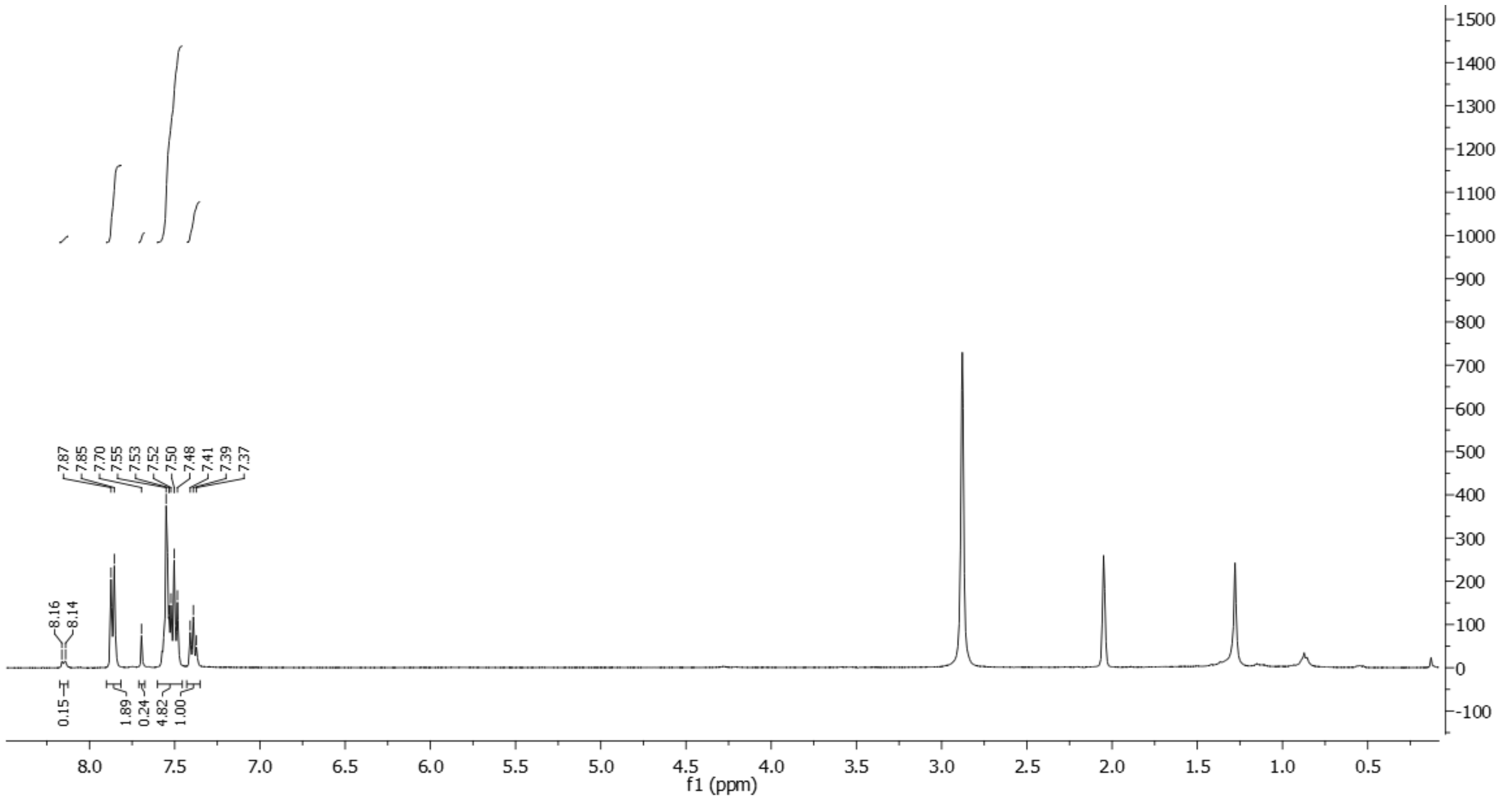

^{1}H-NMR spectrum of **1**

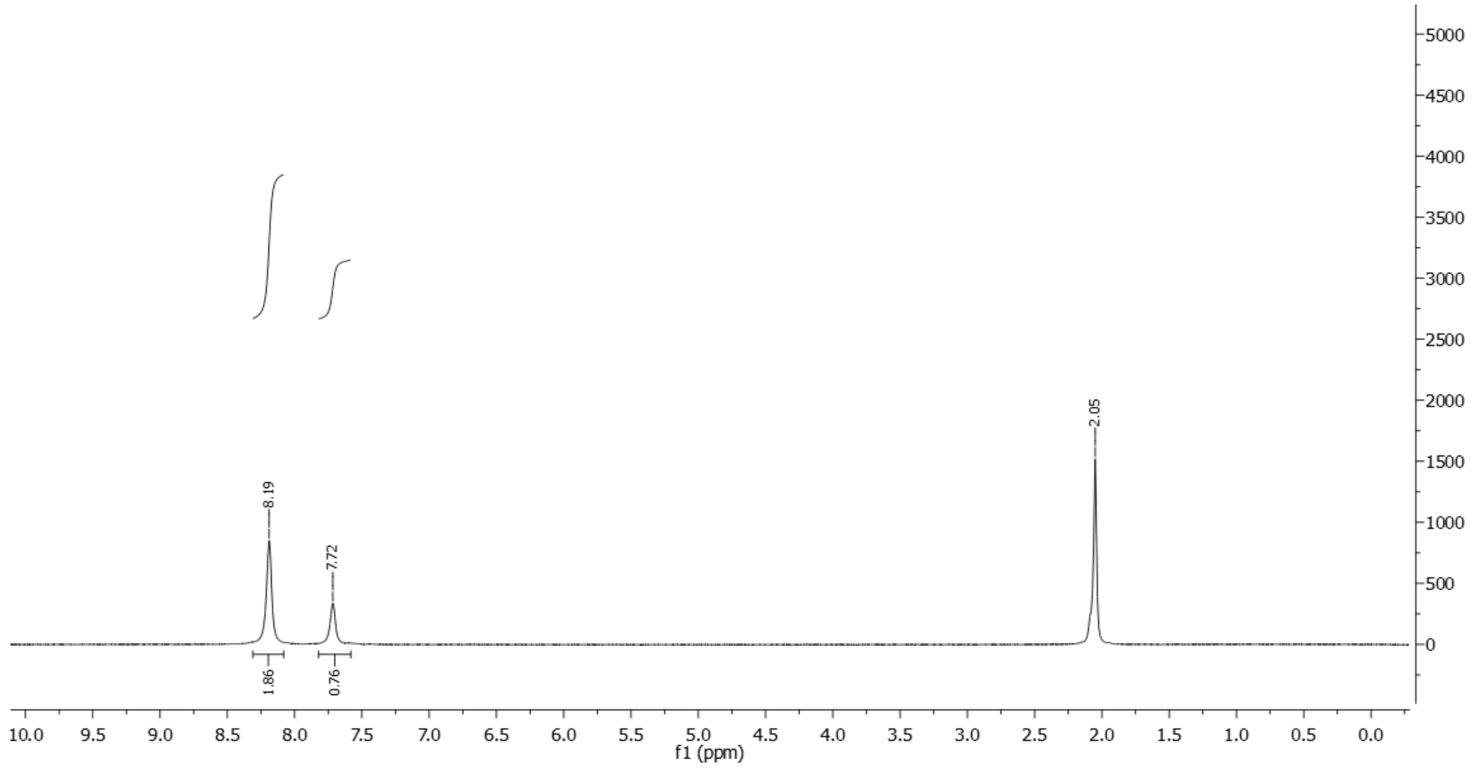

^{2}H-NMR spectrum of **1**

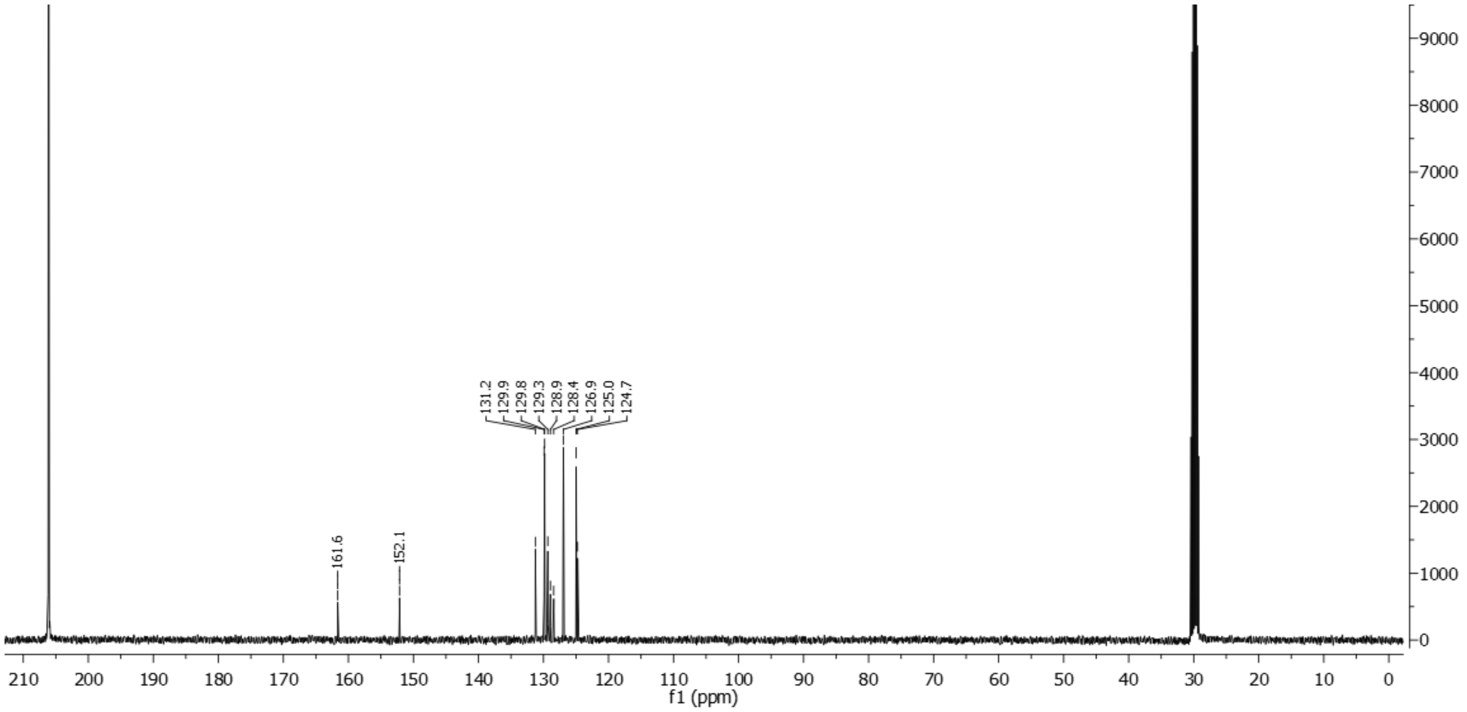

^{13}C-NMR spectrum of the non-deuterated starting material

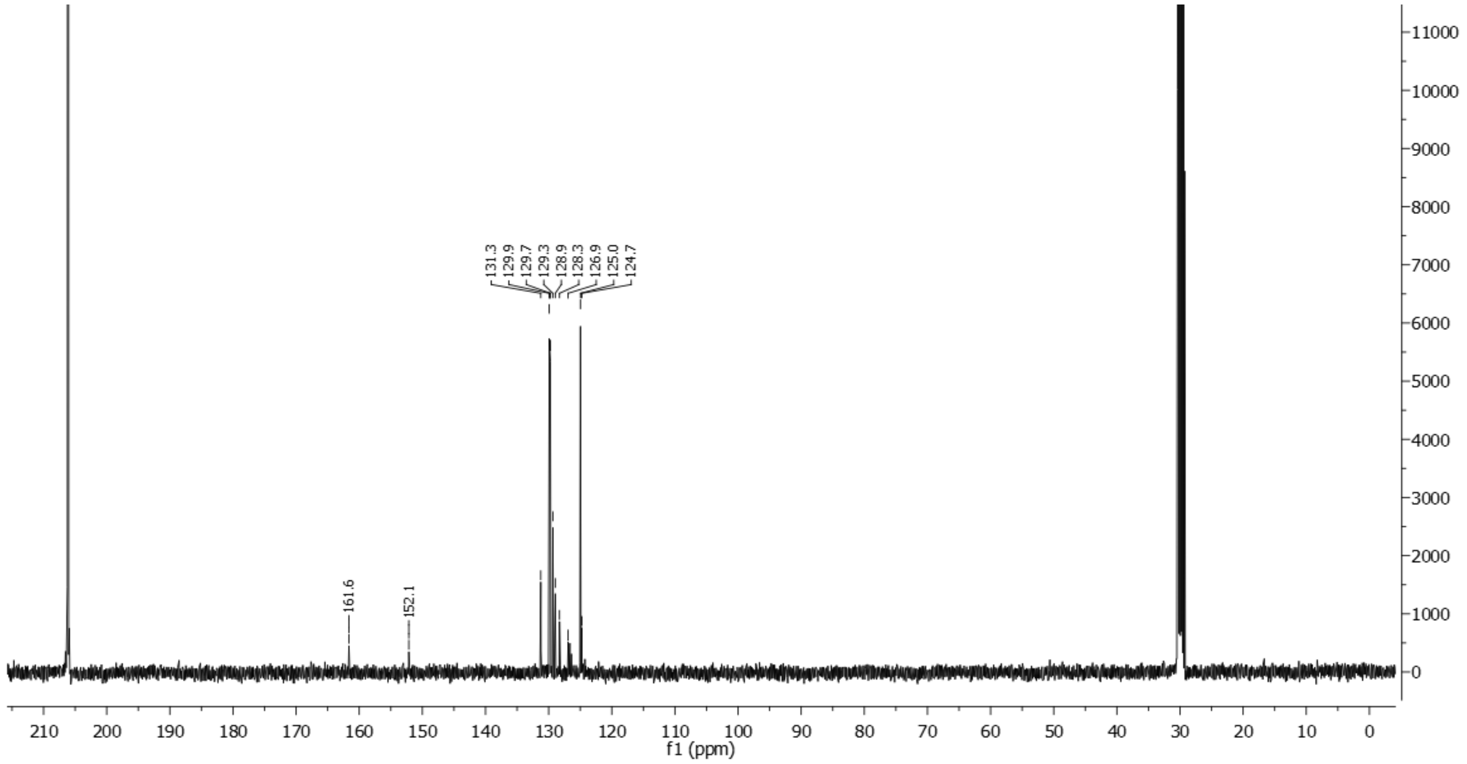

^{13}C-NMR spectrum of **1**

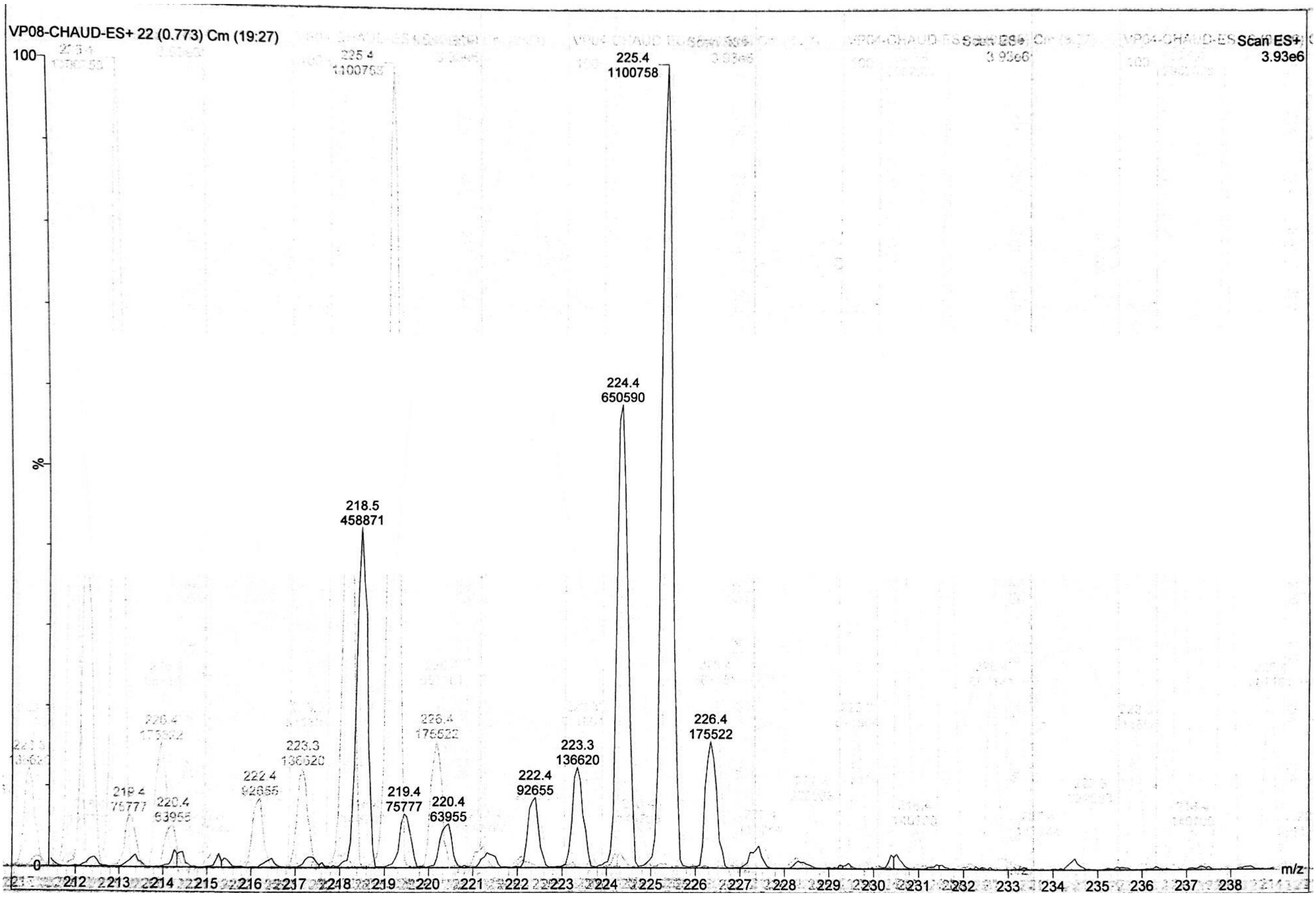

ESI spectrum of **1**

5-(4-Methylphenyl)-oxazole **2**

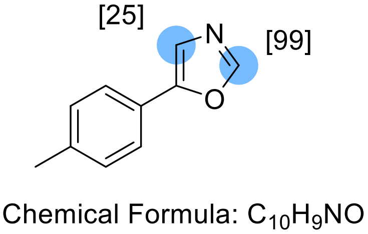

Substrate	Solvent (Volume)	RuNp@PVP cat.
31.8mg, 0.2mmol	THF (2mL)	14.4mg, 5mol%

Workup and purification:
After cooling down to room temperature, cyclohexane (2mL) was added to the reaction mixture and stirred for 10min to let precipitate RuNp@PVP. The suspension was passed through a SiO_2 pad and the product was eluted with ethylacetate (5mL). The solvent was removed under vacuum.

Yield: 25.0mg, 79%, light yellow solid

^{1}H NMR (400 MHz, Acetone-*d*$_6$): δ 8.16 (s, 0.01H), 7.73 – 7.57 (m, 2H), 7.49 (s, 0.75H), 7.36 – 7.11 (m, 2H), 2.36 (s, 3H).

Deuterium incorporation was expected at *δ* 8.16 and at *δ* 7.49. Isotopic enrichment values were determined against the integral at *δ* 7.36 – 7.11.

^{2}H-{^{1}H}NMR (92 MHz, Acetone): δ 8.12 (s, 0.99D), 7.47 (s, 0.20D),

^{13}C-{^{1}H}NMR (100 MHz, Acetone-*d$_6$*): δ 152.2, 151.4 (m), 139.3, 130.4, 126.2, 125.0, 121.9, 21.2.

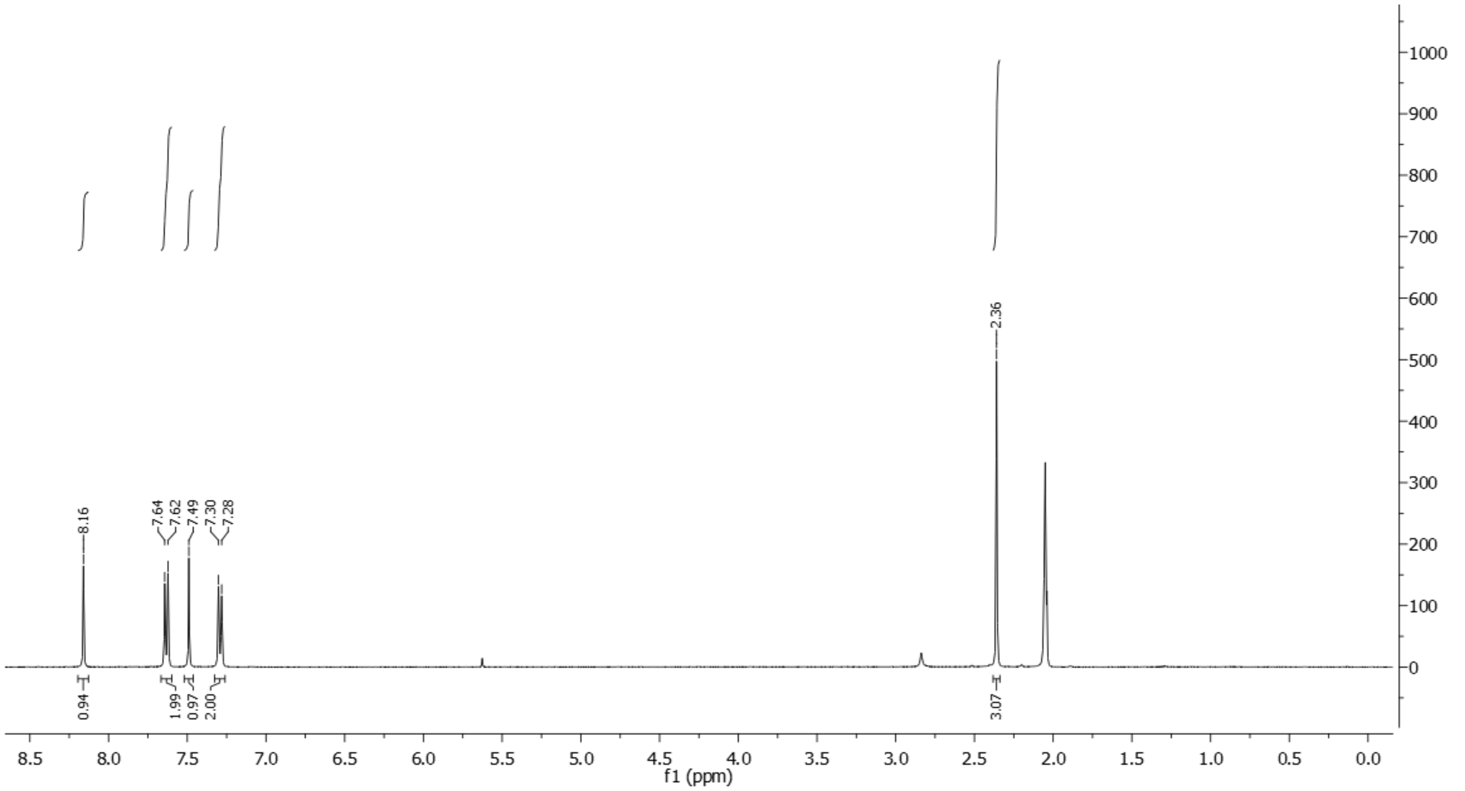

^{1}H-NMR spectrum of the non-deuterated starting material

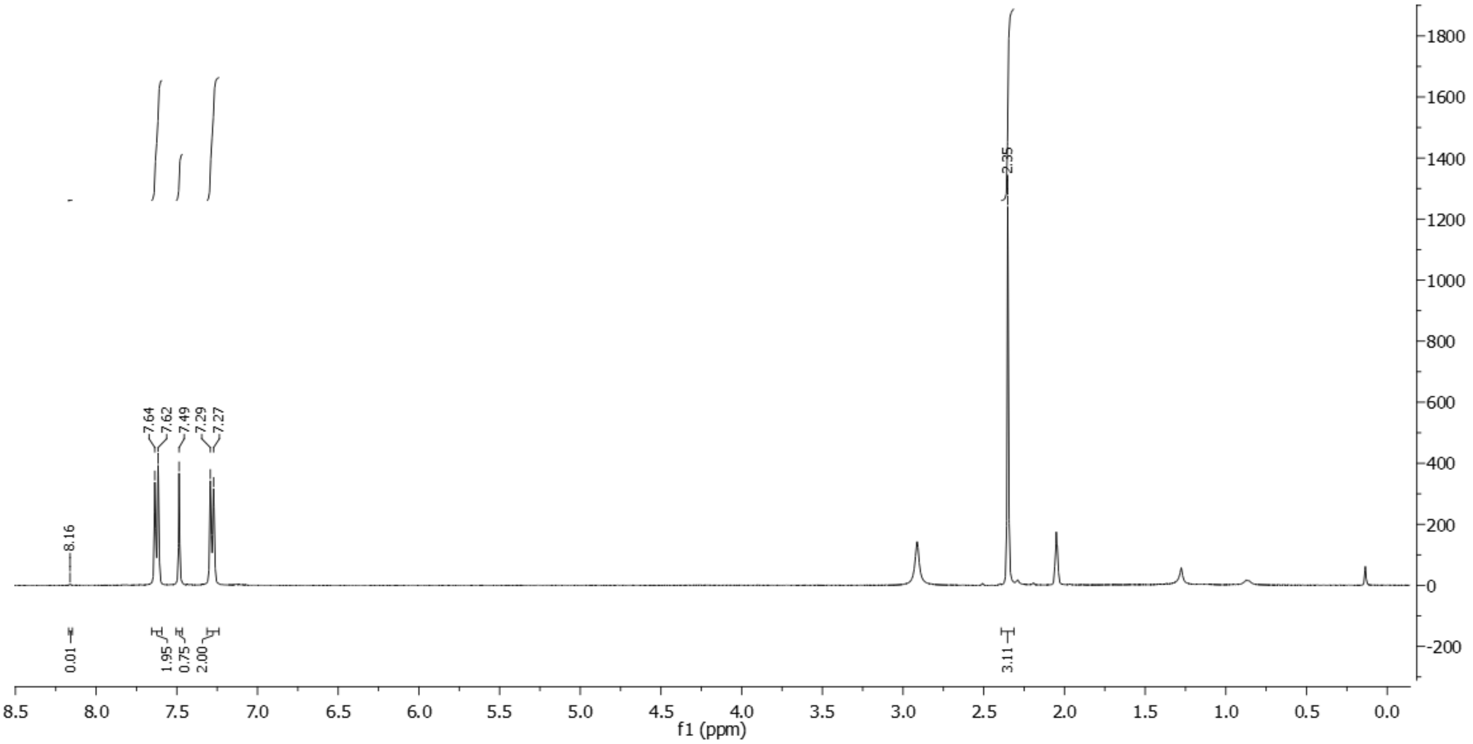

^{1}H-NMR spectrum of **2**

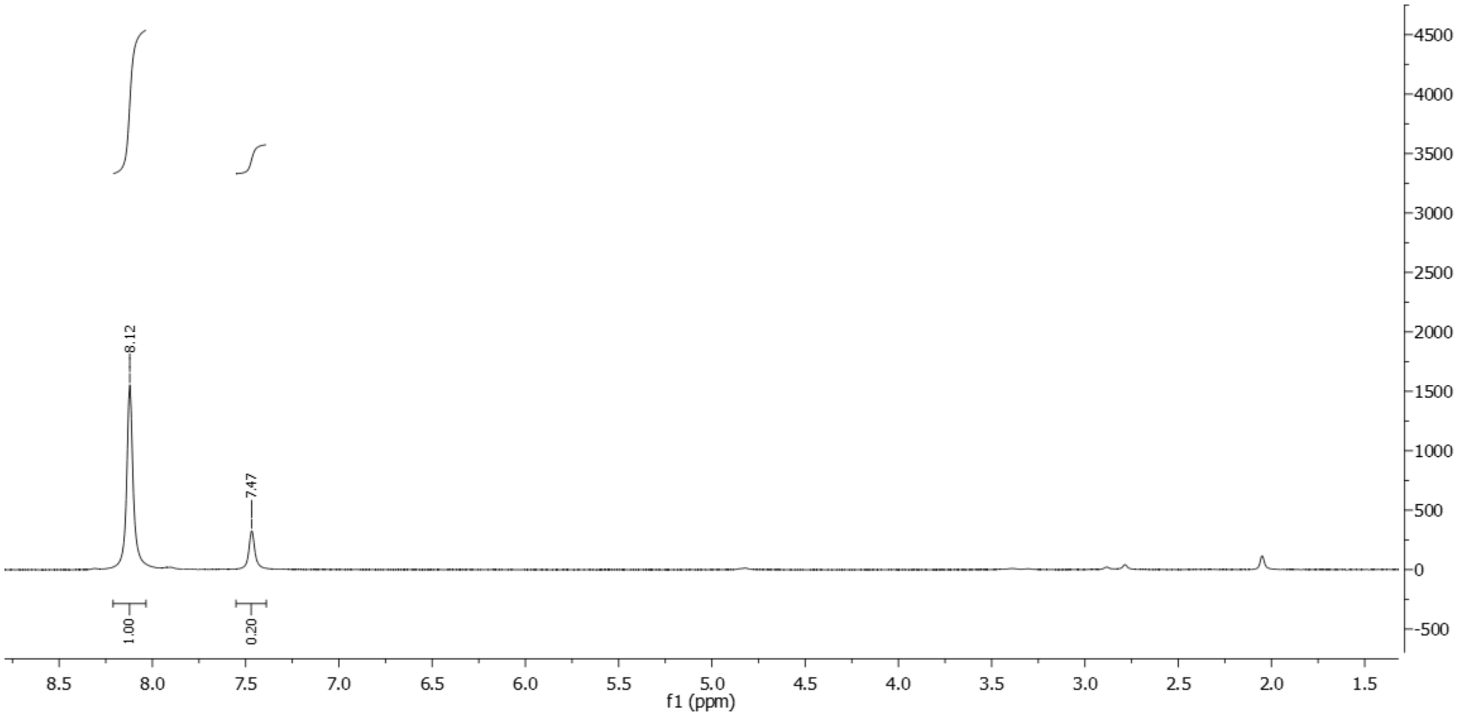

^{2}H-NMR spectrum of **2**

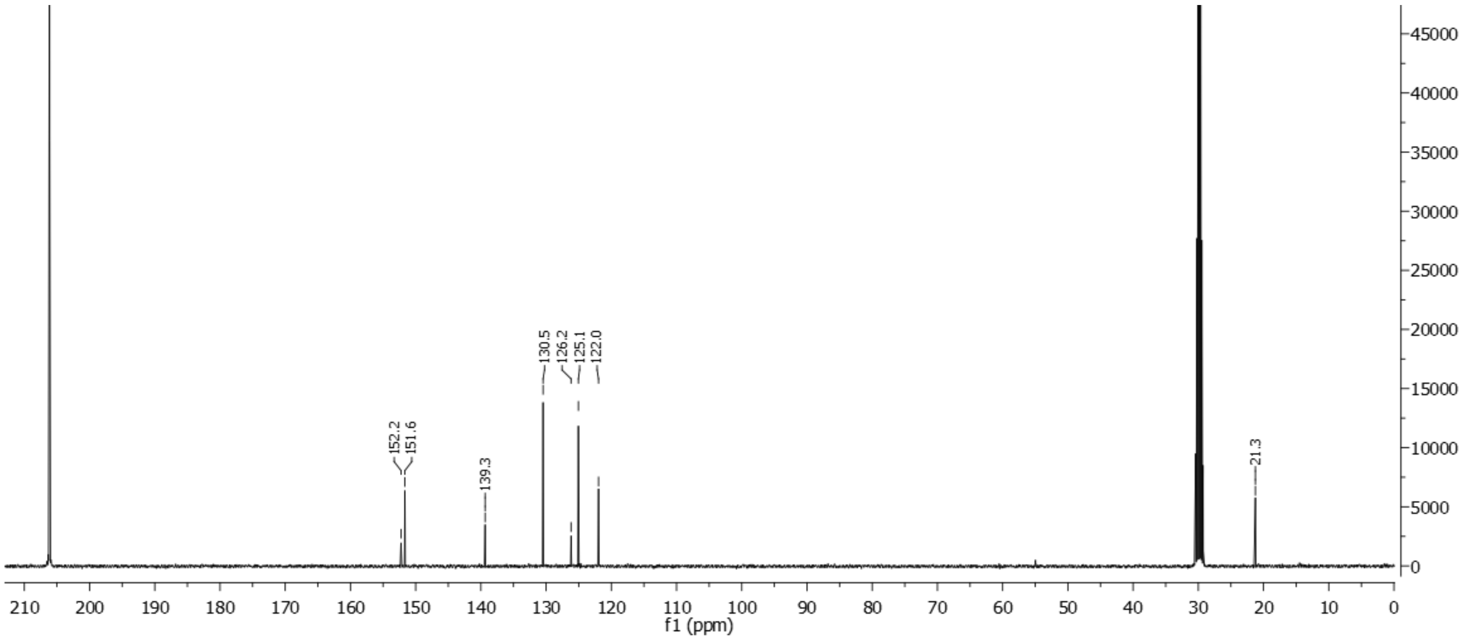

^{13}C-NMR spectrum of the non-deuterated starting material

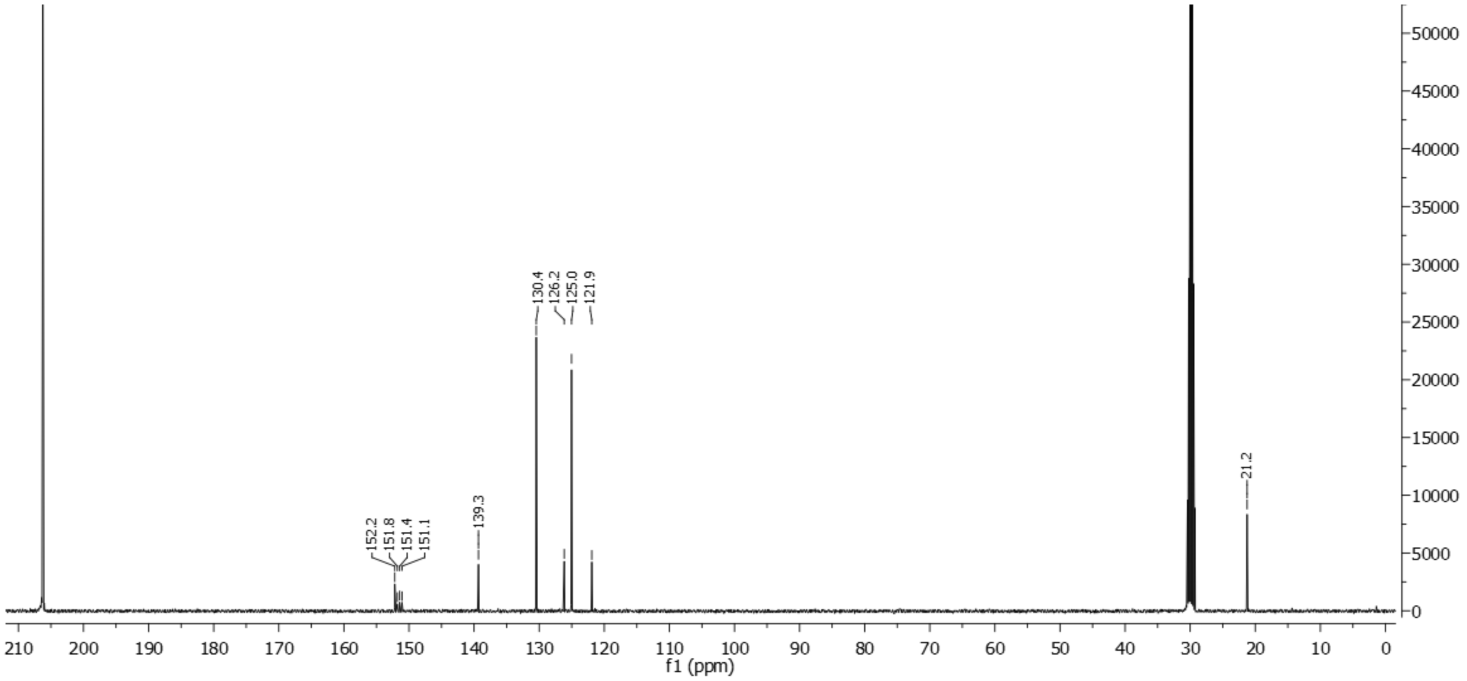

^{13}C-NMR spectrum of **2**

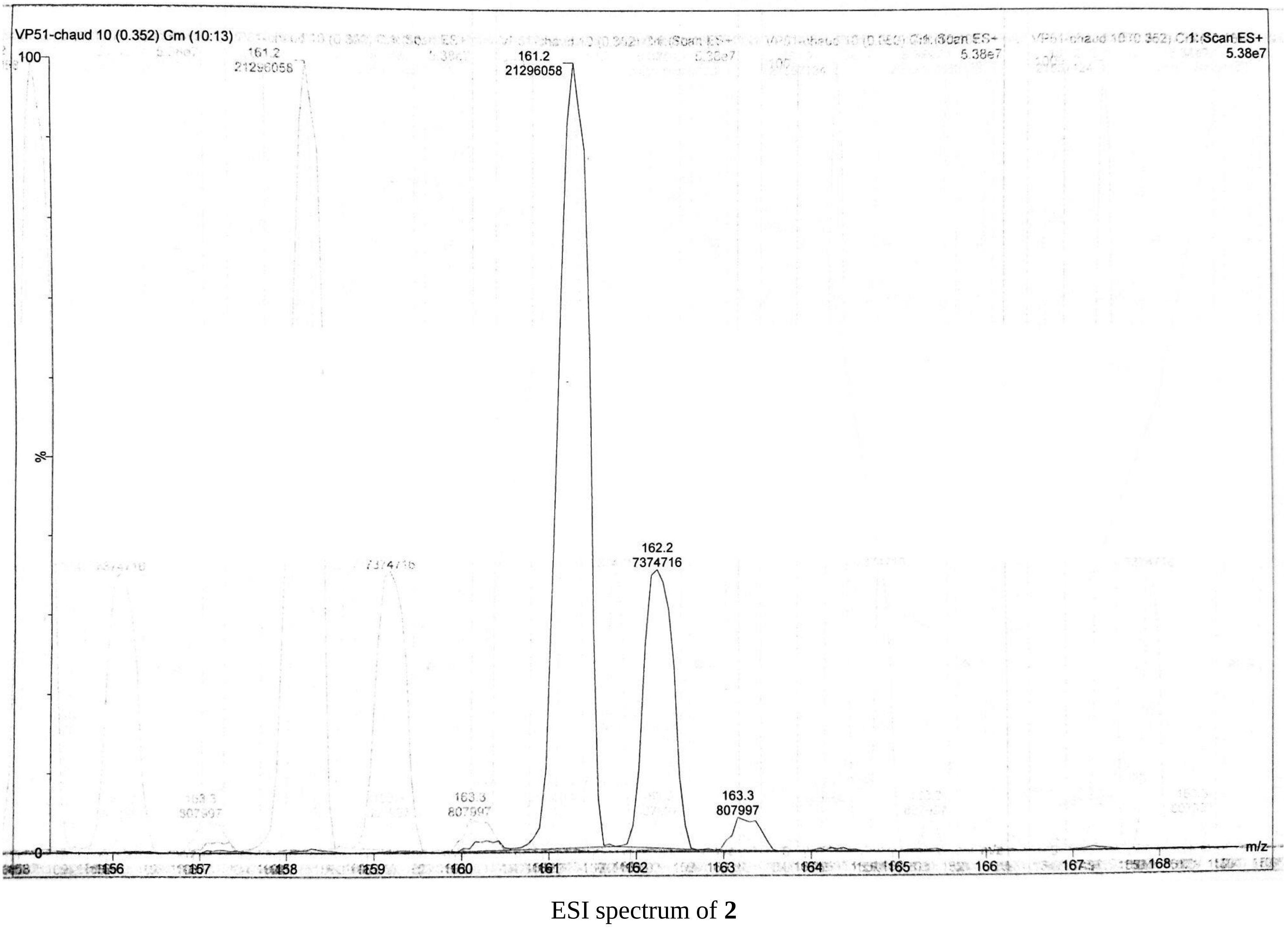

ESI spectrum of **2**

4-(Oxazol-5-yl)aniline **3**

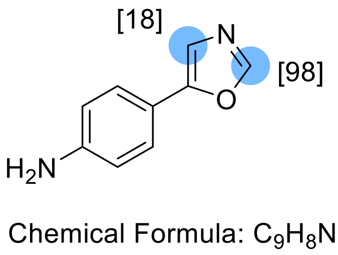

Substrate	Solvent (Volume)	RuNp@PVP cat.
32.0mg, 0.2mmol	DMA (2mL)	14.4mg, 5mol%

Workup and purification:

After cooling down to room temperature, the reaction mixture was poured on a brine solution (50mL) in a separation funnel. The aqueous phase was extracted 3 times with a mixture of ethylacetate and cyclohexane (EtOAc : Cy 3:1; 50mL). The organic phases were combined and dried over $MgSO_4$. The solvent was removed under vacuum.

Yield: 16.0mg, 50%, orange solid

^{1}H NMR (400 MHz, $CDCl_3$): δ 7.83 (s, 0.01H), 7.49 – 7.42 (m, 2H), 7.15 (s, 0.82H), 6.76 – 6.68 (m, 2H), 3.84 (bs, 1H).

Deuterium incorporation was expected at *δ* 7.83 and at *δ* 7.15. Isotopic enrichment values were determined against the integral at *δ* 7.49 – 7.42.

^{2}H-{^{1}H}NMR (92 MHz, $CHCl_3$): δ 7.86 (s, 0.99D), 7.19 (s, 0.18D).

^{13}C-{^{1}H}NMR (100 MHz, $CDCl_3$): δ 147.0, 126.0, 119.2, 118.5, 115.2.

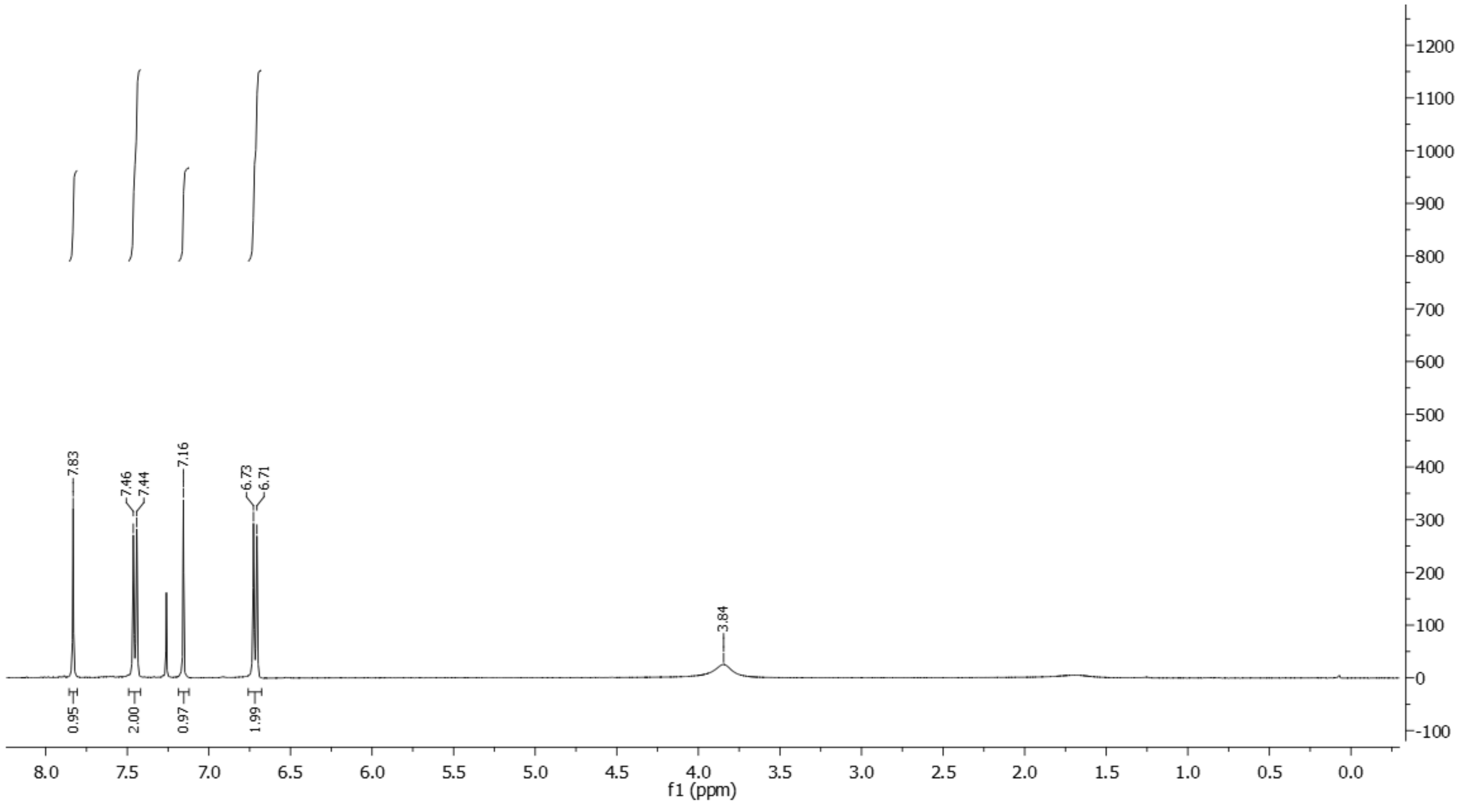

^{1}H-NMR spectrum of the non-deuterated starting material

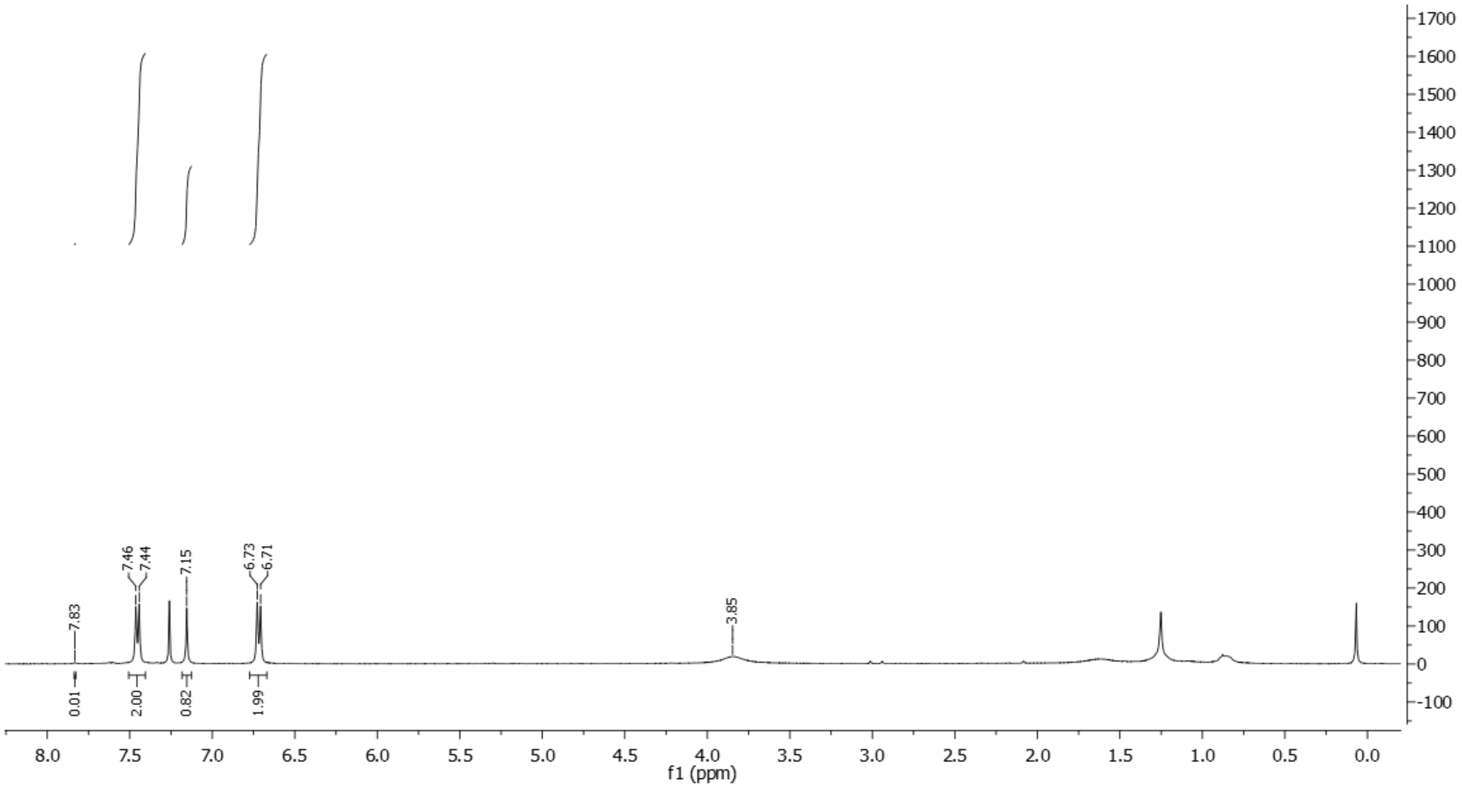

^{1}H-NMR spectrum of **3**

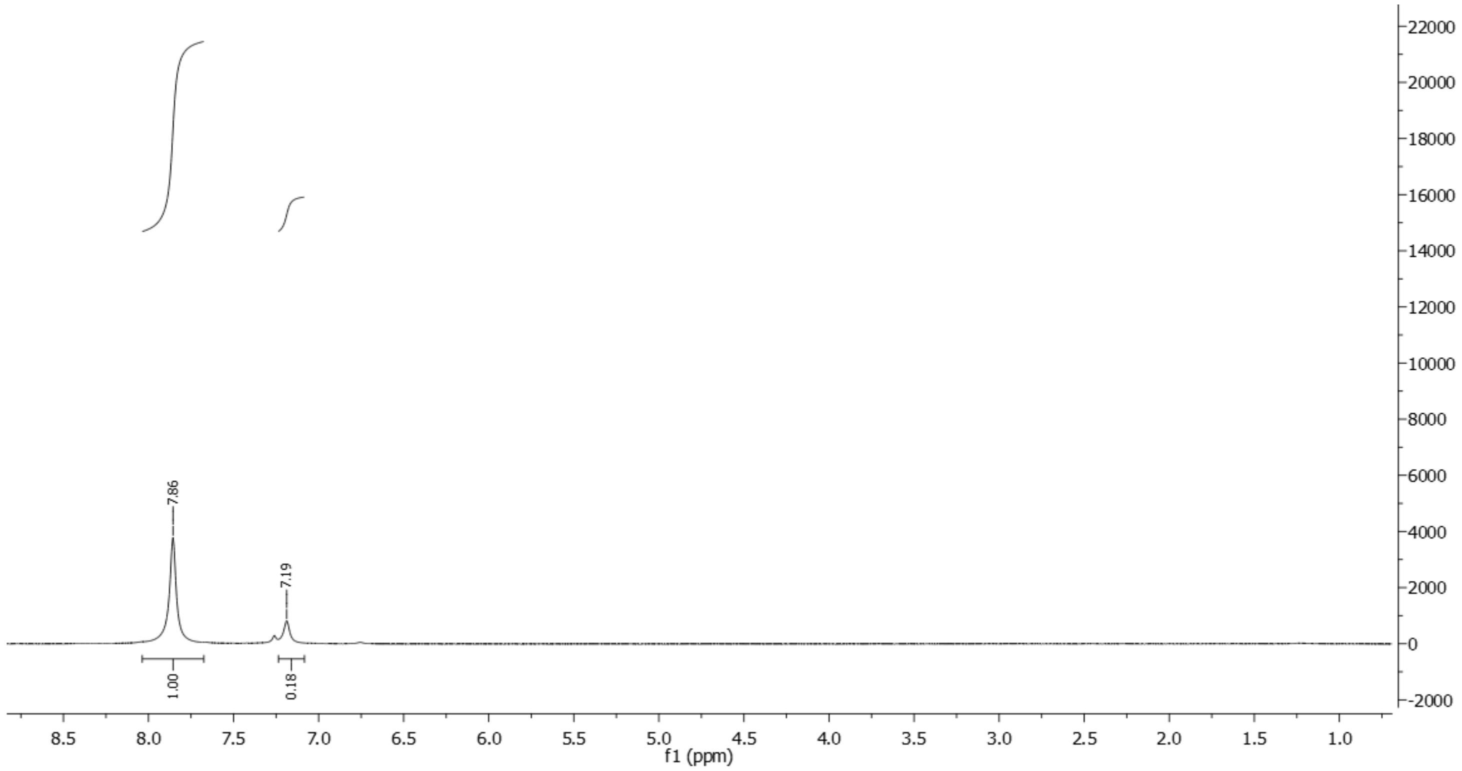

^{2}H-NMR spectrum of **3**

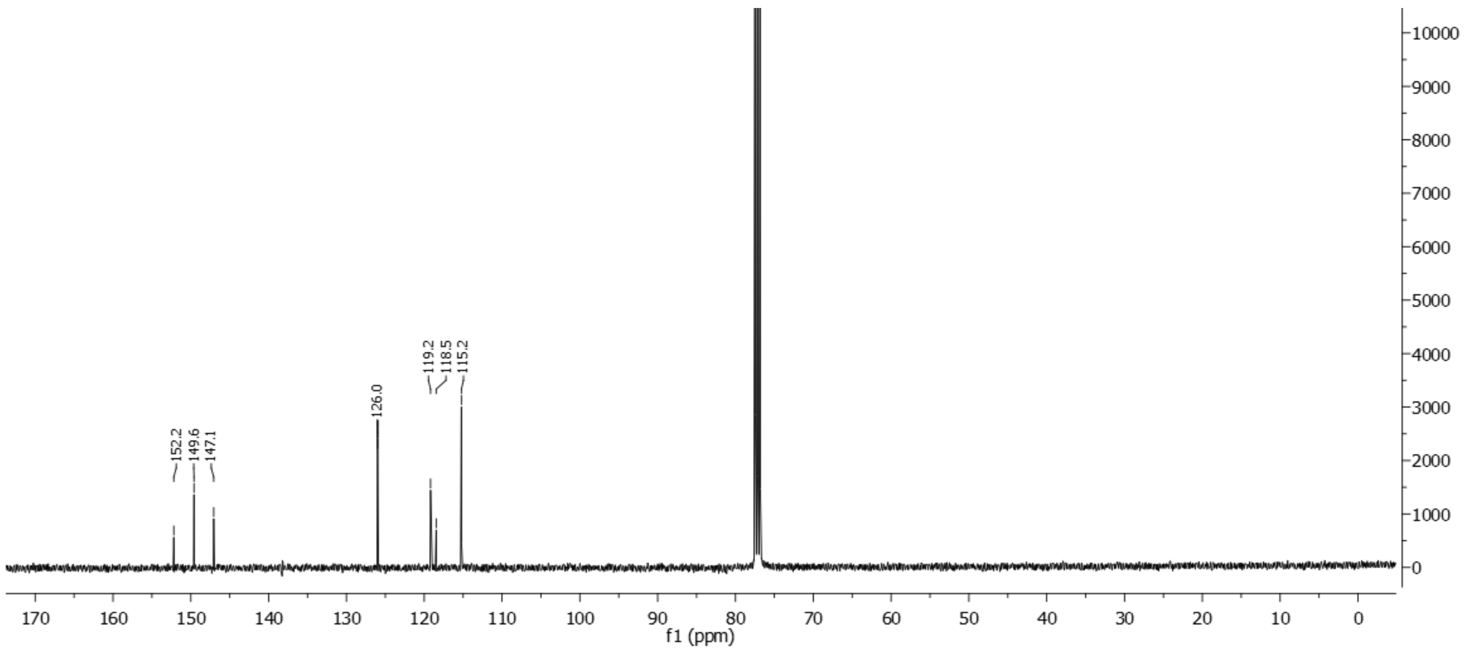

^{13}C-NMR spectrum of the non-deuterated starting material

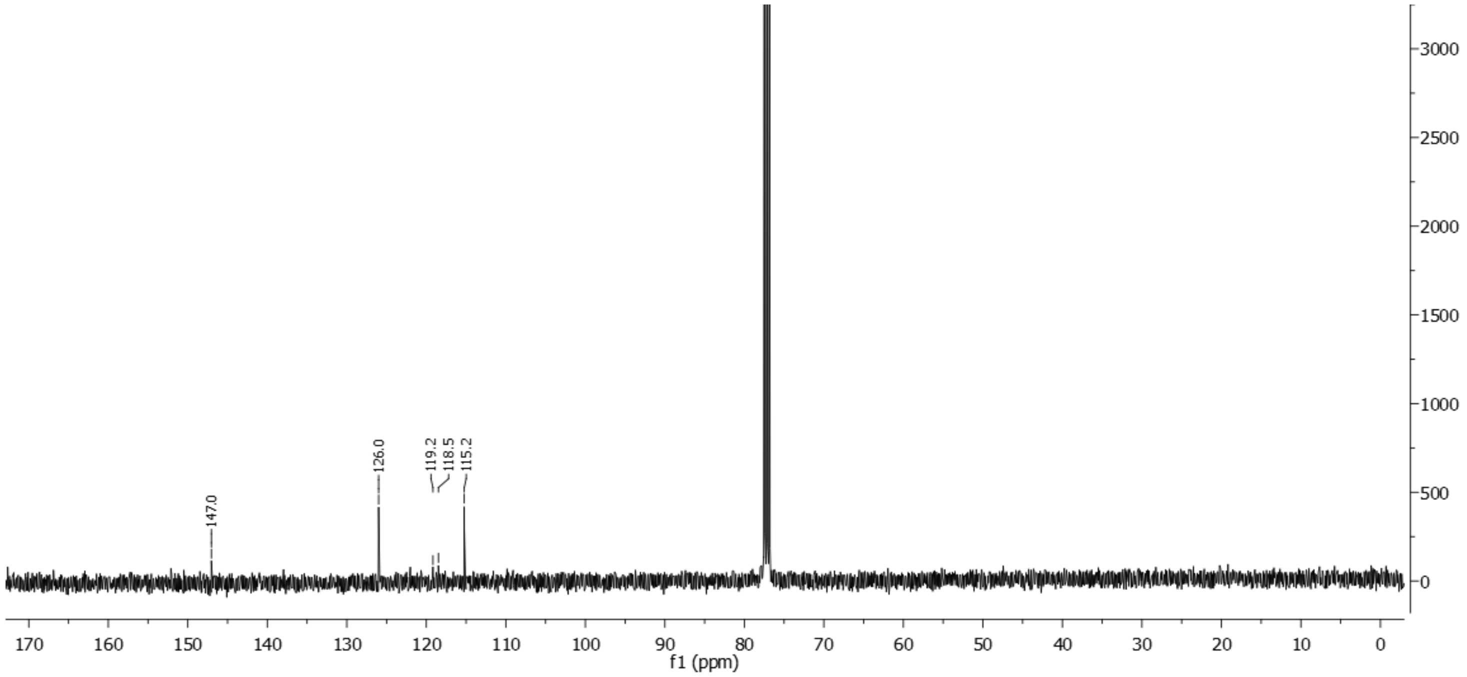

^{13}C-NMR spectrum of **3**

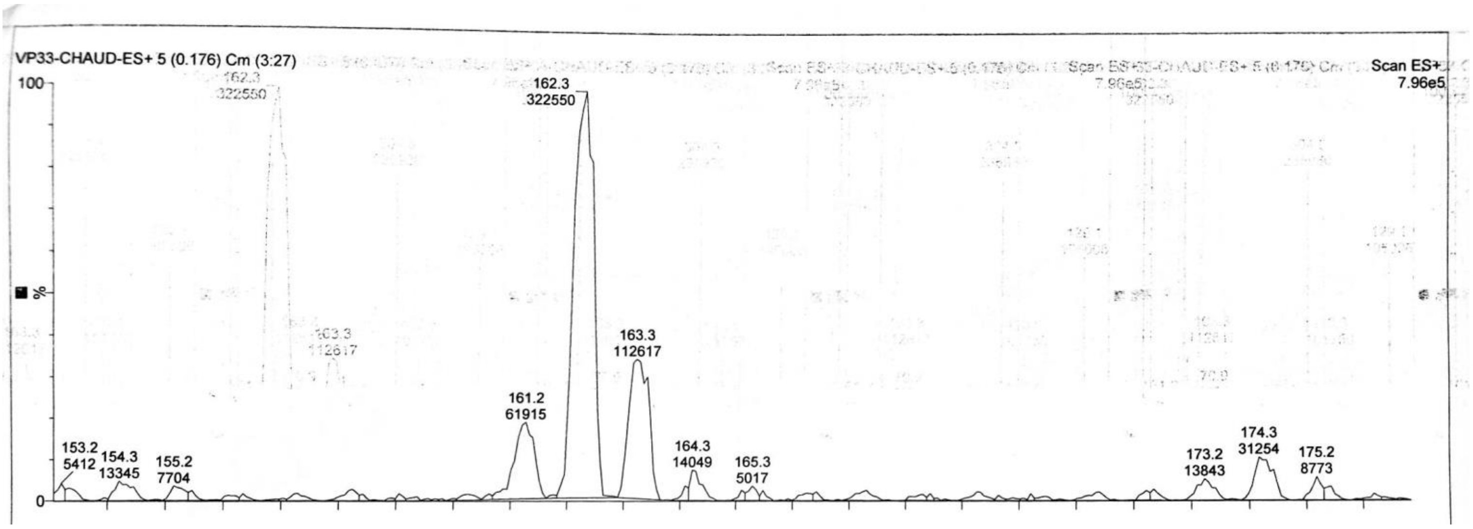

ESI spectrum of **3**

5-(4-Methoxyphenyl)-oxazole-4-carboxylic acid **4**

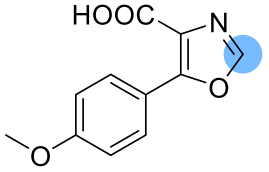

[61]

Chemical Formula: $C_{11}H_9NO_4$

Substrate	Solvent (Volume)	RuNp@PVP cat.
43.8mg, 0.2mmol	DMA (2mL)	14.4mg, 5mol%

Workup and purification:

After cooling down to room temperature, the reaction mixture was poured on a brine solution (50mL) in a separation funnel. The aqueous phase was extracted 3 times with a mixture of ethylacetate and cyclohexane (EtOAc : Cy 3:1; 50mL). The organic phases were combined and dried over $MgSO_4$. The solvent was removed under vacuum.

Yield: 45.0mg, 99%, light yellow solid

^{1}H NMR (400 MHz, Acetone-*d$_6$*): δ 8.24 (s, 0.39H), 8.16 – 8.11 (m, 2H), 7.09 – 7.05 (m, 2H), 3.88 (s, 3H).

Deuterium incorporation was expected at δ 8.24. Isotopic enrichment values were determined against the integral at δ 7.09 – 7.05.

^{2}H-{^{1}H}NMR (92 MHz, Acetone): δ 8.19 (s)

^{13}C-{^{1}H}NMR (100 MHz, Acetone-*d$_6$*): δ 163.3, 162.2, 155.9, 150.0 (m), 130.9, 126.1, 120.3, 114.7, 55.8.

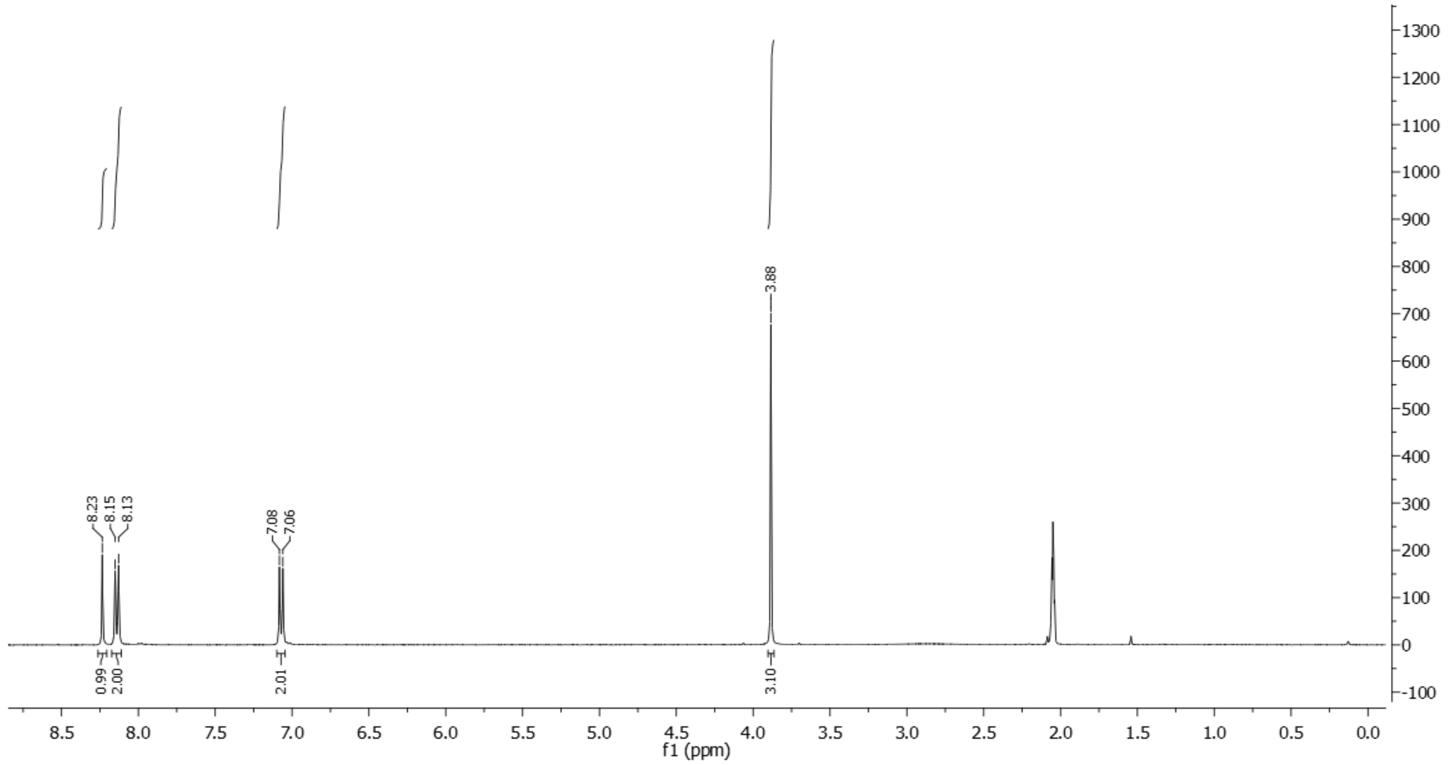

^{1}H-NMR spectrum of the non-deuterated starting material

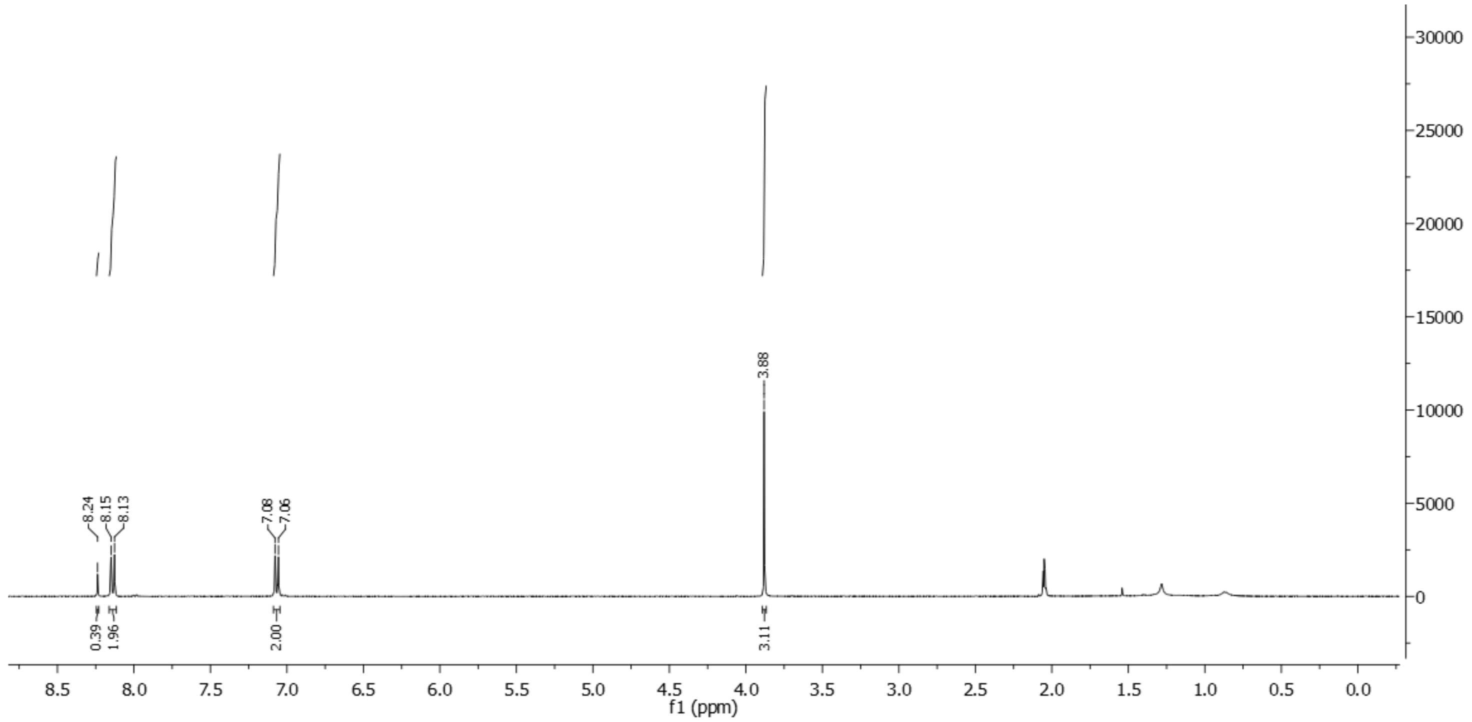

^{1}H-NMR spectrum of **4**

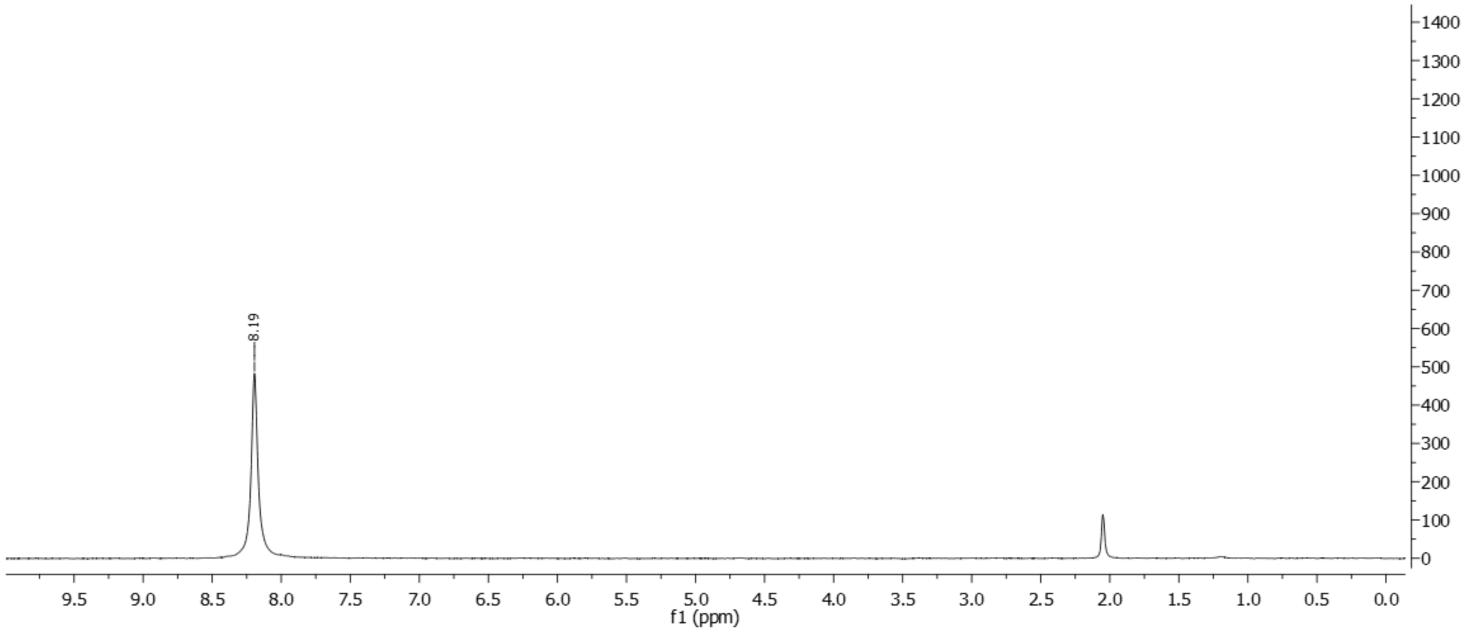

^{2}H-NMR spectrum of **4**

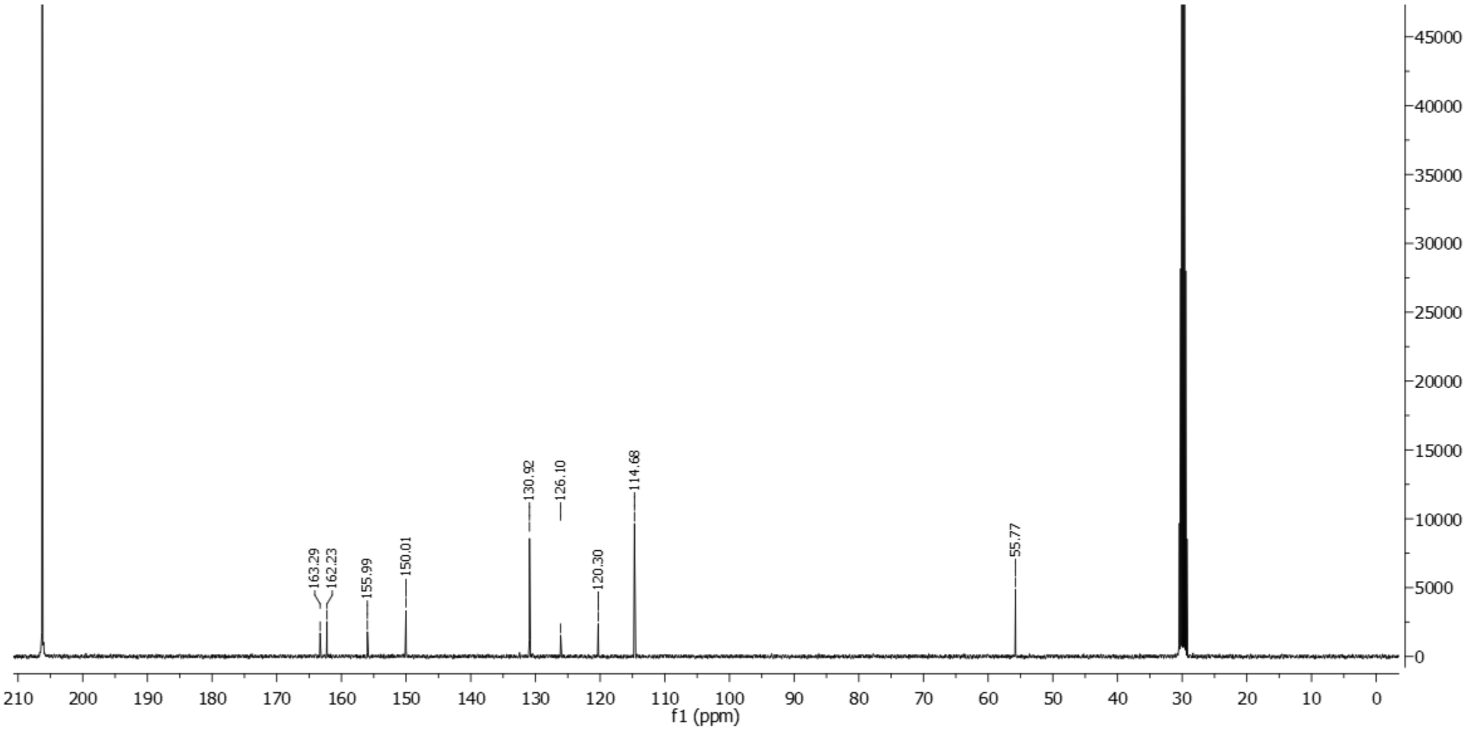

^{13}C-NMR spectrum of the non-deuterated starting material

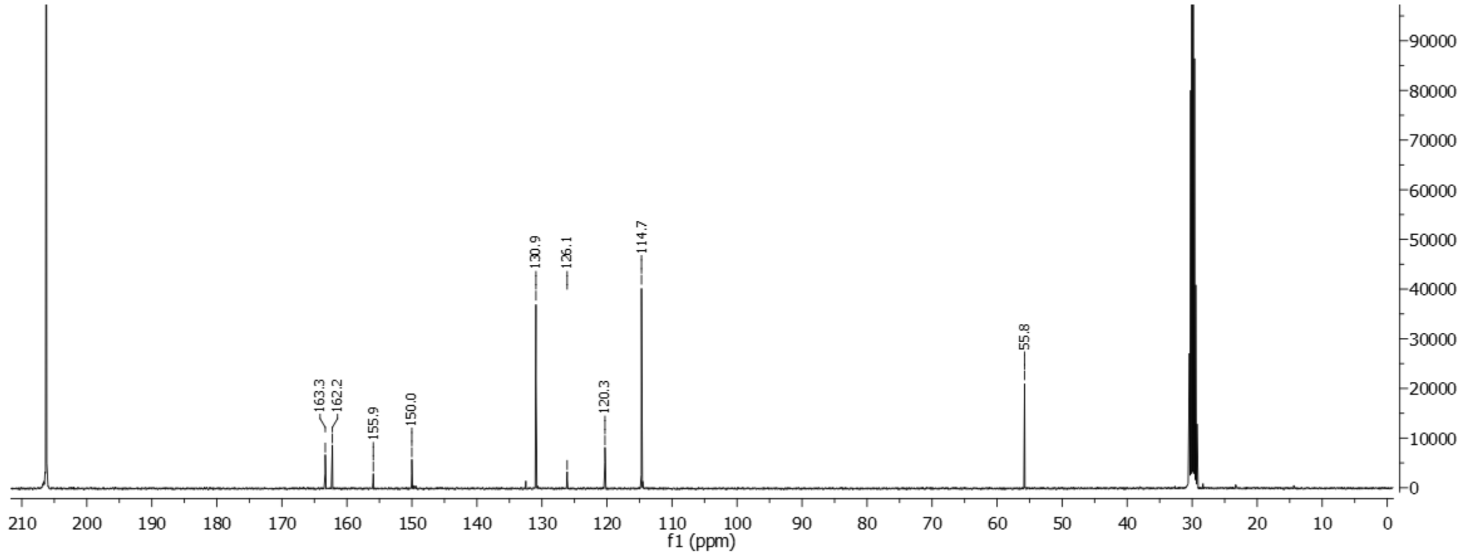

^{13}C-NMR spectrum of **4**

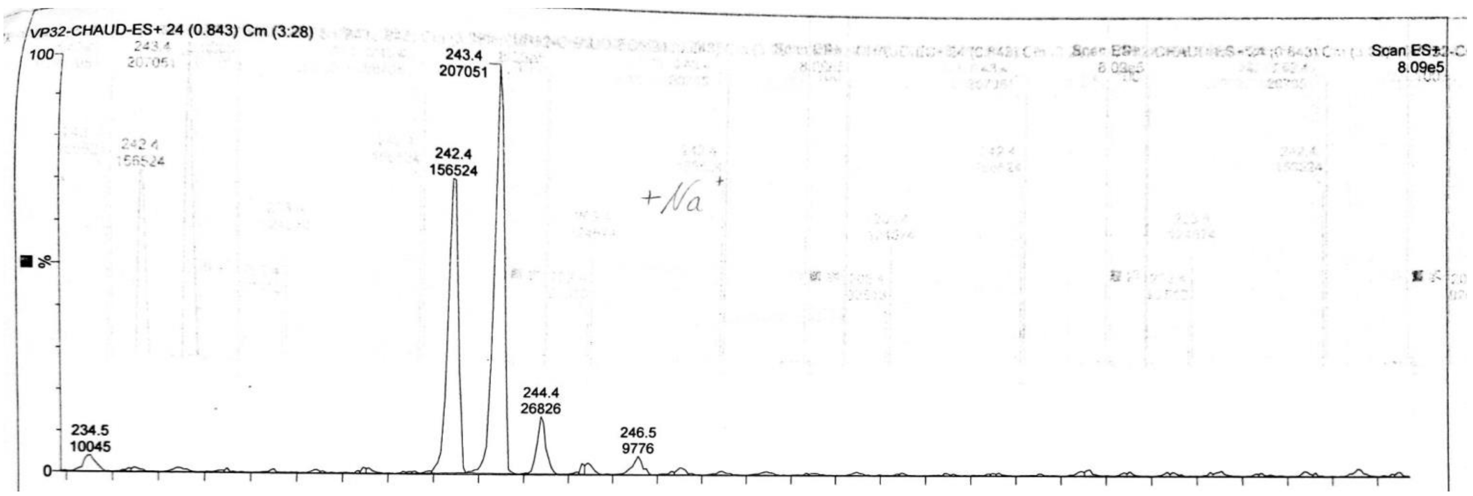

ESI spectrum of **4**

2-Phenylimidazole **5**

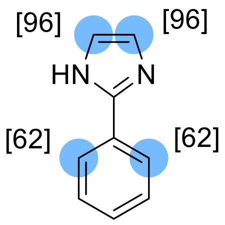

Chemical Formula: $C_9H_8N_2$

Substrate	Solvent (Volume)	RuNp@PVP cat.
28.8mg, 0.2mmol	THF (2mL)	14.4mg, 5mol%

Workup and purification:

After cooling down to room temperature, cyclohexane (2mL) was added to the reaction mixture and stirred for 10min to let precipitating RuNp@PVP. The suspension was passed through a pad of neutral Al_2O_3 and then eluted with ethylacetate (5mL). The solvent was removed under vacuum to obtain 31.0mg of crude product. The crude product (10mg) was purified by HPLC on an XBridge Prep Phenyl 5µm OBD 19x150mm column. Condition: 1mL/min, UV & mass detection, 25°C, Solvents & gradients: Solvent A : H_2O + 0.1% HCOOH; Solvent B : MeOH + 0.1% HCOOH

t (0)	95%A	5%B
t(24min)	50%A	50%B

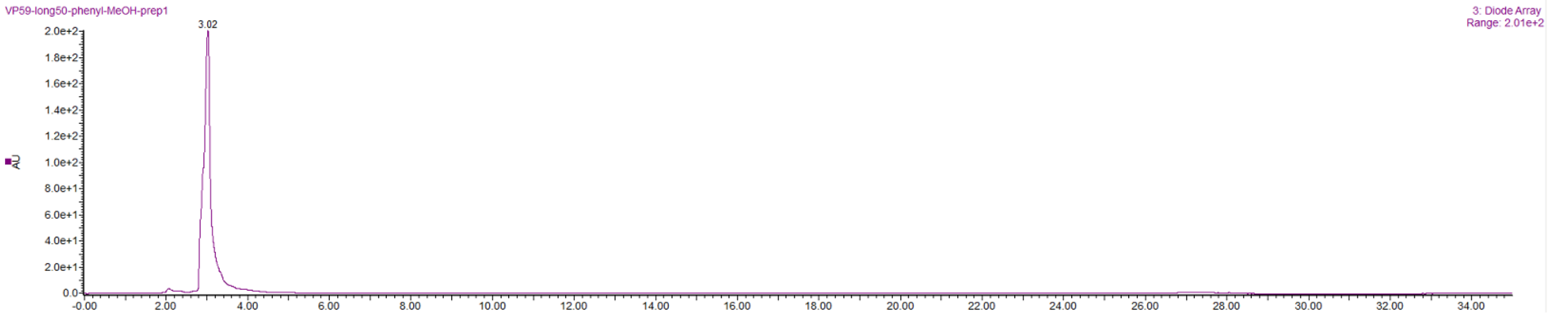

HPLC chromatogram

The obtained formiate salt was dissolved in methanol (1mL) and an aqueous K_2CO_3-solution (0.03M, 1mL) was added under stirring. The mixture was poured in H_20 dist. (50mL) in a separation funnel and extracted 3 times with ethylacetate (50mL). The organic phases were combined and the solvent was removed under vacuum. The neutralized product was purified on neutral Al_2O_3, where it could be eluted with EtOAc/Cy (3:1).

Yield: 3.0mg, 30%, white solid

^{1}H NMR (400 MHz, Acetone-*d*$_6$): δ 11.64 (bs, NH), 8.01 – 7.96 (m, 0.79H), 7.46 – 7.40 (m, 2H), 7.36 – 7.30 (m, 1H), 7.20 (s, 0.04H), 7.06 (s, 0.04H).

Deuterium incorporation was expected at δ 8.01 – 7.96, δ 7.20 and δ 7.06. Isotopic enrichment values were determined against the integral at δ 7.36 – 7.30.

^{2}H-{^{1}H}NMR (92 MHz, Acetone): δ 7.98 (s, 1.24D), 7.14 (s, 1.92D)

^{13}C-{^{1}H}NMR (100 MHz, Acetone-*d_6*): δ 129.4 (m), 128.8, 125.8.

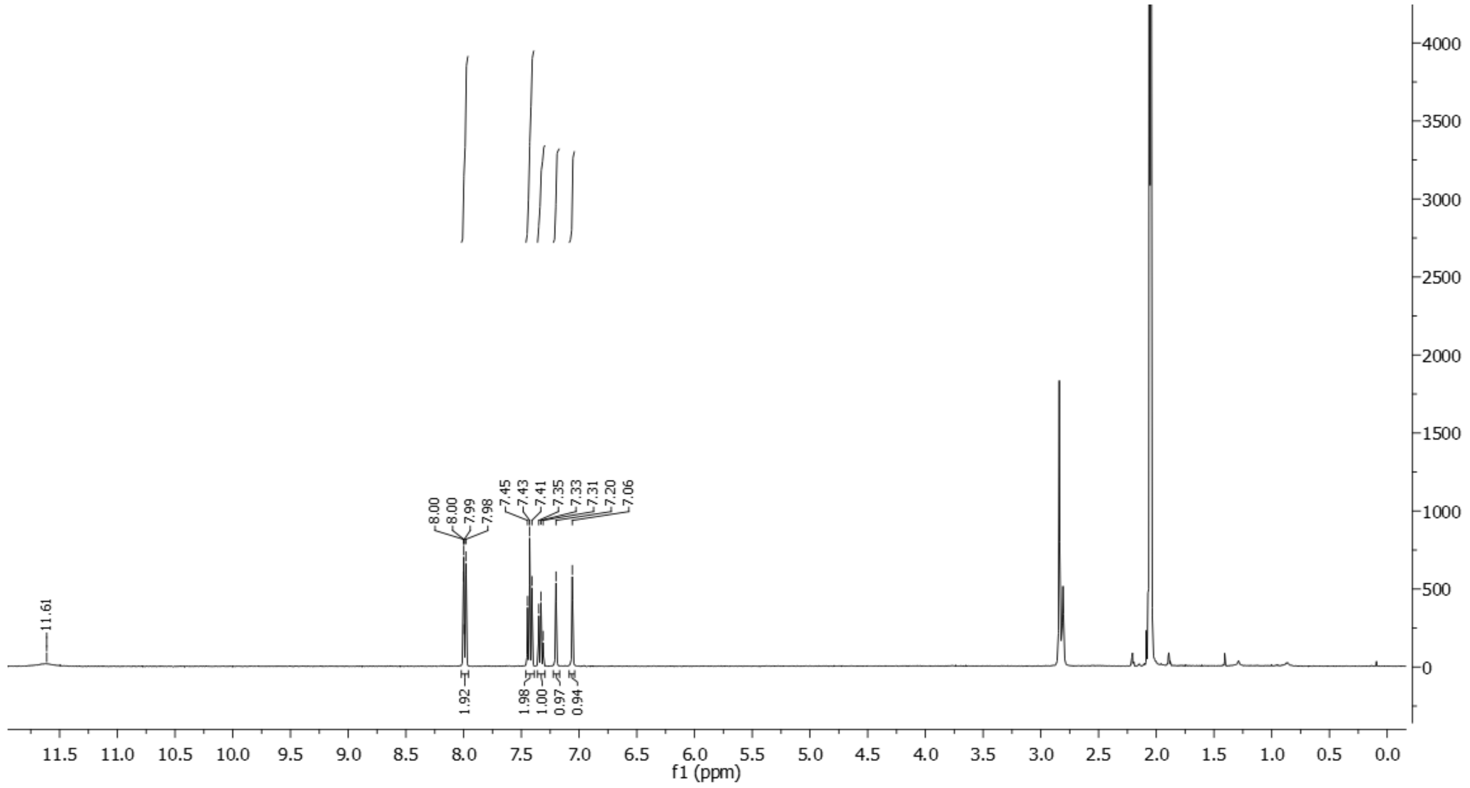

^{1}H-NMR spectrum of the non-deuterated starting material

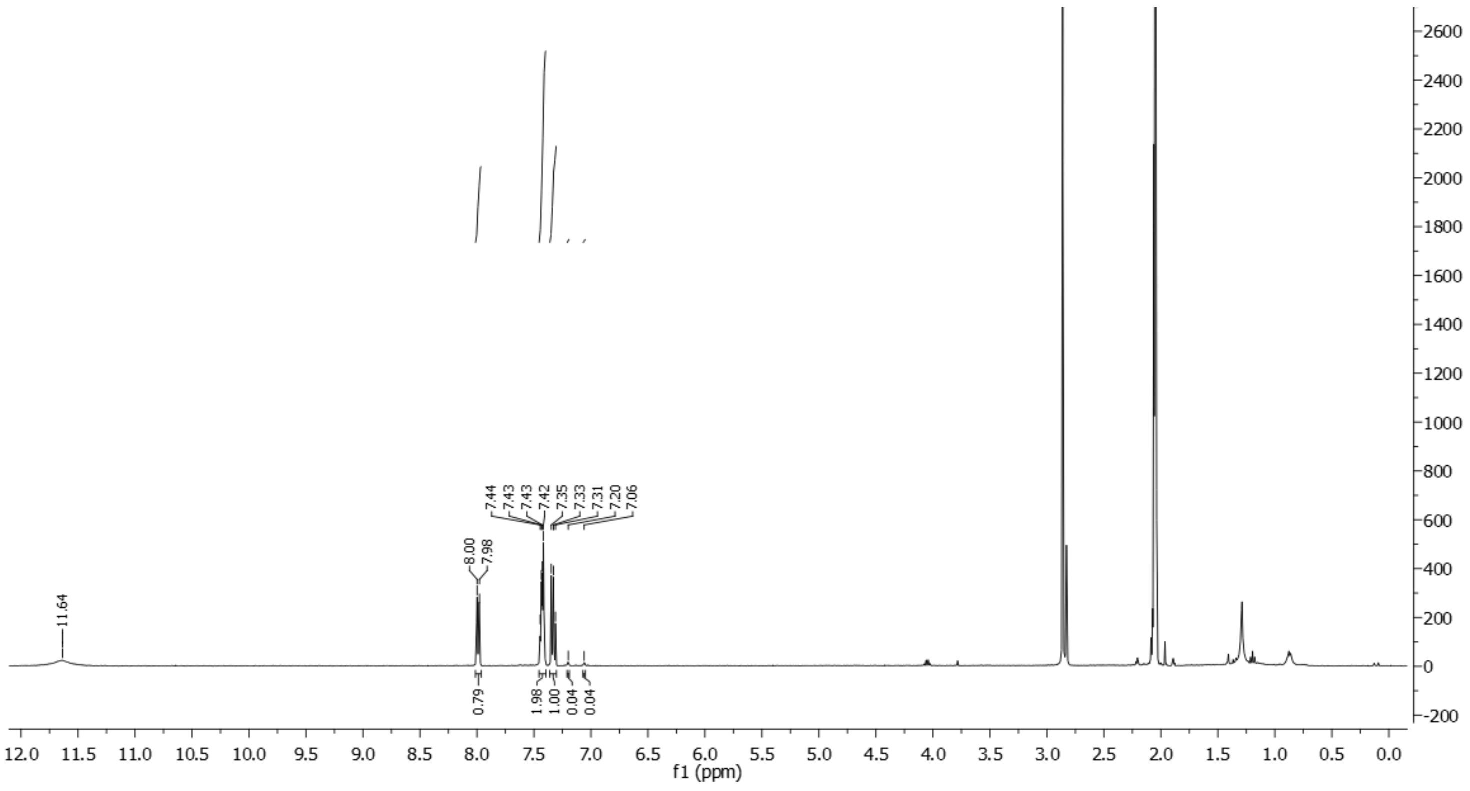

^{1}H-NMR spectrum of **5**

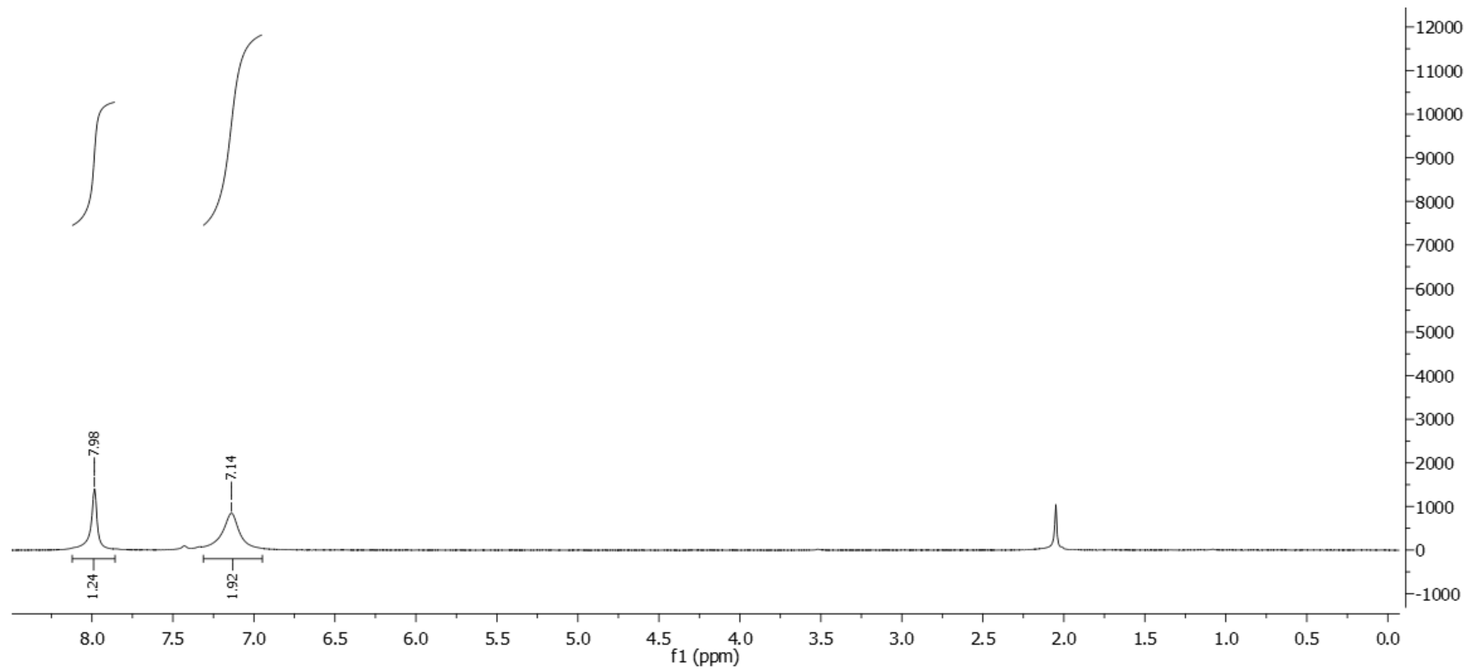

^{2}H-NMR spectrum of **5**

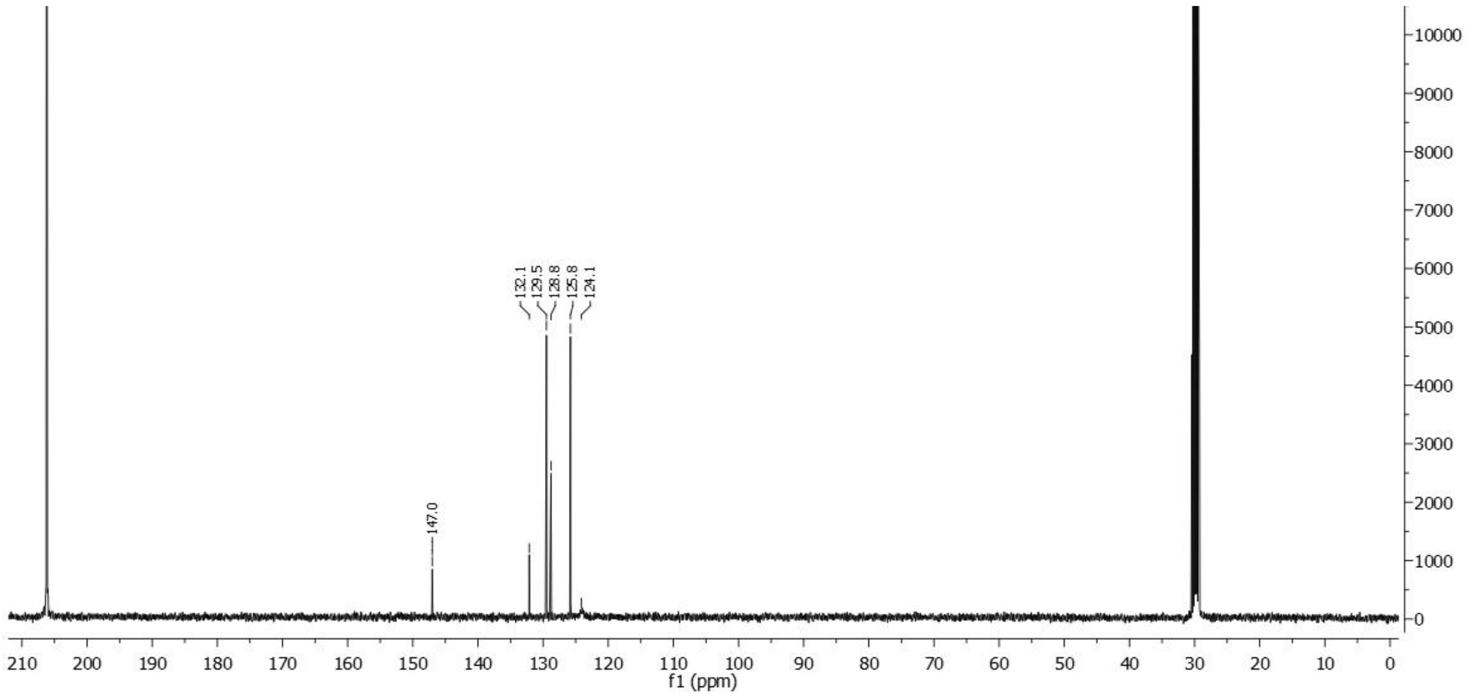

^{13}C-NMR spectrum of the non-deuterated starting material

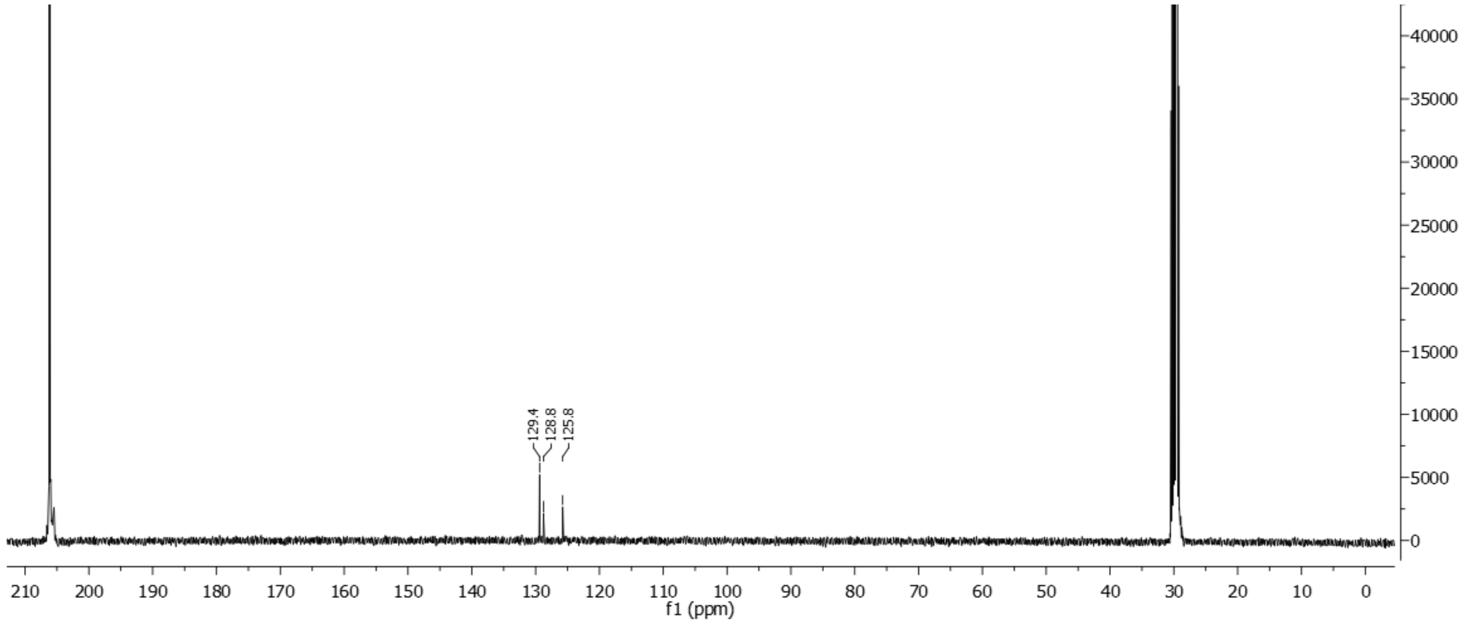

^{13}C-NMR spectrum of **5**

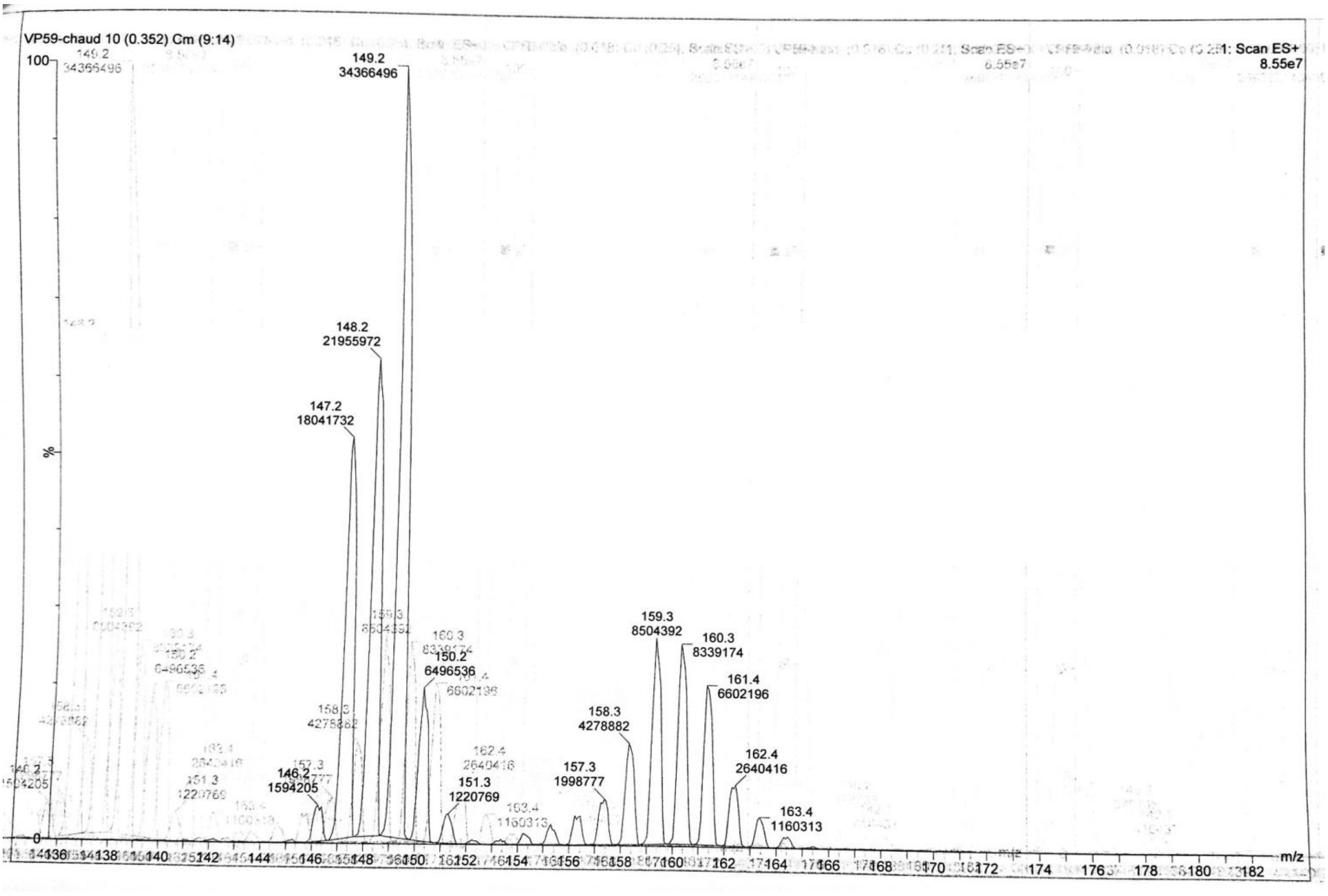

ESI spectrum of **5**

(1-Ethyl-imidazol-2-yl)methanol **6**

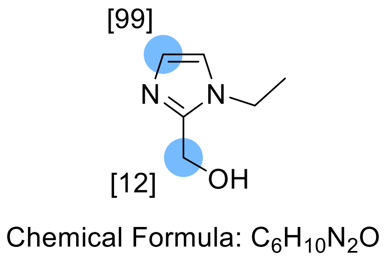

Substrate	Solvent (Volume)	RuNp@PVP cat.
25.2mg, 0.2mmol	DMA (2mL)	14.4mg, 5mol%

Workup and purification:

After cooling down to room temperature, EtOAc : Cy (1:1, 3mL) was added to the reaction mixture and stirred for 10min to let precipitating RuNp@PVP. The suspension was passed through a Sep-Pak® C18 cartridge and then eluted with EtOAc : Cy (1:1, 5mL). The solvent was removed under vacuum.

Yield: 25.0mg, 99%, colourless oil

^{1}H NMR (400 MHz, Acetone-*d$_6$*): δ 7.10 - 7.02 (m, 1H), 6.78 (s, 0.02H), 4.59 (s, 2H), 4.47 (bs, OH), 4.11 (q, J = 7.3 Hz, 2H), 1.39 (t, J = 7.3 Hz, 3H).

Deuterium incorporation was expected at δ 6.78 and at δ 4.59. Isotopic enrichment values were determined against the integral at δ 7.10 - 7.02.

^{2}H-{^{1}H}NMR (92 MHz, Acetone): δ 6.79 (s, 0.99D), 4.55 (s, 0.24D)

^{13}C-{^{1}H}NMR (100 MHz, Acetone-*d$_6$*): δ 148.1, 127.0 (m), 120.3, 56.5 (m), 41.4, 16.7.

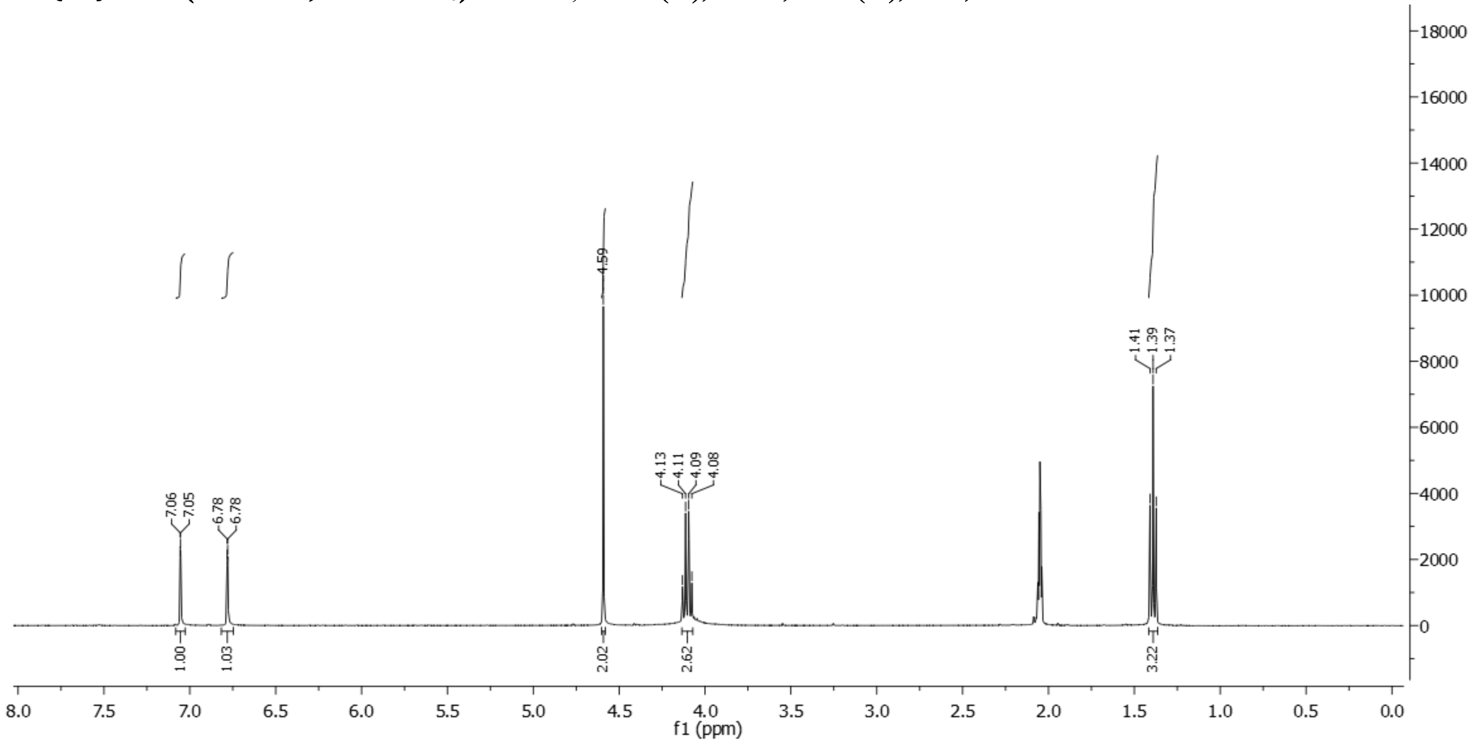

^{1}H-NMR spectrum of the non-deuterated starting material

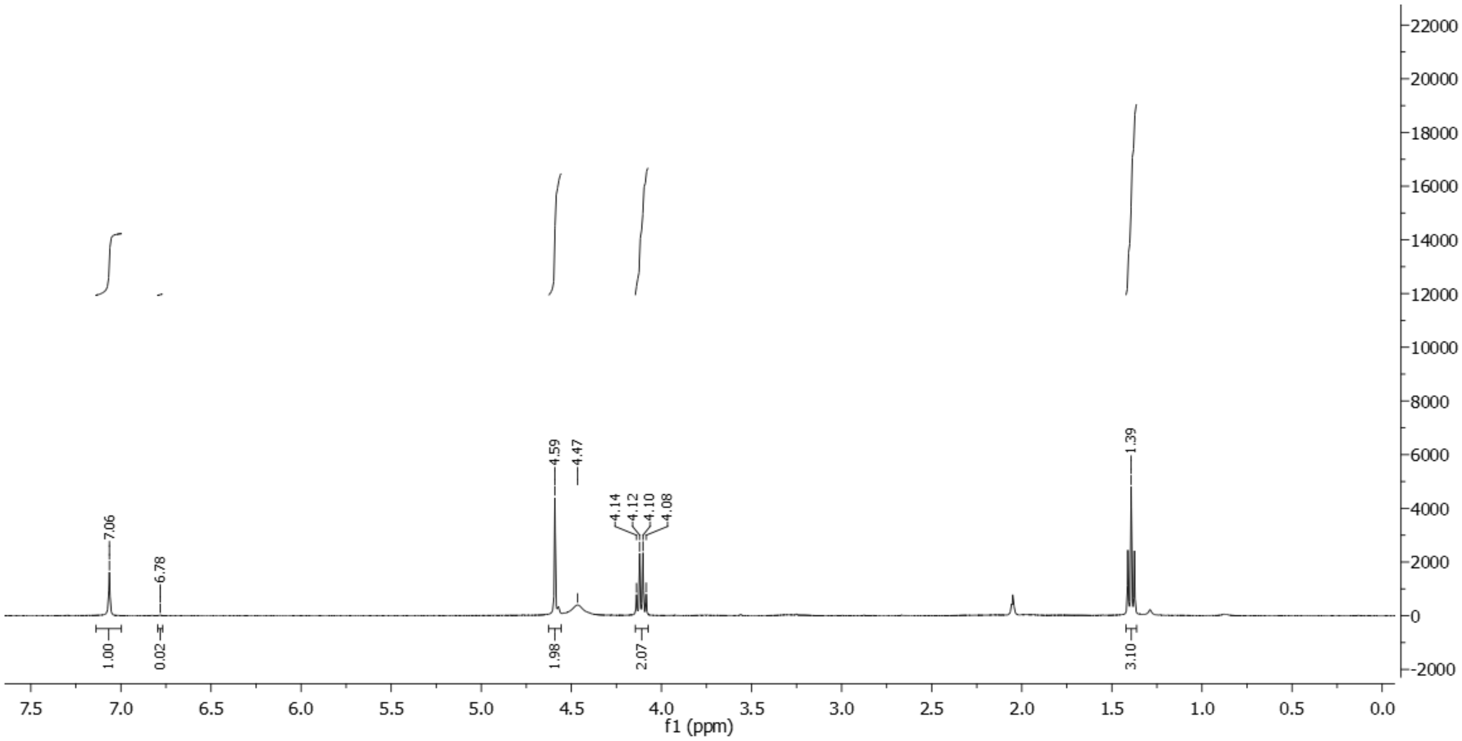

^{1}H-NMR spectrum of **6**

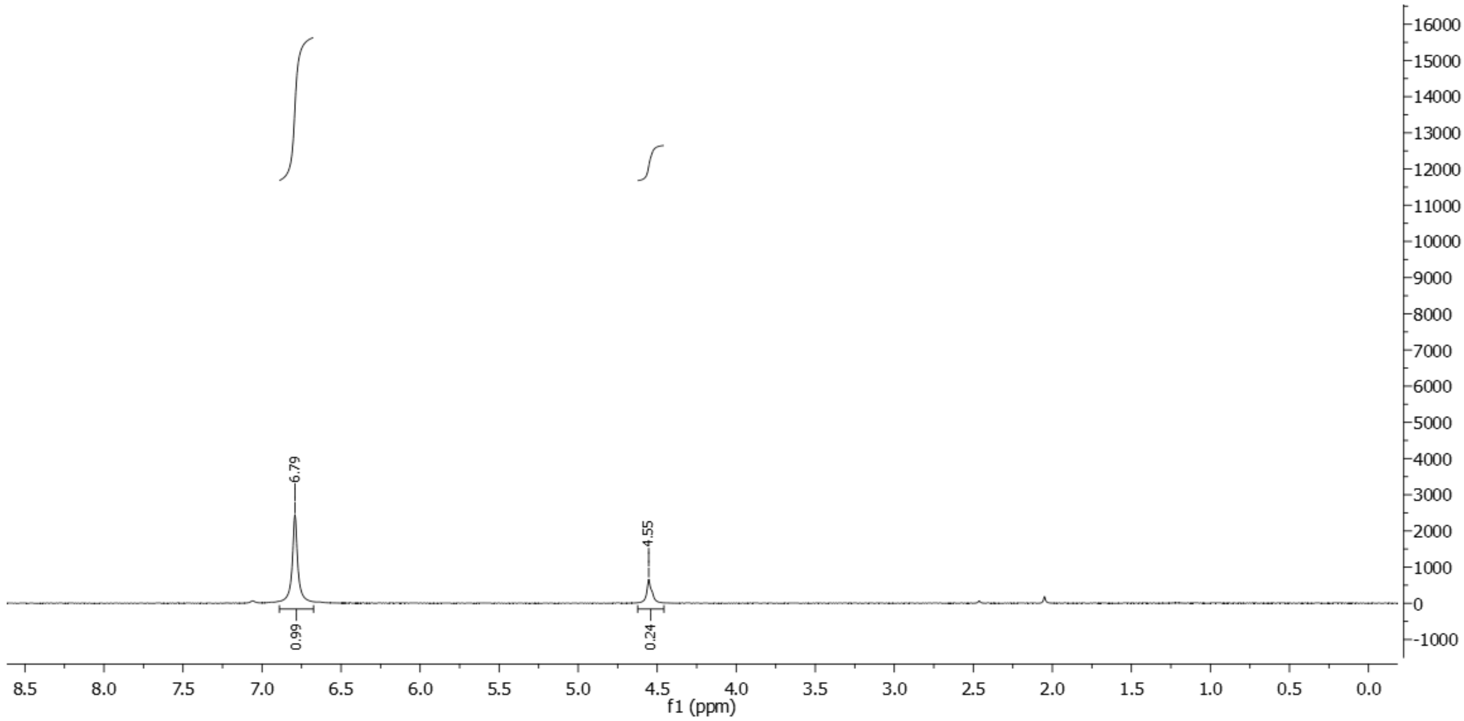

^{2}H-NMR spectrum of **6**

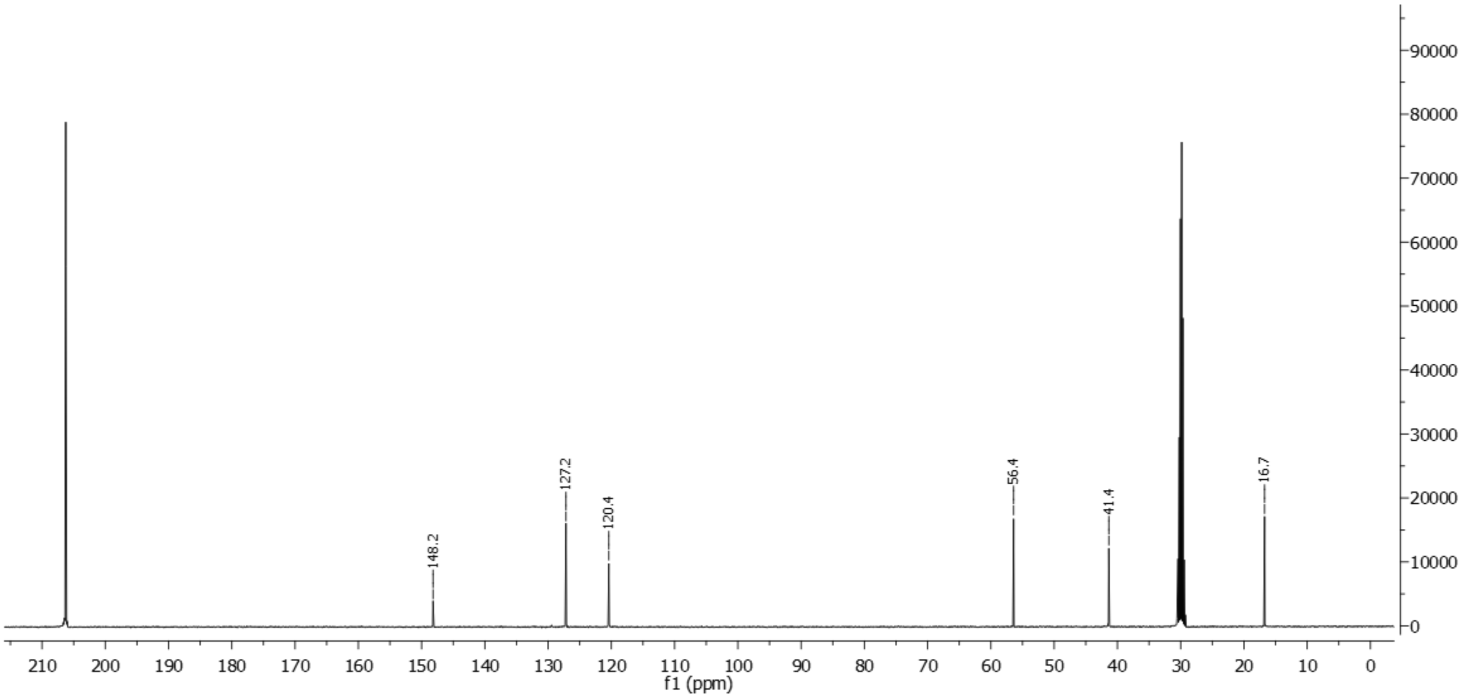

^{13}C-NMR spectrum of the non-deuterated starting material

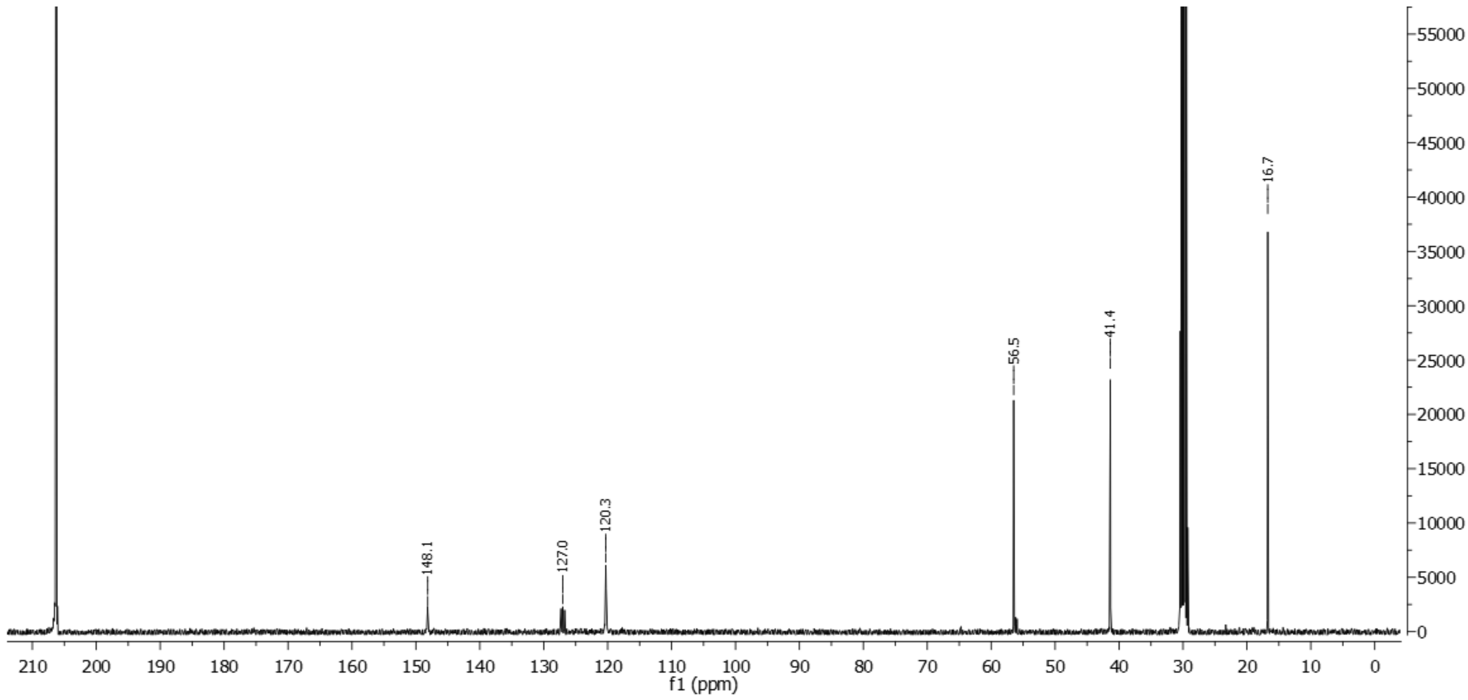

^{13}C-NMR spectrum of **6**

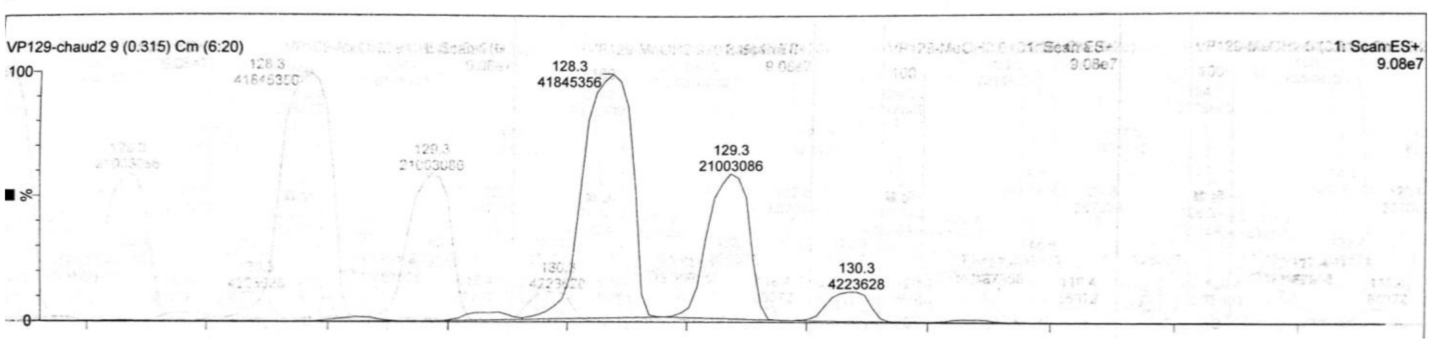

ESI spectrum of **6**

Benzimidazole **7**

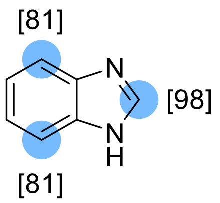

Chemical Formula: $C_7H_6N_2$

Substrate	Solvent (Volume)	RuNp@PVP cat.
23.6mg, 0.2mmol	THF (2mL)	14.4mg, 5mol%

Workup and purification:

After cooling down to room temperature, cyclohexane (2mL) was added to the reaction mixture and stirred for 10min to let precipitating RuNp@PVP. The suspension was passed through a Sep-Pak® C18 cartridge and then eluted with EtOAc : Cy (1:1, 5mL). The solvent was removed under vacuum.

Yield: 25.0mg, 99%, white solid

^{1}H NMR (400 MHz, Acetone-*d$_6$*): δ 8.18 (s, 0.03H), 7.66 – 7.59 (m, 0.39H), 7.25 – 7.18 (m, 2H).

Deuterium incorporation was expected at δ 8.18 and at δ 7.66 – 7.59. Isotopic enrichment values were determined against the integral at δ 7.25 – 7.18.

^{2}H-{^{1}H}NMR (92 MHz, Acetone): δ 8.12 (s, 0.97D), 7.61 (s, 1.62D)

^{13}C-{^{1}H}NMR (100 MHz, Acetone-*d$_6$*): δ 142.0 (m), 122.8 (m).

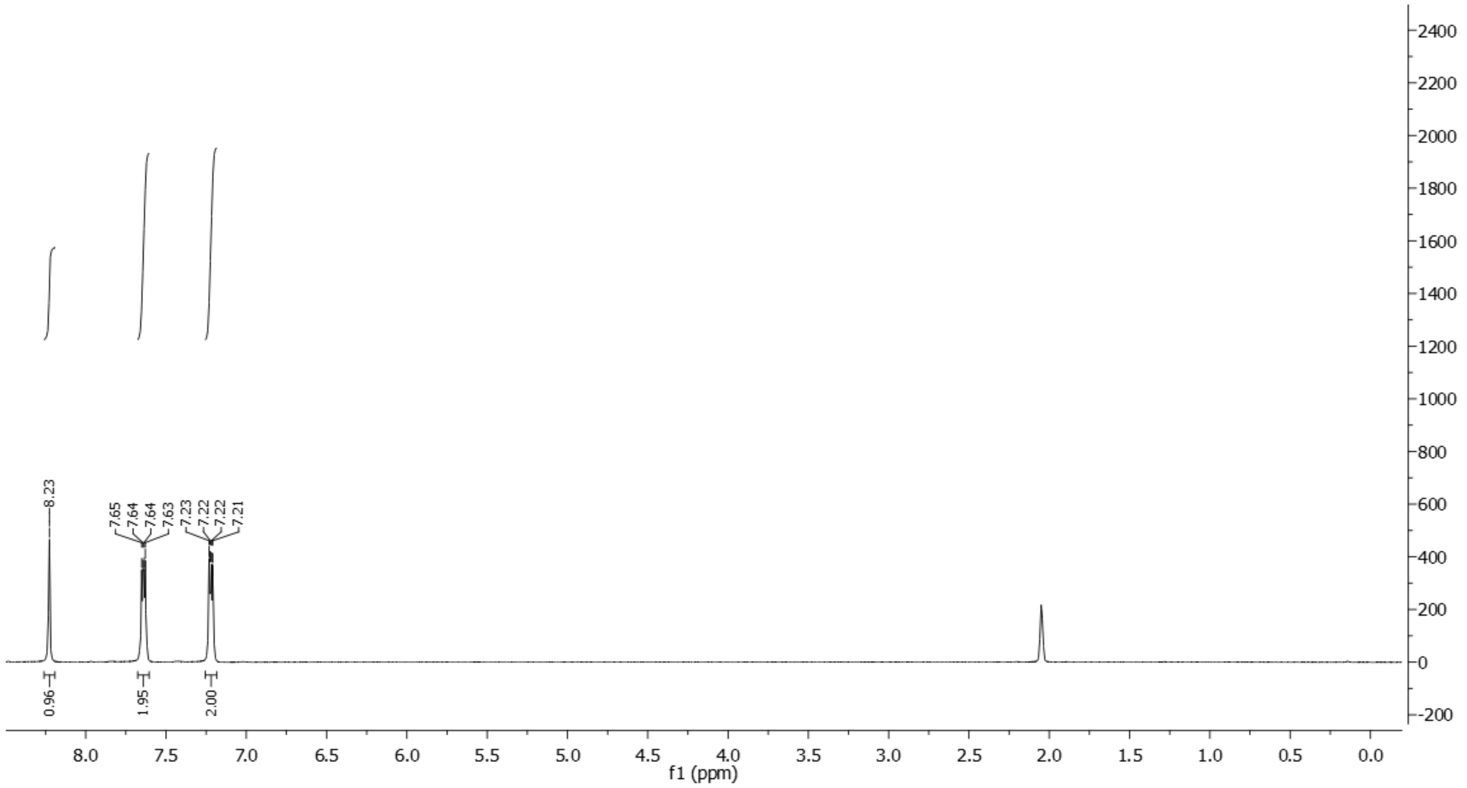

^{1}H-NMR spectrum of the non-deuterated starting material

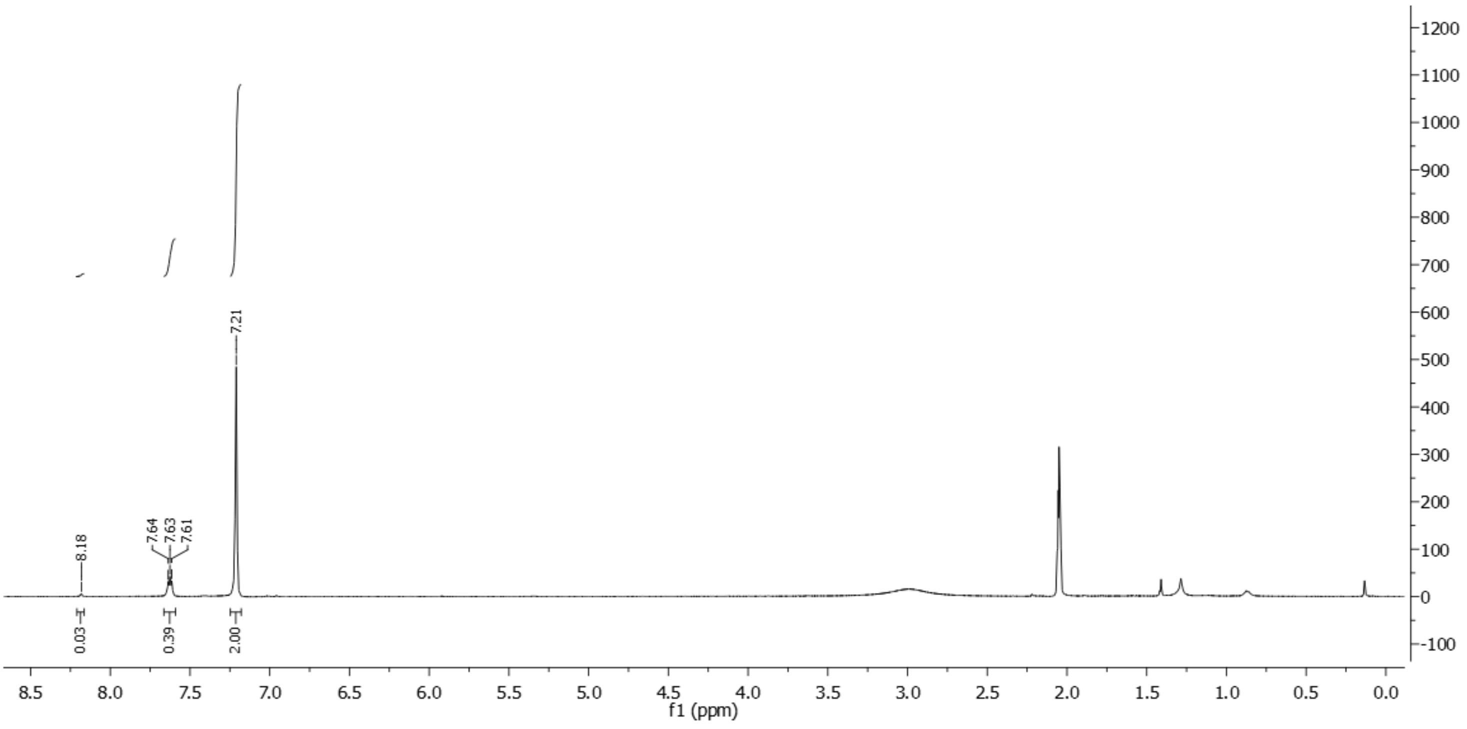

^{1}H-NMR spectrum of **7**

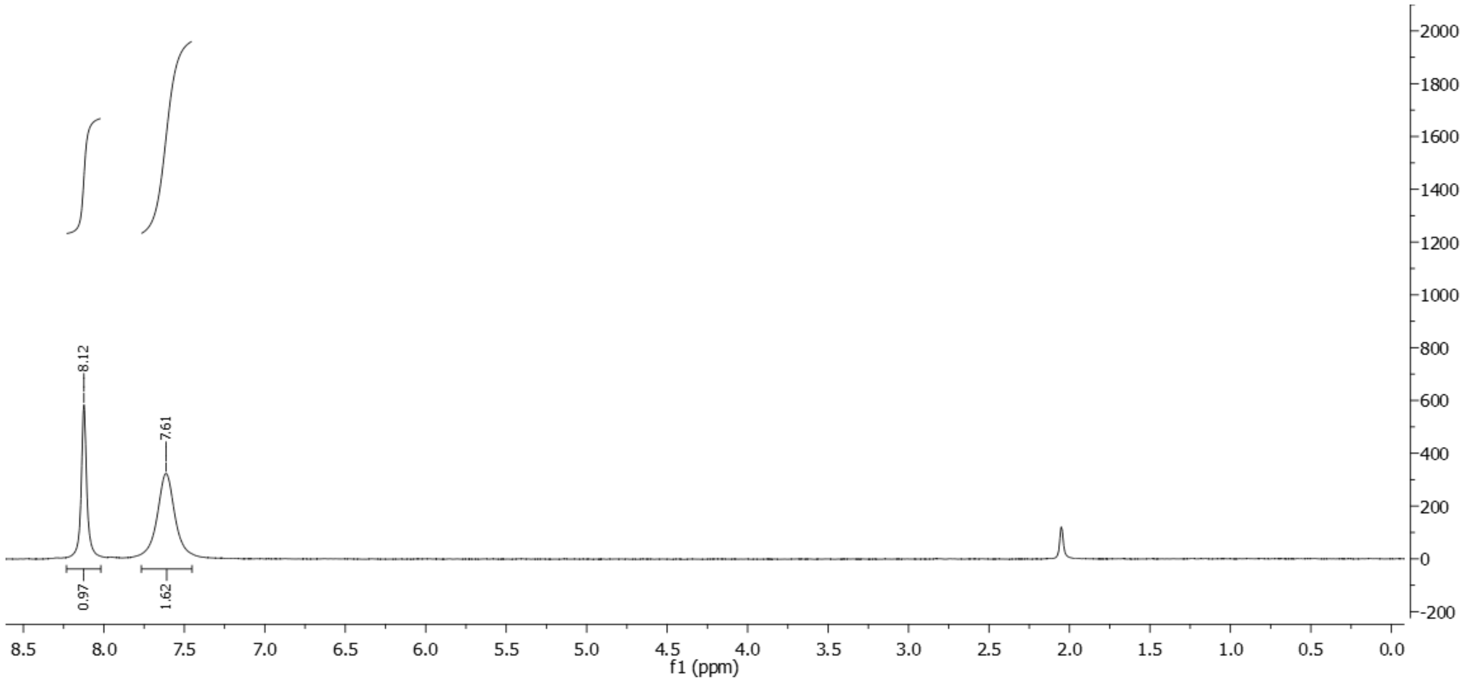

^{2}H-NMR spectrum of **7**

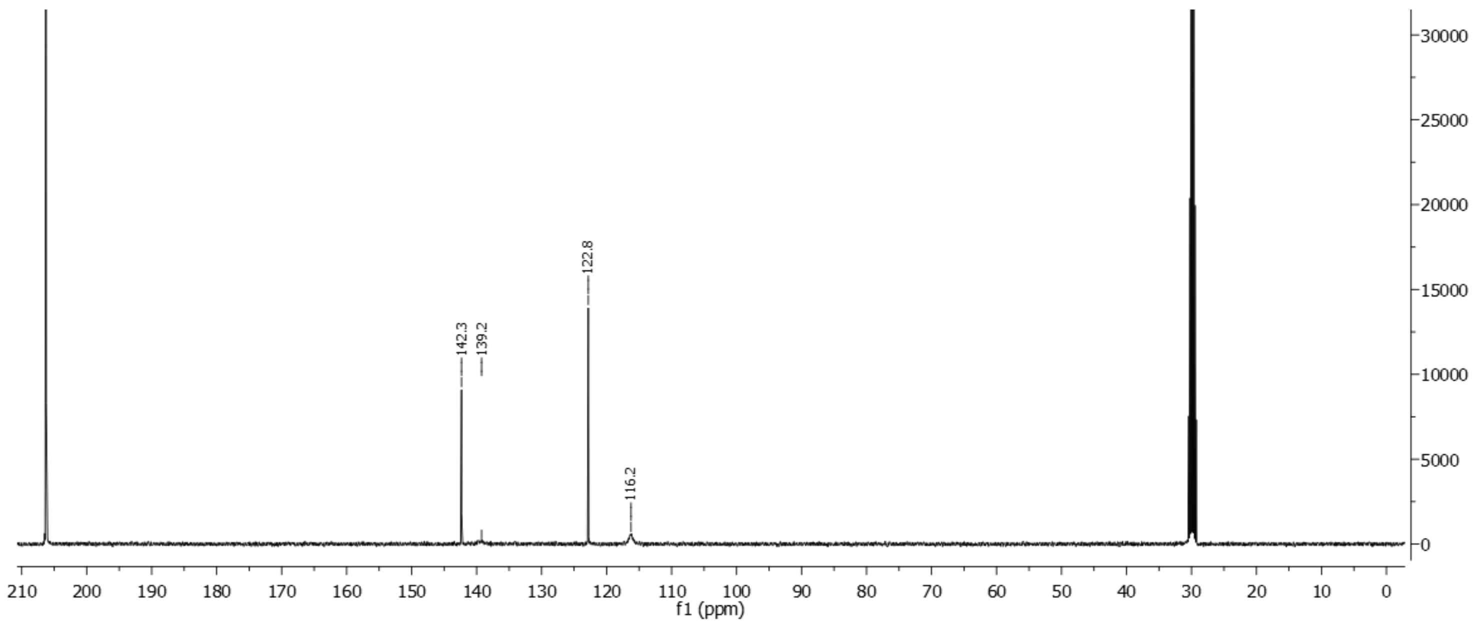

^{13}C-NMR spectrum of the non-deuterated starting material

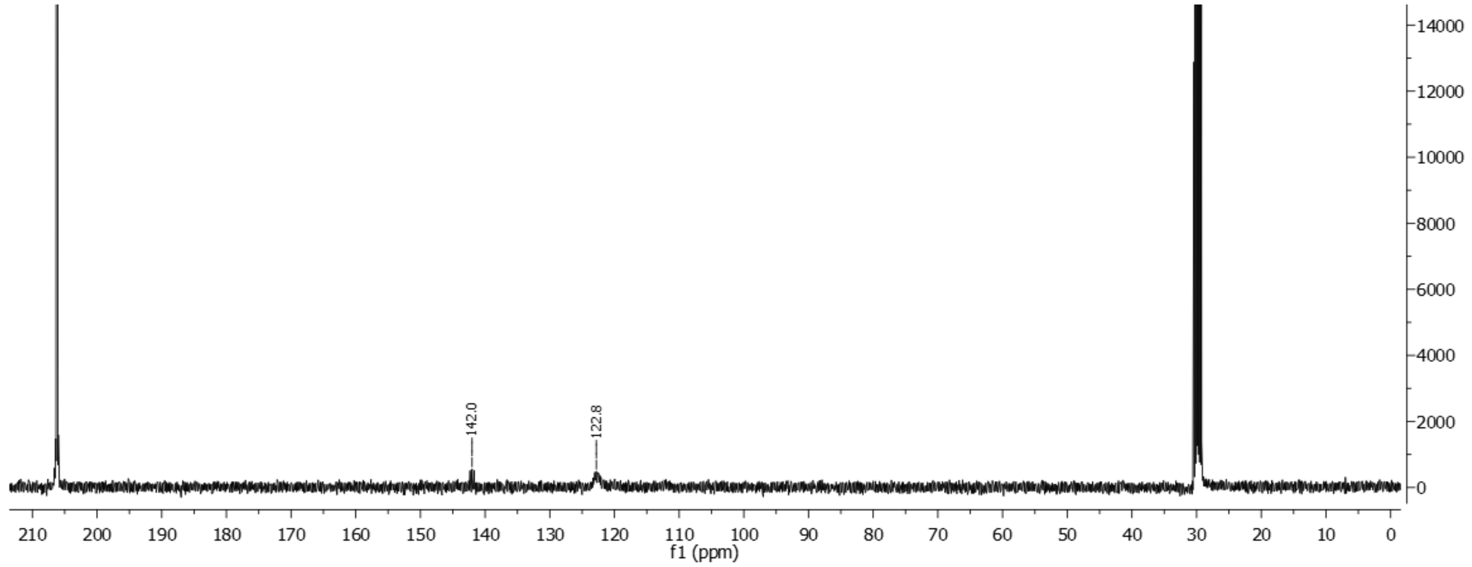

^{13}C-NMR spectrum of **7**

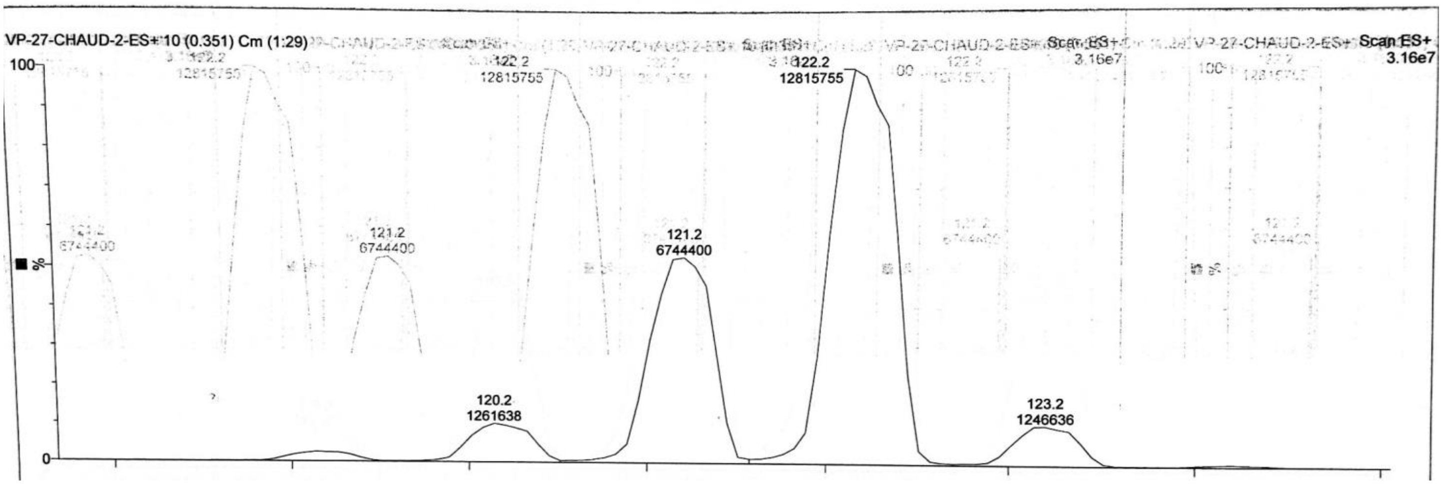

ESI spectrum of **7**

2-Methyl-benzimidazole **8** (first deuteration run)

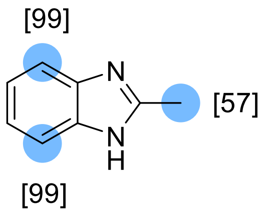

Chemical Formula: $C_8H_8N_2$

Substrate	Solvent (Volume)	RuNp@PVP cat.
26.4mg, 0.2mmol	THF (2mL)	14.4mg, 5mol%

Workup and purification:

After cooling down to room temperature, cyclohexane (2mL) was added to the reaction mixture and stirred for 10min to let precipitating RuNp@PVP. The suspension was passed through a Sep-Pak® C18 cartridge and then eluted with EtOAc : Cy (1:1, 5mL). The solvent was removed under vacuum.

Yield: 26.0mg, 99%, white solid

^{1}H NMR (400 MHz, Acetone-*d*$_6$): δ 7.51 – 7.46 (m, 0.06H), 7.18 – 7.08 (m, 2H), 2.57 – 2.51 (m, 1.28H).

Deuterium incorporation was expected at *δ* 7.51 – 7.46 and at *δ* 2.57 – 2.51. Isotopic enrichment values were determined against the integral at *δ* 7.18 – 7.08.

^{13}C-{^{1}H}NMR (100 MHz, Acetone-*d*$_6$): δ 152.0, 122.0, 114.8 (m), 14.9 (m).

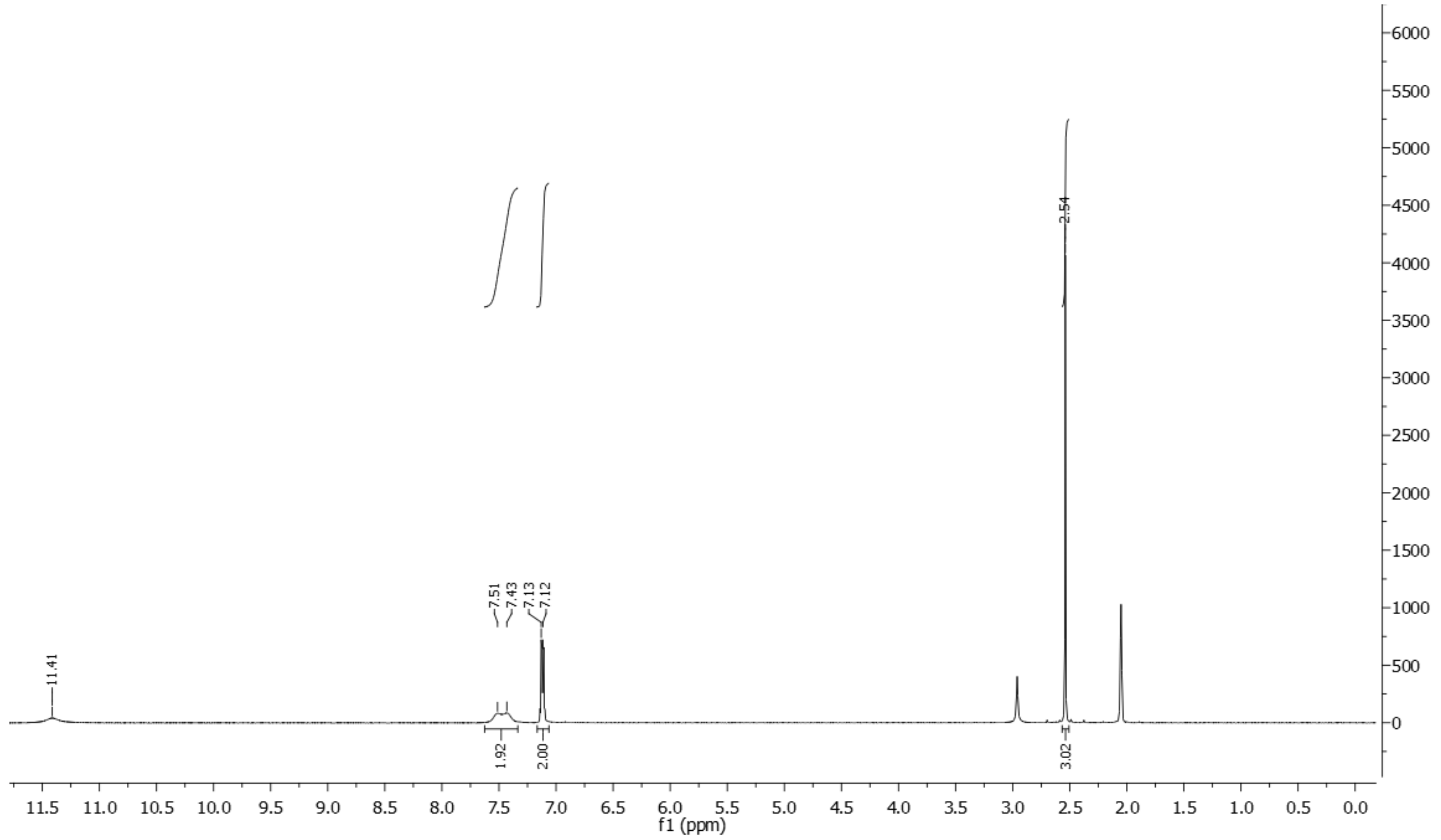

^{1}H-NMR spectrum of the non-deuterated starting material

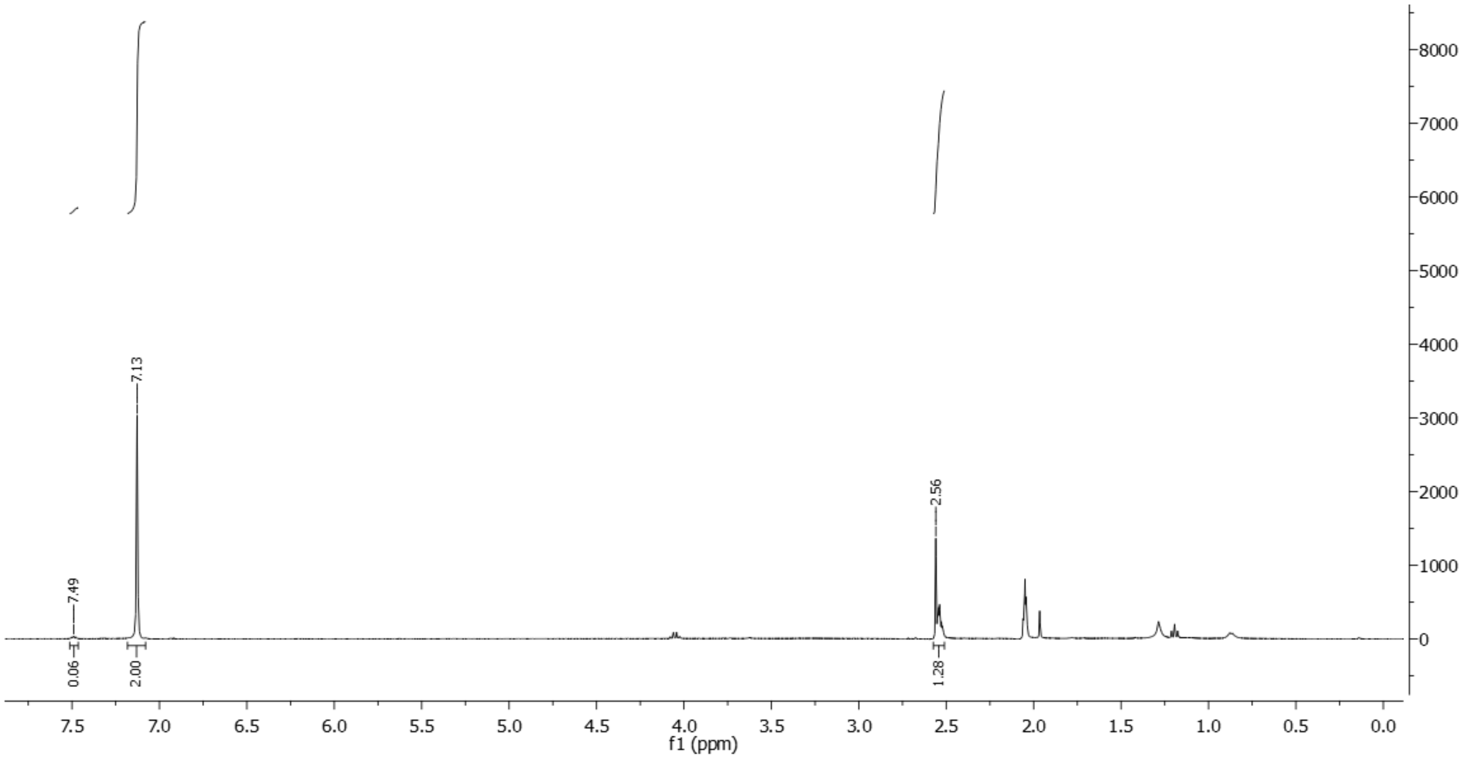

^{1}H-NMR spectrum of **8**

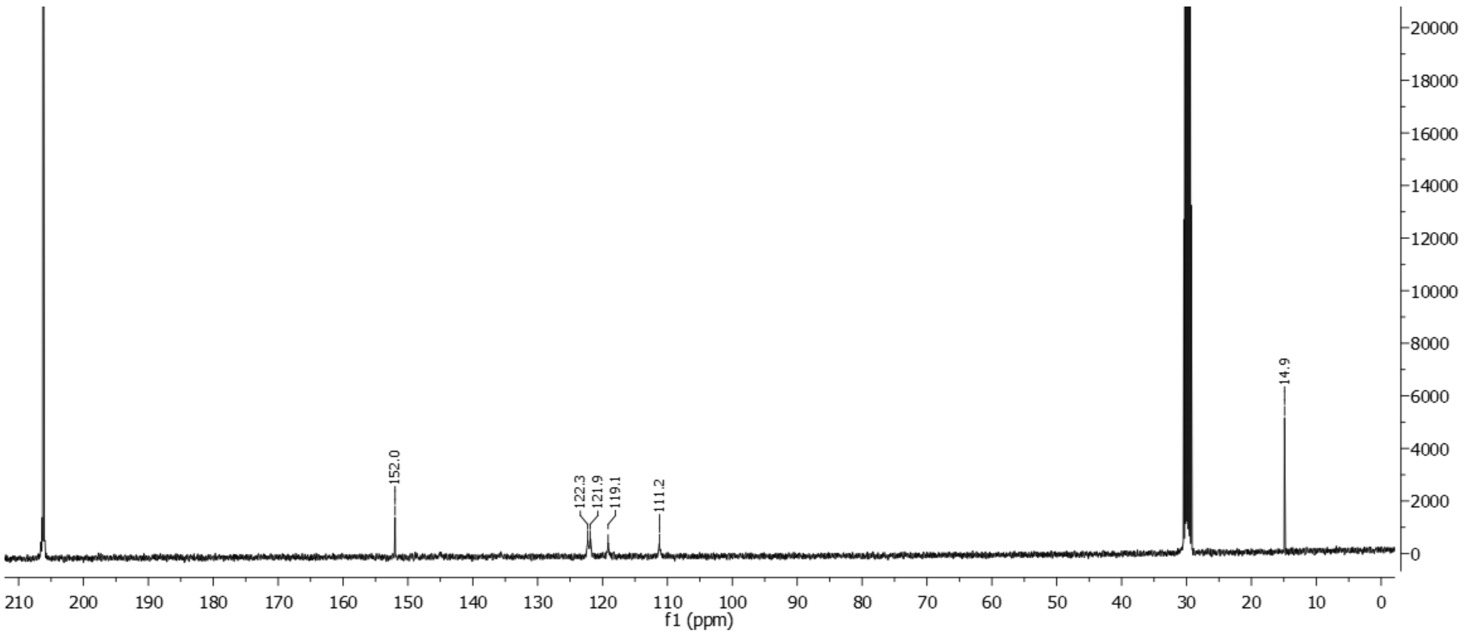

^{13}C-NMR spectrum of the non-deuterated starting material

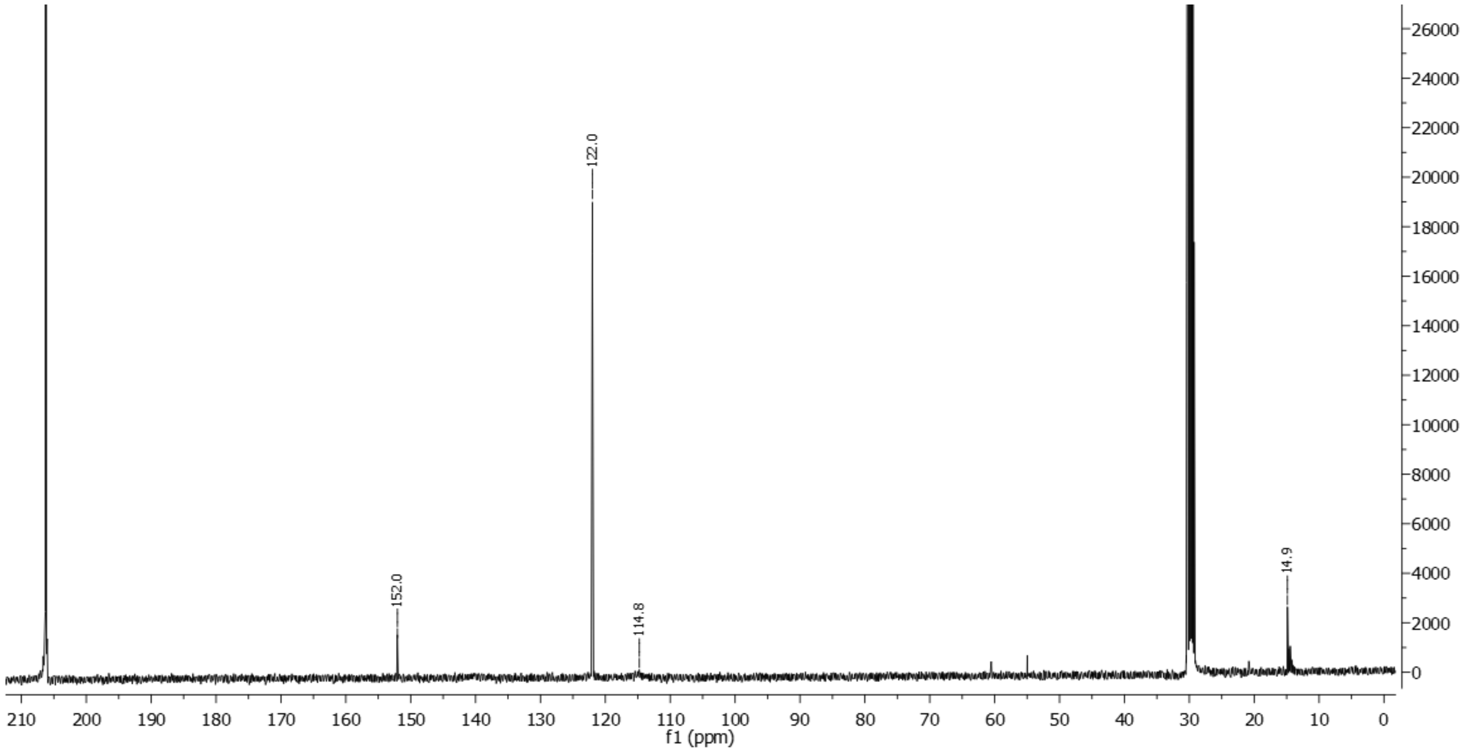

^{13}C-NMR spectrum of **8**

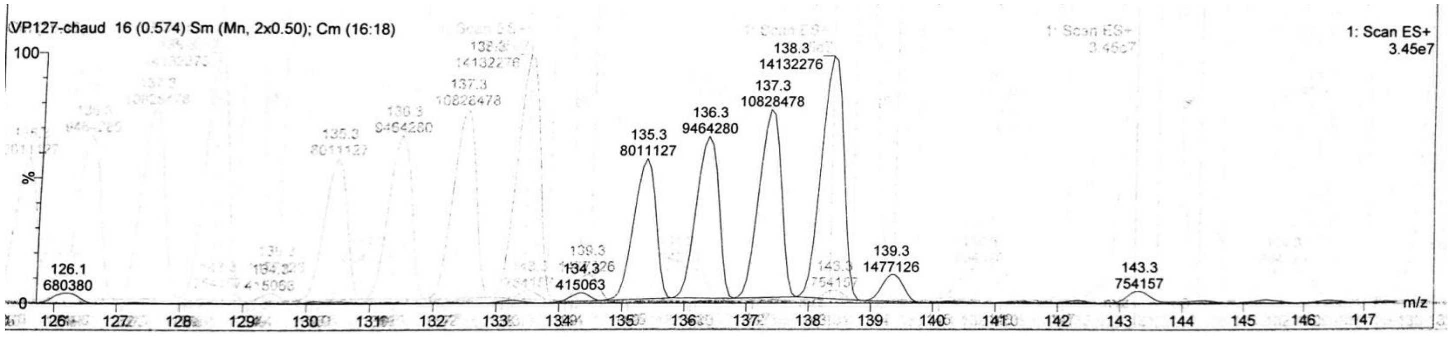

ESI spectrum of **8**

2-Methyl-benzimidazole **8** (second deuteration run)

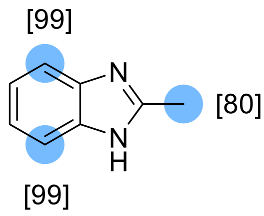

Chemical Formula: $C_8H_8N_2$

Substrate	Solvent (Volume)	RuNp@PVP cat.
26.0mg, 0.2mmol	THF (2mL)	14.4mg, 5mol%

Workup and purification:

After cooling down to room temperature, cyclohexane (2mL) was added to the reaction mixture and stirred for 10min to let precipitating RuNp@PVP. The suspension was passed through a Sep-Pak® C18 cartridge and then eluted with EtOAc : Cy (1:1, 5mL). The solvent was removed under vacuum.

Yield: 26.0mg, 99%, white solid

^{1}H NMR (400 MHz, Acetone-*d_6*): δ 7.51 – 7.48 (m, 0.04H), 7.19 – 7.09 (m, 2H), 2.58 – 2.52 (m, 0.59H).

Deuterium incorporation was expected at *δ* 7.51 – 7.48 and at *δ* 2.58 – 2.52. Isotopic enrichment values were determined against the integral at *δ* 7.19 – 7.09.

^{2}H-{^{1}H}NMR (92 MHz, Acetone): δ 7.48 (s, 2D), 2.47 (s, 2.38D)

^{13}C-{^{1}H}NMR (100 MHz, Acetone-*d_6*): δ 152.0, 122.0, 114.9 (m), 14.4 (m).

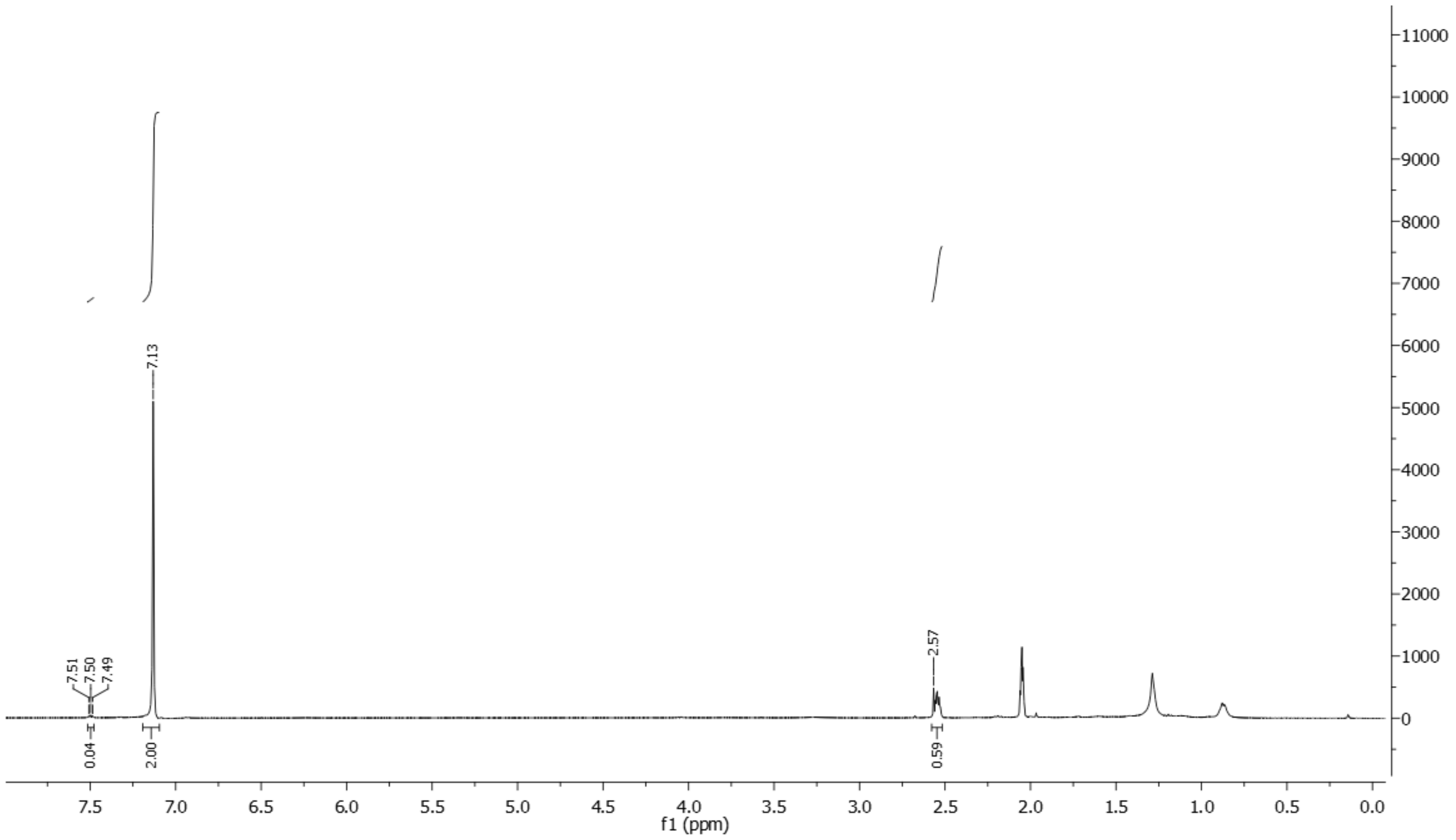

^{1}H-NMR spectrum of **8** (2nd run)

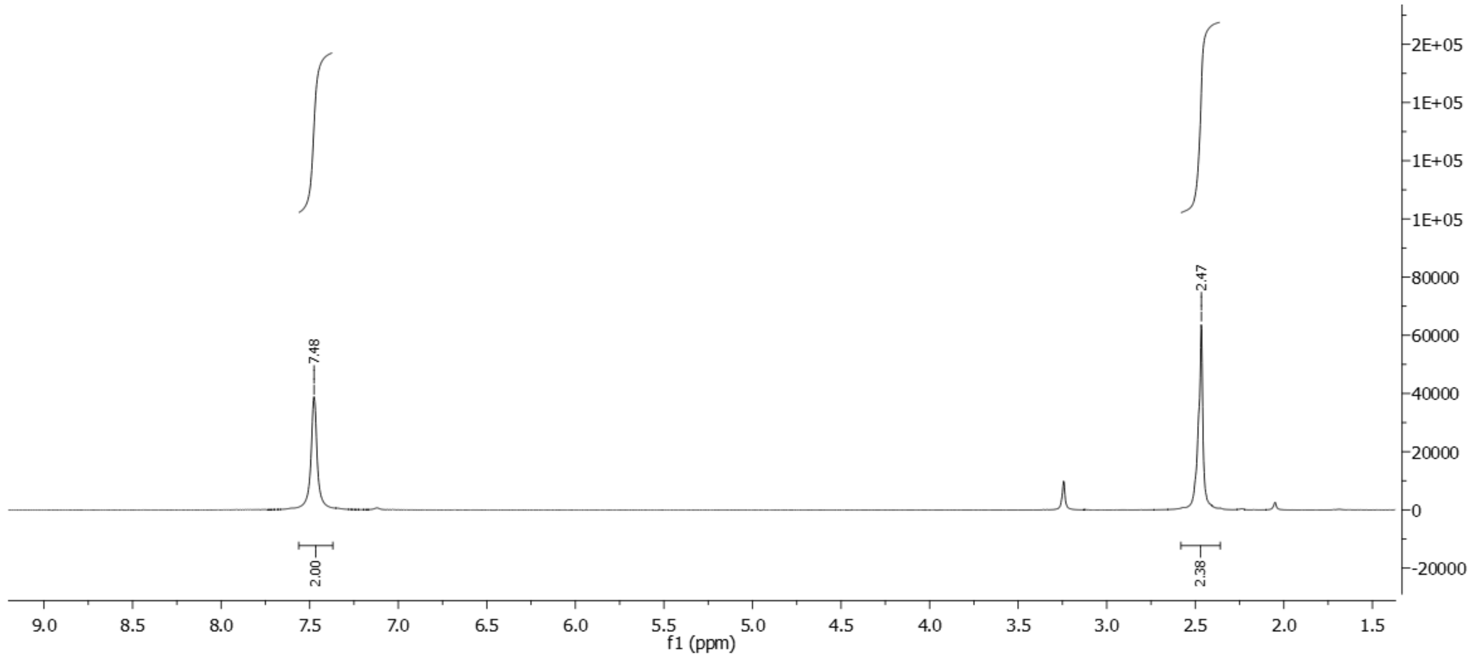

^{2}H-NMR spectrum of **8** (2nd run)

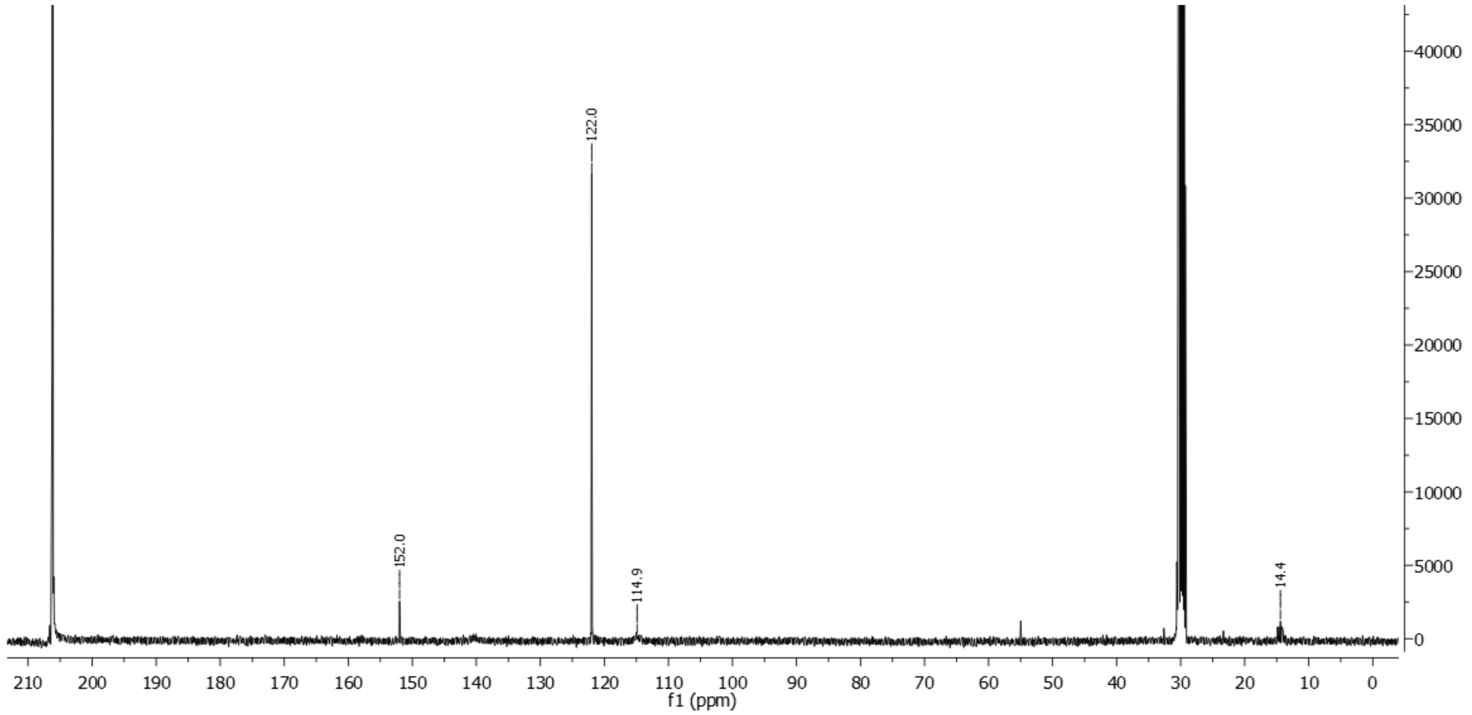

^{13}C-NMR spectrum of **8** (2nd run)

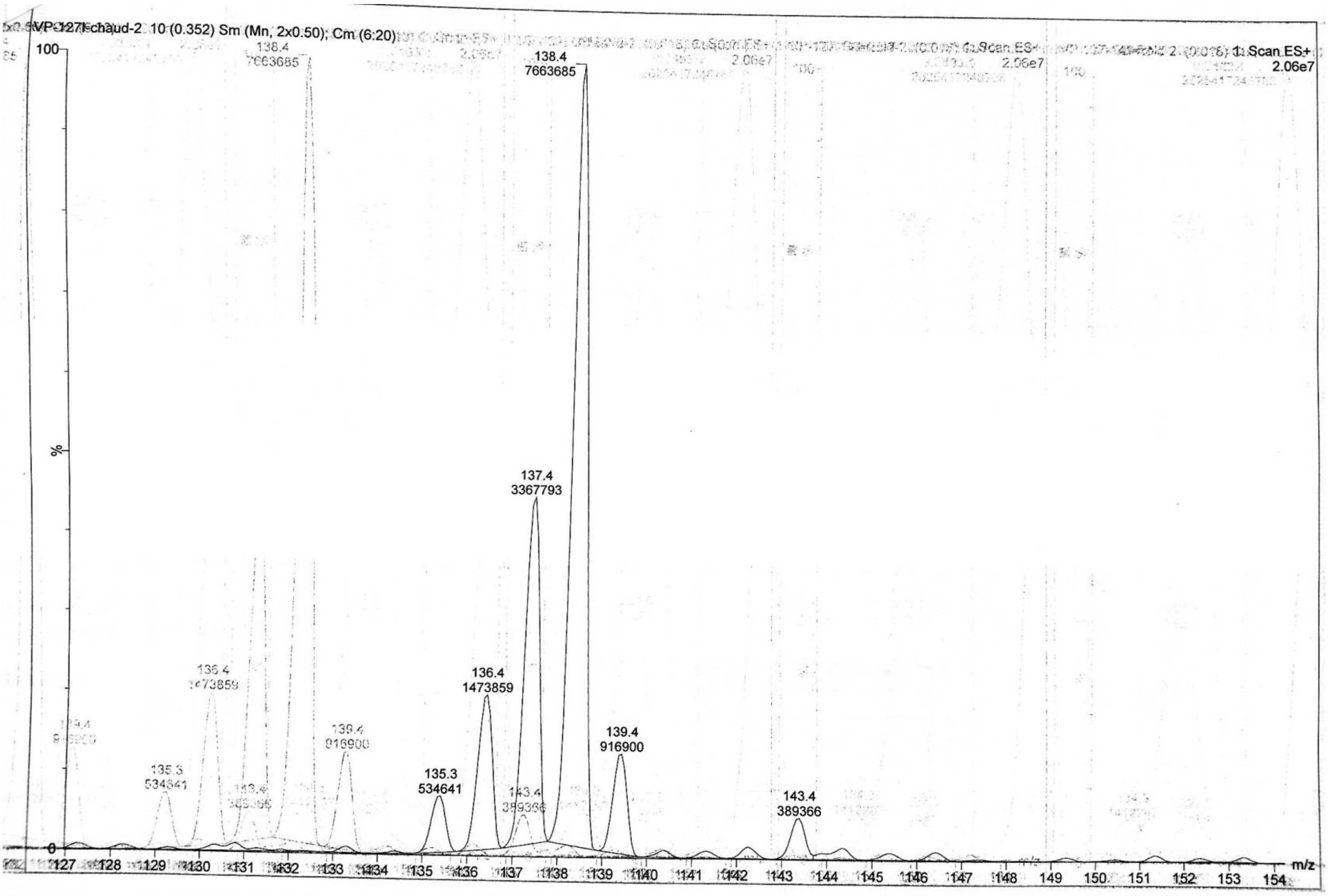

ESI spectrum of **8** (2nd run)

1-Phenyl-1*H*-1,2,4-triazole **9**

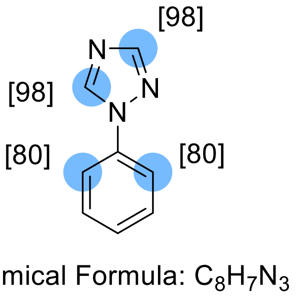

Substrate	Solvent (Volume)	RuNp@PVP cat.	Reaction time
29.0mg, 0.2mmol	THF (0.5mL)	14.4mg, 5mol%	48h

Workup and purification:

After cooling down to room temperature, EtOAc (3mL) was added to the reaction mixture and stirred for 10min to let precipitate RuNp@PVP. The suspension was passed through a basic Al_2O_3 pad and then eluted with EtOAc (3mL). The solvent was removed under vacuum. The crude product was recrystallized from acetone.

Yield: 18.0mg, 62%, white solid

^{1}H NMR (400 MHz, Acetone-d_6): δ 9.03 (s, 0.02H), 8.10 (s, 0.02H), 7.91 – 7.84 (m, 0.40H), 7.62 – 7.50 (m, 2H), 7.46 – 7.38 (m, 1H).

Deuterium incorporation was expected at *δ* 9.03, *δ* 8.10 and at *δ* 7.91 – 7.84. Isotopic enrichment values were determined against the integral at *δ* 7.46 – 7.38.

^{2}H-{^{1}H}NMR (92 MHz, Acetone): δ 9.01 (m, 0.98D), 8.09 (m, 0.96D), 7.88 (m, 1.60D)

^{13}C-{^{1}H}NMR (100 MHz, Acetone-d_6): δ 153.4 (m), 142.6 (m), 138.2, 130.5, 128.6, 120.4 (m).

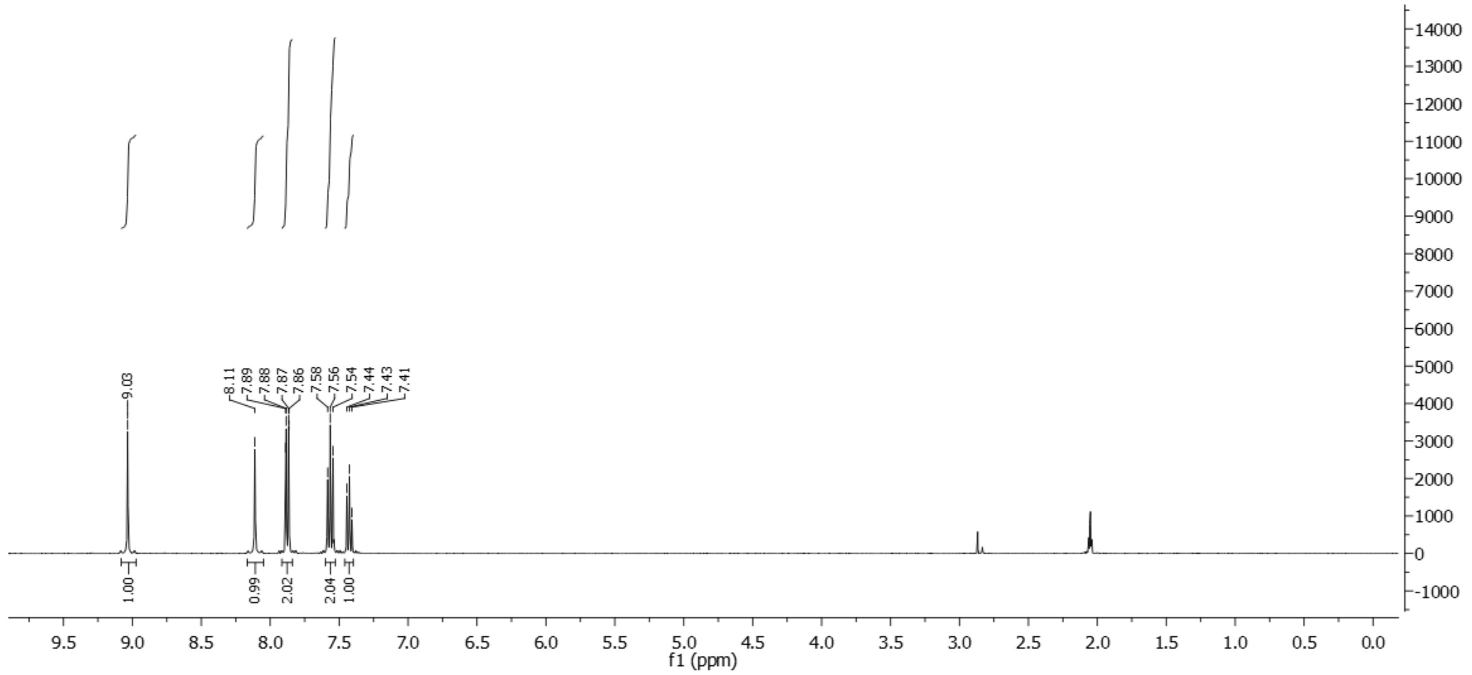

^{1}H-NMR spectrum of the non-deuterated starting material

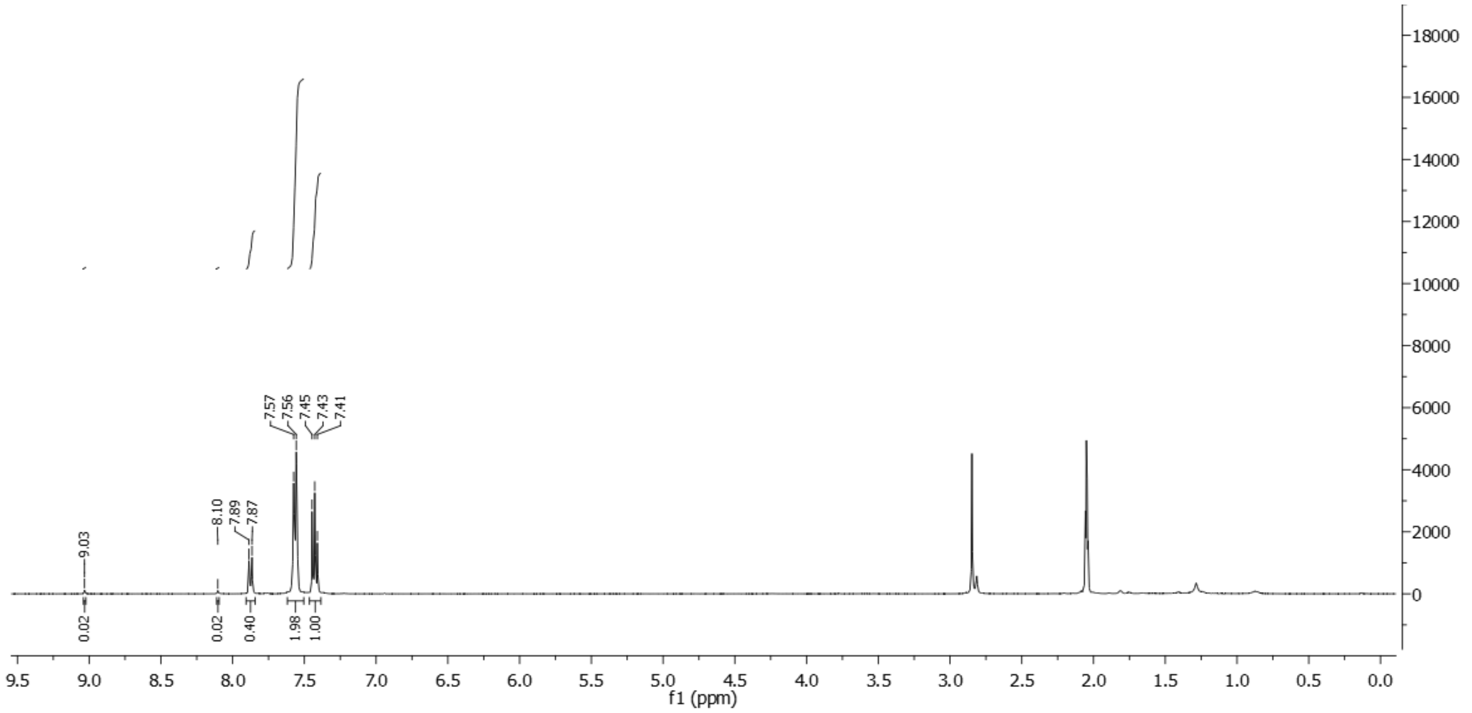

^{1}H-NMR spectrum of **9**

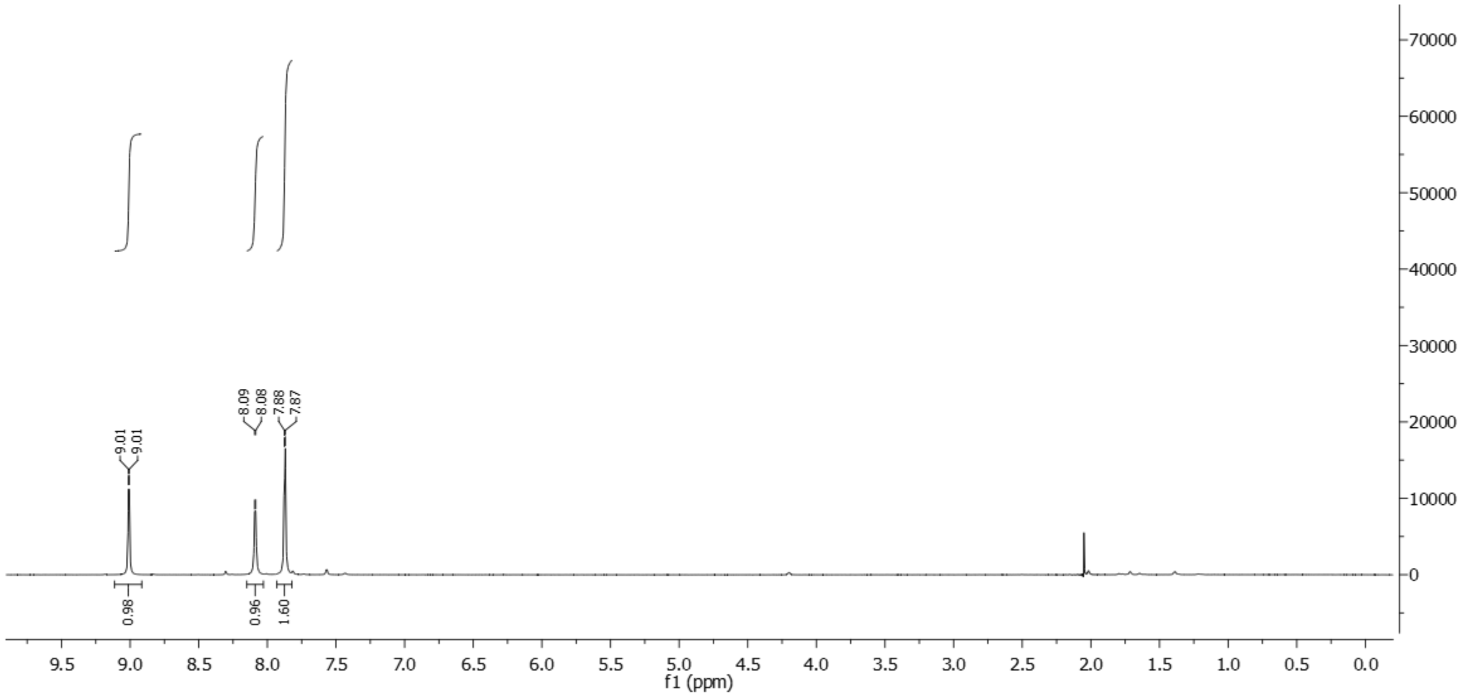

^{2}H-NMR spectrum of **9**

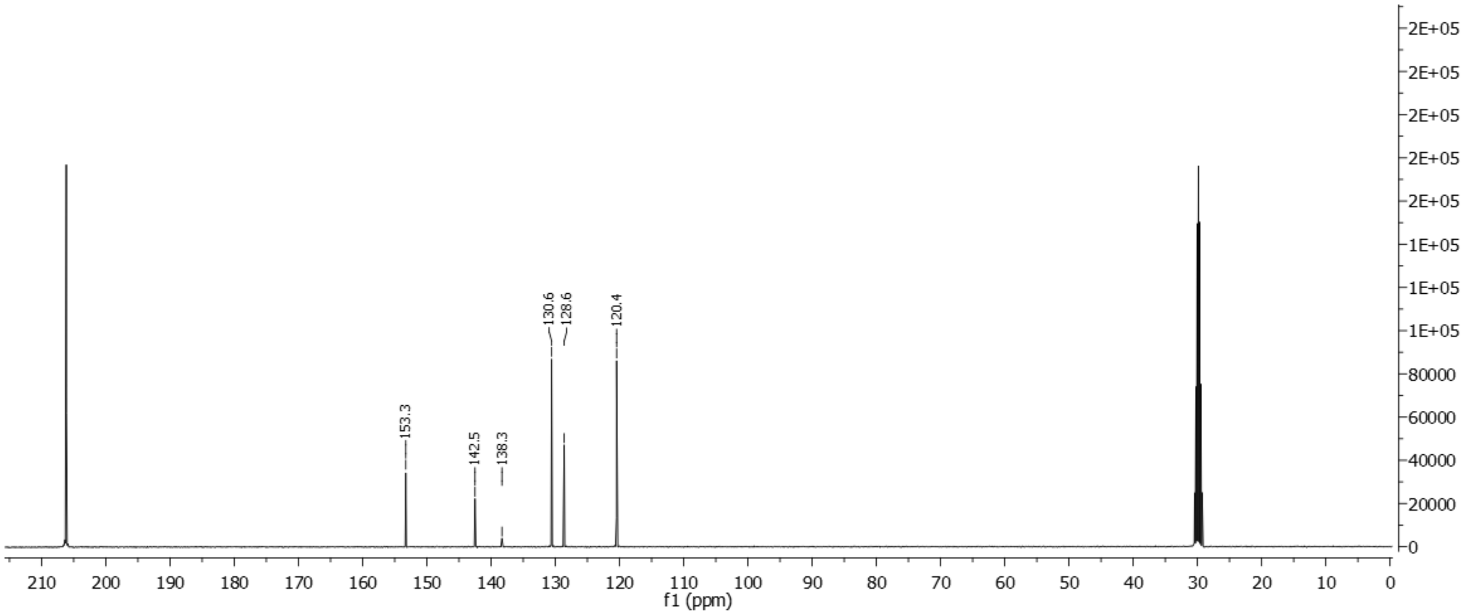

^{13}C-NMR spectrum of the non-deuterated starting material

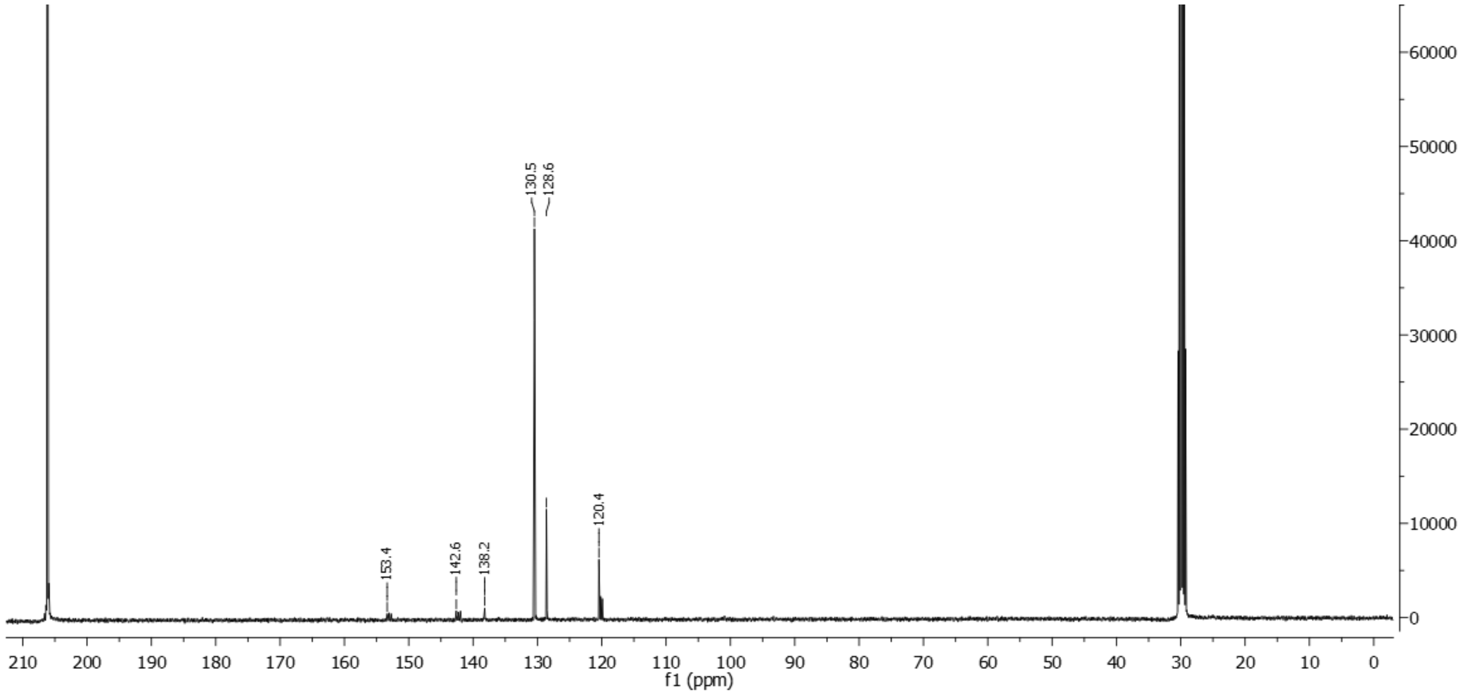

^{13}C-NMR spectrum of **9**

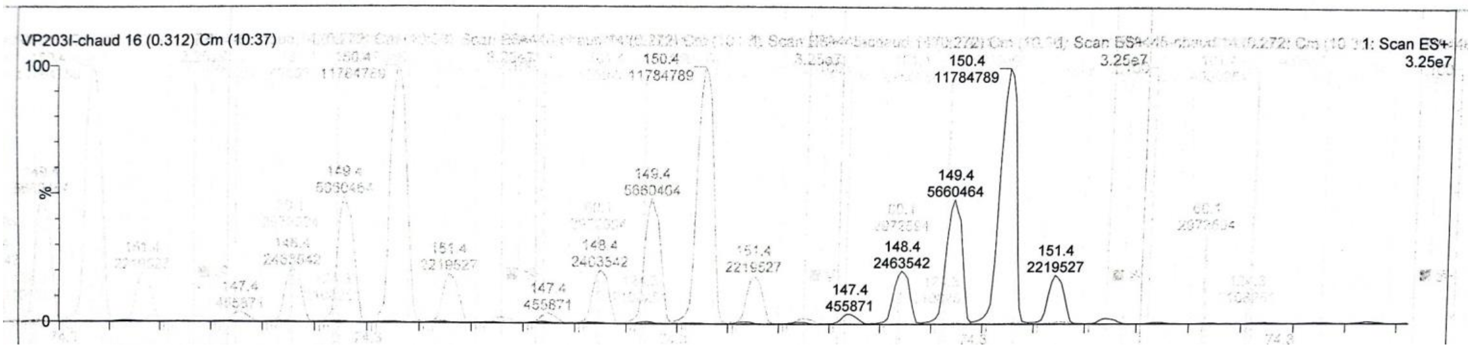

ESI spectrum of **9**

1-(4-Methoxyphenyl)-1*H*-1,2,4-triazole **10**

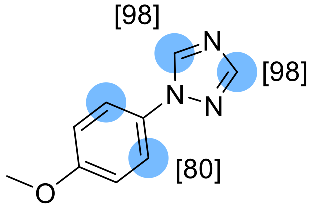

Substrate	Solvent (Volume)	RuNp@PVP cat.
35.0mg, 0.2mmol	THF (2mL)	14.4mg, 5mol%

Workup and purification:

After cooling down to room temperature, EtOAc : Cy (1:1, 3mL) was added to the reaction mixture and stirred for 10min to let precipitate RuNp@PVP. The suspension was passed through a Sep-Pak® C18 cartridge and then eluted with EtOAc : Cy (1:1, 5mL). The solvent was removed under vacuum.

Yield: 37.0mg, 99%, white solid

^{1}H NMR (400 MHz, Acetone-*d_6*): δ 8.90 (s, 0.02H), 8.06 (s, 0.02H), 7.81 – 7.71 (m, 0.41H), 7.14 – 7.06 (m, 2H), 3.86 (s, 3H).

Deuterium incorporation was expected at δ 9.05, δ 8.90, δ 8.06 and at δ 7.81 – 7.71. Isotopic enrichment values were determined against the integral at δ 7.14 – 7.06.

^{2}H-{^{1}H}NMR (92 MHz, Acetone): δ 8.91 (s, 0.98D), 8.08 (s, 0.98D), 7.79 (s, 1.59D)

^{13}C-{^{1}H}NMR (100 MHz, Acetone-*d_6*): δ 160.1, 153.0 (m), 142.3 (m), 131.5, 122.1 (m), 115.4, 55.9.

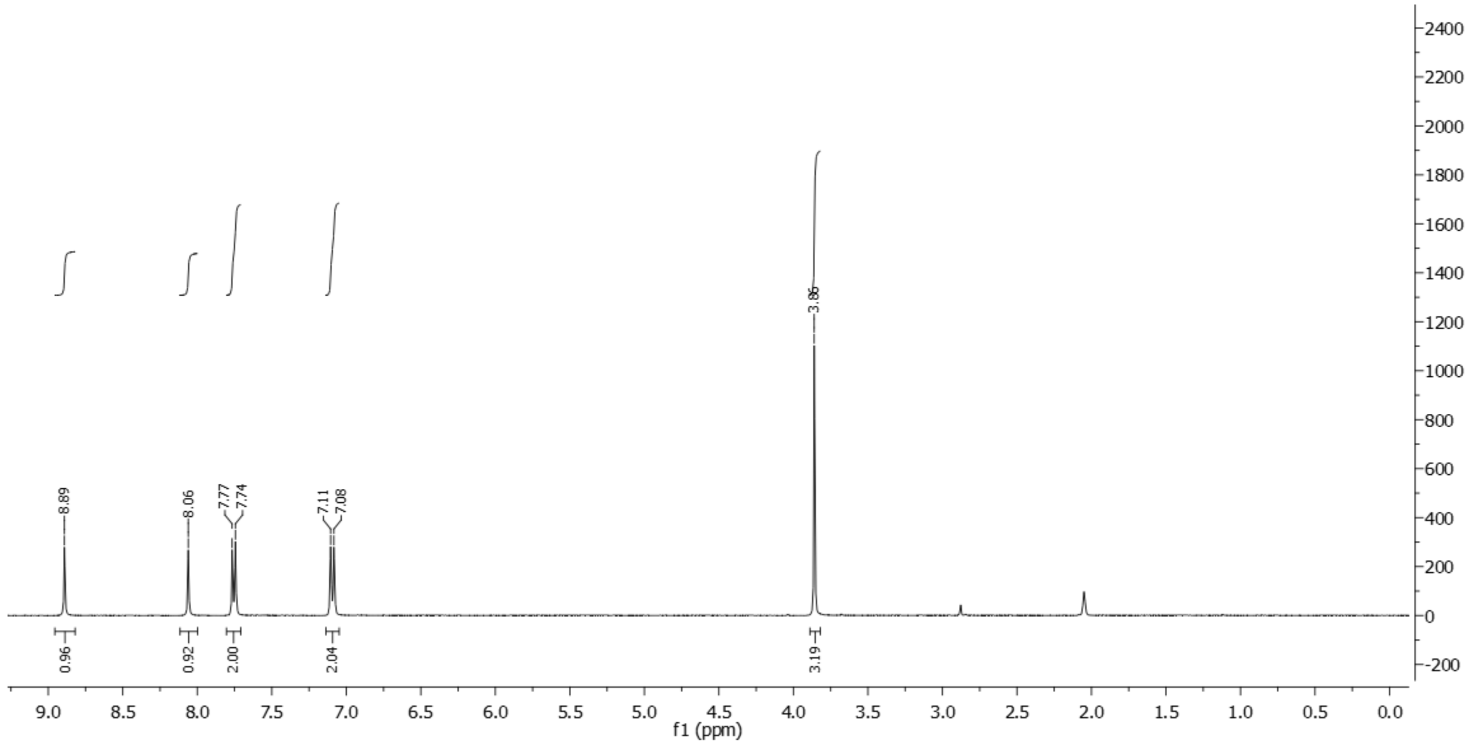

^{1}H-NMR spectrum of the non-deuterated starting material

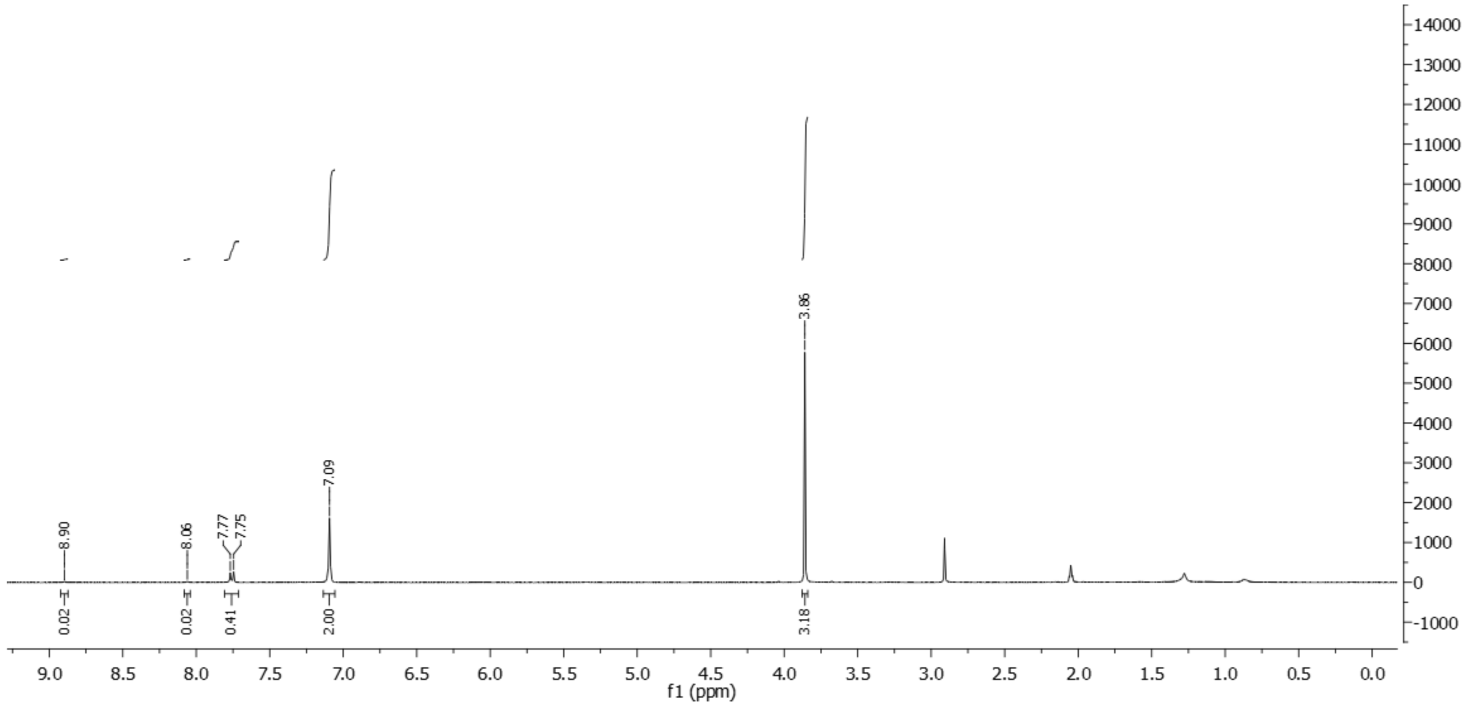

^{1}H-NMR spectrum of **10**

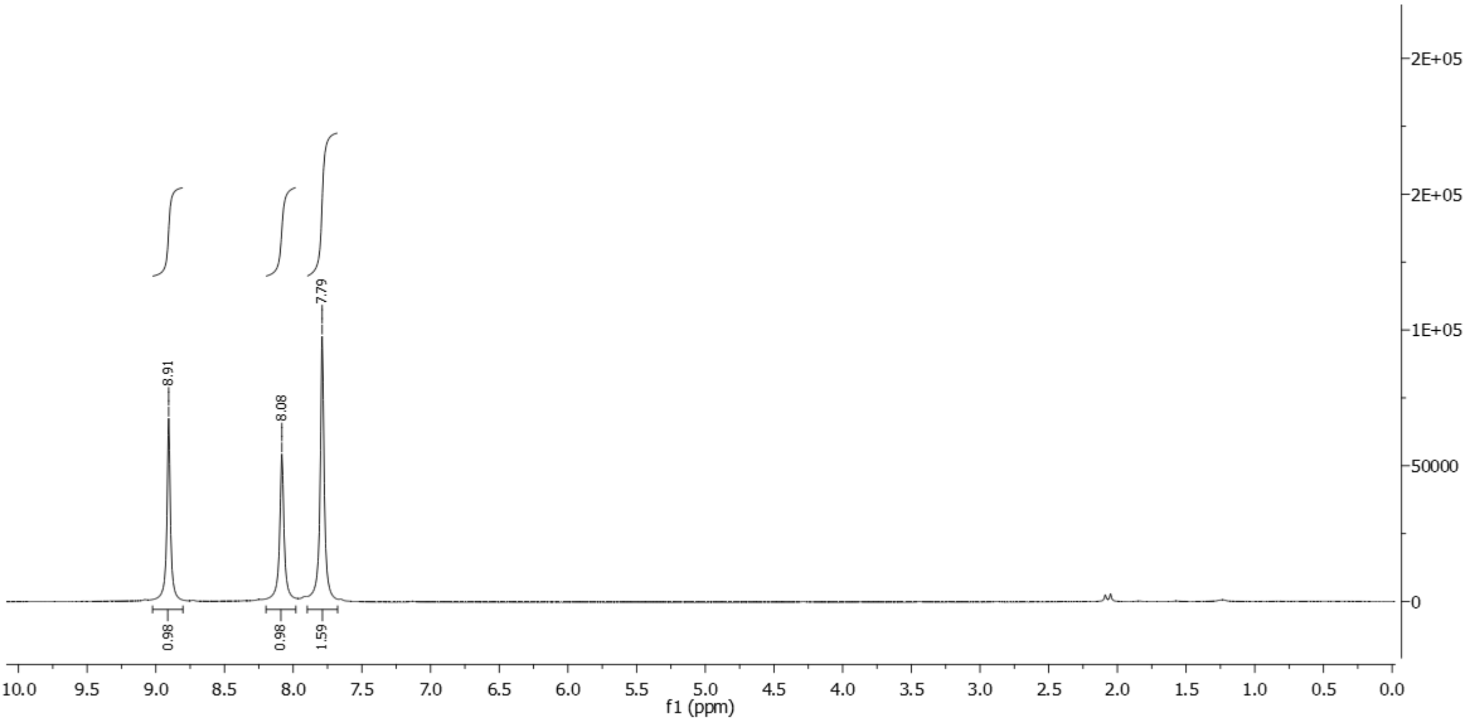

^{2}H-NMR spectrum of **10**

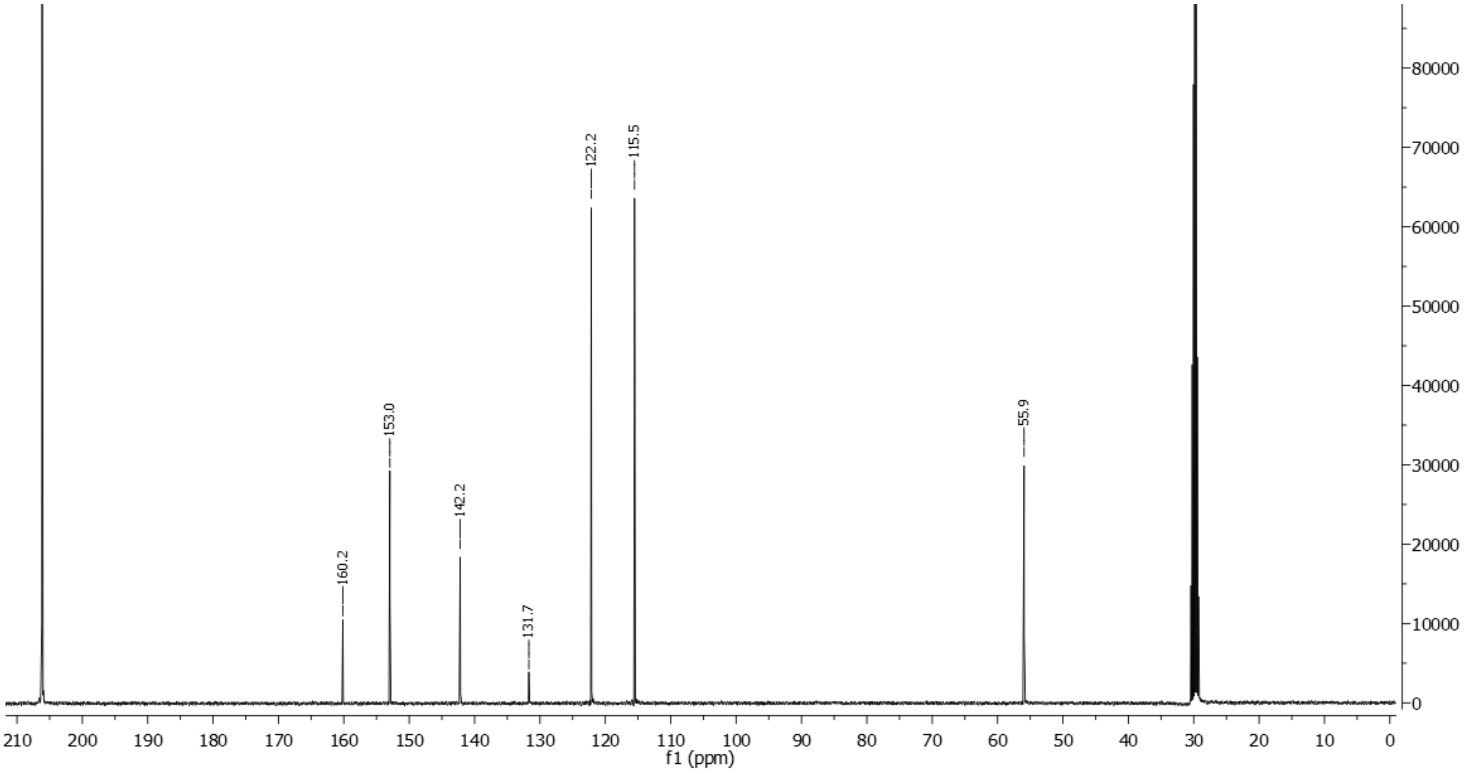

^{13}C-NMR spectrum of the non-deuterated starting material

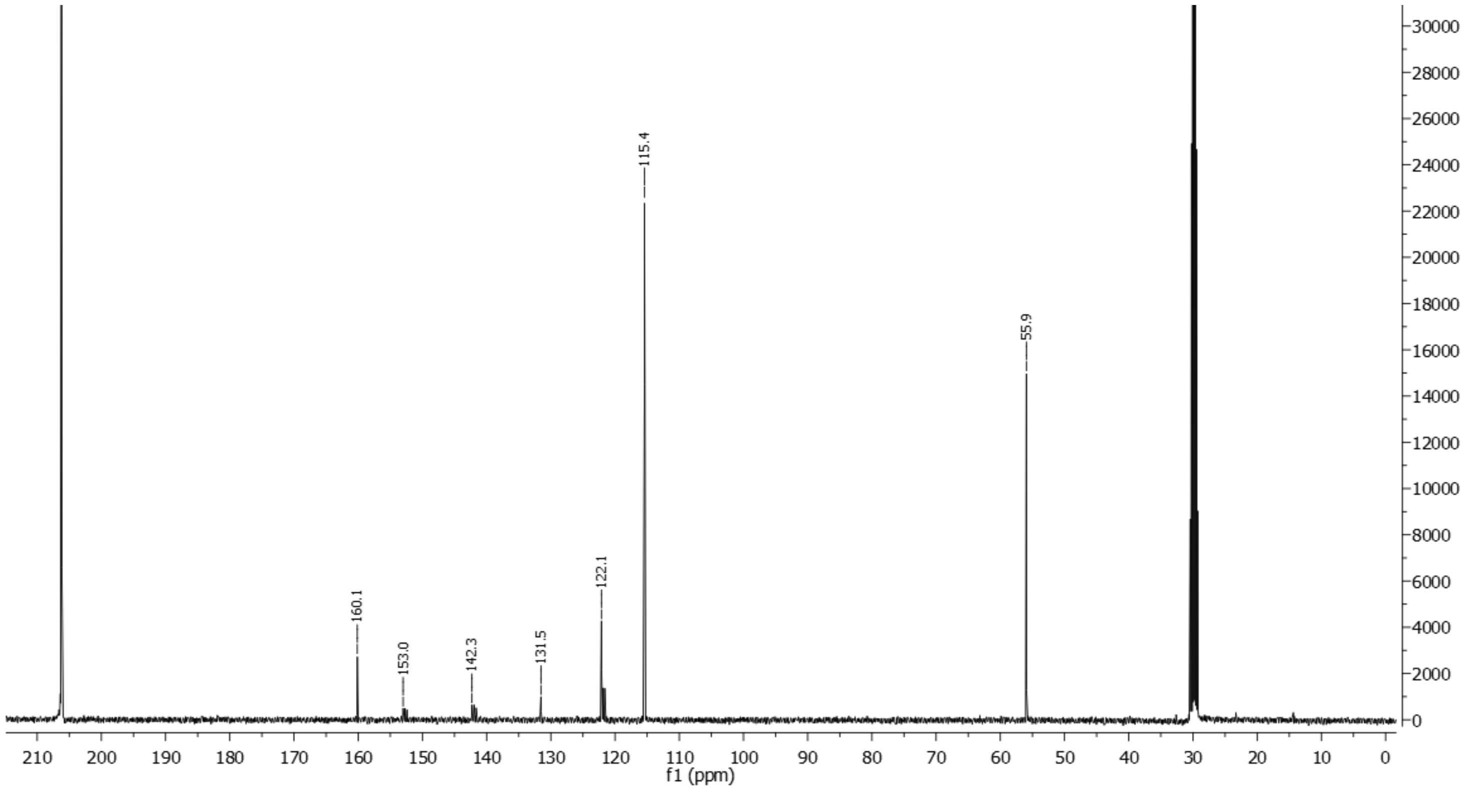

^{13}C-NMR spectrum of **10**

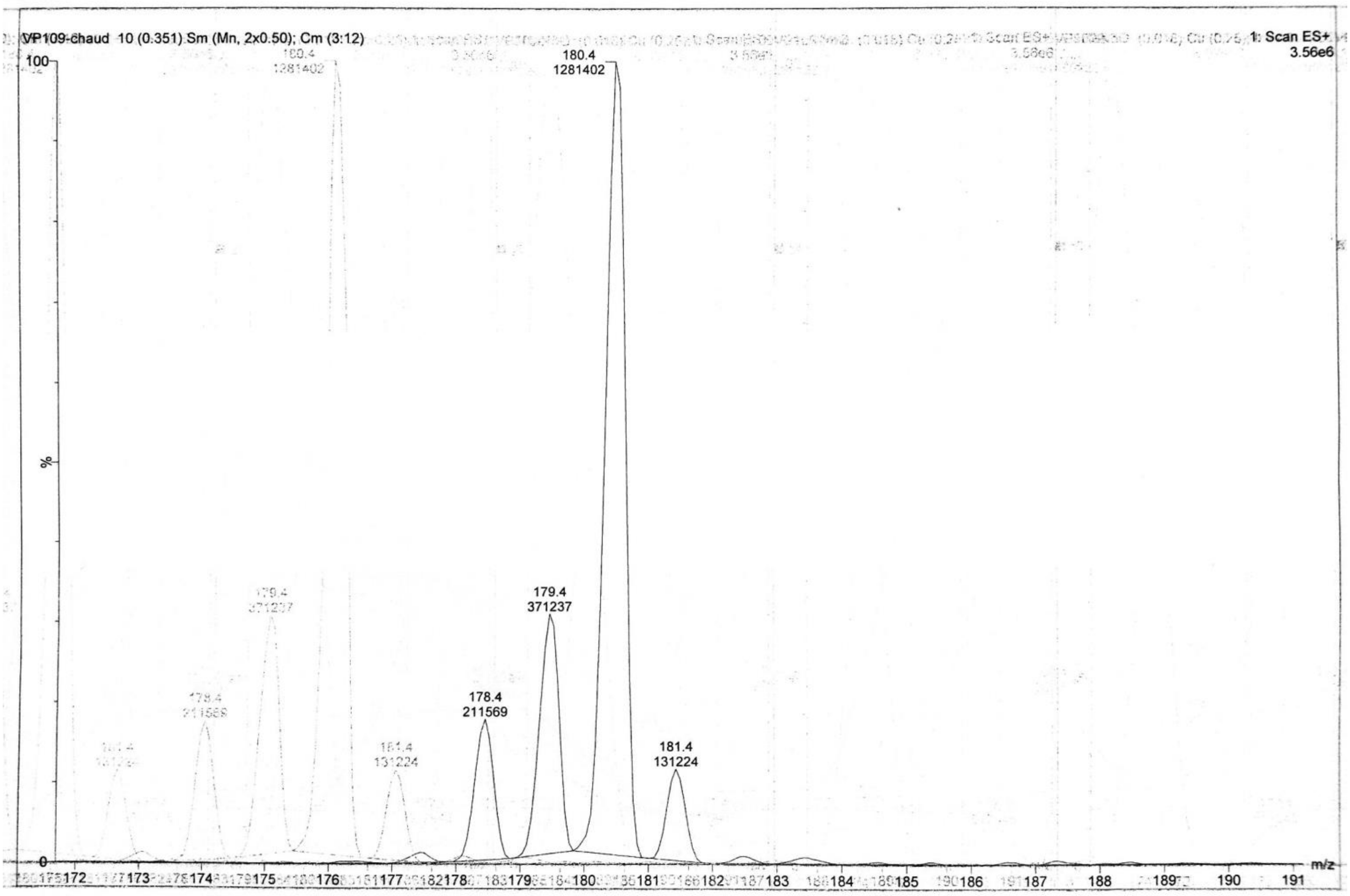

ESI spectrum of **10**

4-(1*H*-1,2,4-Triazol-1-yl)aniline **11**

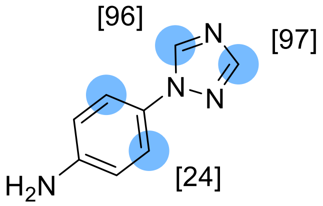

Chemical Formula: $C_8H_8N_4$

Substrate	Solvent (Volume)	RuNp@PVP cat.
32.0mg, 0.2mmol	DMA (2mL)	14.4mg, 5mol%

Workup and purification:

After cooling down to room temperature, EtOAc : Cy (1:1, 3mL) was added to the reaction mixture and stirred for 10min to let precipitate RuNp@PVP. The suspension was passed through a Sep-Pak® C18 cartridge and then eluted with EtOAc : Cy (1:1, 5mL). The solvent was removed under vacuum.

Yield: 33.0mg, 99%, grey solid

^{1}H NMR (400 MHz, CDCl$_3$): δ 8.38 (s, 0.03H), 8.04 (s, 0.04H), 7.45 – 7.34 (m, 1.53H), 6.80 – 6.69 (m, 2H), 3.87 (bs, NH$_2$).

Deuterium incorporation was expected at *δ* 8.38, *δ* 8.04 and at *δ* 7.45 – 7.34. Isotopic enrichment values were determined against the integral at *δ* 6.80 – 6.69.

^{2}H-{^{1}H}NMR (92 MHz, CHCl$_3$): δ 8.42 (s, 0.97D), 8.07 (s, 0.96D), 7.43 (s, 0.48D).

^{13}C-{^{1}H}NMR (100 MHz, CDCl$_3$): δ 152.2 (m), 146.8, 140.8 (m), 128.7, 122.1 (m), 115.5.

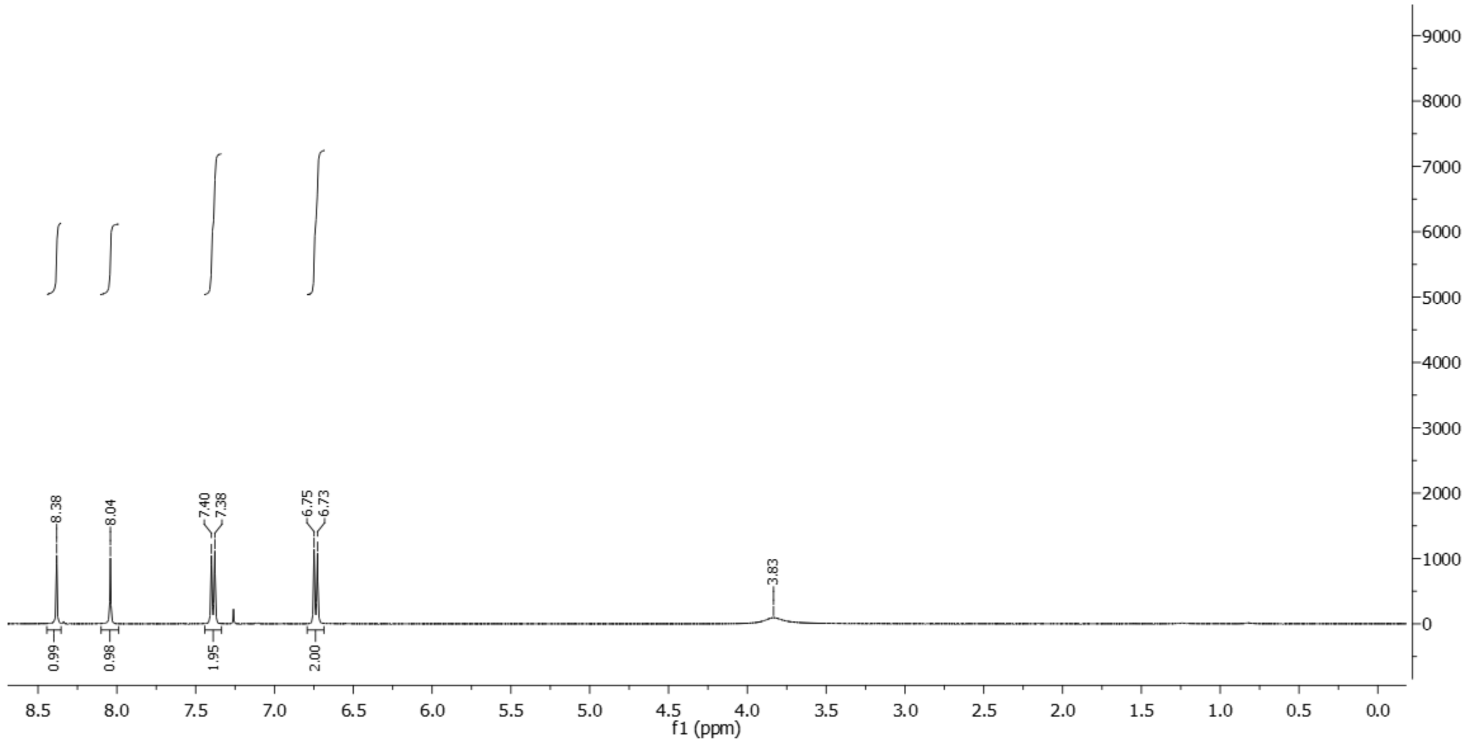

^{1}H-NMR spectrum of the non-deuterated starting material

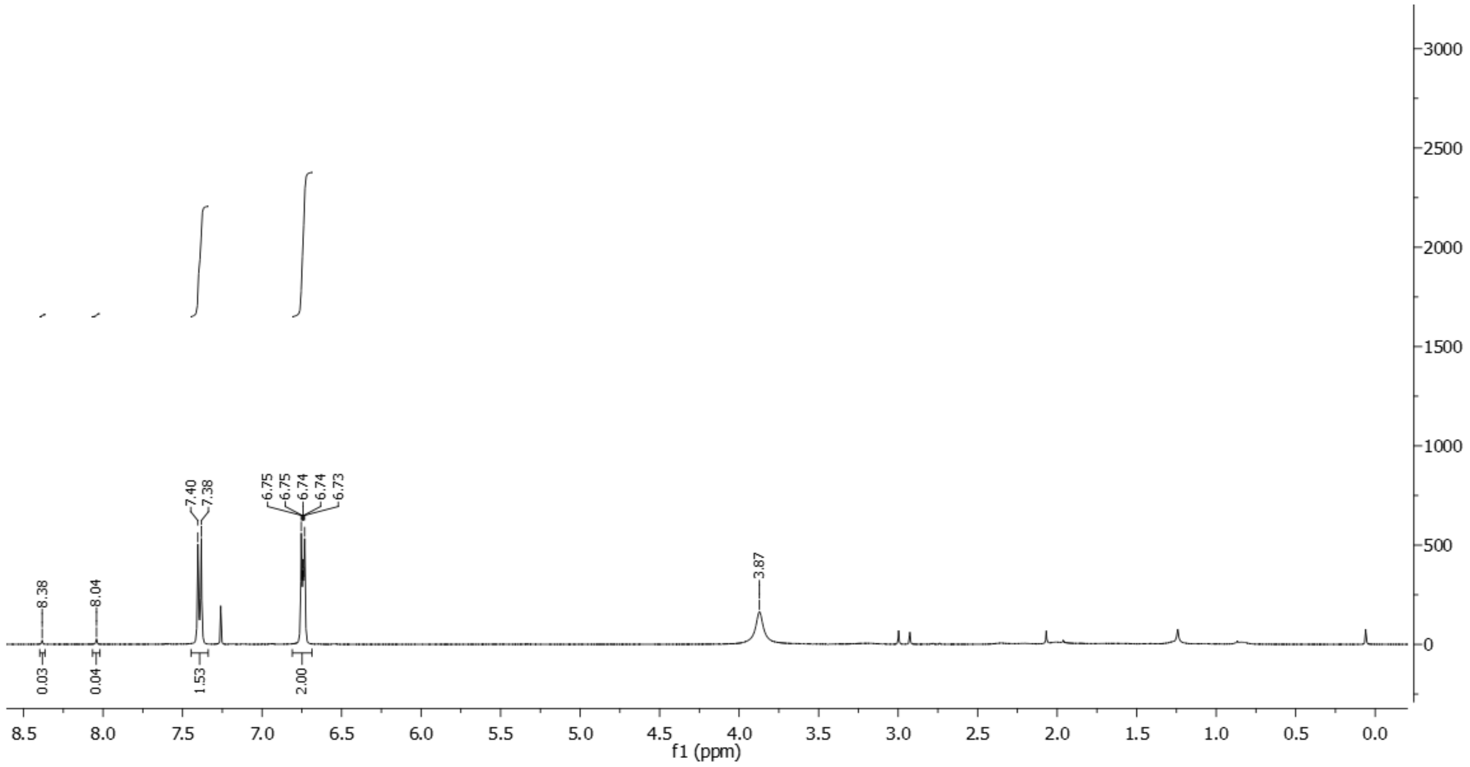

^{1}H-NMR spectrum of **11**

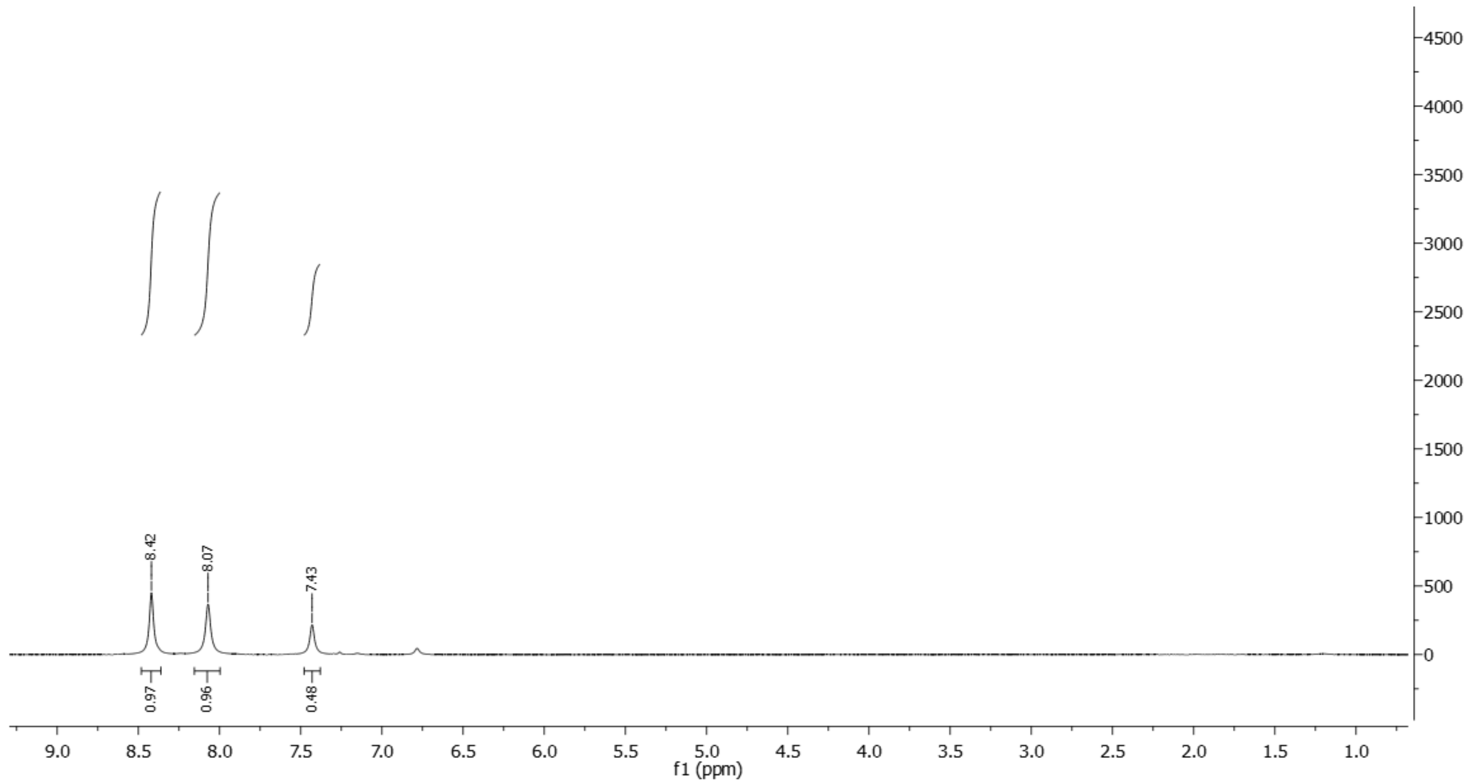

^{2}H-NMR spectrum of **11**

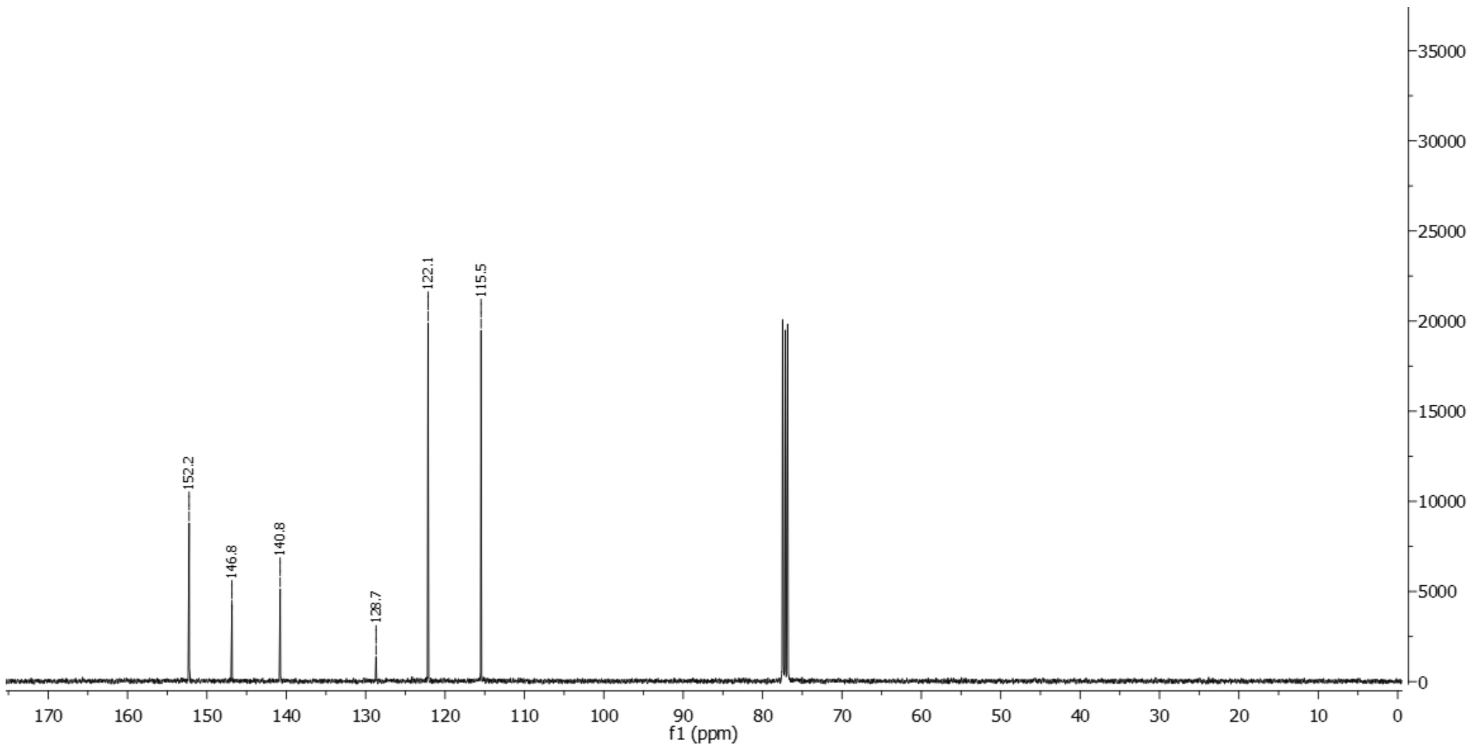

^{13}C-NMR spectrum of the non-deuterated starting material

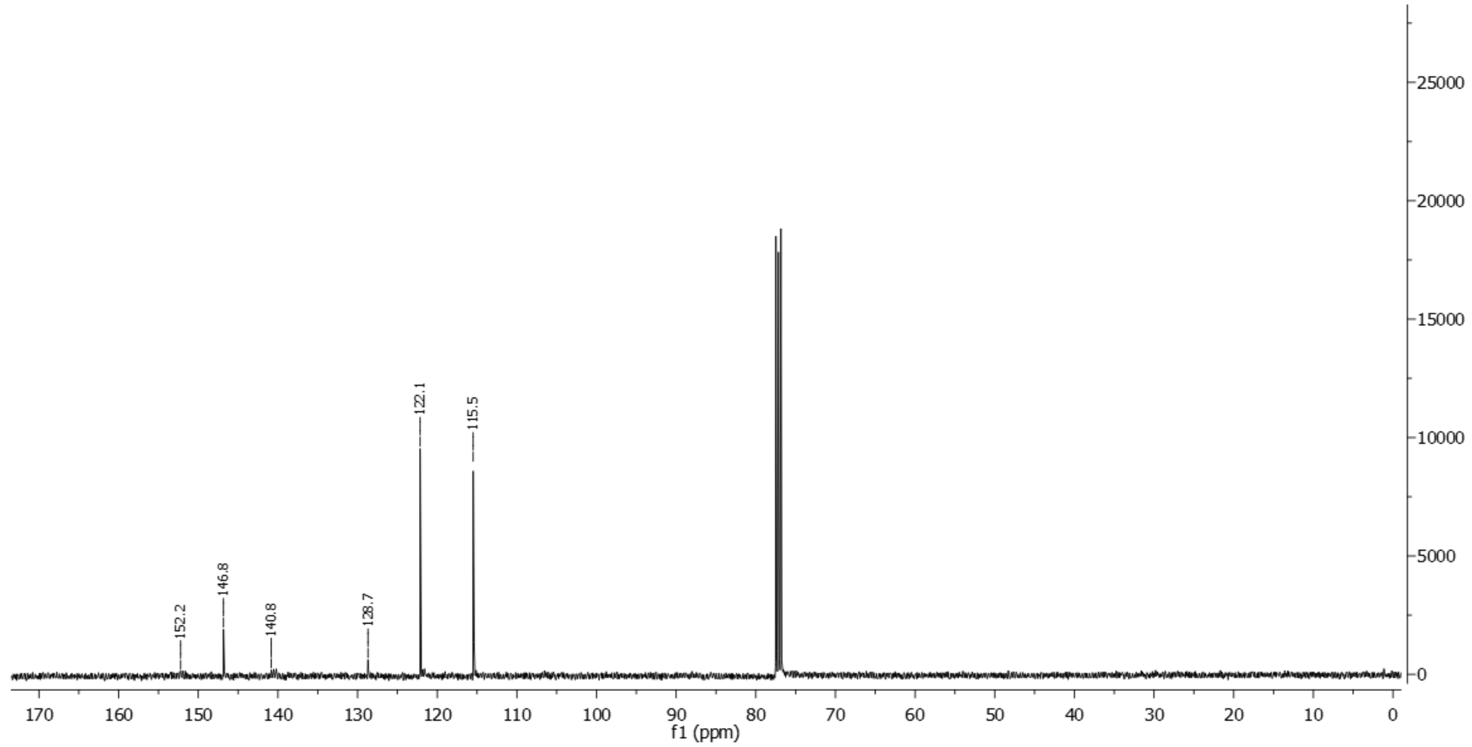

^{13}C-NMR spectrum of **11**

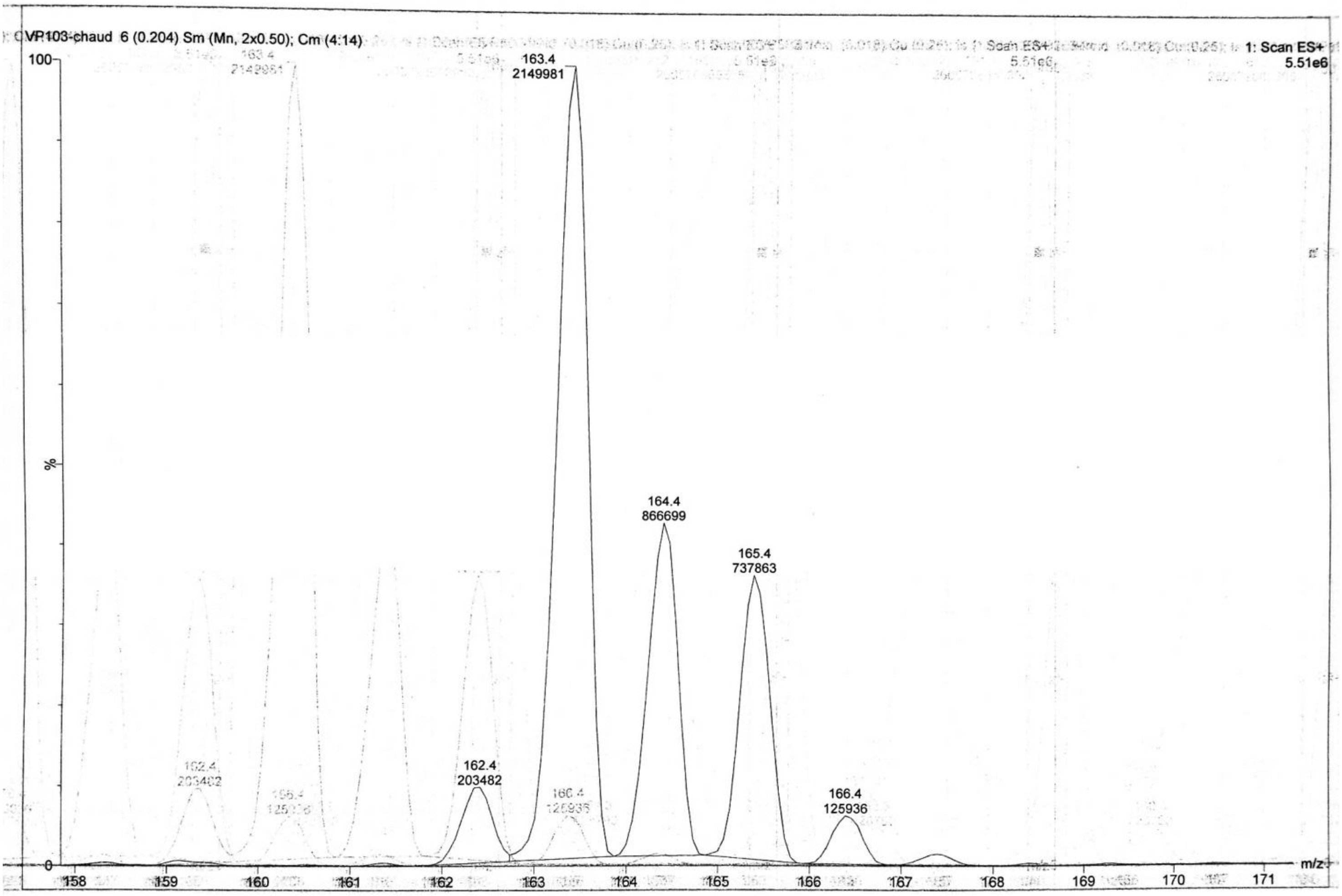

ESI-spectrum of **11**

4'-(1,2,4-Triazol-1-yl)acetanilide **12**

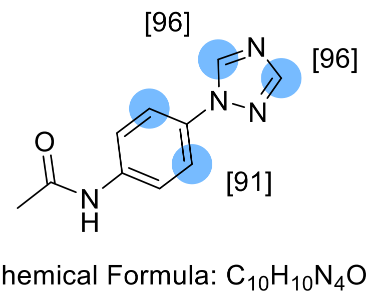

Substrate	Solvent (Volume)	RuNp@PVP cat.
40.4mg, 0.2mmol	DMA (2mL)	14.4mg, 5mol%

Workup and purification:

After cooling down to room temperature, EtOAc : Cy (1:1, 3mL) was added to the reaction mixture and stirred for 10min to let precipitate RuNp@PVP. The suspension was passed through a Sep-Pak® C18 cartridge and then eluted with ethylacetate (5mL). The solvent was removed under vacuum and the crude product was recrystallized from DCM/MeOH (10:1).

Yield: 26.0mg, 64%, white solid

^{1}H NMR (400 MHz, Methanol-*d$_4$*): δ 9.03 (s, 0.04H), 8.14 (s, 0.04H), 7.78 – 7.71 (m, 2.18H), 2.15 (s, 3H).

Deuterium incorporation was expected at δ 9.03, δ 8.14 and at δ 7.78 – 7.71. Isotopic enrichment values were determined against the integral at δ 2.15.

^{2}H-{^{1}H}NMR (92 MHz, Methanol): δ 8.98 (s, 0.95D), 8.12 (s, 0.96D), 7.73 (s, 1.81D).

^{13}C-{^{1}H}NMR (100 MHz, Methanol-*d$_4$*): δ 171.8, 152.8, 142.9 (m), 140.2 (m), 133.9, 121.8 (m), 121.7, 23.9.

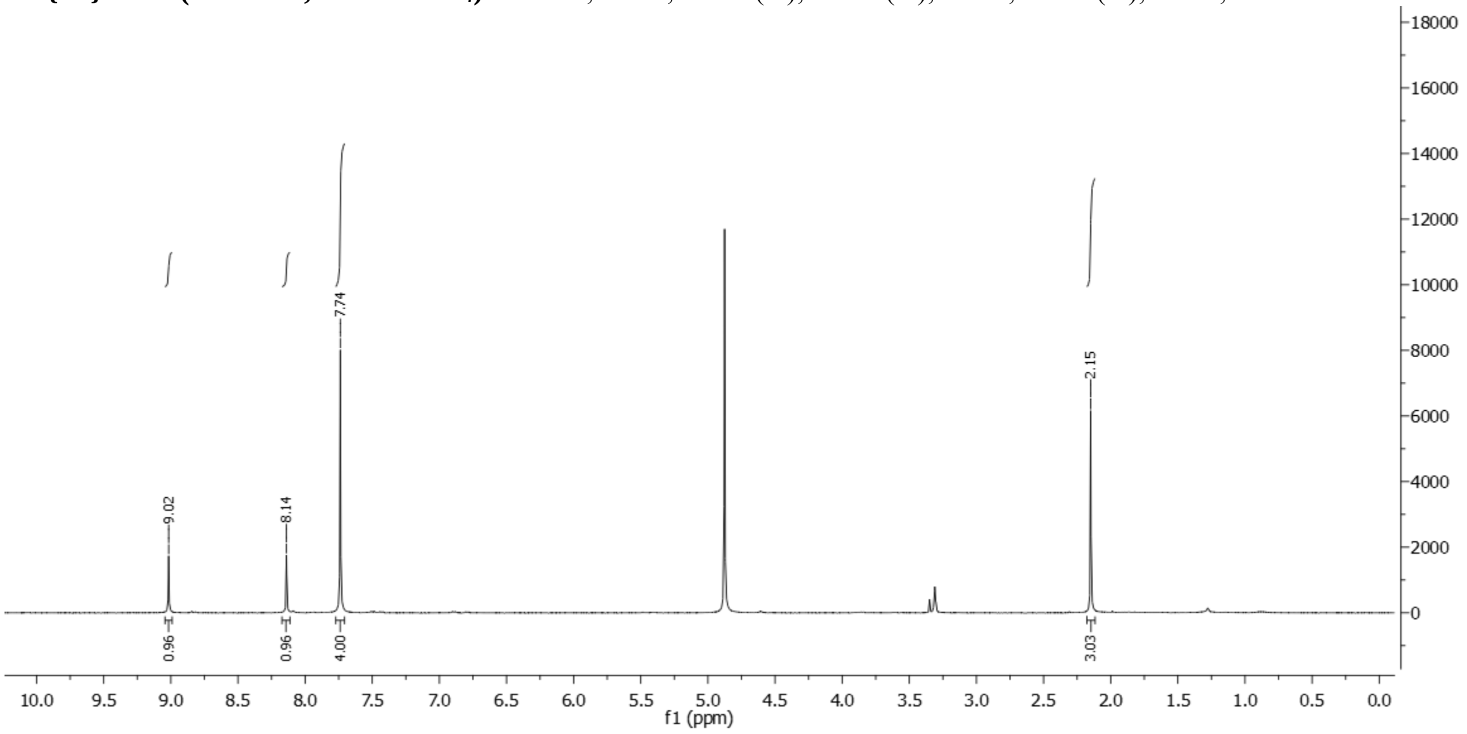

^{1}H-NMR spectrum of the non-deuterated starting material

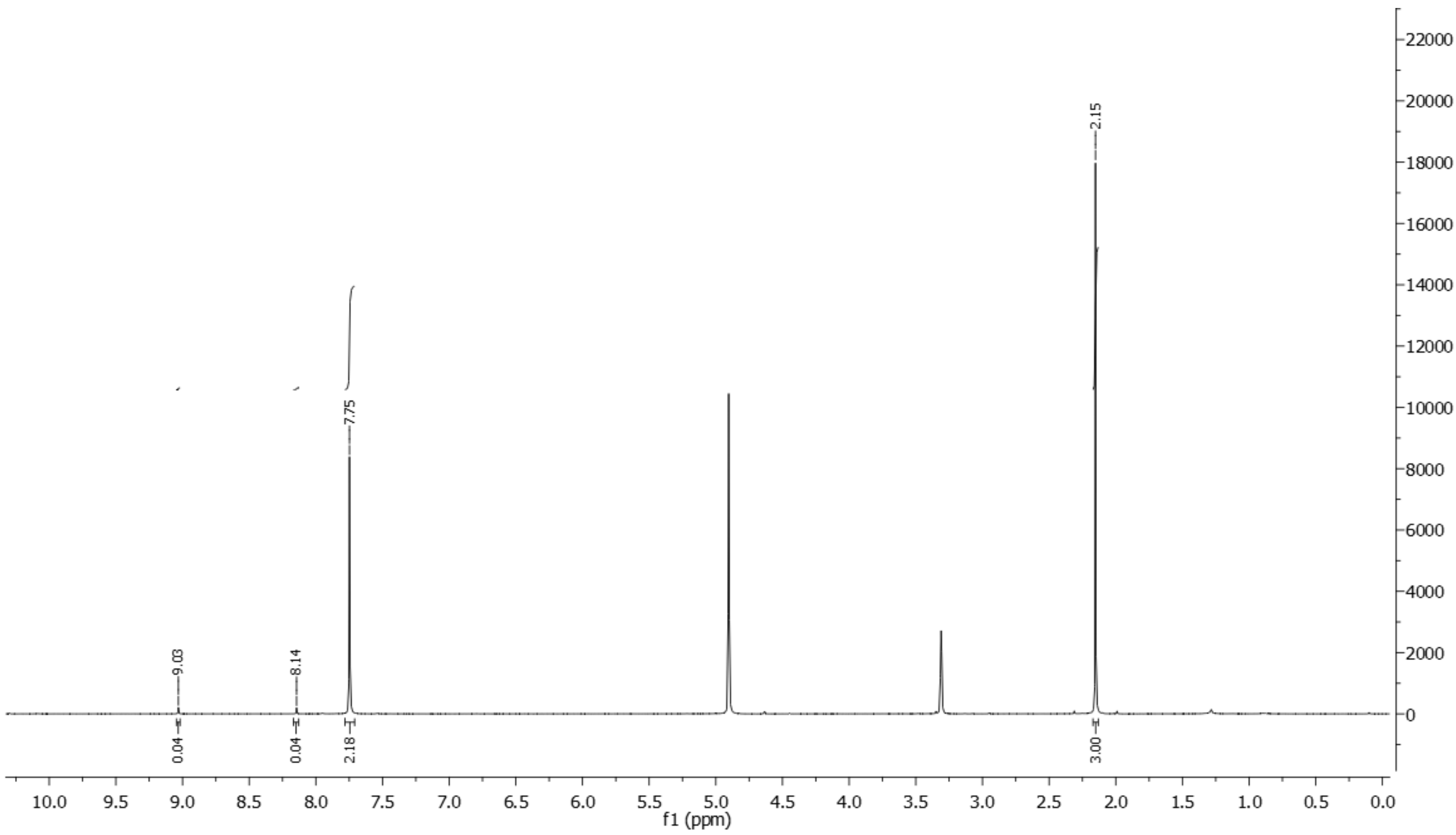

^{1}H-NMR spectrum of **12**

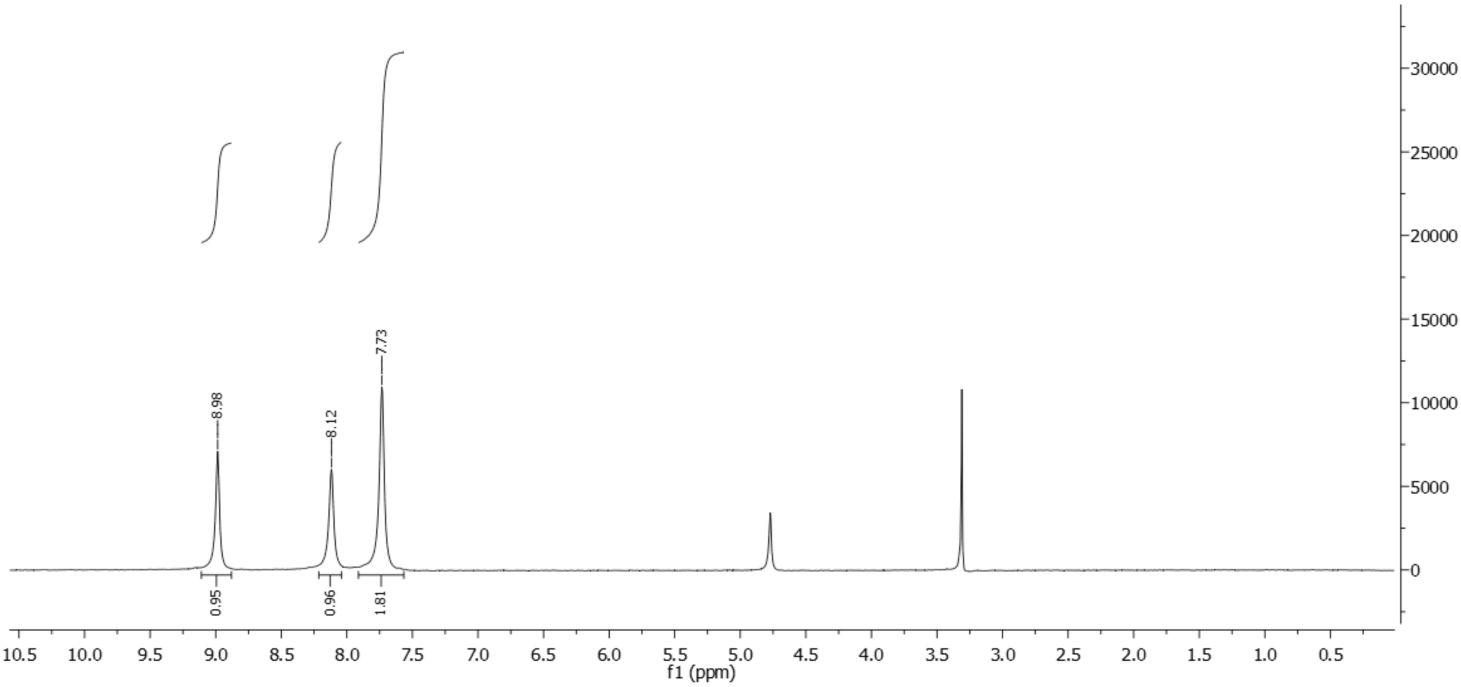

^{2}H-NMR spectrum of **12**

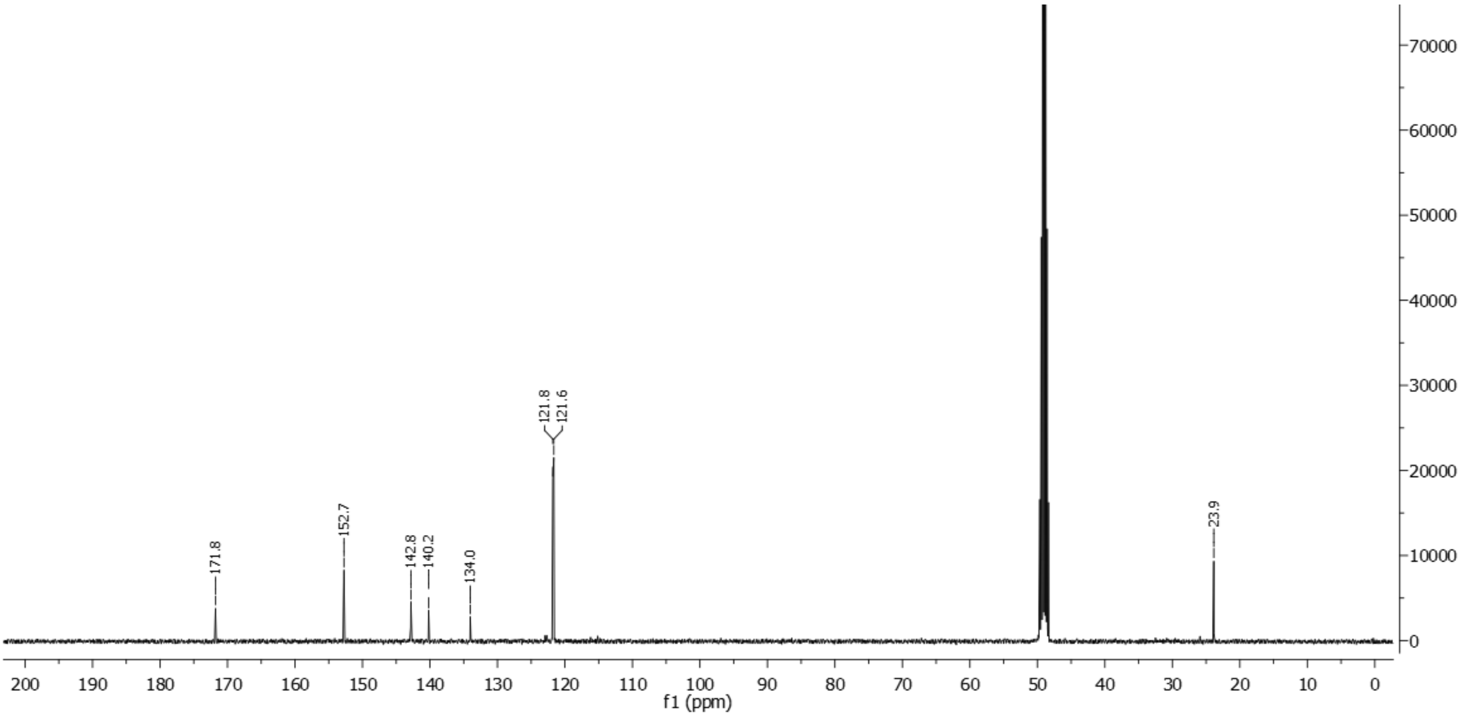

^{13}C-NMR spectrum of the non-deuterated starting material

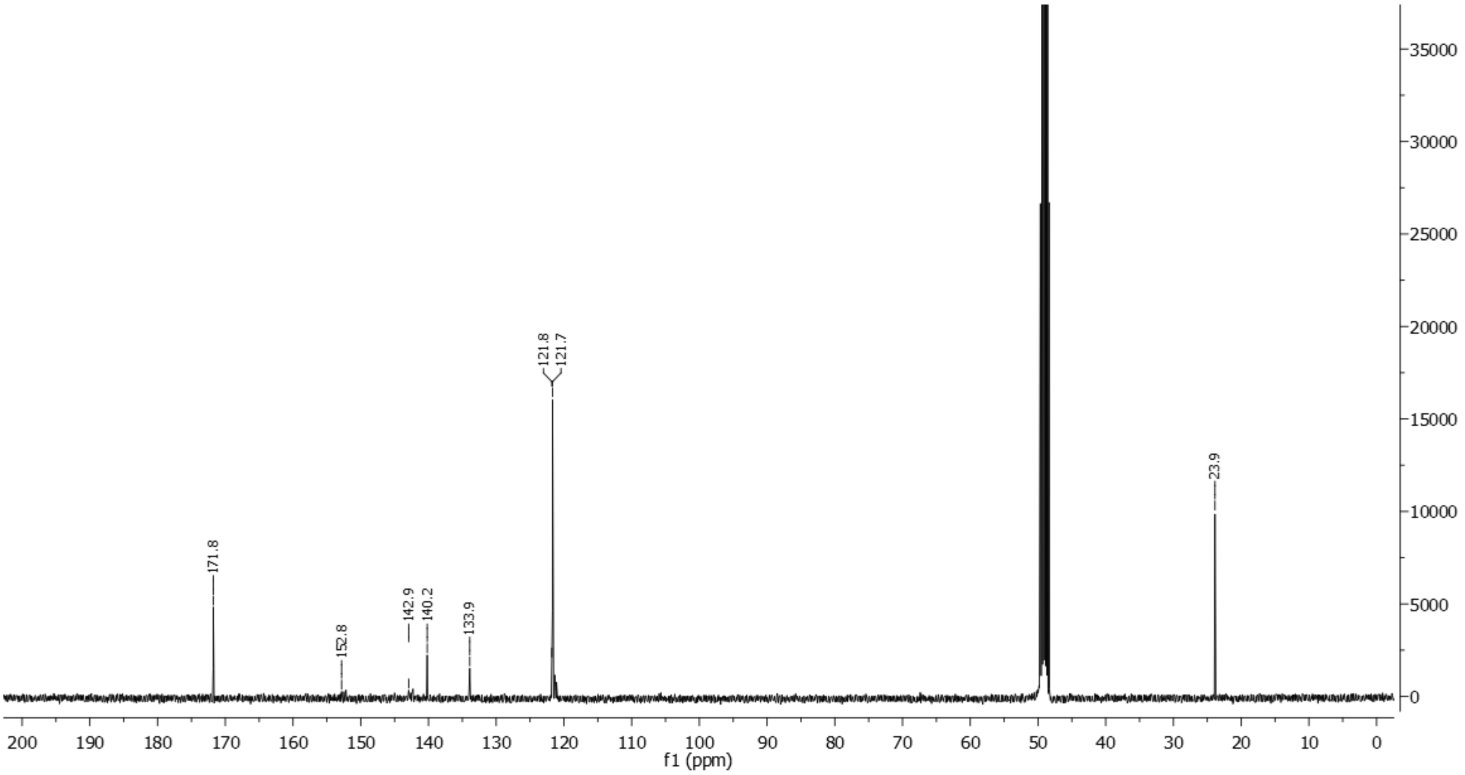

^{13}C-NMR spectrum of **12**

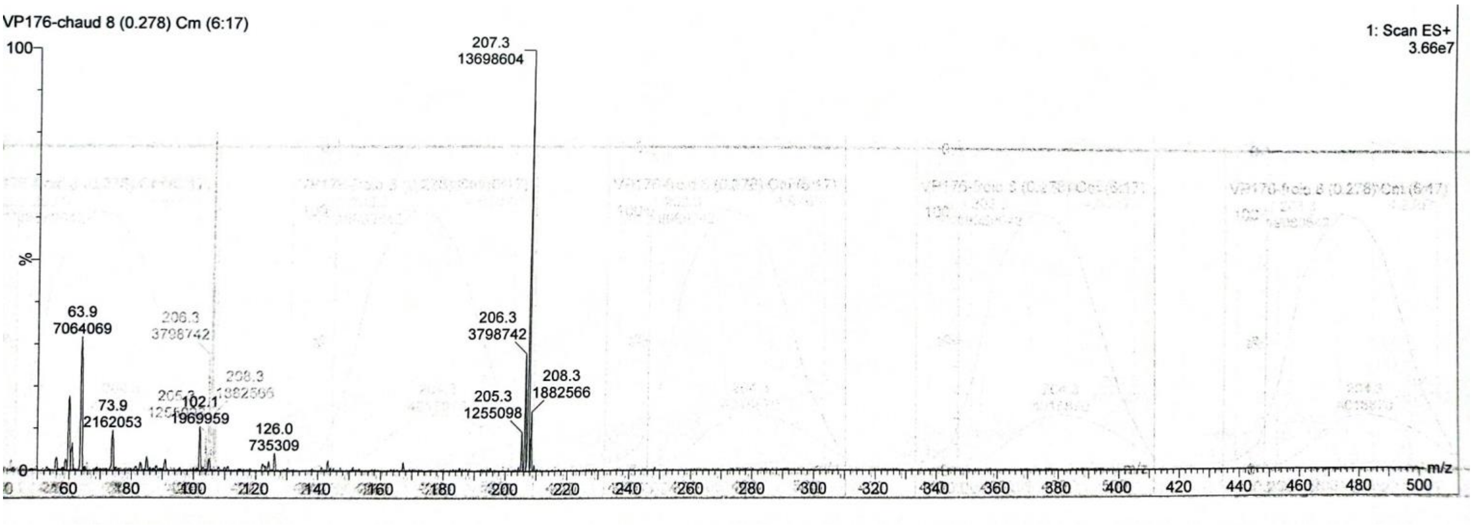

ESI-spectrum of **12**

Deuteration of carbazoles with RuNp@PVP and Cs_2CO_3

Carbazole **13**

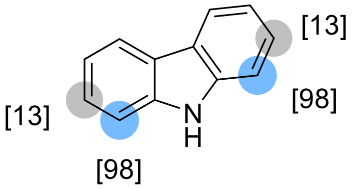

Substrate	Cs_2CO_3	Solvent (Volume)	RuNp@PVP cat.
33.4mg, 0.2mmol	65.2mg, 0.2mmol	THF (2mL)	14.4mg, 5mol%

Workup and purification:

After cooling down to room temperature the reaction mixture was poured on a 5mM solution of acetic acid in H_2O dist. (100mL). The aqueous phase was extracted three times with EtOAc (3 x 50mL) in a separation funnel. The solvent was removed under vacuum and the crude product was recrystallized from THF and MeOH (THF : MeOH, 10:1).

Yield: 34.0mg, 99%, white solid

^{1}H NMR (400 MHz, Acetone-*d*$_6$): δ 10.37 (bs, NH), 8.16 – 8.08 (m, 2H), 7.54 – 7.50 (m, 0.04H), 7.42 – 7.36 (m, 2H), 7.22 – 7.15 (m, 2H).

Deuterium incorporation was expected at δ 7.54 – 7.50. Isotopic enrichment values were determined against the integral at δ 8.16 – 8.08.

^{2}H-{^{1}H}NMR (92 MHz, Acetone): δ 7.52 (m, 1.96D), 7.39 (m, 0.27D).

^{13}C-{^{1}H}NMR (100 MHz, Acetone-*d*$_6$): δ 140.9, 126.3, 123.90, 120.8, 119.6, 111.7 (m).

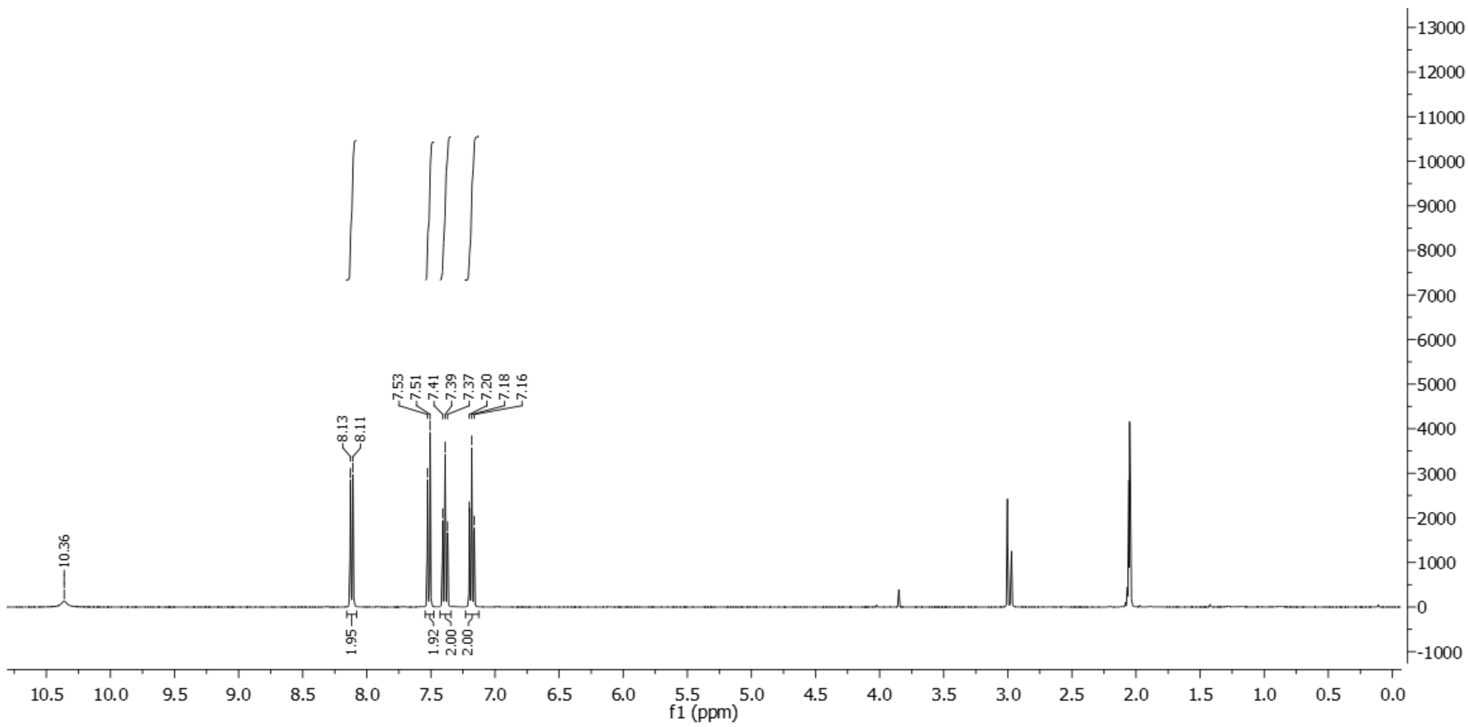

^{1}H-NMR spectrum of the non-deuterated starting material

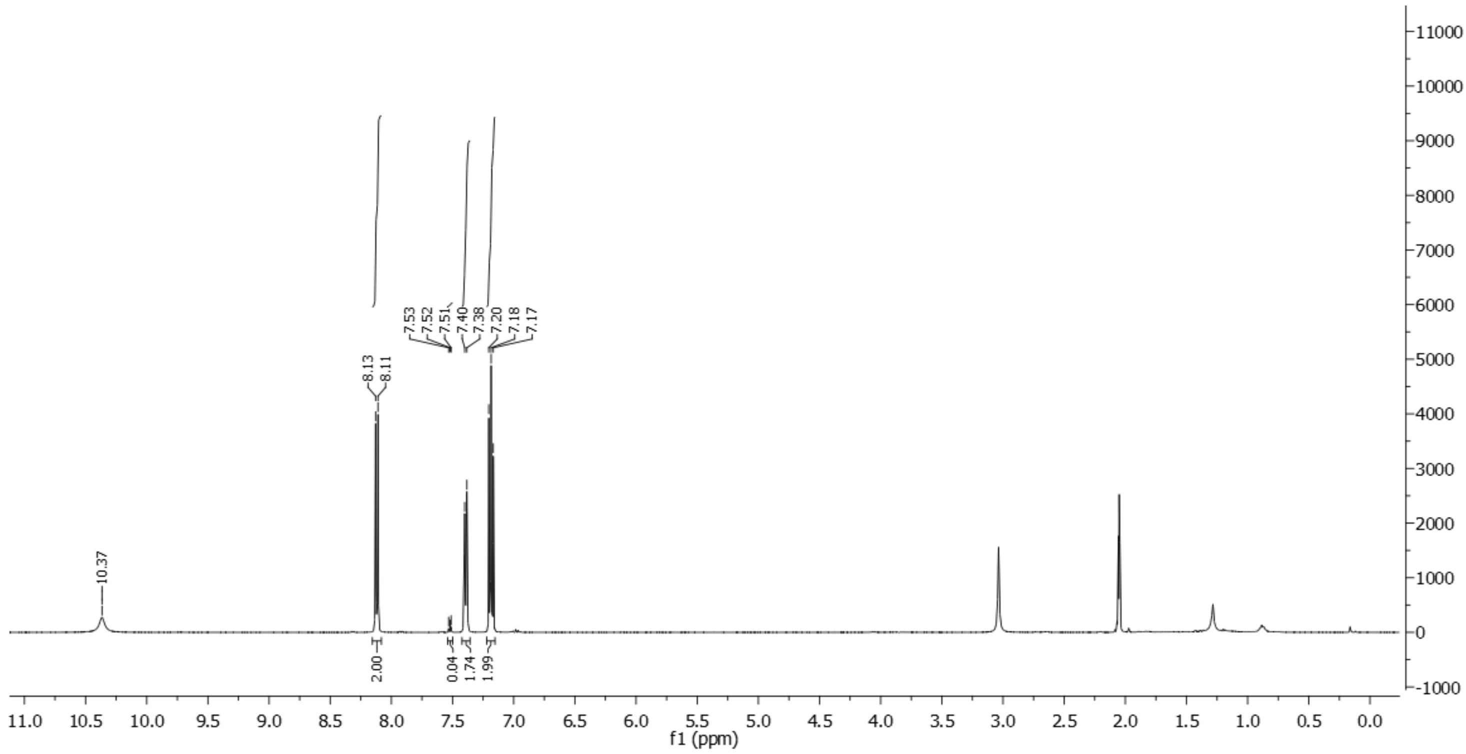

^{1}H-NMR spectrum of **13**

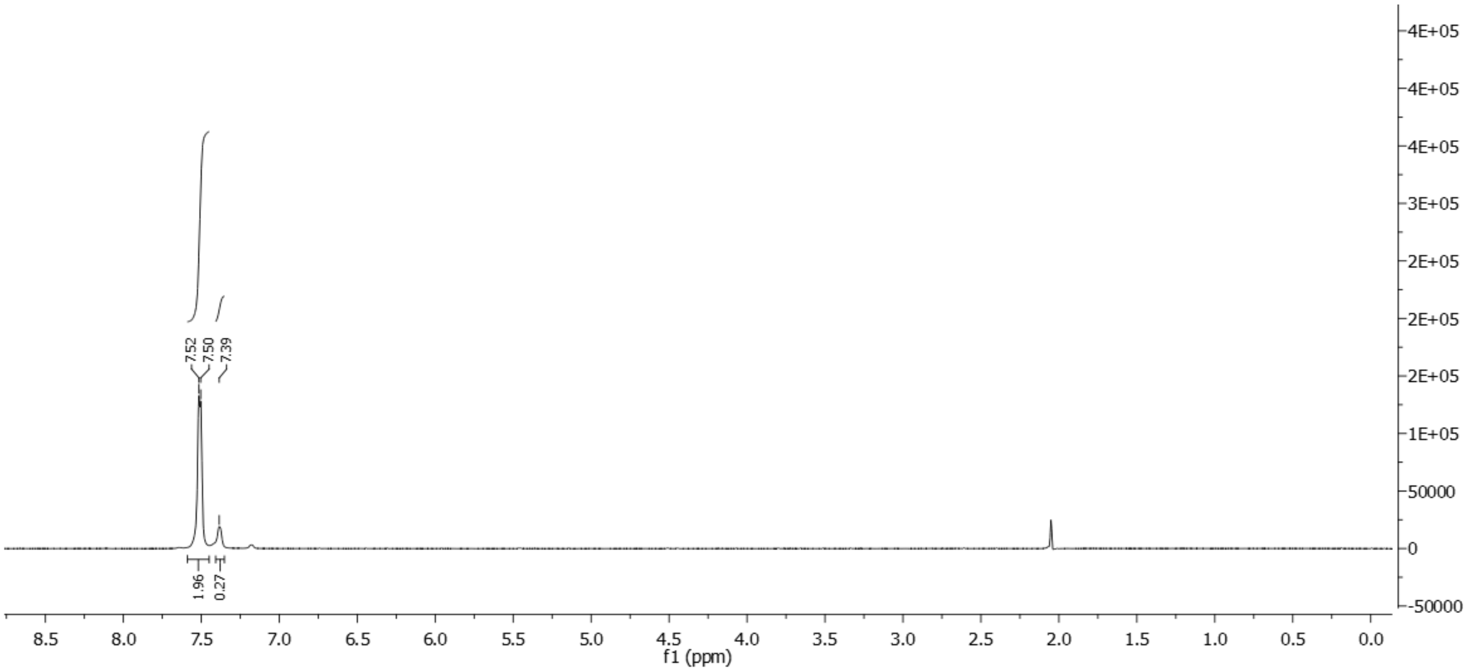

^{2}H-NMR spectrum of **13**

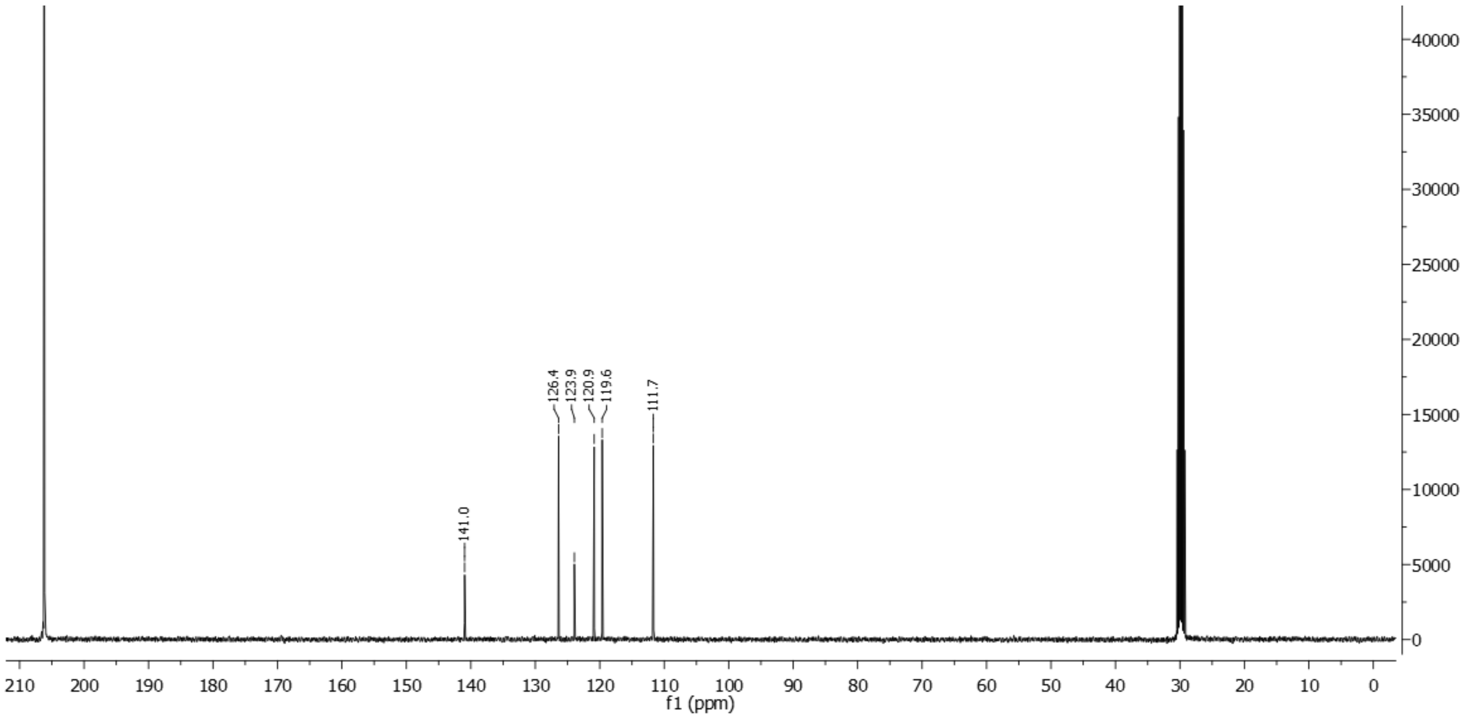

^{13}C-NMR spectrum of the non-deuterated starting material

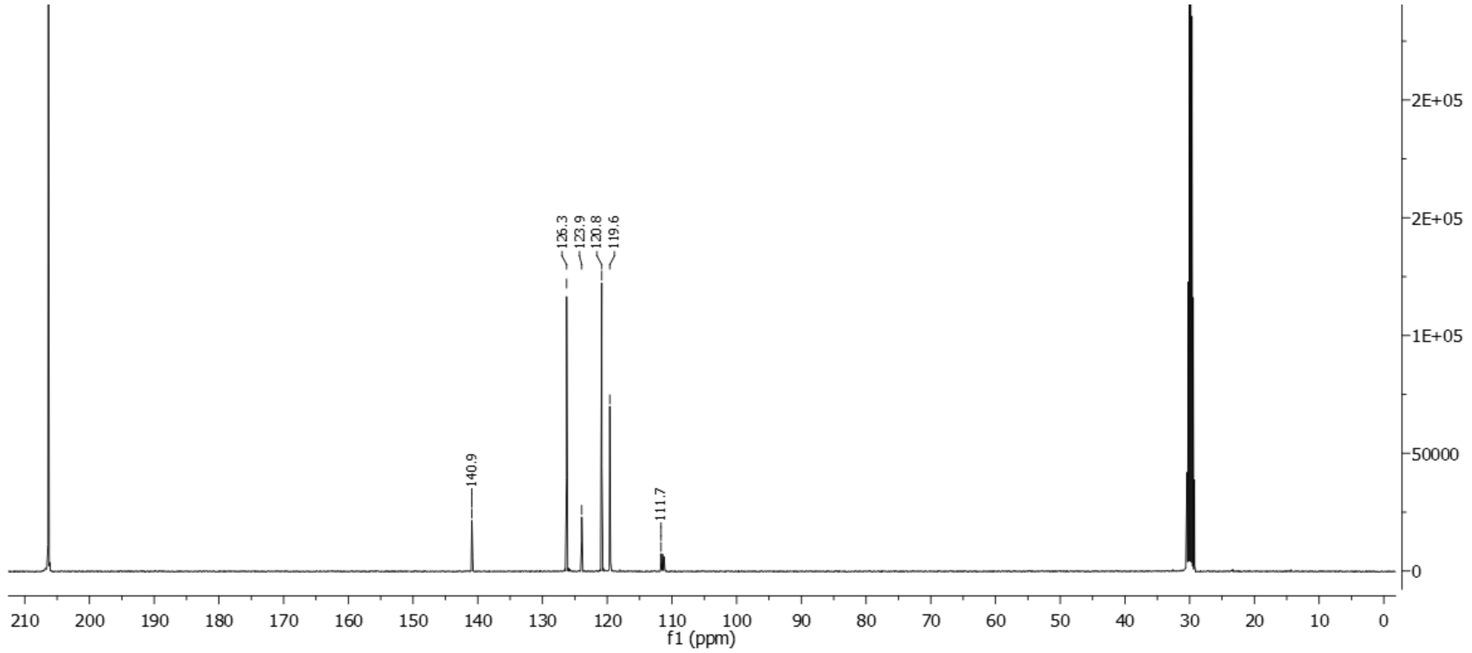

^{13}C-NMR spectrum of **13**

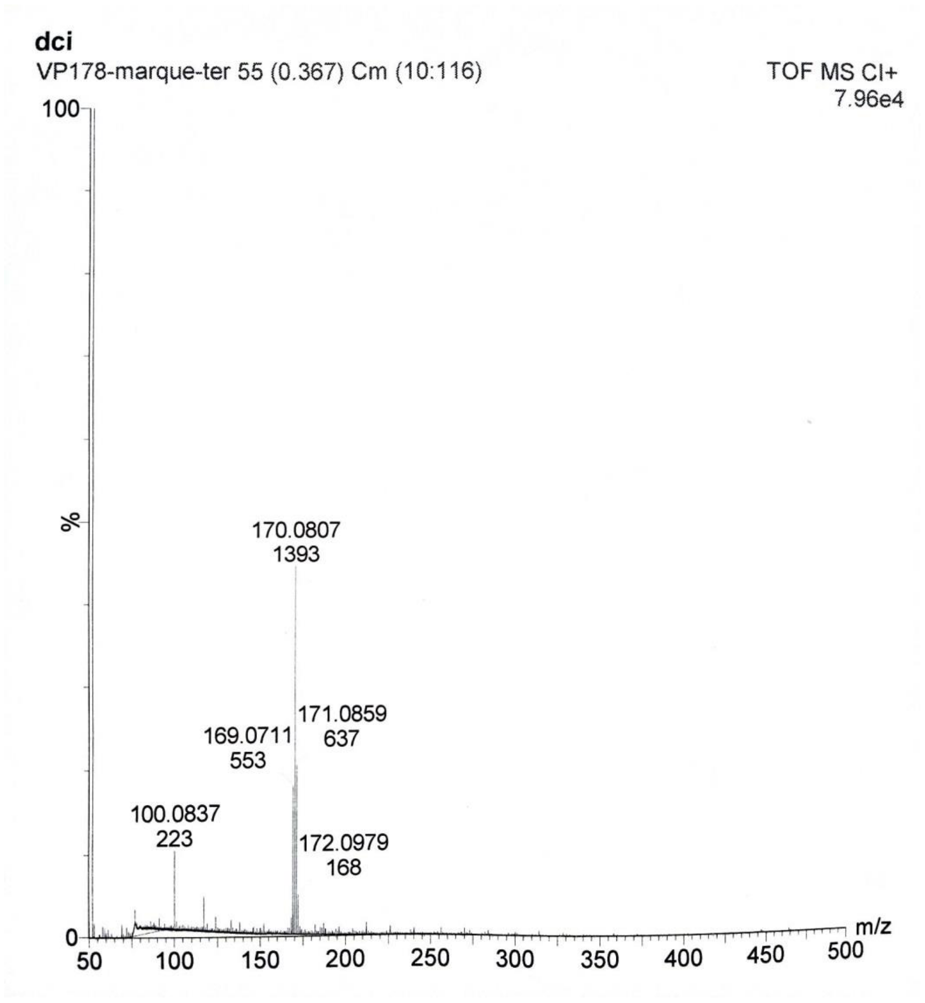

TOF-spectrum of **13** after GC-MS analysis

3,6-Di-*tert*-butylcarbazole **14**

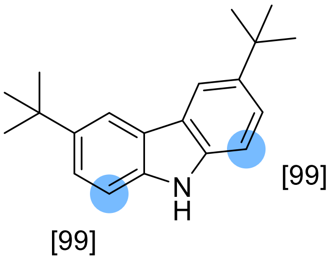

Chemical Formula: $C_{20}H_{25}N$

Substrate	Cs_2CO_3	Solvent (Volume)	RuNp@PVP cat.
22.9mg, 0.1mmol	32.6mg, 0.1mmol	THF (2mL)	7.22mg, 5mol%

Workup and purification:

After cooling down to room temperature the reaction mixture was poured on a 5mM solution of acetic acid in H_2O dist. (100mL). The aqueous phase was extracted three times with EtOAc (3 x 50mL) in a separation funnel. The solvent was removed under vacuum.

Yield: 23mg, 99%, white solid

^{1}H NMR (400 MHz, Acetone-*d$_6$*): δ 10.04 (bs, NH), 8.23 – 8.15 (m, 2H), 7.51 – 7.42 (m, 2H), 7.41 – 7.36 (m, 0.03H), 1.43 (s, 18H).

Deuterium incorporation was expected at *δ* 7.41 – 7.36. Isotopic enrichment values were determined against the integral at *δ* 8.23 – 8.15.

^{2}H-{^{1}H}NMR (92 MHz, Acetone): δ 7.40 (s).

^{13}C-{^{1}H}NMR (100 MHz, Acetone-*d$_6$*): δ 142.1, 139.5, 139.3, 123.9, 116.9, 111.1 (m), 35.2, 32.4.

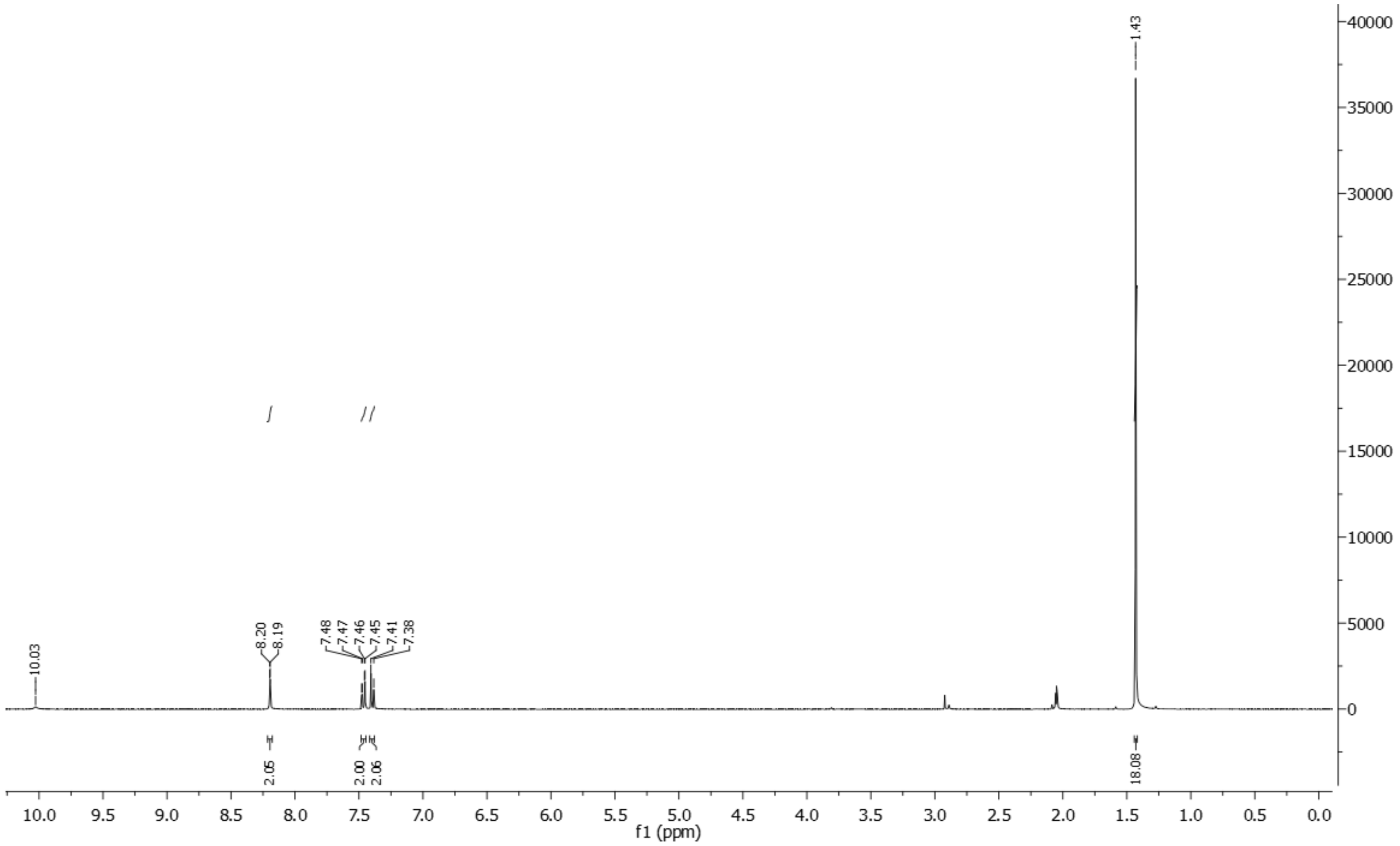

^{1}H-NMR spectrum of the non-deuterated starting material

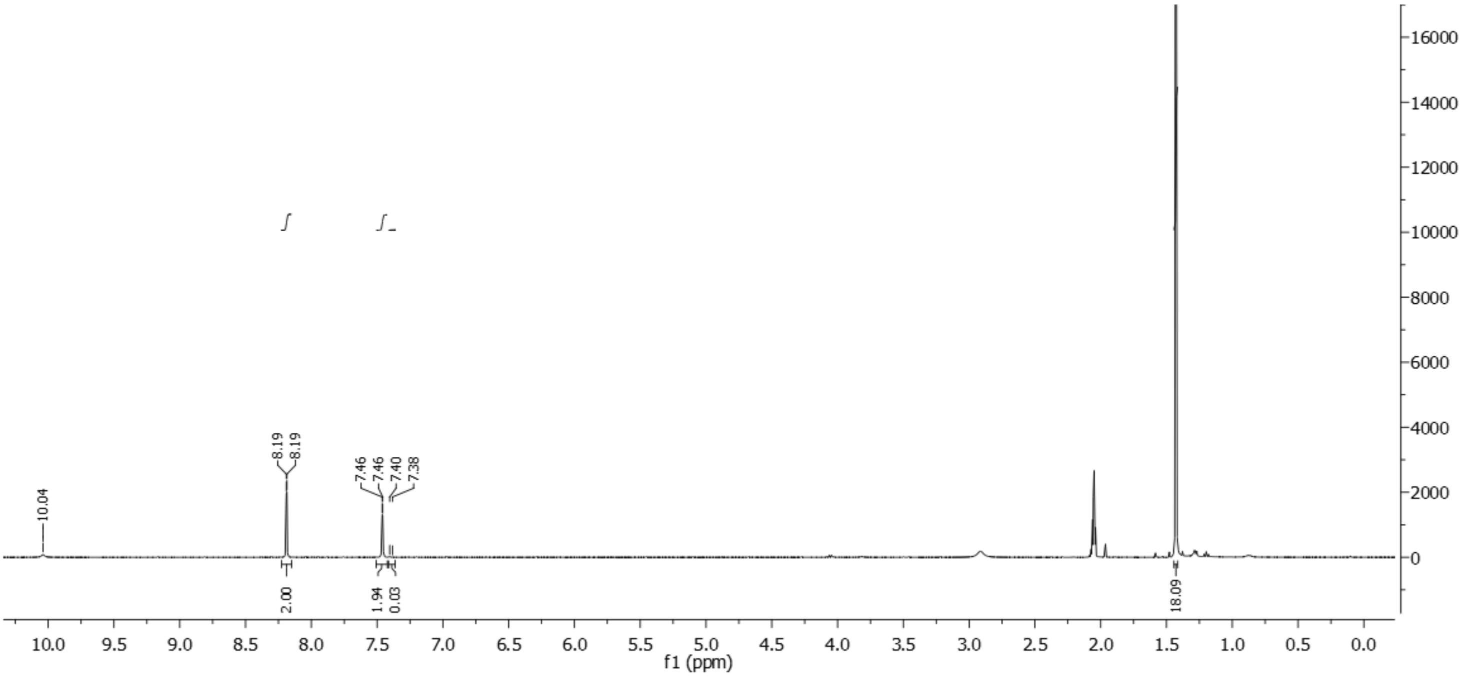

^{1}H-NMR spectrum of **14**

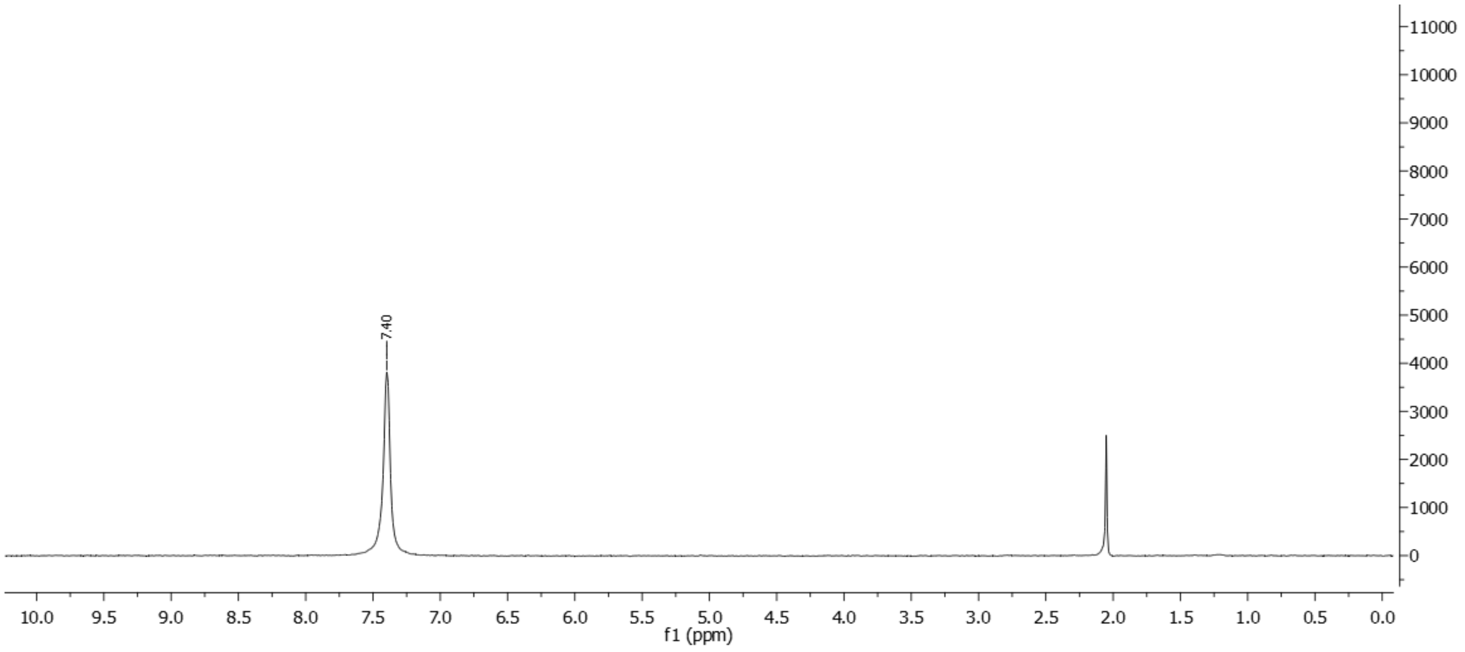

^{2}H-NMR spectrum of **14**

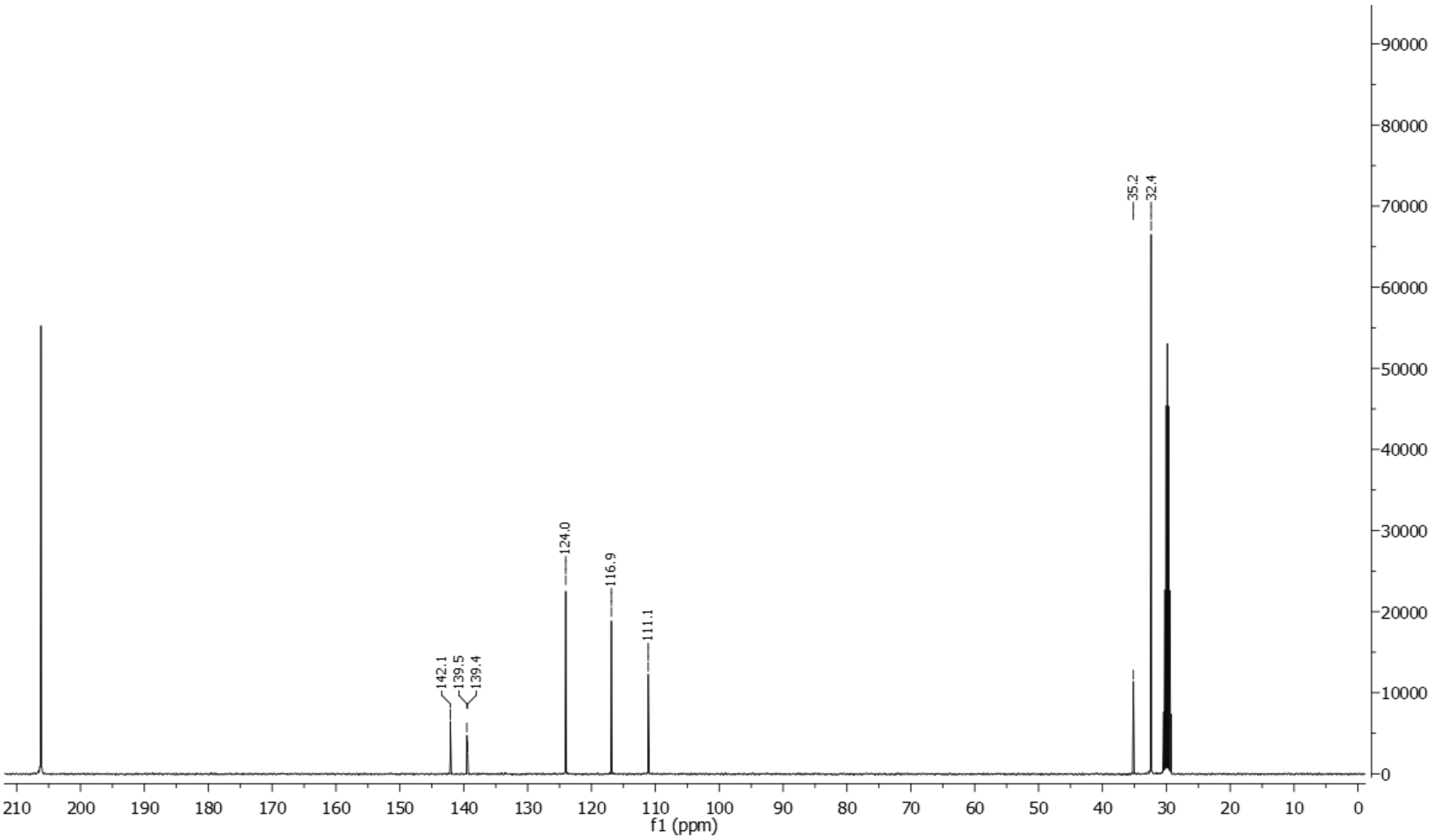

^{13}C-NMR spectrum of the non-deuterated starting material

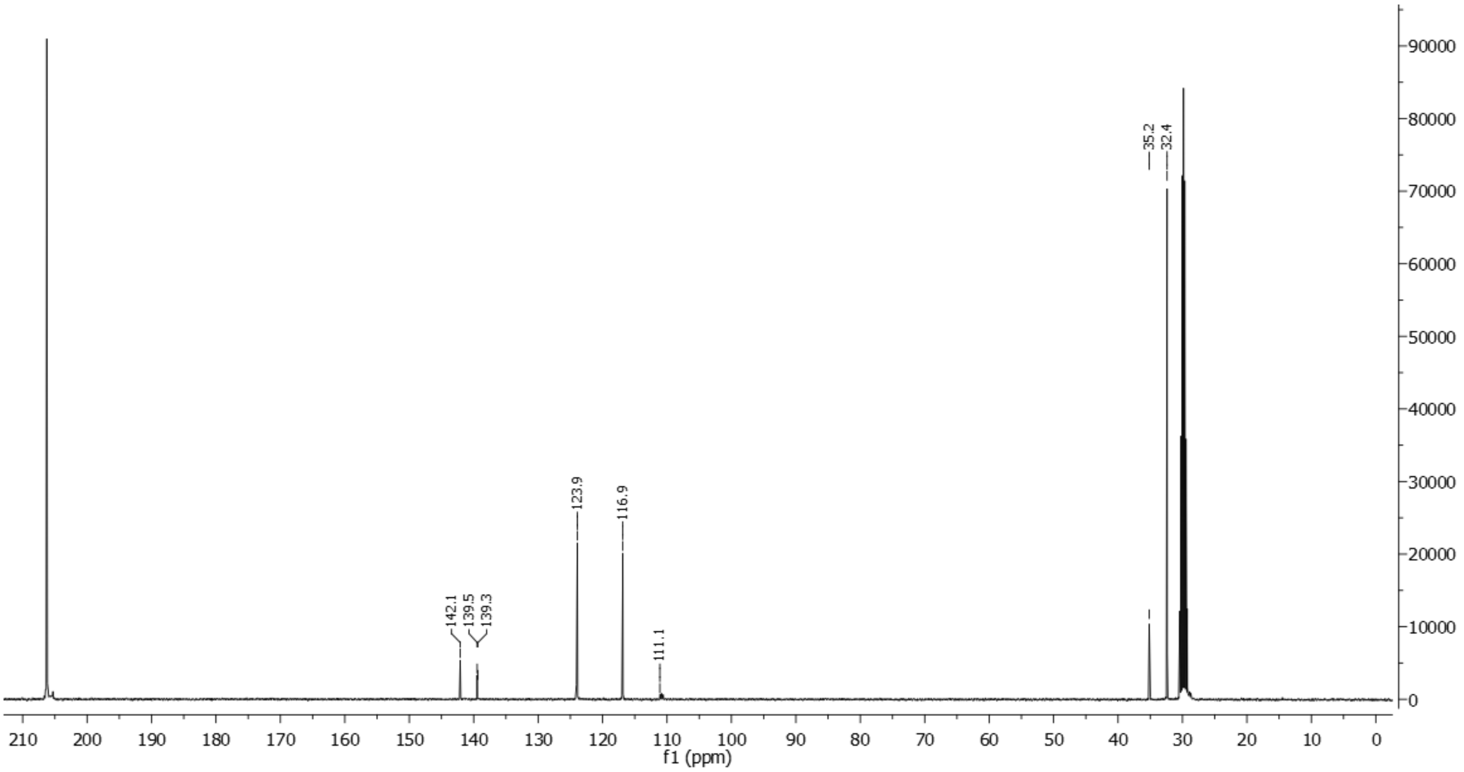

^{13}C-NMR spectrum of **14**

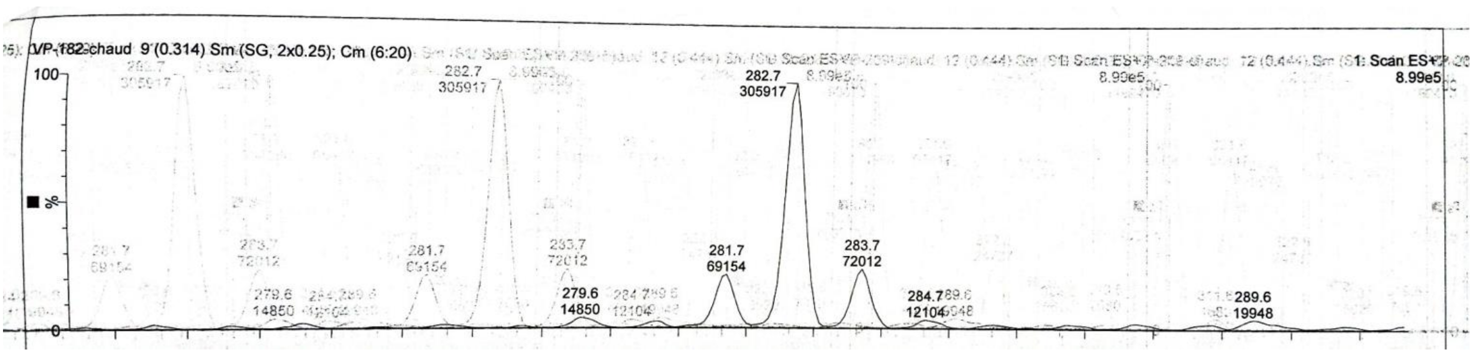

ESI-spectrum of **14**

3,6-Diphenylcarbazole **15**

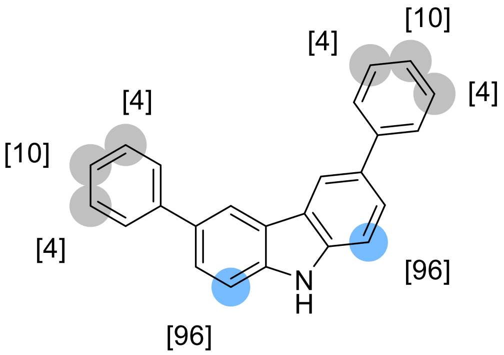

Chemical Formula: $C_{24}H_{17}N$

Substrate	Cs_2CO_3	Solvent (Volume)	RuNp@PVP cat.
63.8mg, 0.2mmol	65.2mg, 0.2mmol	THF (2mL)	14.4mg, 5mol%

Workup and purification:

After cooling down to room temperature the reaction mixture was poured on a 5mM solution of acetic acid in H_2O dist. (100mL). The aqueous phase was extracted three times with EtOAc (3 x 50mL) in a separation funnel. The solvent was removed under vacuum.

Yield: 68mg, 99%, white solid

^{1}H NMR (400 MHz, Acetone-*d*$_6$): δ 10.46 (bs, NH), 8.58 – 8.53 (m, 2H), 7.82 – 7.77 (m, 4H), 7.75 – 7.72 (m, 2H), 7.64 – 7.60 (m, 0.09H), 7.51 – 7.44 (m, 4H), 7.36 – 7.30 (m, 2H).

Deuterium incorporation was expected at *δ* 7.64 – 7.60. Isotopic enrichment values were determined against the integral at *δ* 8.58 – 8.53.

^{2}H-{^{1}H}NMR (92 MHz, Acetone): δ 7.62 (s, 1.91D), 7.48 (s, 0.17D). 7.33 (s, 0.20D)

^{13}C-{^{1}H}NMR (100 MHz, Acetone-*d*$_6$): δ 142.9, 141.0, 133.1, 129.6, 127.8, 127.2, 125.9, 124.9, 119.5, 112.2 (m).

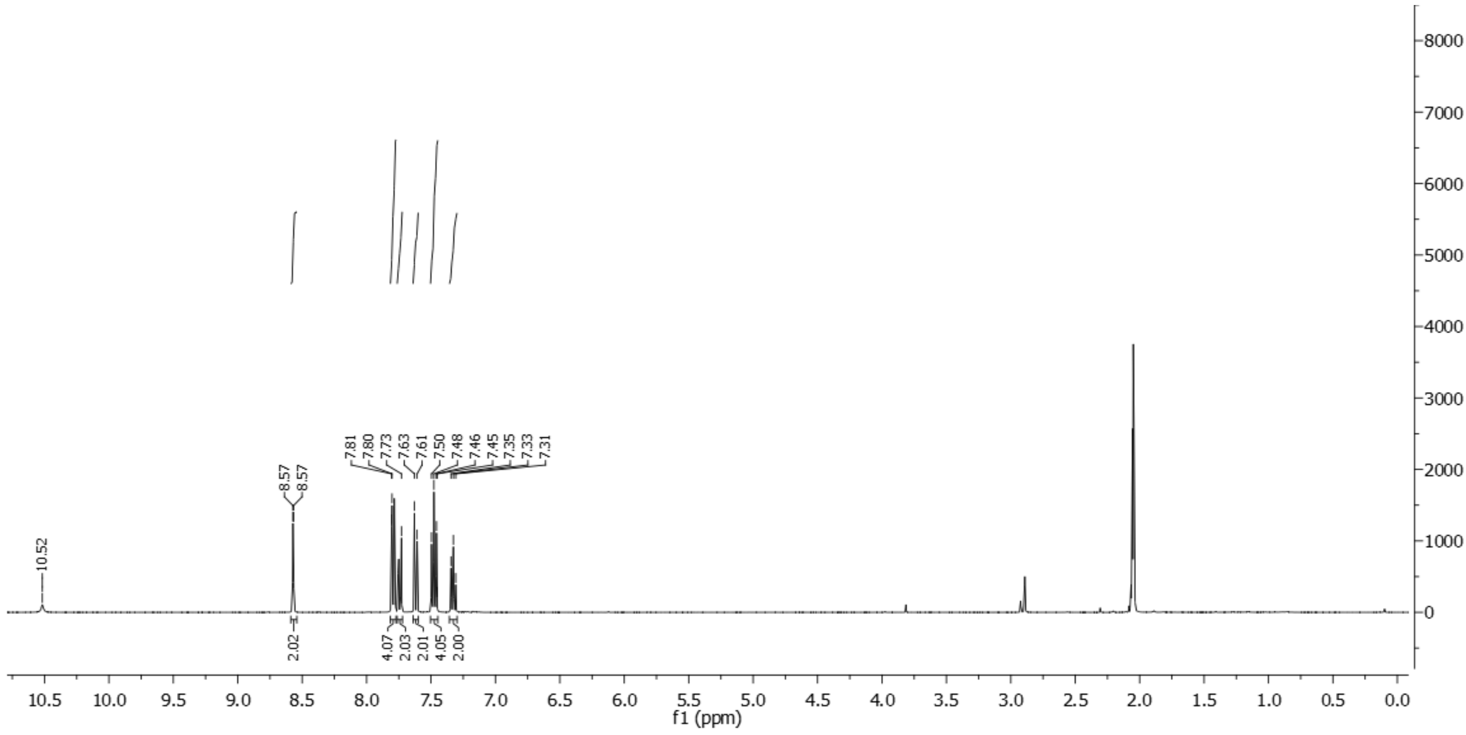

^{1}H-NMR spectrum of the non-deuterated starting material

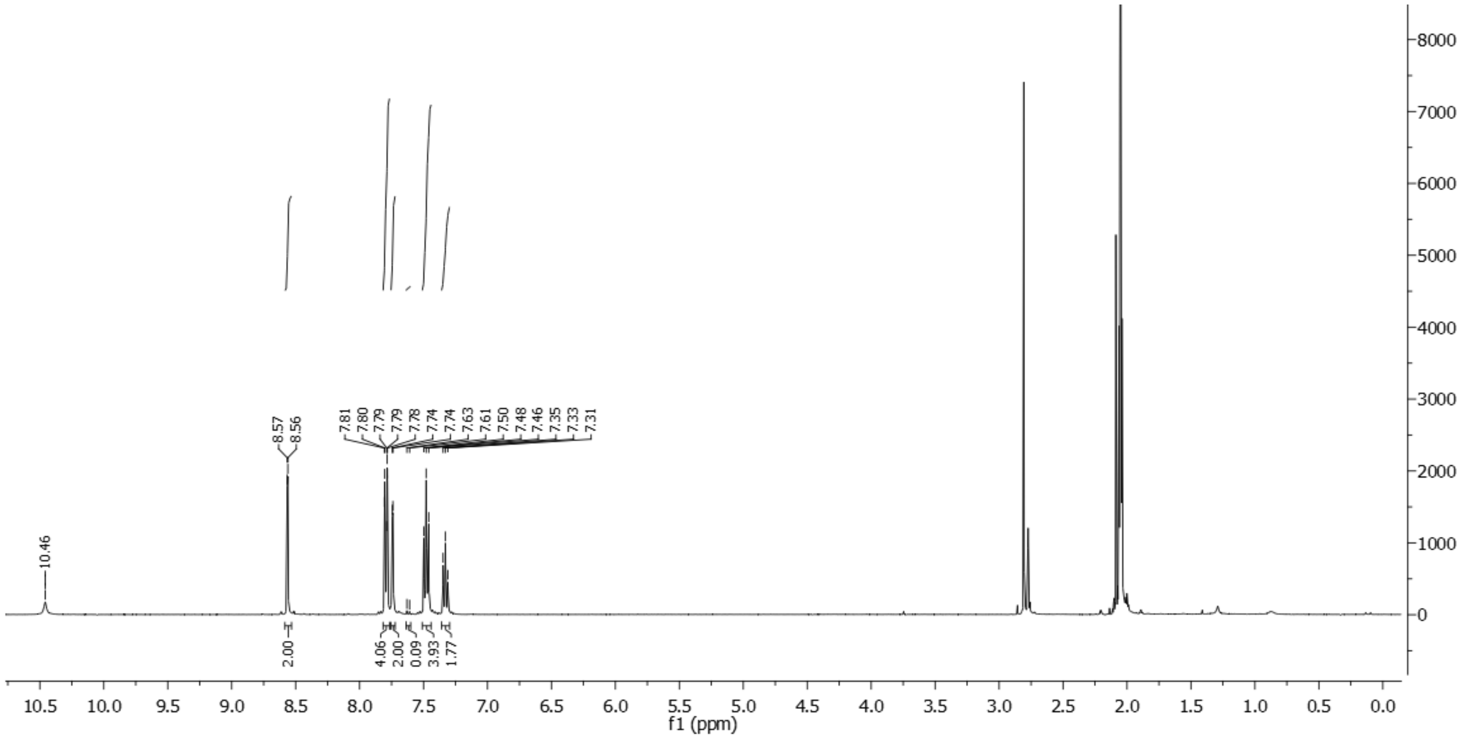

^{1}H-NMR spectrum of **15**

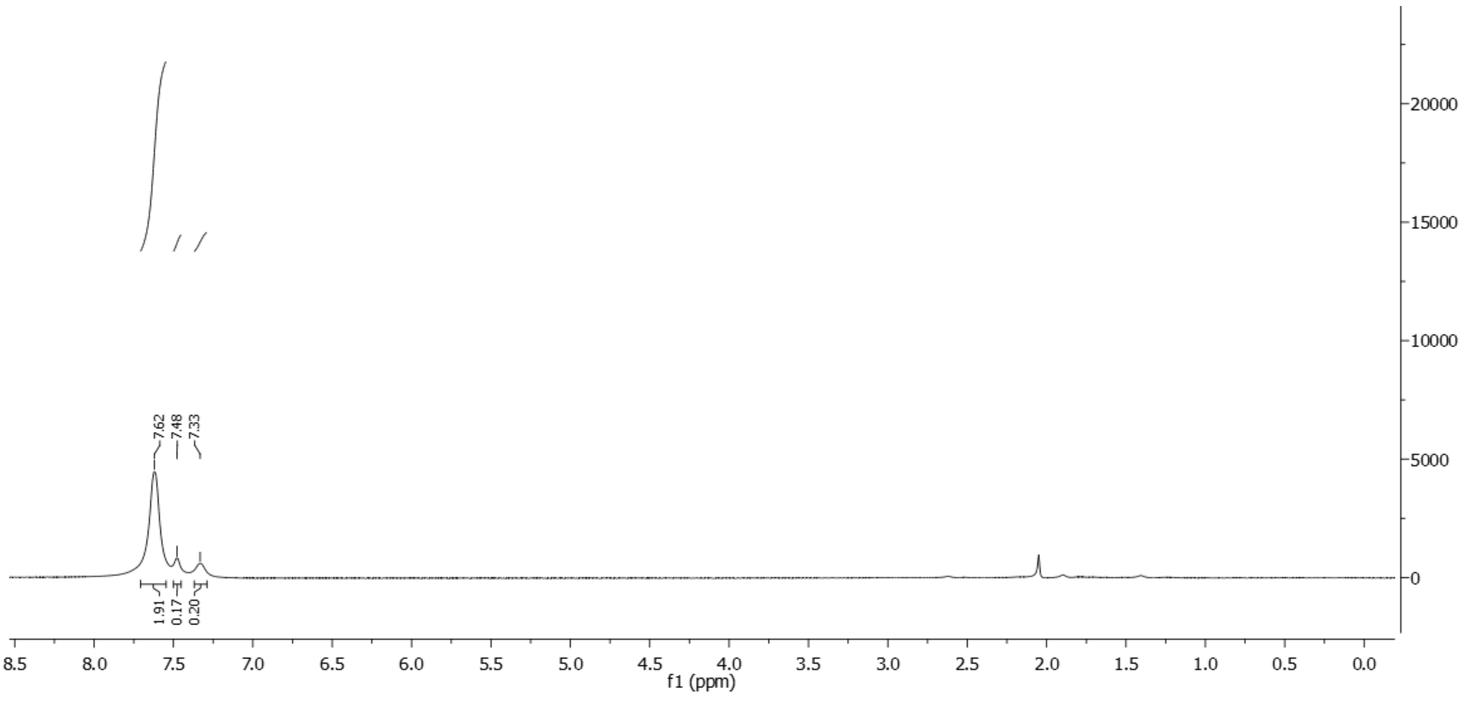

^{2}H-NMR spectrum of **15**

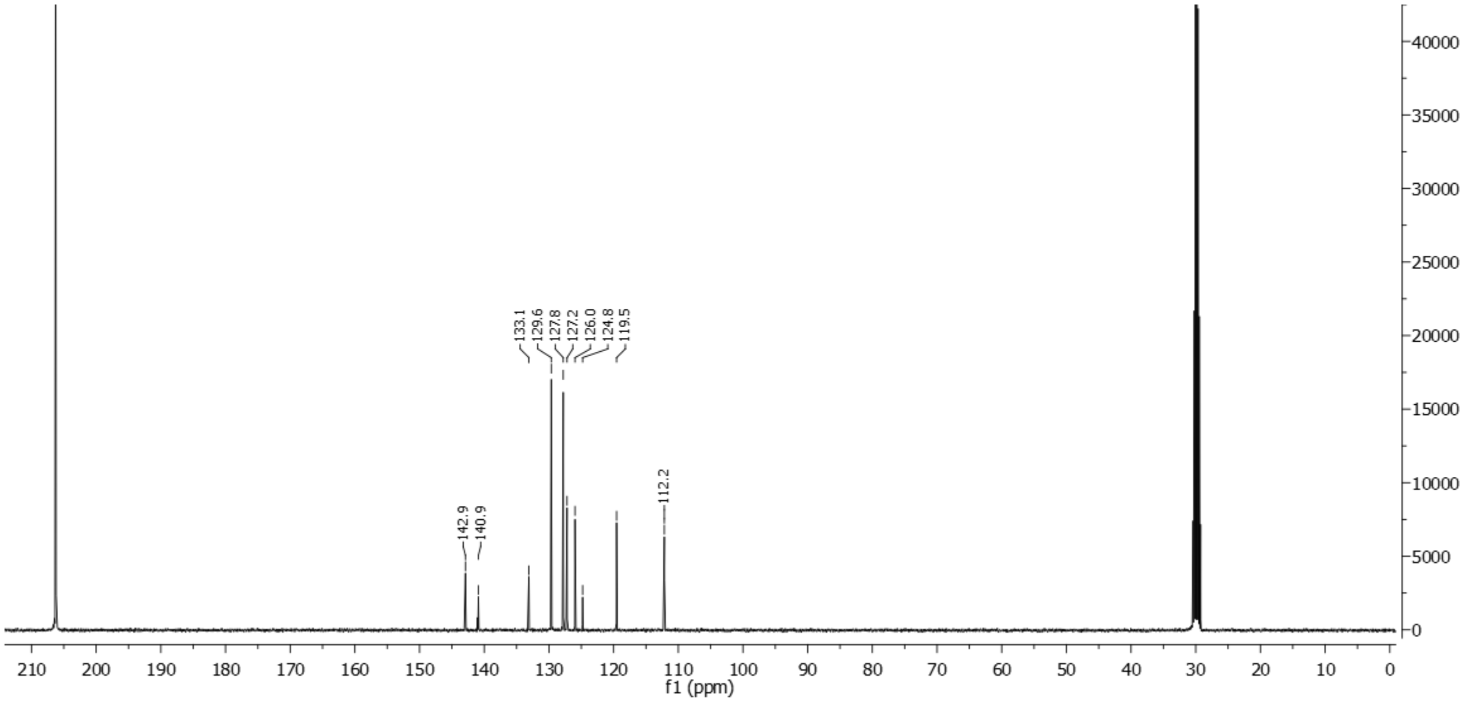

^{13}C-NMR spectrum of the non-deuterated starting material

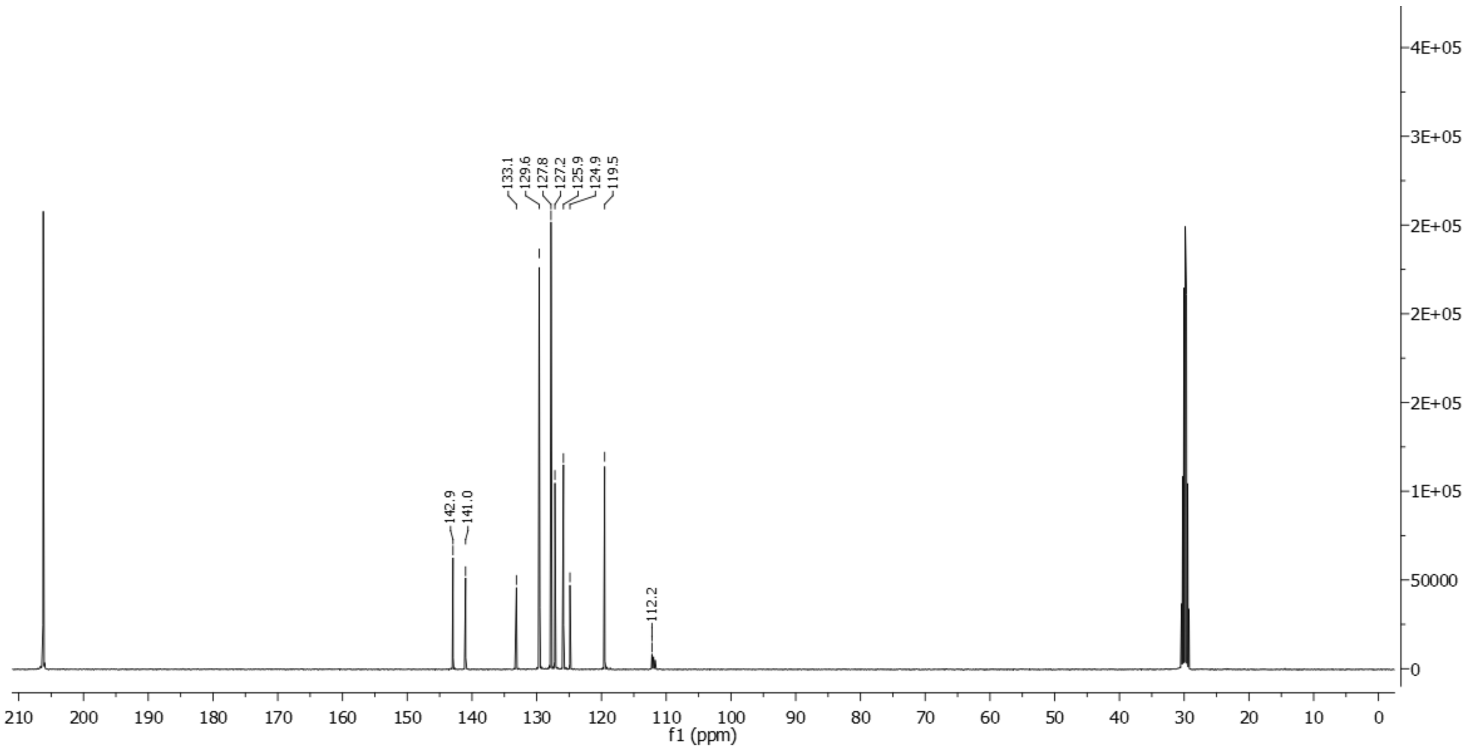

^{13}C-NMR spectrum of **15**

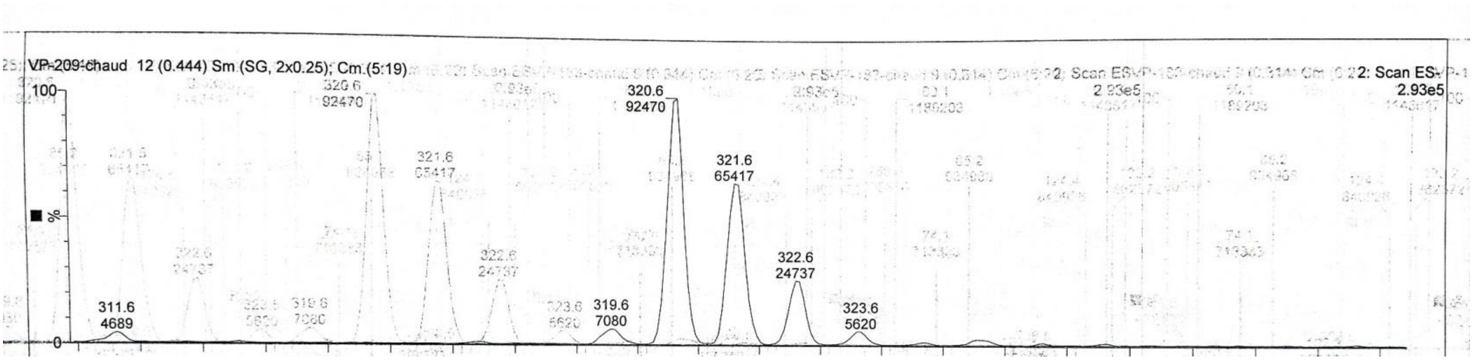

ESI-spectrum of **15**

11,12-Dihydroindolo[2,3-a]carbazole **16**

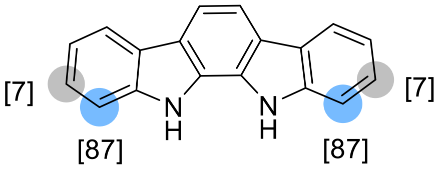

Chemical Formula: $C_{18}H_{12}N_2$

Substrate	Cs_2CO_3	Solvent (Volume)	RuNp@PVP cat.
25.6mg, 0.1mmol	65.2mg, 0.2mmol	THF (2mL)	7.22mg, 10mol%

Workup and purification:

After cooling down to room temperature the reaction mixture was poured on a 5mM solution of acetic acid in H_2O dist. (100mL). The aqueous phase was extracted three times with EtOAc (3 x 50mL) in a separation funnel. The solvent was removed under vacuum.

Yield: 26.0mg, 99%, white solid

^{1}H NMR (400 MHz, Acetone-*d*$_6$): δ 10.50 (bs, NH), 8.19 – 8.14 (m, 2H), 7.98 – 7.93 (m, 2H), 7.63 – 7.59 (m, 0.27H), 7.41 – 7.34 (m, 2H), 7.26 – 7.19 (m, 2H).

Deuterium incorporation was expected at *δ* 7.63 – 7.59. Isotopic enrichment values were determined against the integral at *δ* 8.19 – 8.14.

^{2}H-{^{1}H}NMR (92 MHz, Acetone): δ 7.62 (m, 1.74D), 7.39 (m, 0.15D).

^{13}C-{^{1}H}NMR (100 MHz, Acetone-*d*$_6$): δ 140.3, 126.8, 125.5, 125.3, 121.9, 120.5, 120.0, 112.6, 112.1 (m).

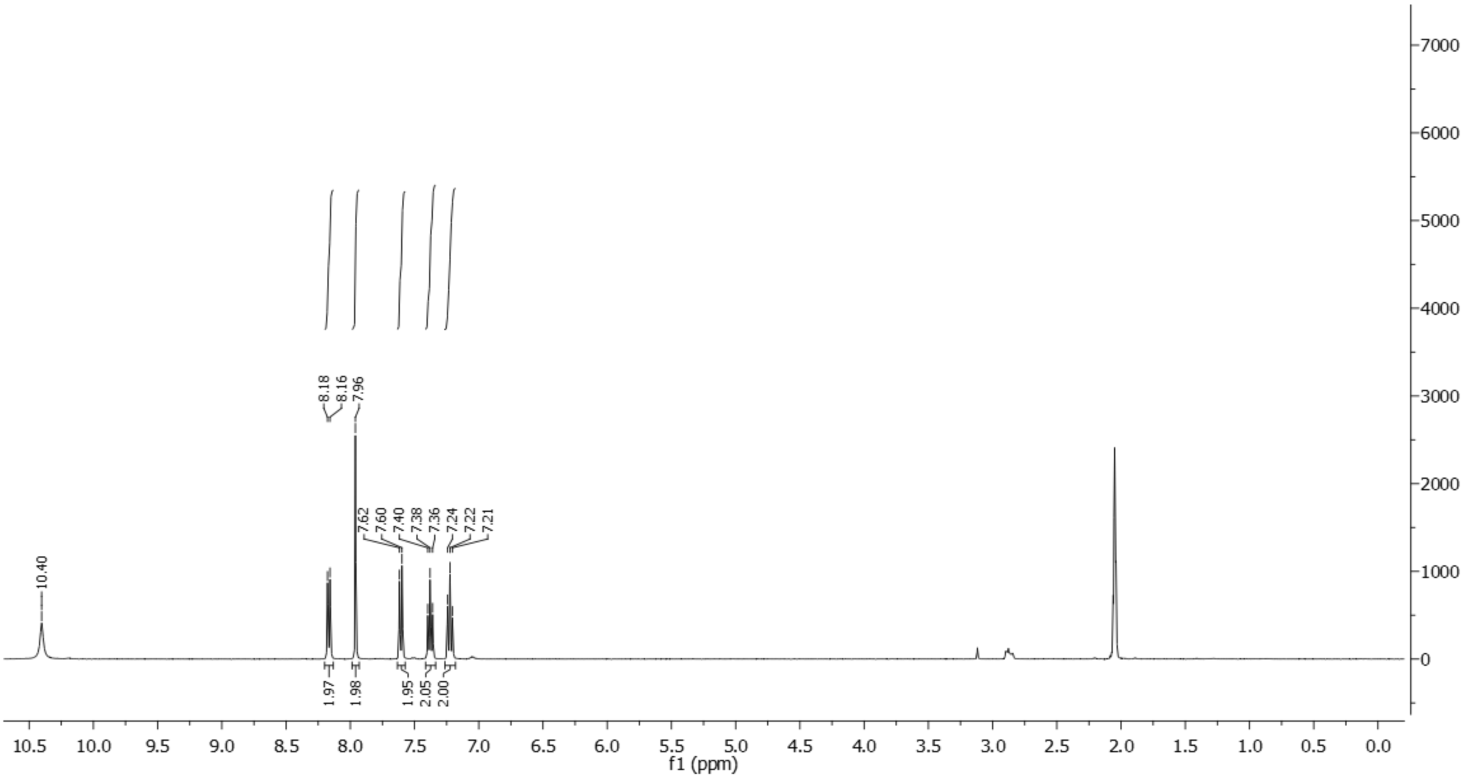

^{1}H-NMR spectrum of the non-deuterated starting material

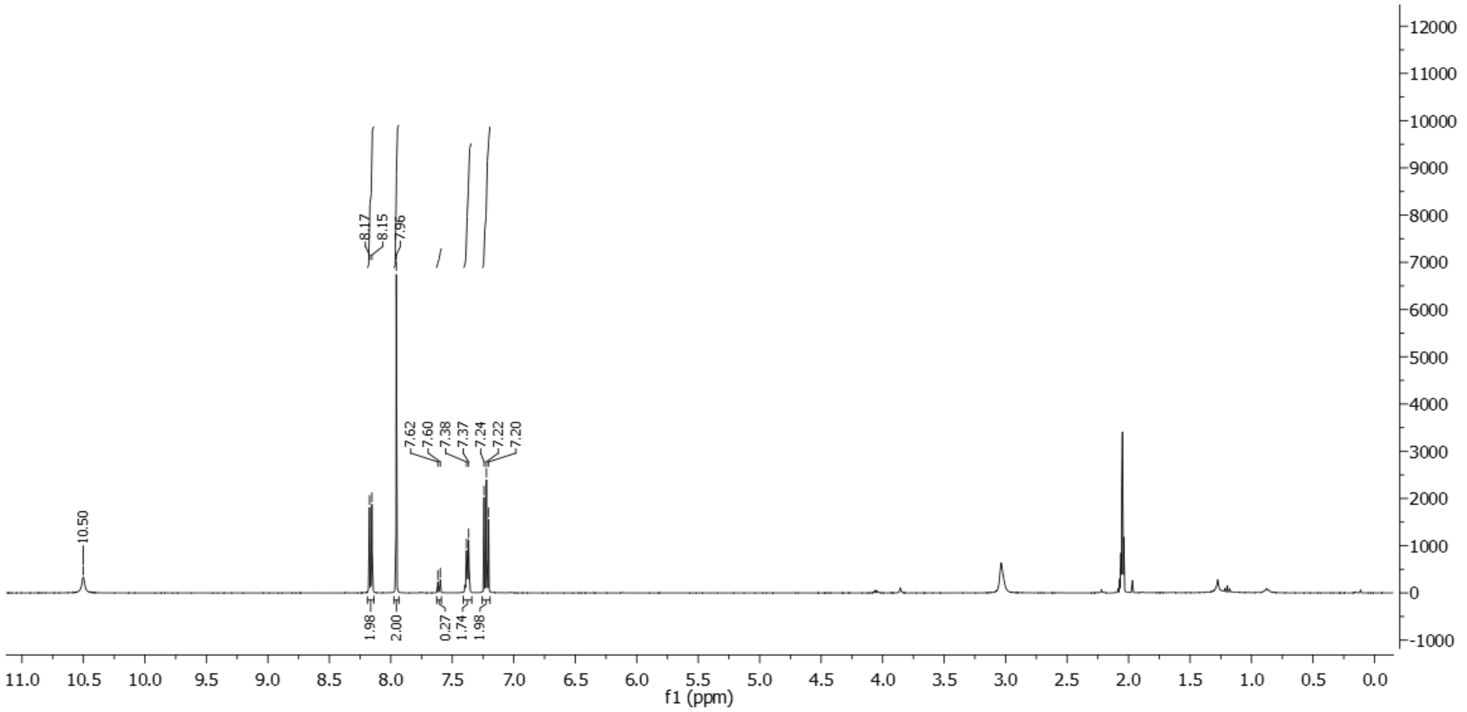

^{1}H-NMR spectrum of **16**

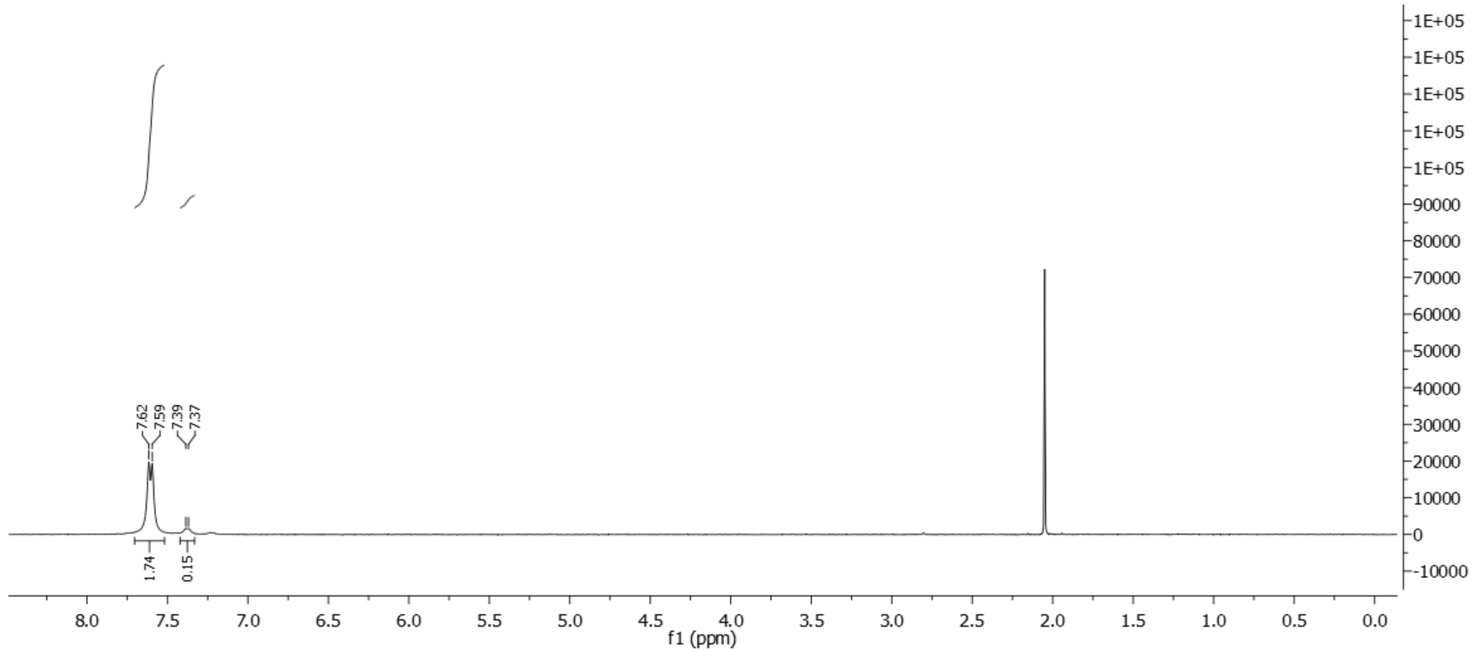

^{2}H-NMR spectrum of **16**

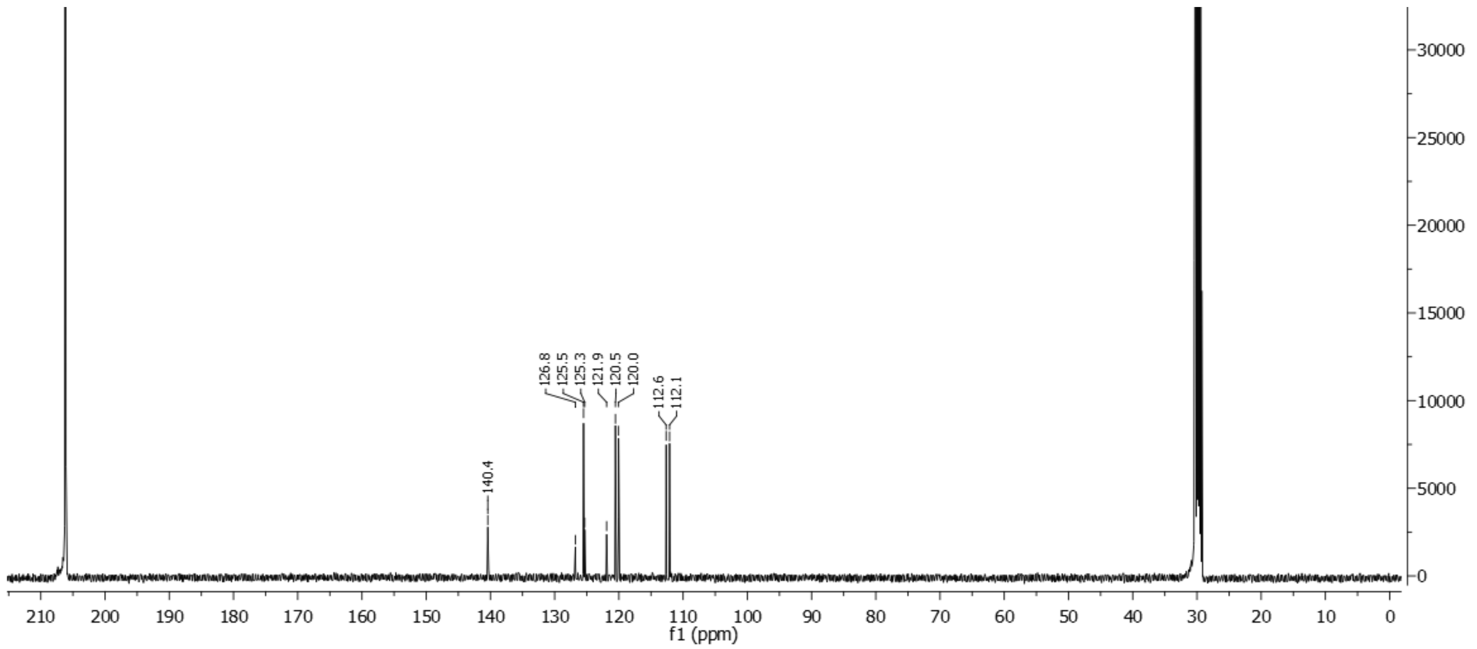

^{13}C-NMR spectrum of the non-deuterated starting material

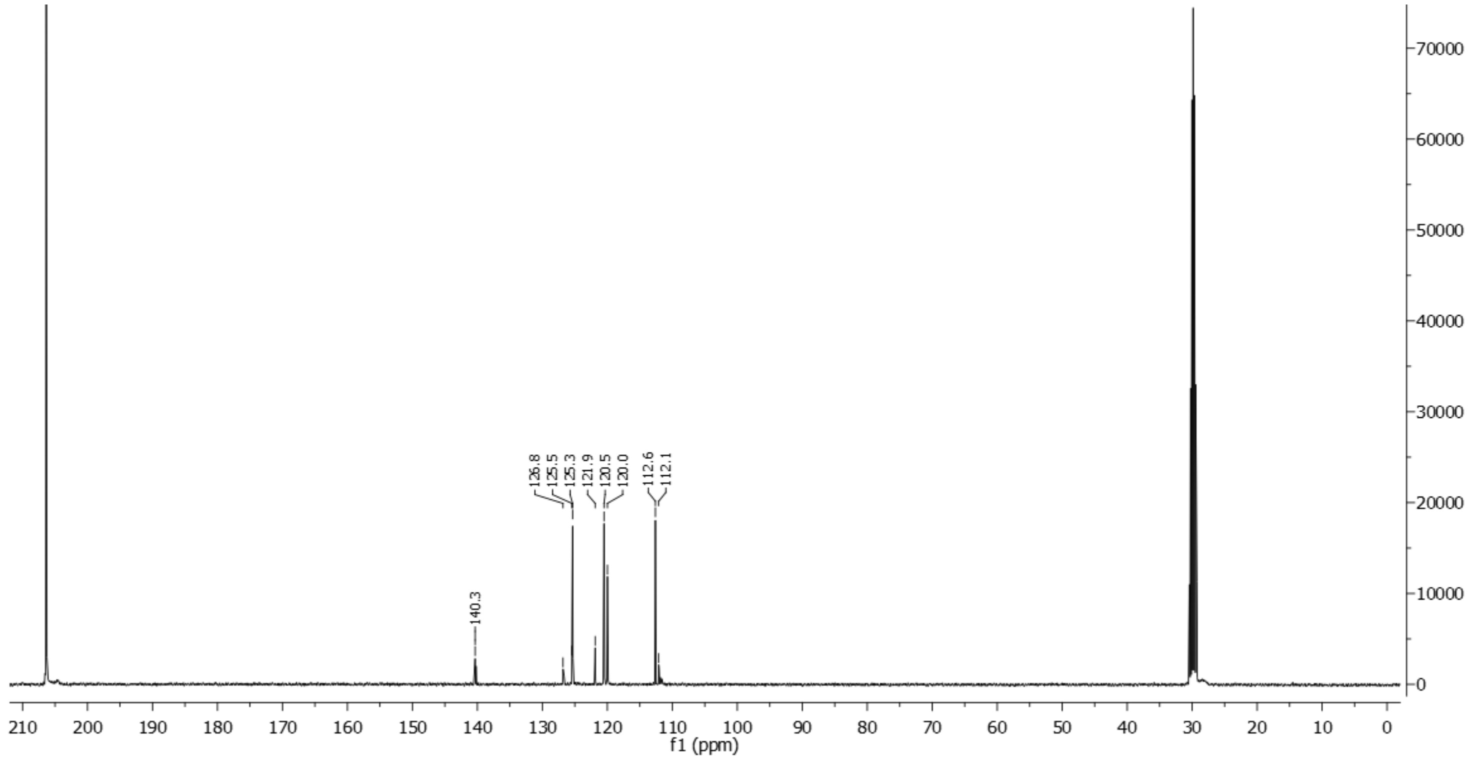

^{13}C-NMR spectrum of **16**

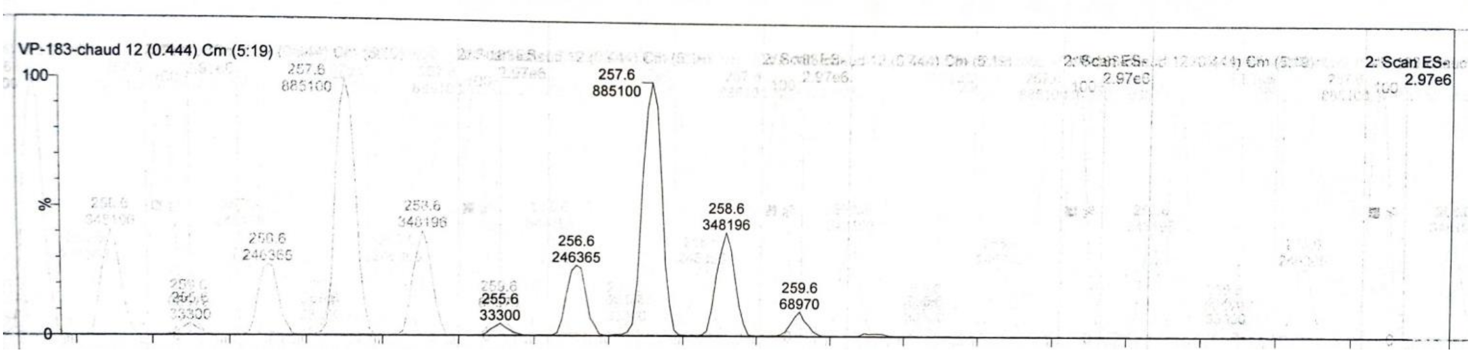

ESI-spectrum of **16**

Deuteration of carbazoles without Cs_2CO_3

Carbazole **13’**

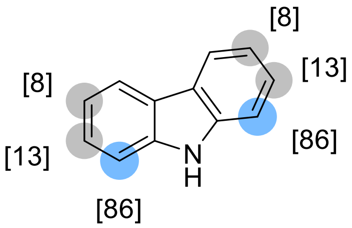

Chemical Formula: $C_{12}H_9N$

Substrate	Solvent (Volume)	RuNp@PVP cat.
33.4mg, 0.2mmol	EtOAc (2mL)	14.4mg, 5mol%

Workup and purification:

After cooling down to room temperature EE:Cy (1:1, 3mL) was added to the reaction mixture and stirred for 10mins to let precipitate RuNp@PVP. The suspension was passed through a SiO_2 pad and the crude product was eluted with THF (5mL). The solvent was removed under vacuum and the crude product was recrystallized from THF and MeOH (THF : MeOH, 10:1).

Yield: 16.0mg, 48%, white solid

^{1}H NMR (400 MHz, Acetone-*d_6*): δ 10.35 (bs, NH), 8.14 – 8.09 (m, 2H), 7.53 – 7.49 (m, 0.28H), 7.41 – 7.35 (m, 1.74H), 7.21 – 7.15 (m, 1.87H).

Deuterium incorporation was expected at *δ* 7.53 – 7.49. Isotopic enrichment values were determined against the integral at *δ* 8.14 – 8.09.

^{2}H-{^{1}H}NMR (600 MHz, Acetone): δ 7.51 (s, 1.72D), 7.39 (s, 0.26D), 7.18 (s, 0.16D).

^{13}C-{^{1}H}NMR (100 MHz, Acetone-*d_6*): δ 140.8, 126.3, 123.9, 120.8, 119.6, 111.6 (m).

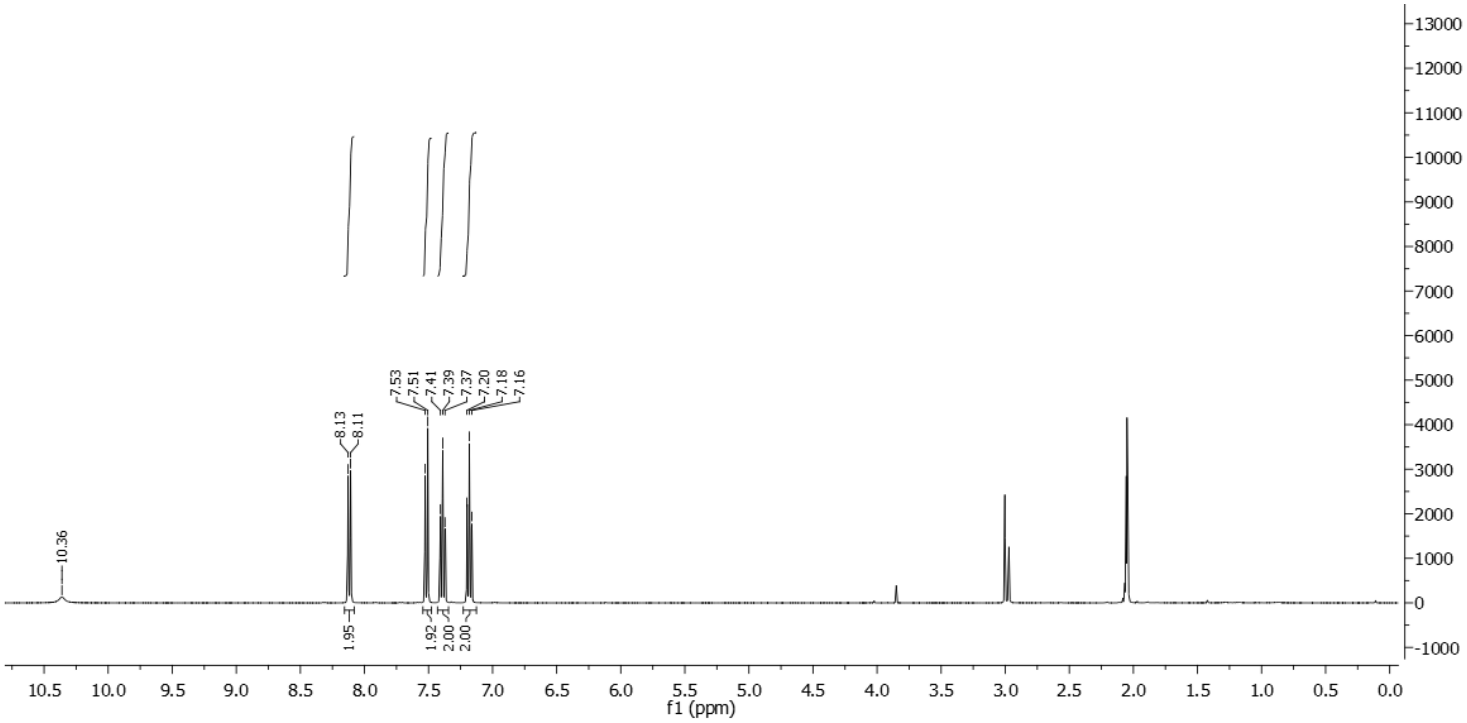

^{1}H-NMR spectrum of the non-deuterated starting material

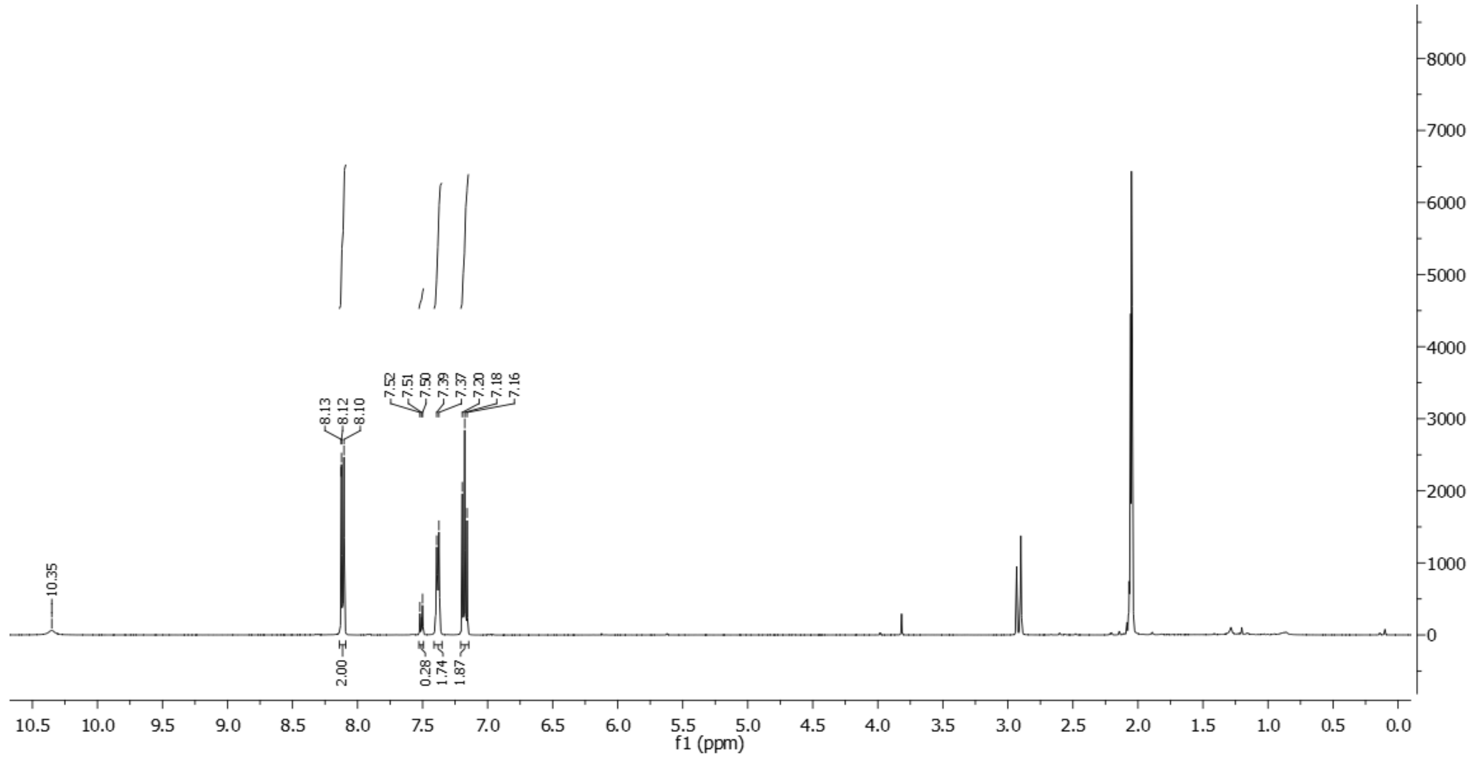

^{1}H-NMR spectrum of **13’**

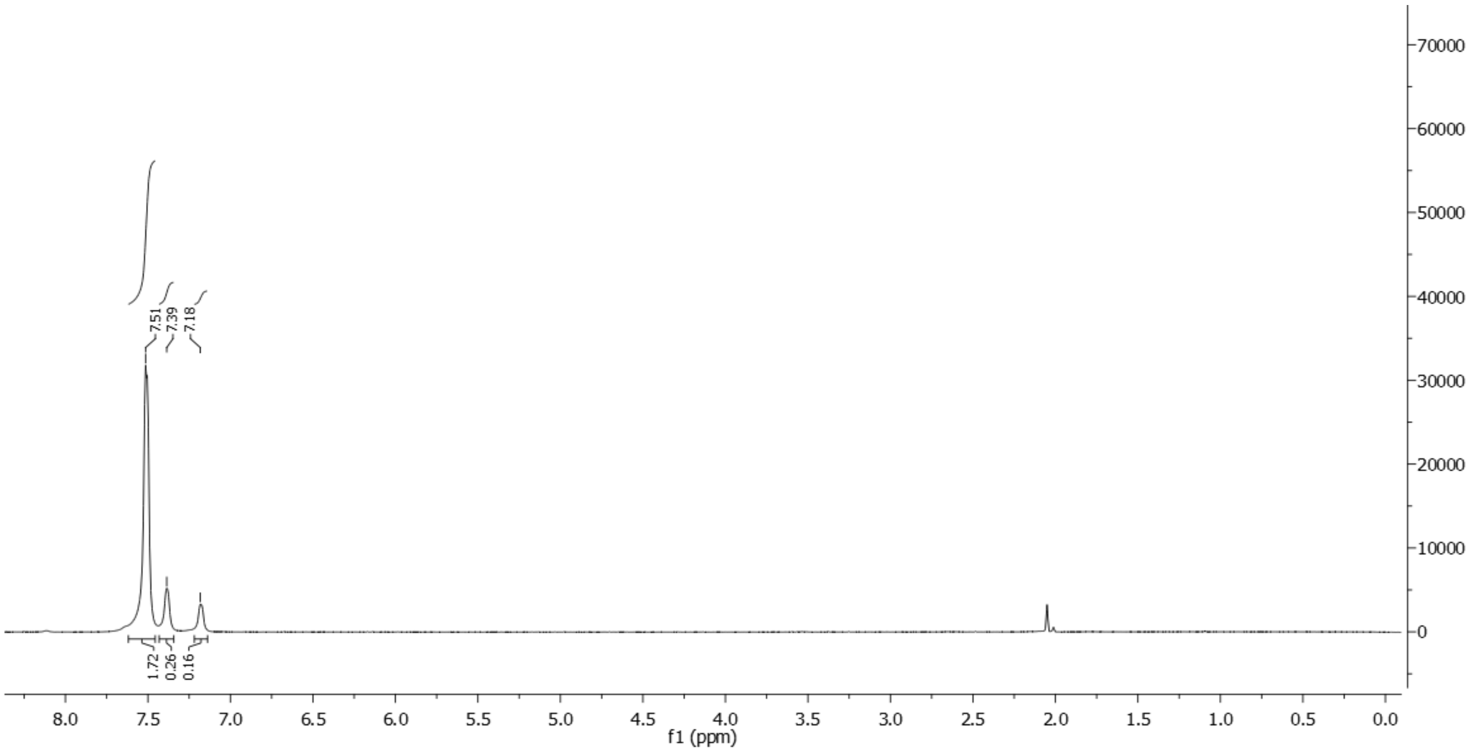

^{2}H-NMR spectrum of **13’**

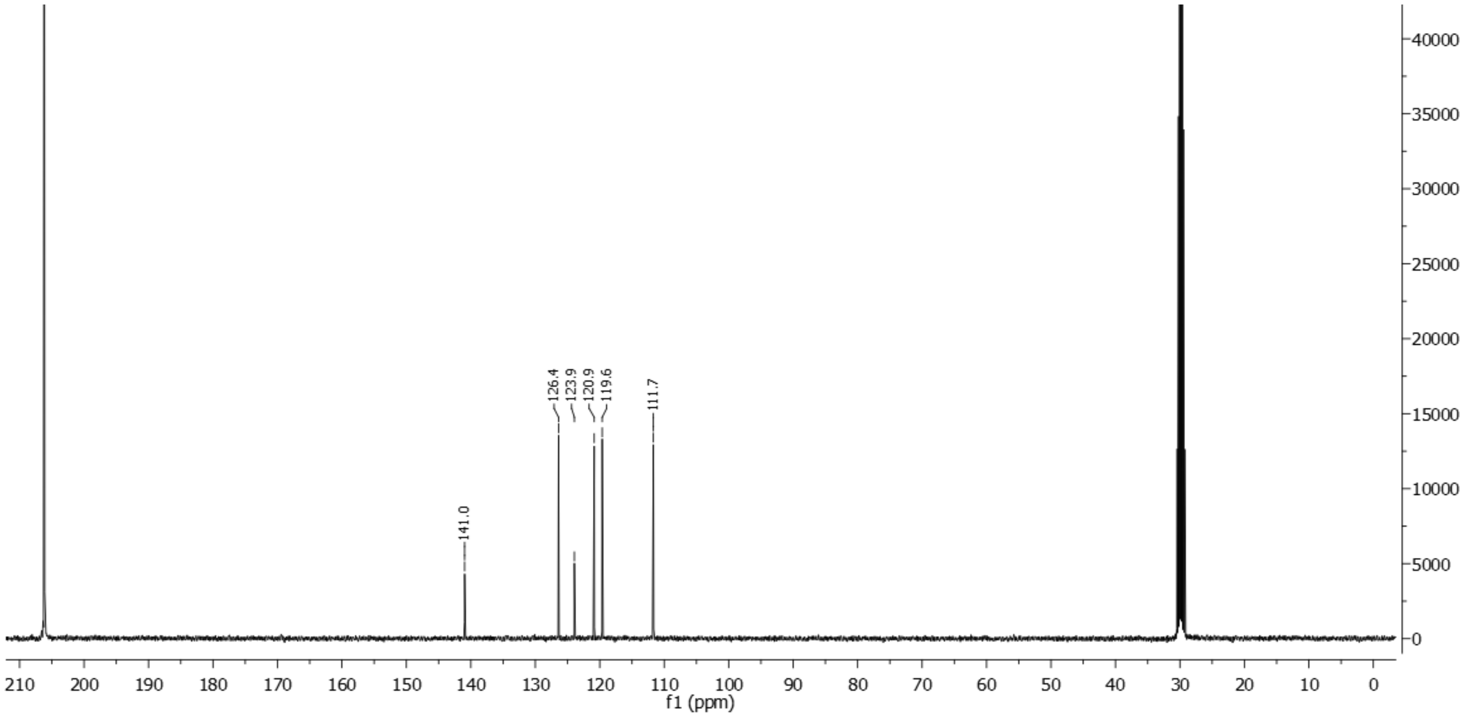

^{13}C-NMR spectrum of the non-deuterated starting material

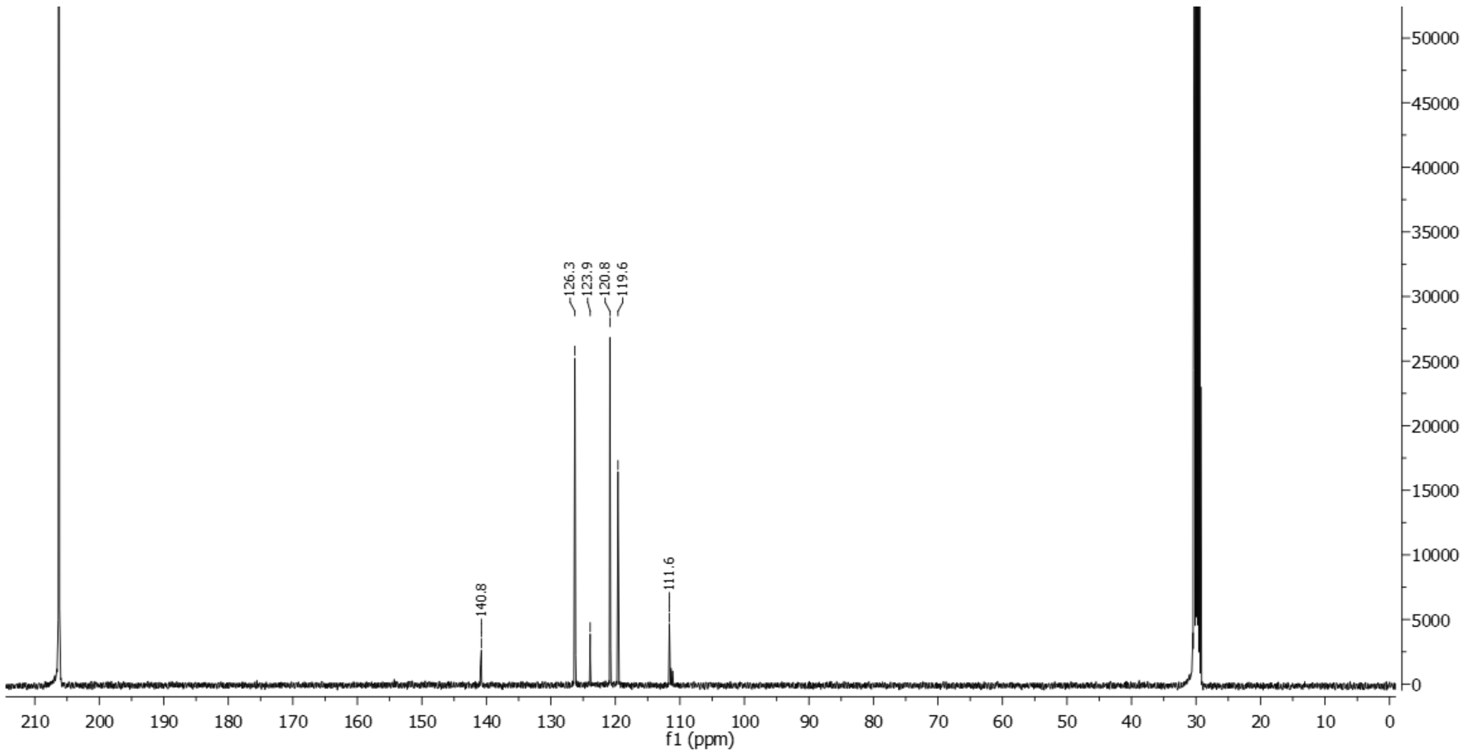

^{13}C-NMR spectrum of **13'**

3,6-Di-*tert*-butylcarbazole **14'**

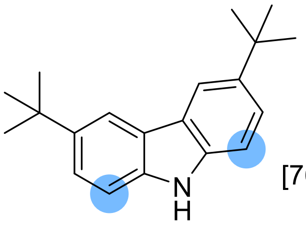

Chemical Formula: $C_{20}H_{25}N$

Substrate	Solvent (Volume)	RuNp@PVP cat.
22.9mg, 0.1mmol	THF (2mL)	14.4mg, 10mol%

Workup and purification:

After cooling down to room temperature EE:Cy (1:1, 3mL) was added to the reaction mixture and stirred for 10mins to let precipitate RuNp@PVP. The suspension was passed through a SiO_2 pad and the crude product was eluted with THF (5mL). The solvent was removed under vacuum and the crude product was recrystallized from THF and MeOH (THF : MeOH, 10:1).

Yield: 10.0mg, 44%, white solid

^{1}H NMR (400 MHz, Acetone-*d*$_6$): δ 10.02 (bs, NH), 8.23 – 8.16 (m, 2H), 7.51 – 7.43 (m, 2H), 7.42 – 7.37 (m, 0.48H), 1.43 (s, 18H).

Deuterium incorporation was expected at *δ* 7.42 – 7.37. Isotopic enrichment values were determined against the integral at *δ* 8.23 – 8.16.

^{2}H-{^{1}H}NMR (600 MHz, Acetone): δ 7.39 (s).

^{13}C-{^{1}H}NMR (100 MHz, Acetone-*d*$_6$): δ 142.1, 139.5, 139.5, 123.9, 116.9, 111.1 (m), 35.2, 32.4.

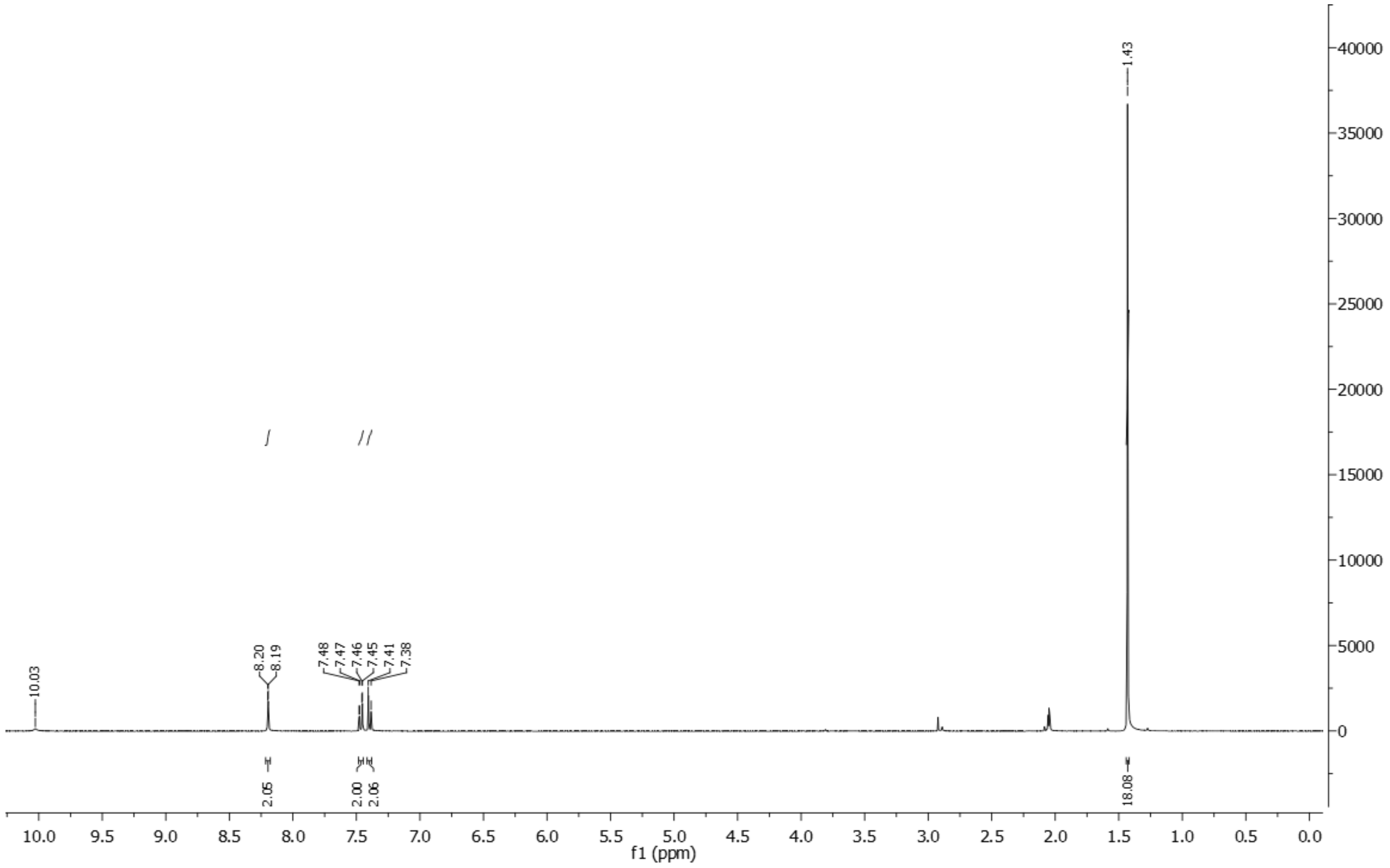

^{1}H-NMR spectrum of the non-deuterated starting material

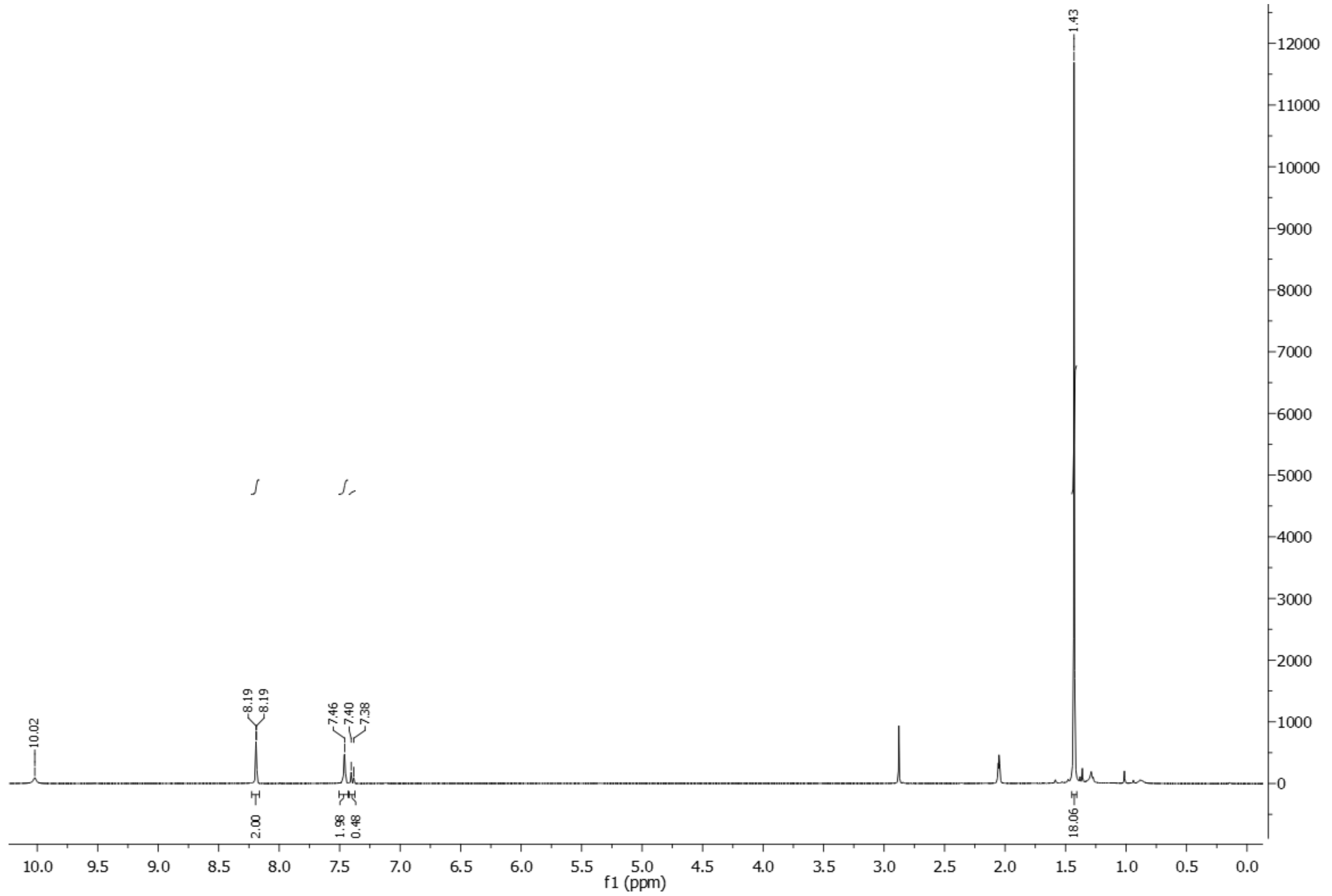

^{1}H-NMR spectrum of **14’**

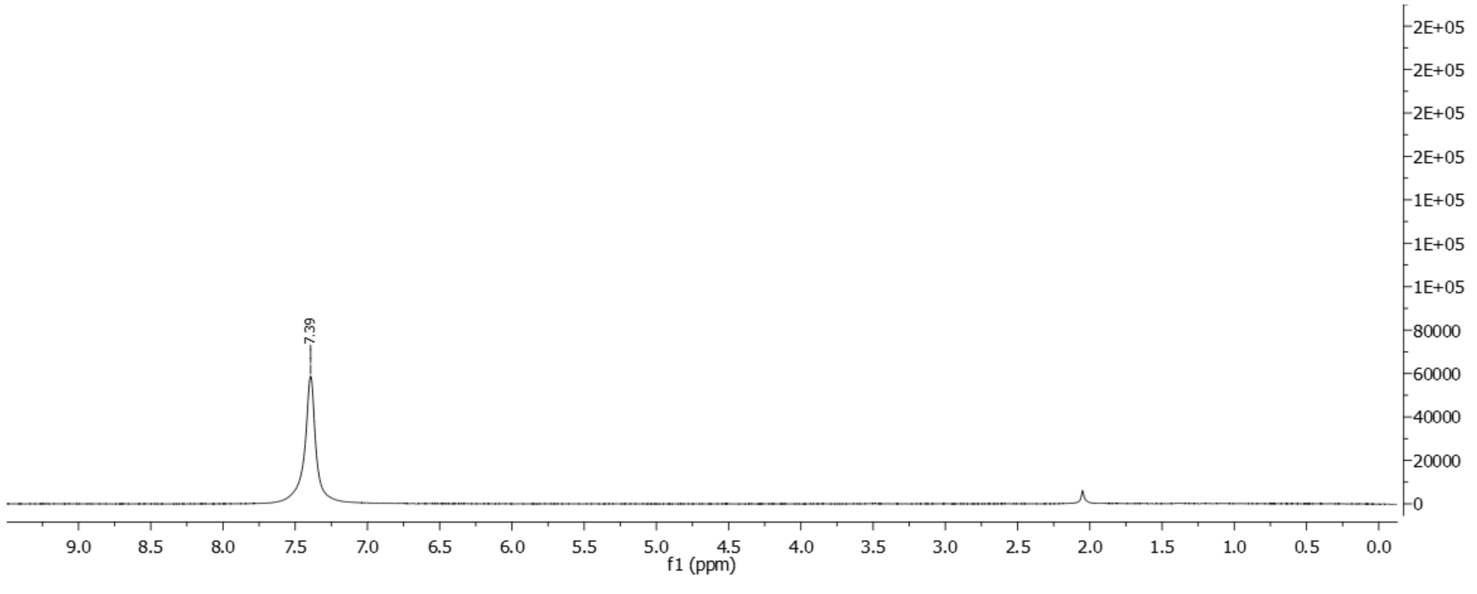

^{2}H-NMR spectrum of **14’**

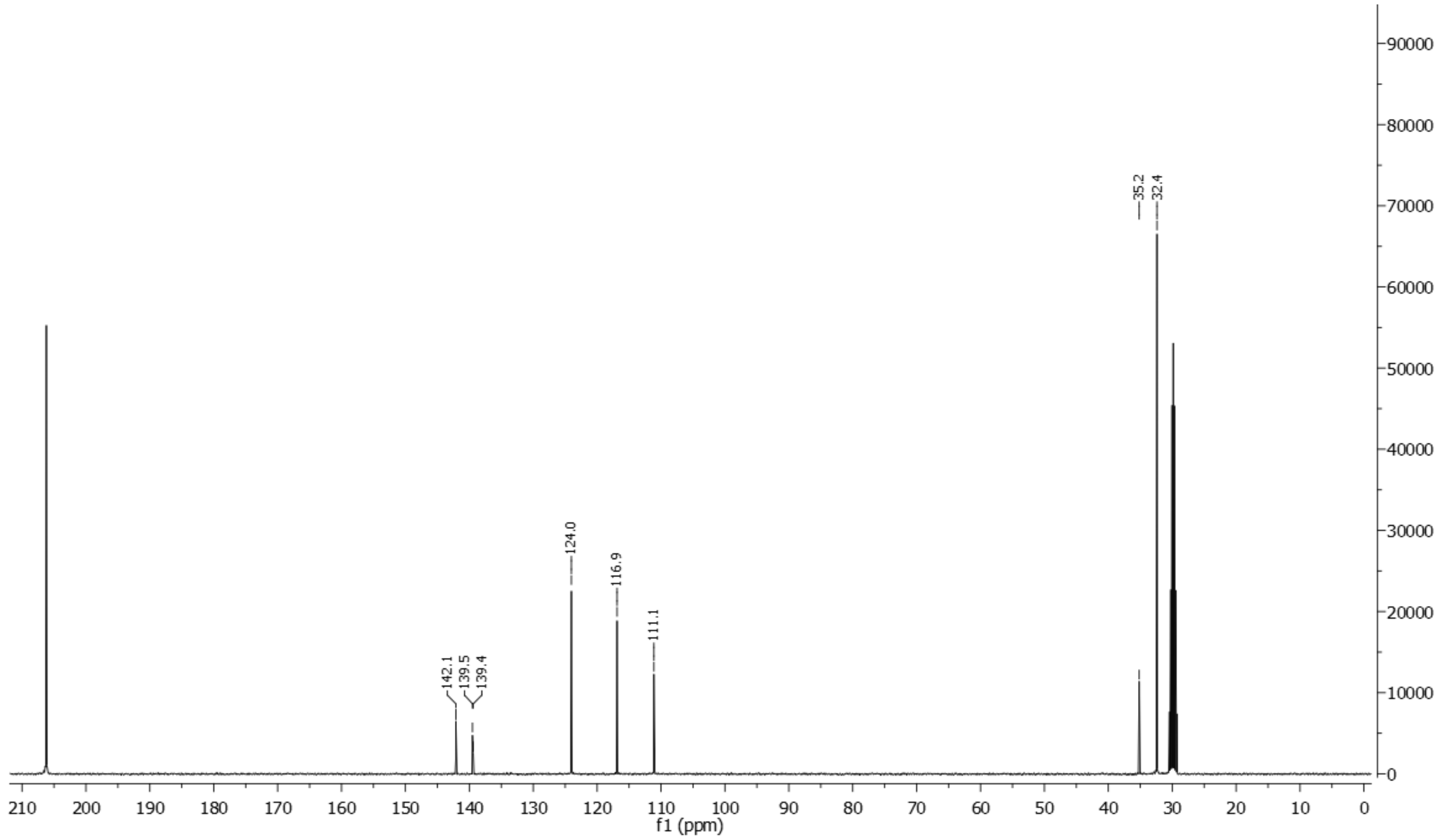

^{13}C-NMR spectrum of the non-deuterated starting material

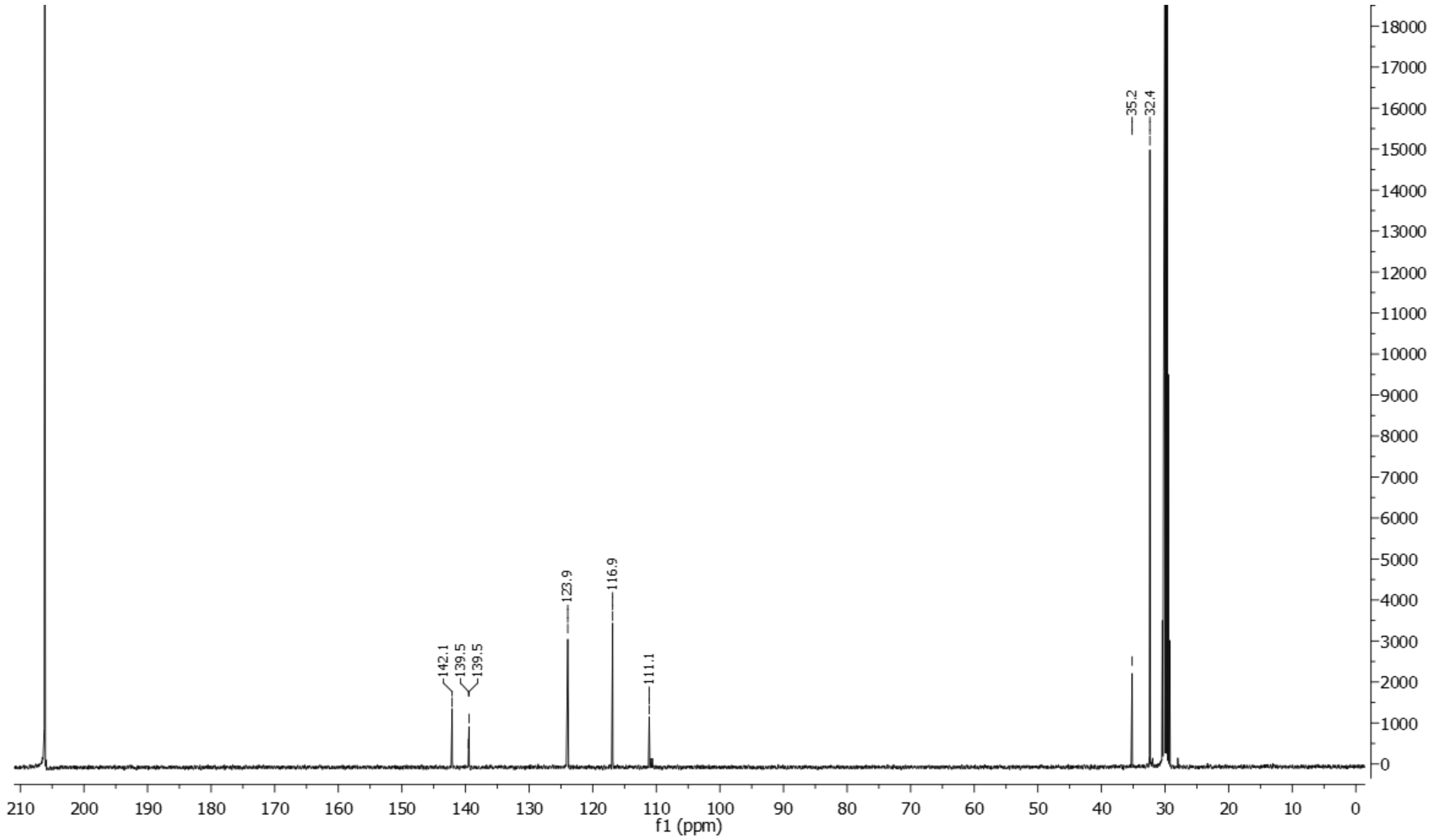

^{13}C-NMR spectrum of **14'**

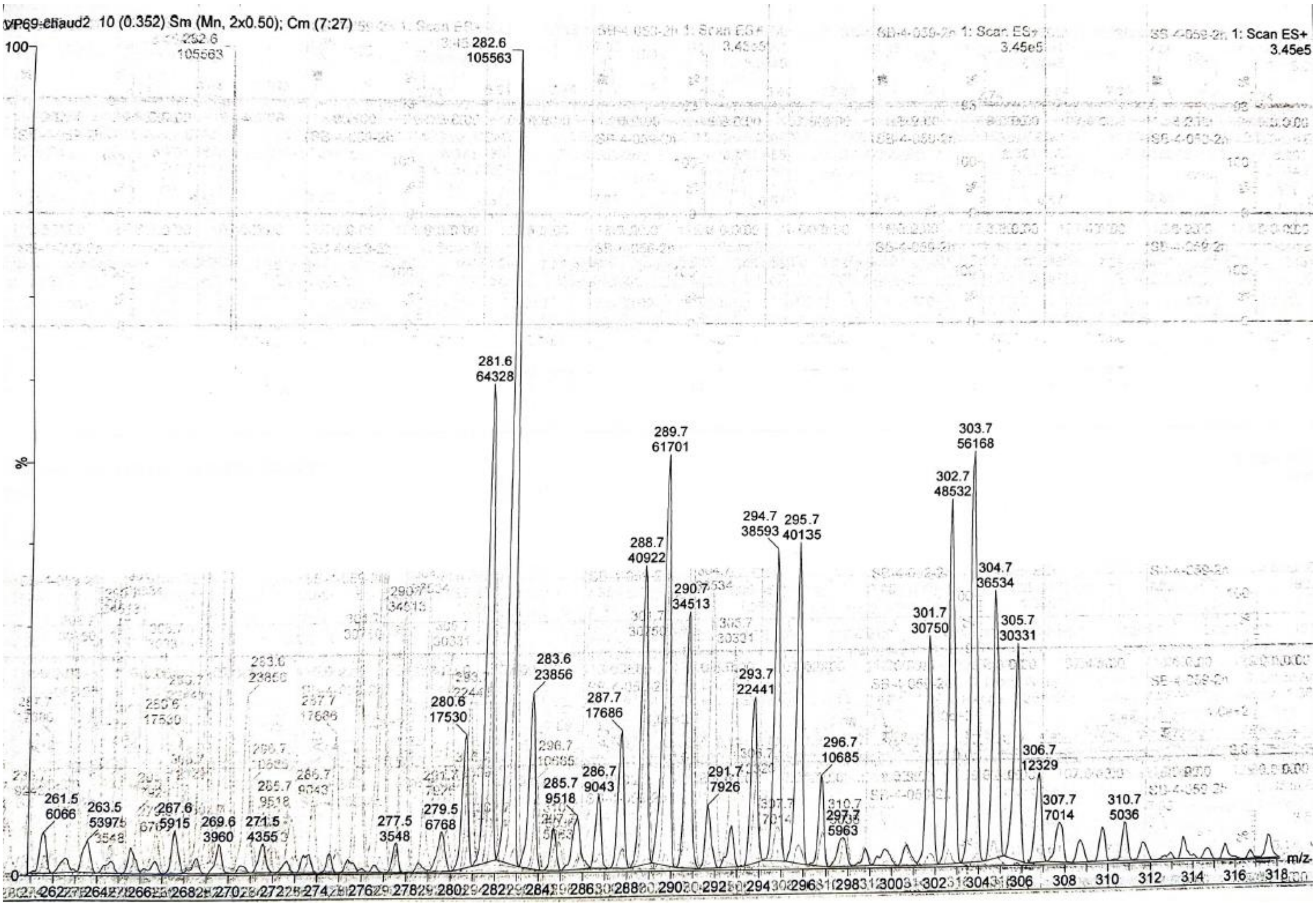

ESI-spectrum of **14’**

3,6-Diphenylcarbazole **15’**

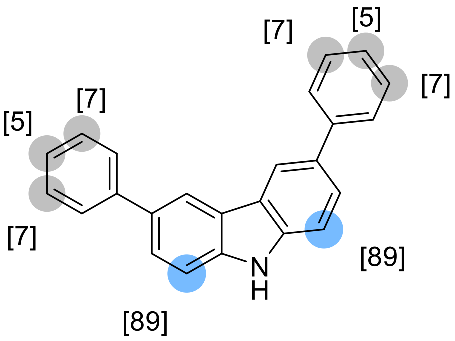

Chemical Formula: $C_{24}H_{17}N$

Substrate	Solvent (Volume)	RuNp@PVP cat.
31.9mg, 0.1mmol	THF (2mL)	14.4mg, 10mol%

Workup and purification:

After cooling down to room temperature EE:Cy (1:1, 3mL) was added to the reaction mixture and stirred for 10mins to let precipitate RuNp@PVP. The suspension was passed through a SiO_2 pad and the crude product was eluted with EtOAc (5mL). The solvent was removed under vacuum and the crude product was purified over SiO_2. The product was eluted with 10:1 cyclohexane / ethylacetate.

Yield: 10.0mg, 31%, white solid

^{1}H NMR (400 MHz, Acetone-*d$_6$*): δ 8.58 – 8.55 (m, 2H), 7.82 – 7.77 (m, 4H), 7.76 – 7.72 (m, 2H), 7.63 – 7.60 (m, 0.23H), 7.51 – 7.44 (m, 4H), 7.36 – 7.29 (m, 2H).

Deuterium incorporation was expected at *δ* 7.63 – 7.60. Isotopic enrichment values were determined against the integral at *δ* 8.58 – 8.55.

^{2}H-{^{1}H}NMR (600 MHz, Acetone): δ 7.62 (s).

^{13}C-{^{1}H}NMR (100 MHz, Acetone-*d$_6$*): δ 142.9, 140.9, 133.1, 129.6, 127.8, 127.2, 125.8, 124.8, 119.5, 112.2 (m).

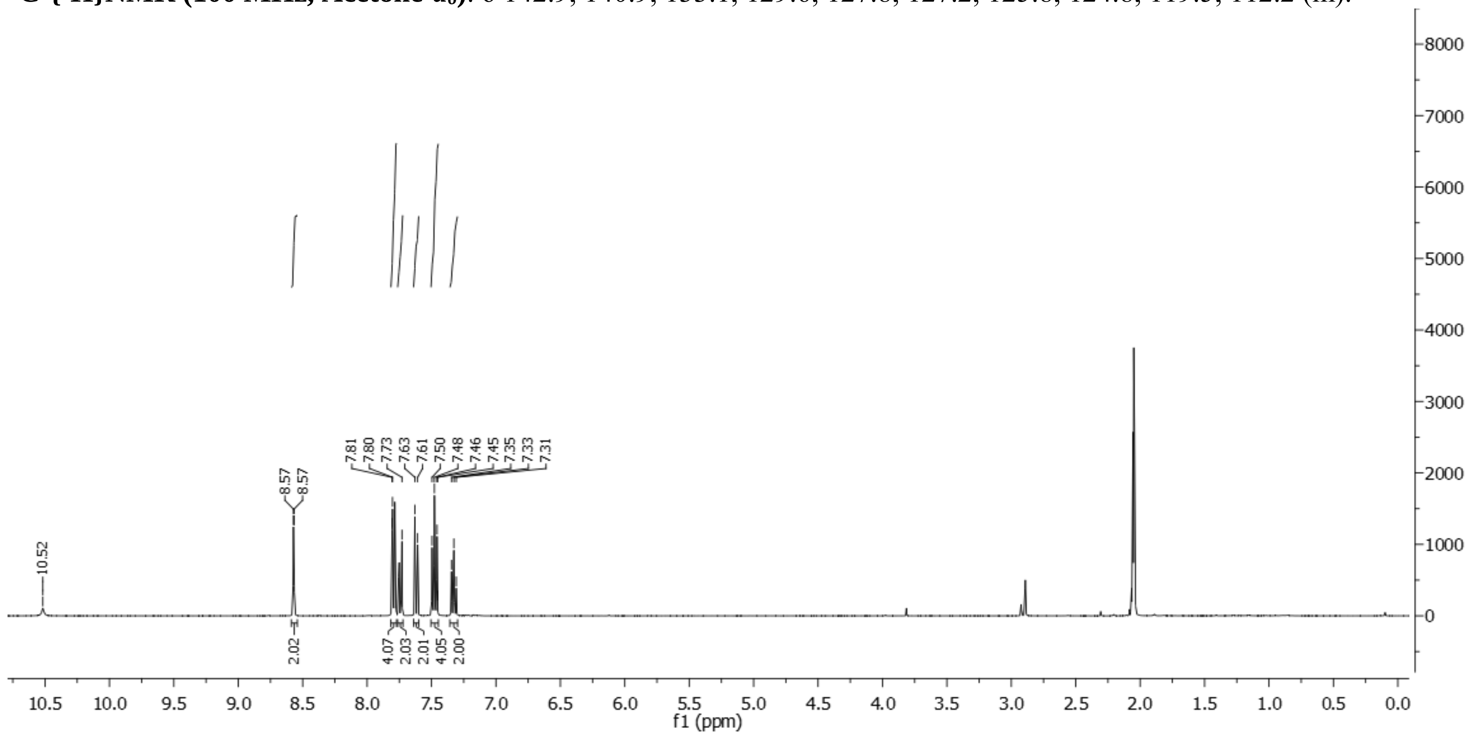

^{1}H-NMR spectrum of the non-deuterated starting material

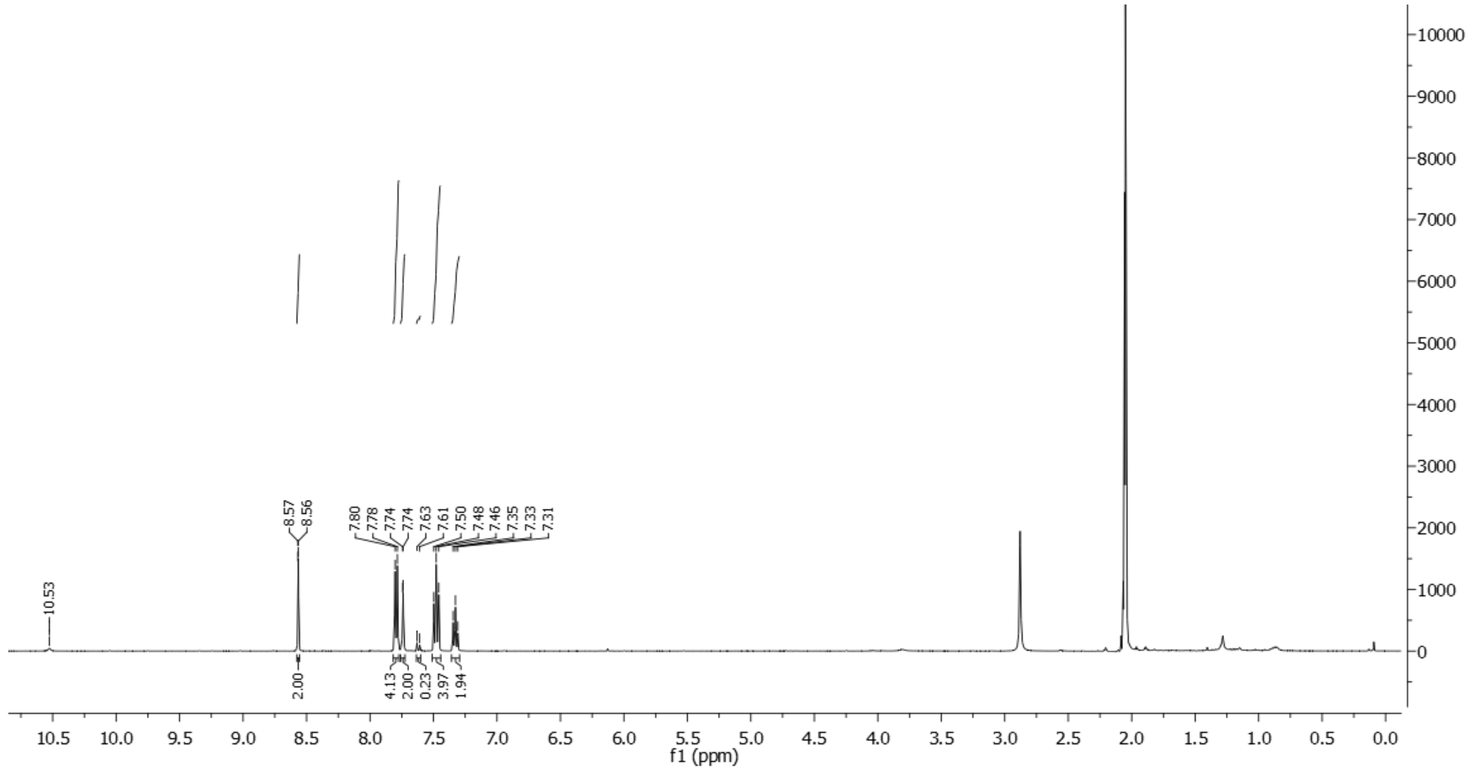

^{1}H-NMR spectrum of **15'**

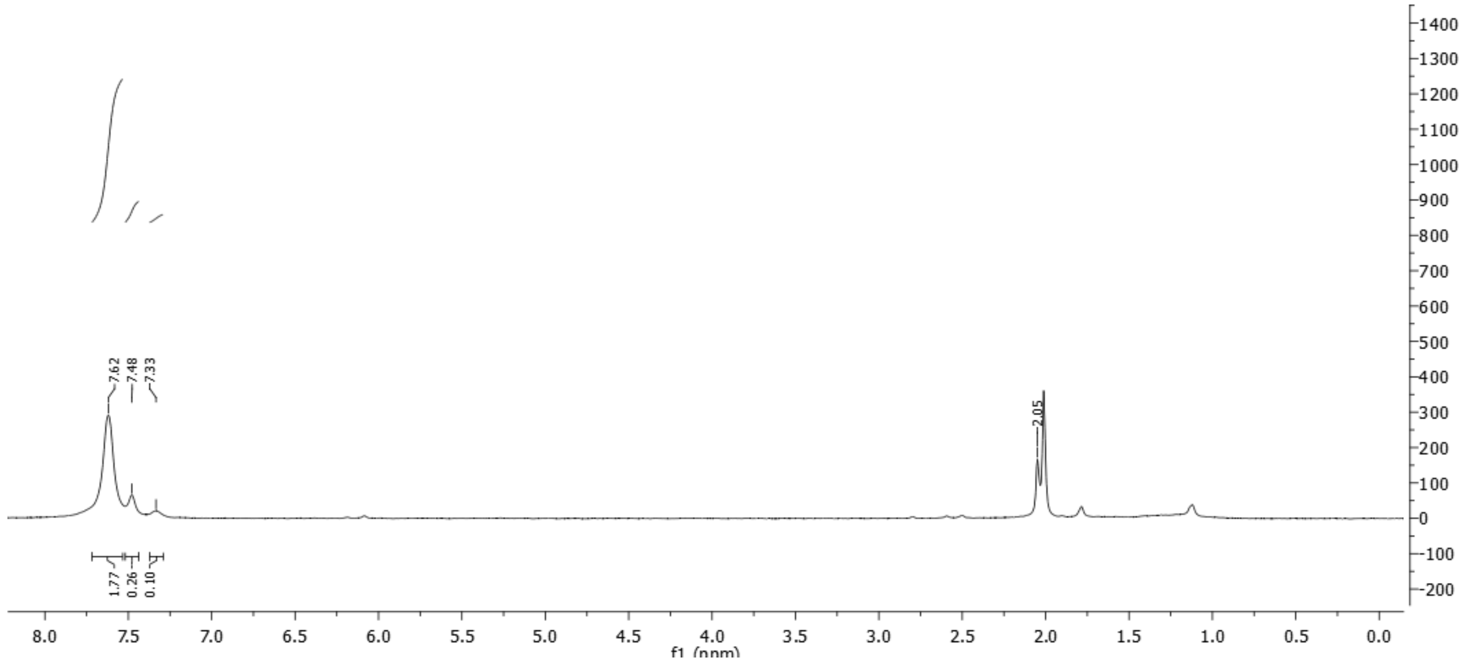

^{2}H-NMR spectrum of **15'**

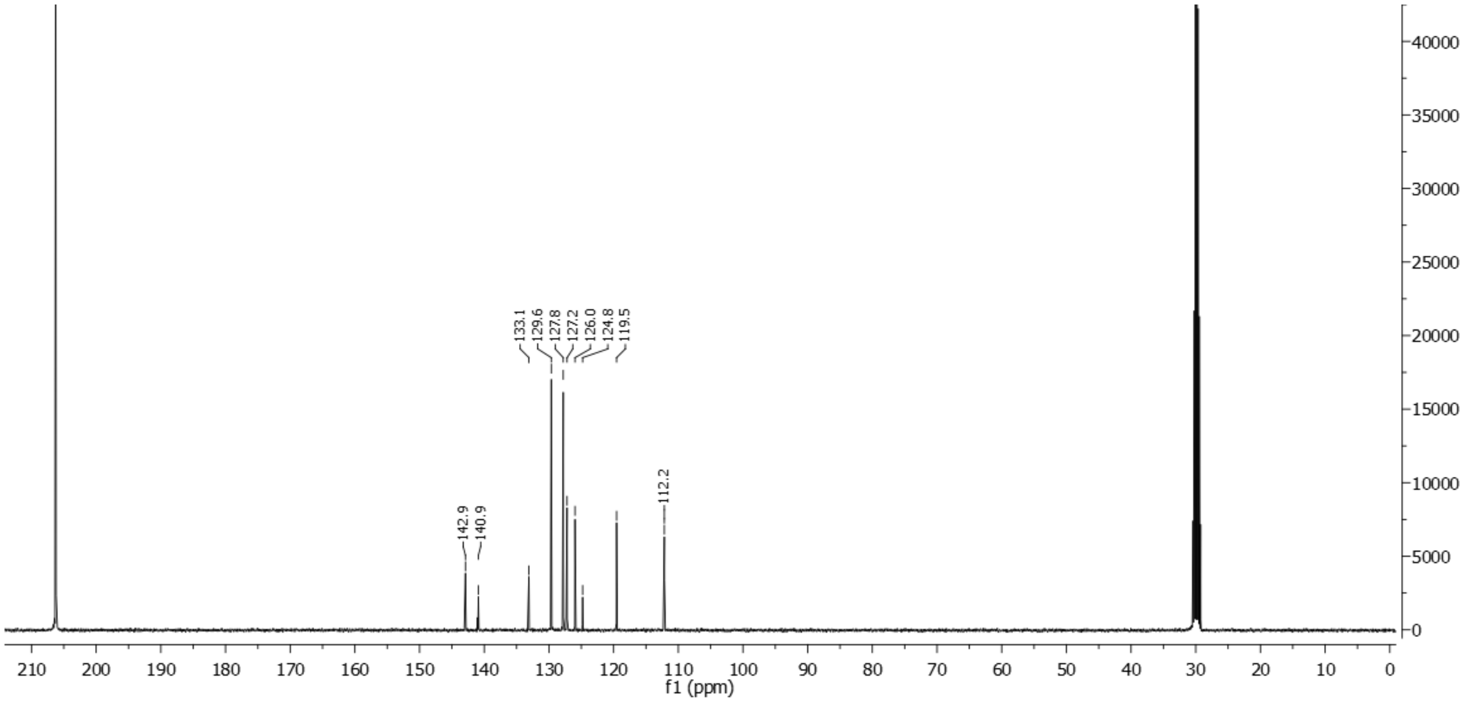

^{13}C-NMR spectrum of the non-deuterated starting material

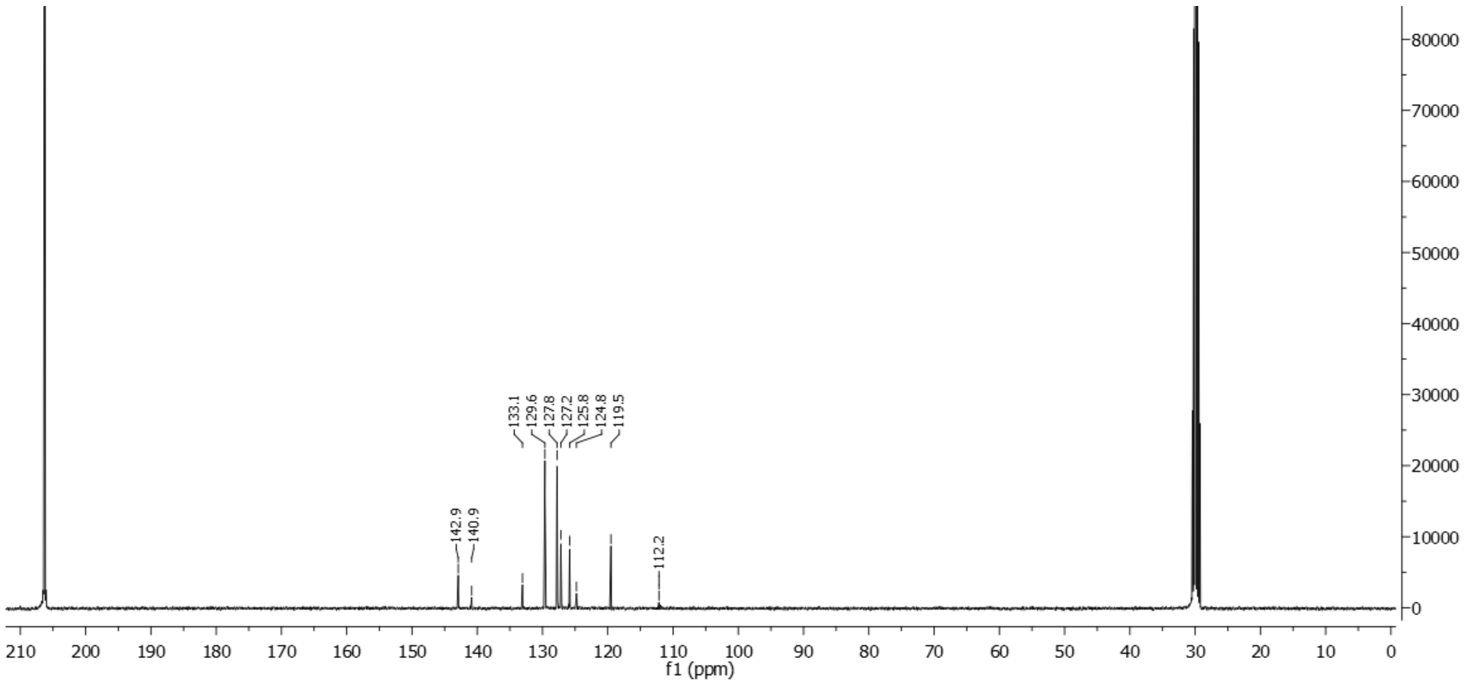

^{13}C-NMR spectrum of **15**'

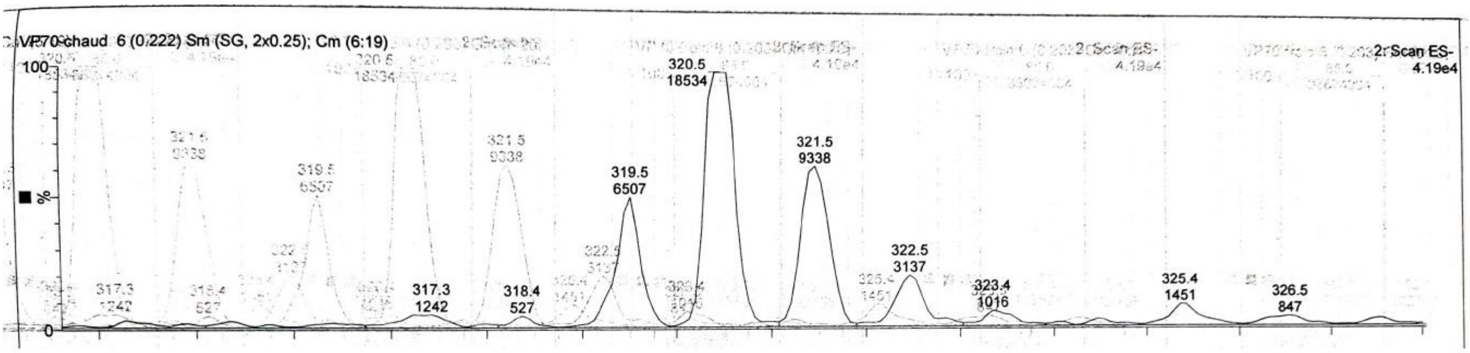

ESI-spectrum of **15’**

11,12-Dihydroindolo[2,3-a]carbazole **16’**

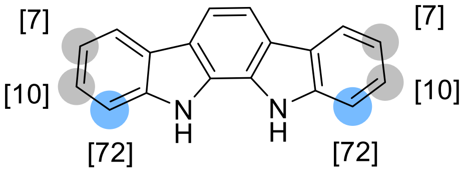

Chemical Formula: $C_{18}H_{12}N_2$

Substrate	Solvent (Volume)	RuNp@PVP cat.
51.2mg, 0.2mmol	THF (2mL)	28.9mg, 10mol%

Workup and purification:

After cooling down to room temperature EE:Cy (1:1, 3mL) was added to the reaction mixture and stirred for 10mins to let precipitate RuNp@PVP. The suspension was passed through a neutral Al_2O_3 pad and the crude product was eluted with THF (5mL). The solvent was removed under vacuum and the crude product was recrystallized from THF and MeOH (THF : MeOH, 10:1).

Yield: 18.0mg, 35%, white solid

^{1}H NMR (400 MHz, Acetone-*d*$_6$): δ 10.41 (bs, NH), 8.19 – 8.14 (m, 2H), 7.98 – 7.94 (m, 2H), 7.63 – 7.59 (m, 0.56H), 7.40 – 7.35 (m, 2H), 7.25 – 7.19 (m, 2H).

Deuterium incorporation was expected at *δ* 7.63 – 7.59. Isotopic enrichment values were determined against the integral at *δ* 8.19 – 8.14.

^{2}H-{^{1}H}NMR (600 MHz, Acetone): δ 7.61 (s, 1.44D), 7.38 (s, 0.20D), 7.23 (s, 0.14D).

^{13}C-{^{1}H}NMR (100 MHz, Acetone-*d*$_6$): δ 140.3, 126.8, 125.5, 125.4, 121.9, 120.5, 120.0, 112.6, 112.1 (m).

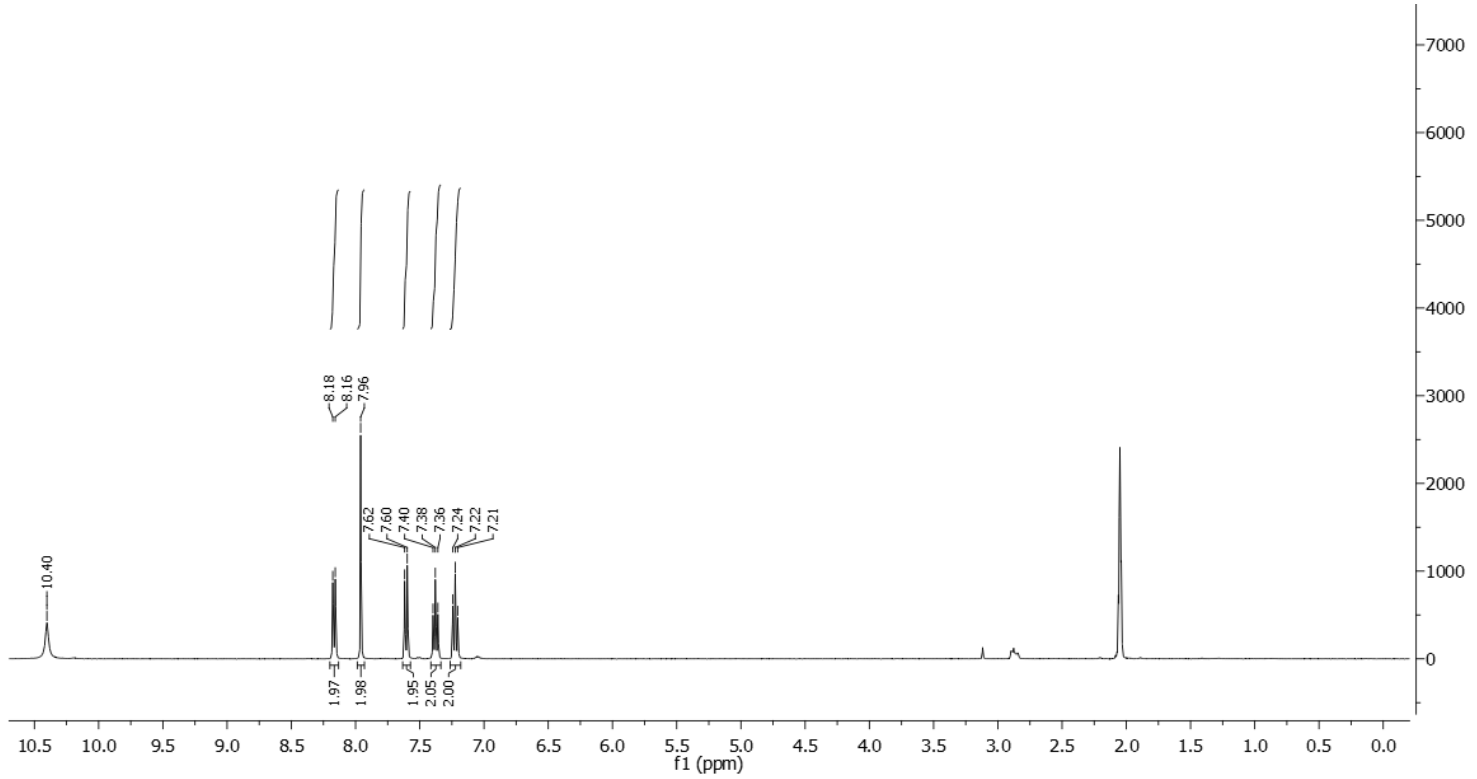

^{1}H-NMR spectrum of the non-deuterated starting material

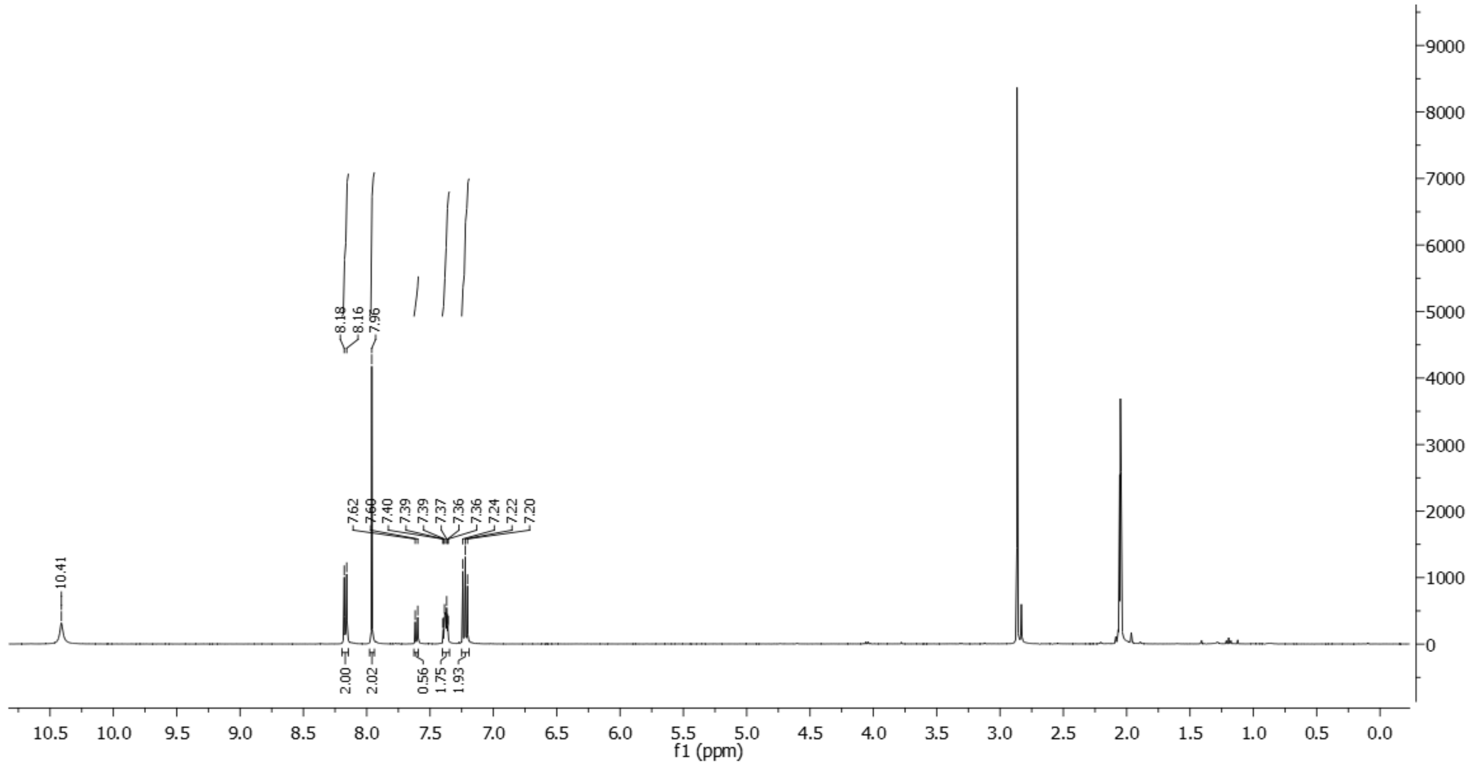

^{1}H-NMR spectrum of **16’**

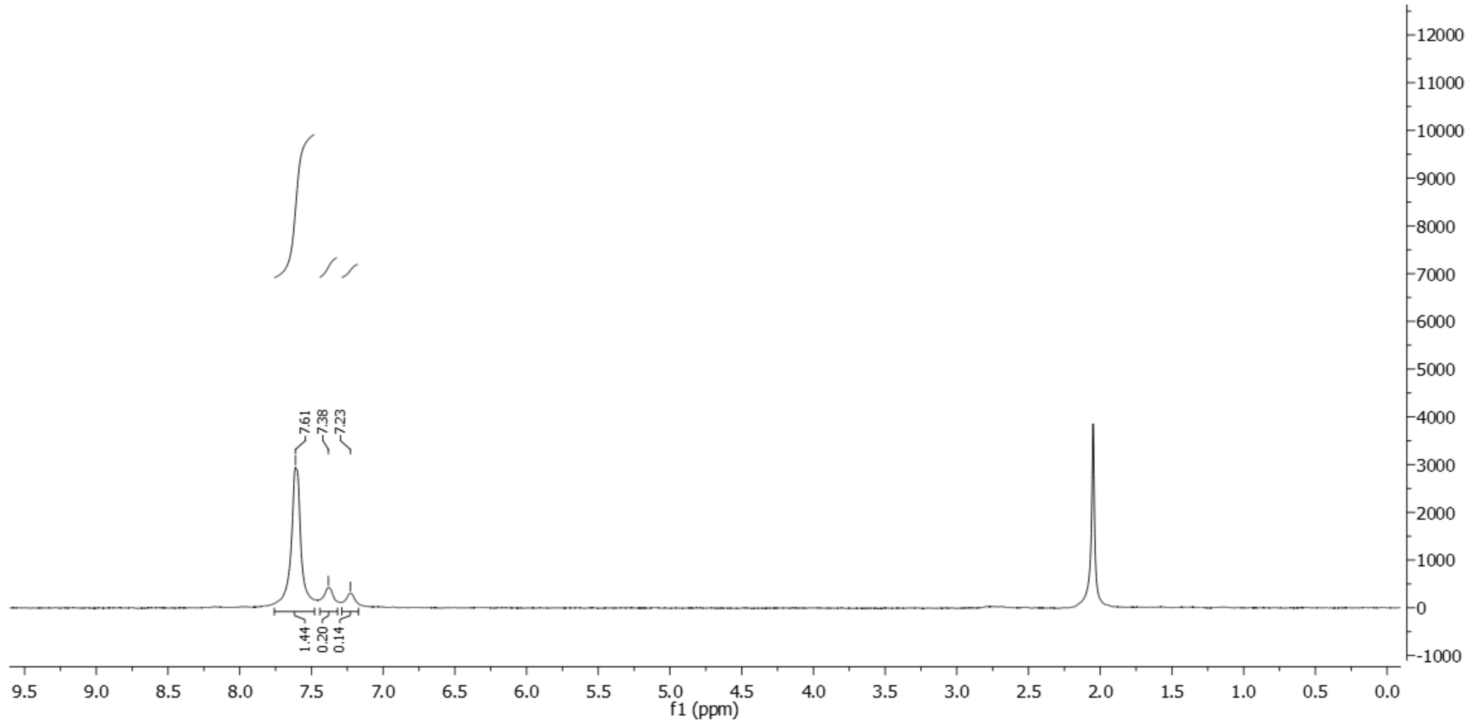

^{2}H-NMR spectrum of **16’**

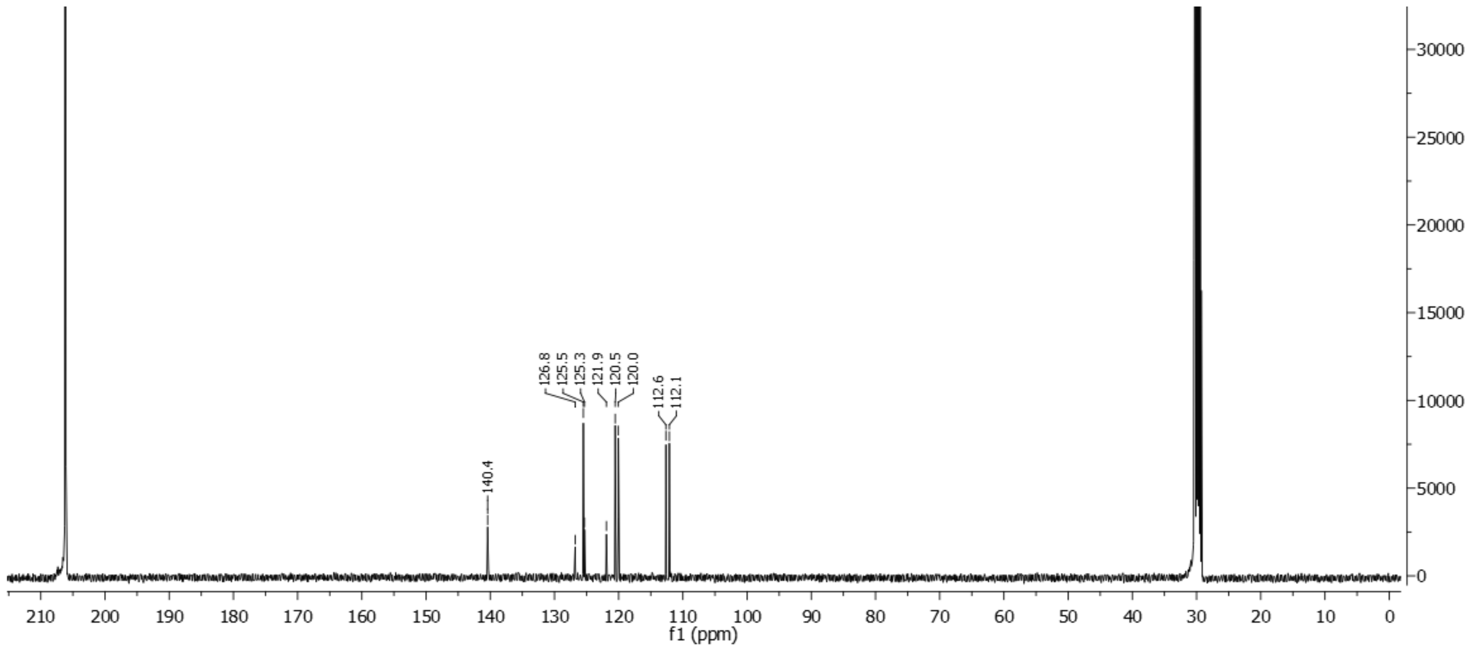

^{13}C-NMR spectrum of the non-deuterated starting material

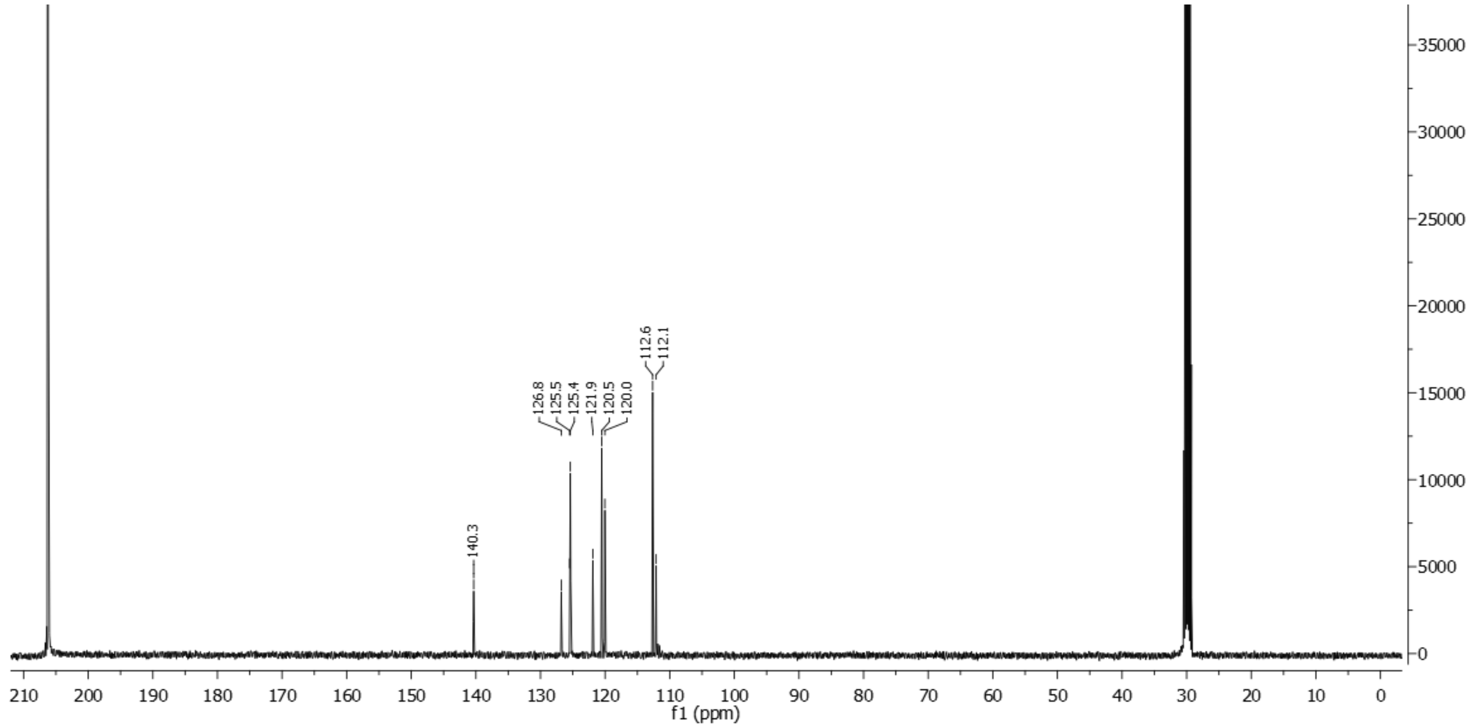

^{13}C-NMR spectrum of **16’**

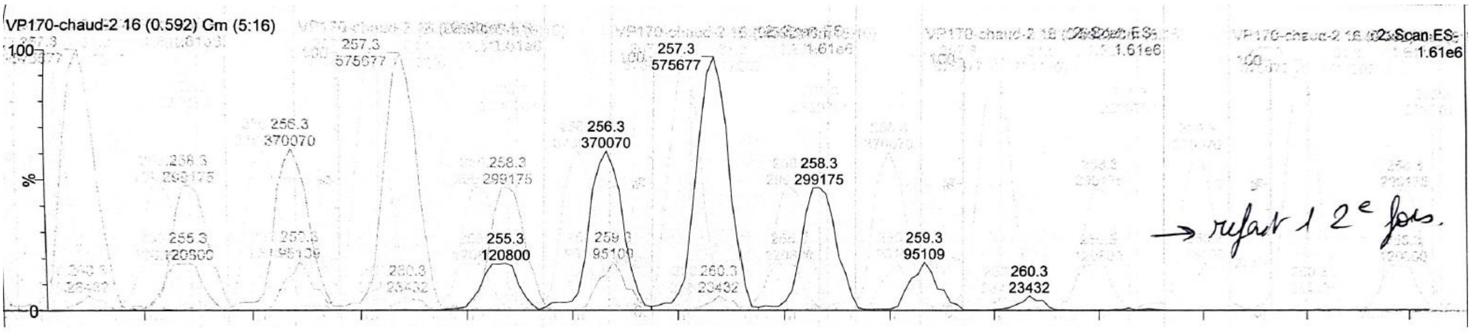

ESI-spectrum of **16’**

Compound	**M_0**	**M_{+1}**	**M_{+2}**	**M_{+3}**	**M_{+4}**	**M_{+5}**	**M_{+6}**	**M_{+7}**	**Total D**
**13’**	13.7%	20.4%	43.8%	22.1%	6.0%	1.1%	0.1%		2.0D
**14’**	11.0%	35.9%	53.1%	1.4%					1.4D
**15’**	7.7%	21.2%	56.0%	15.1%	4.3%	1.7%			2.0D
**16’**	9.7%	30.5%	42.0%	13.5%	3.1%	1.2%			1.7D

Deuterations of Drugs and other bioactive molecules

Pimprinine **17I** (without base)

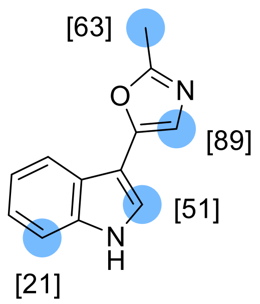

Chemical Formula: $C_{12}H_{10}N_2O$

Substrate	Solvent (Volume)	RuNp@PVP cat.
20.0mg, 0.1mmol	Methanol-d_4 (2mL)	28.9mg, 20mol%

Workup and purification:

After cooling down to room temperature the reaction mixture was poured on H_2O dist. (100mL) in a separation funnel. The aqueous phase was extracted three times with EtOAc (100mL). The solvent was removed under vacuum at room temperature and the crude product was recrystallized from acetone and MeOH (acetone : MeOH, 3:1).

Yield: 4.0mg, 20%, white solid

^{1}H NMR (400 MHz, Methanol-d_4): δ 7.81 – 7.75 (m, 1H), 7.60 (s, 0.38H), 7.46 – 7.41 (m, 0.71H), 7.23 – 7.12 (m, 2.21H), 2.53 – 2.48 (m, 1.08H).

Deuterium incorporation was expected at *δ* 7.60, *δ* 7.46 – 7.41, *δ* 7.23 – 7.12 and at *δ* 2.53 – 2.48. Isotopic enrichment values were determined against the integral at *δ* 7.81 – 7.75.

^{2}H-{^{1}H}NMR (92 MHz, Methanol): δ 7.58 (s, 0.51D), 7.42 (s, 0.21D), 7.13 (s, 0.89D), 2.49 – 2.41 (m, 1.89D).

^{13}C-{^{1}H}NMR (100 MHz, Methanol-d_4): δ 160.8, 150.0, 138.2, 125.3, 123.6, 123.4 (m), 121.3, 120.4, 119.2 (m), 112.8, 105.5 (m), 13.5 (m).

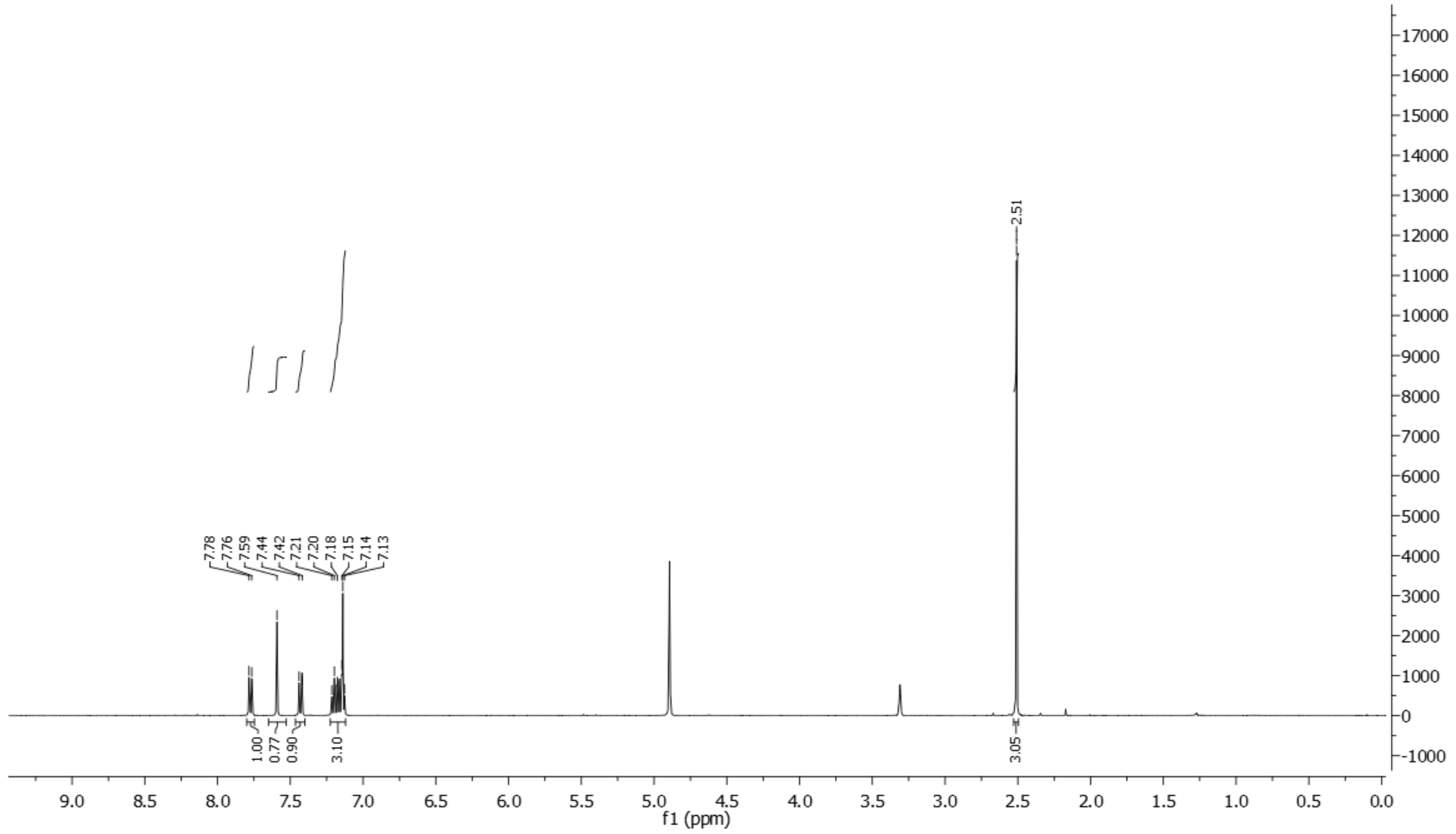

^{1}H-NMR spectrum of the non-deuterated starting material

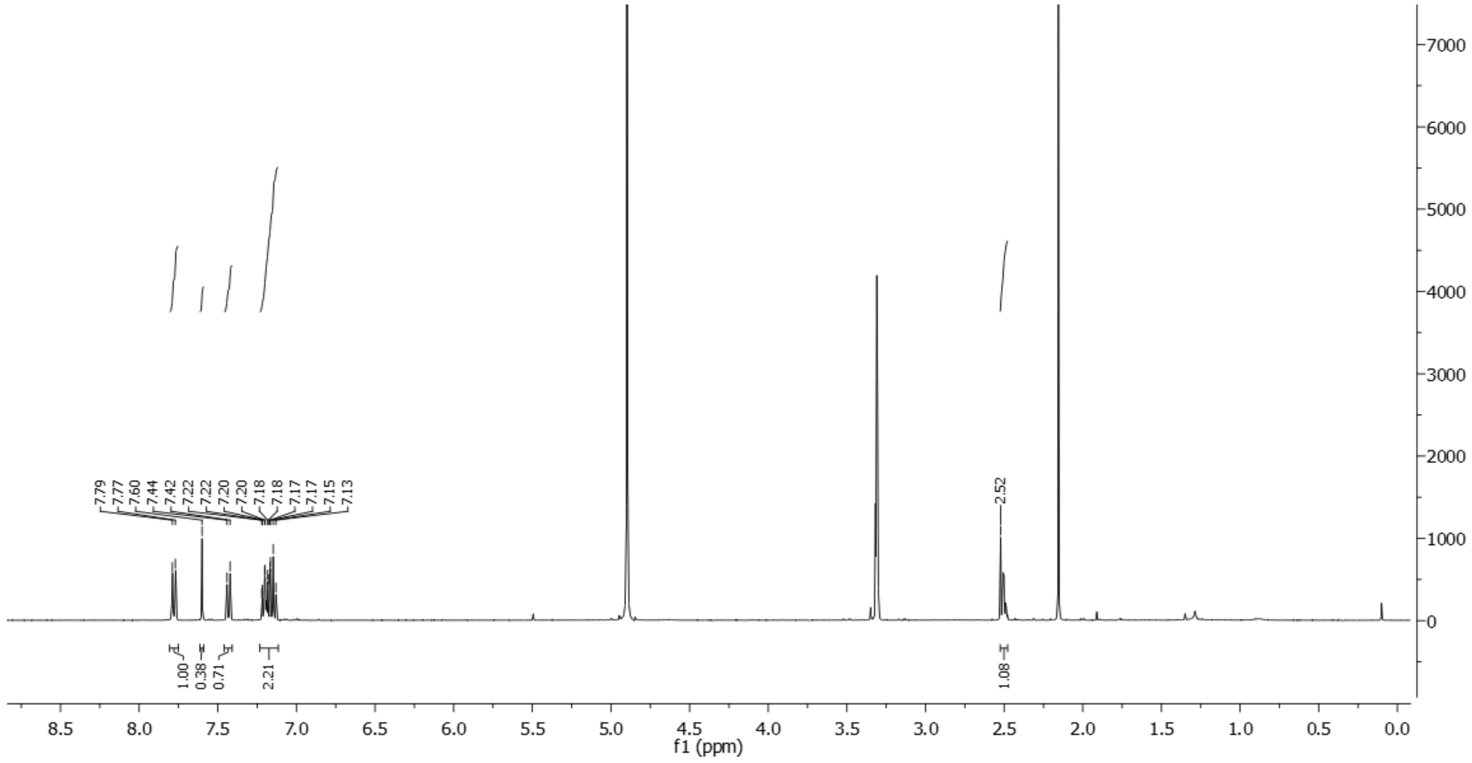

^{1}H-NMR spectrum of **17I**

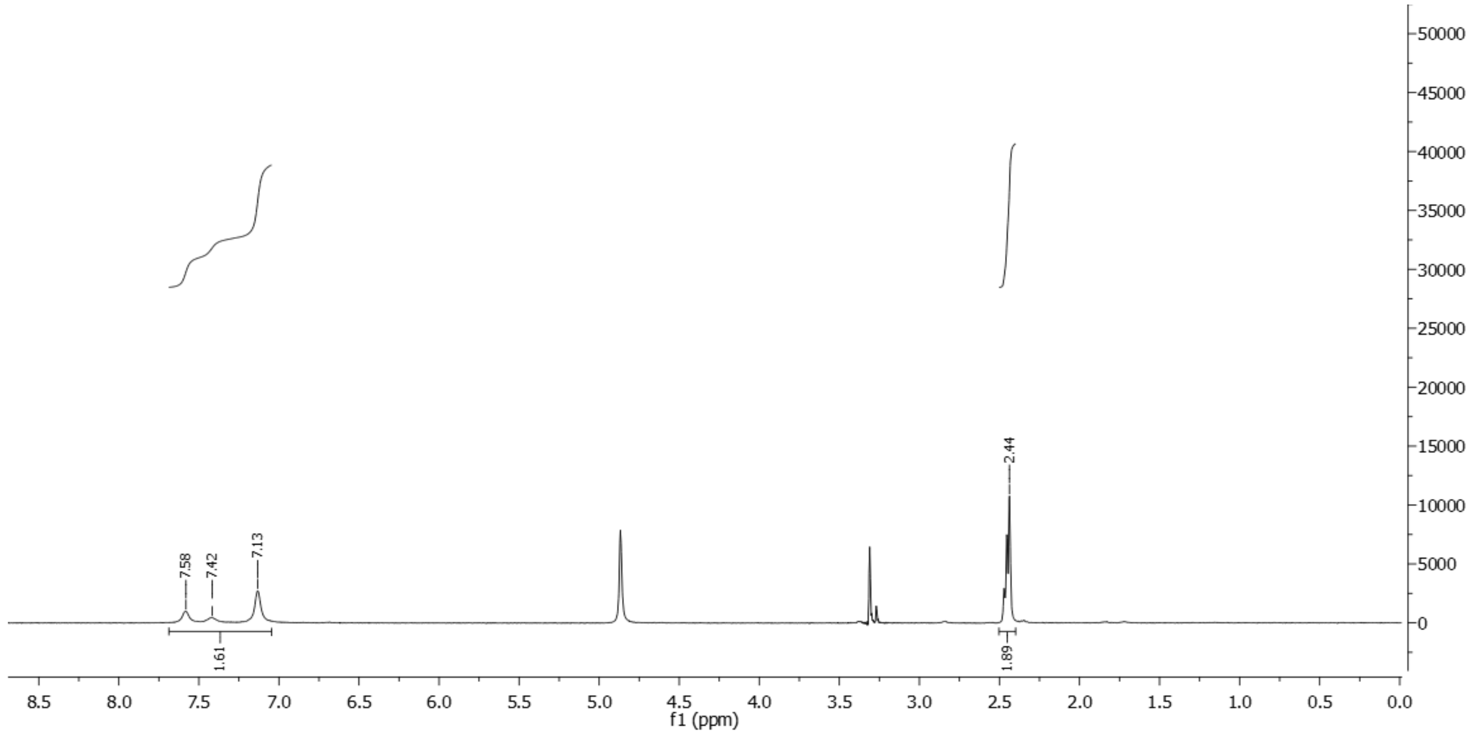

^{2}H-NMR spectrum of **17I**

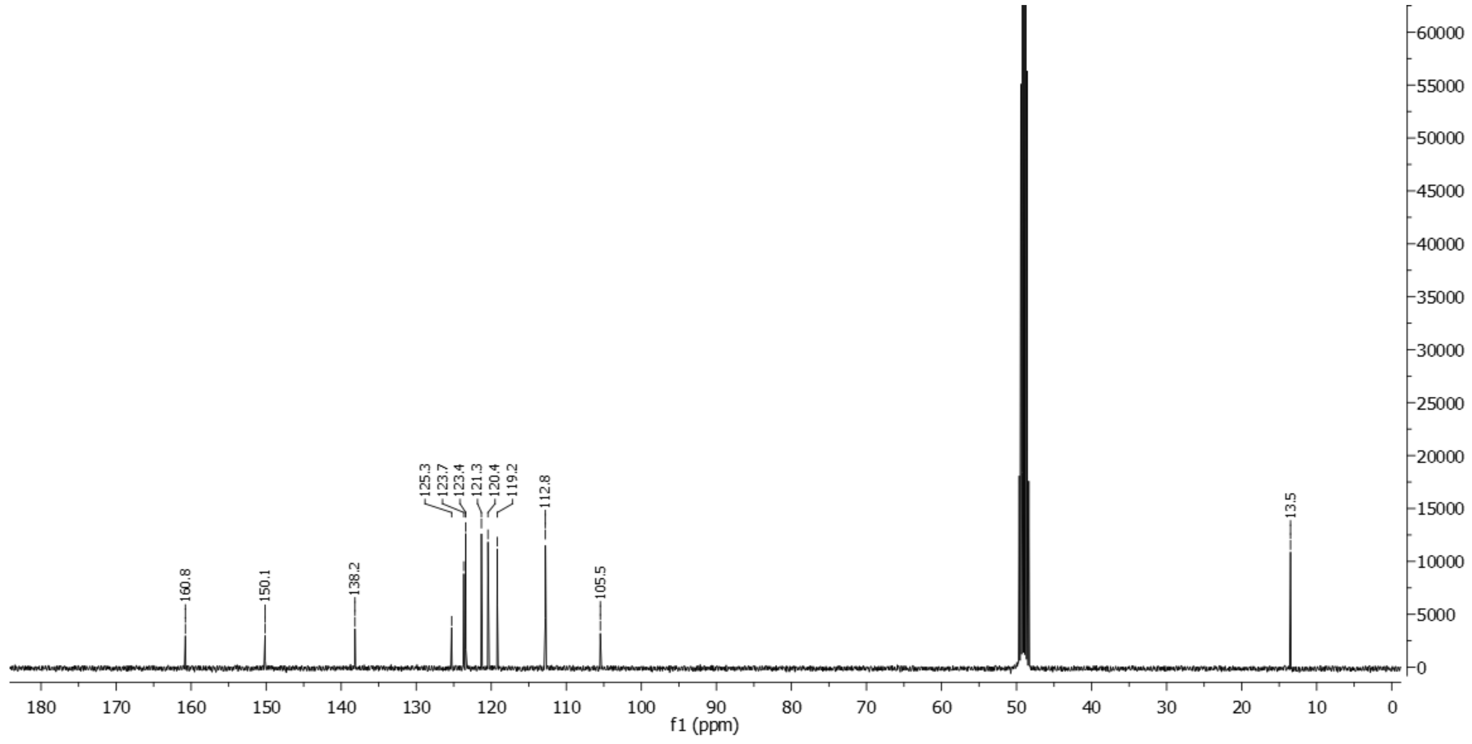

^{13}C-NMR spectrum of the non-deuterated starting material

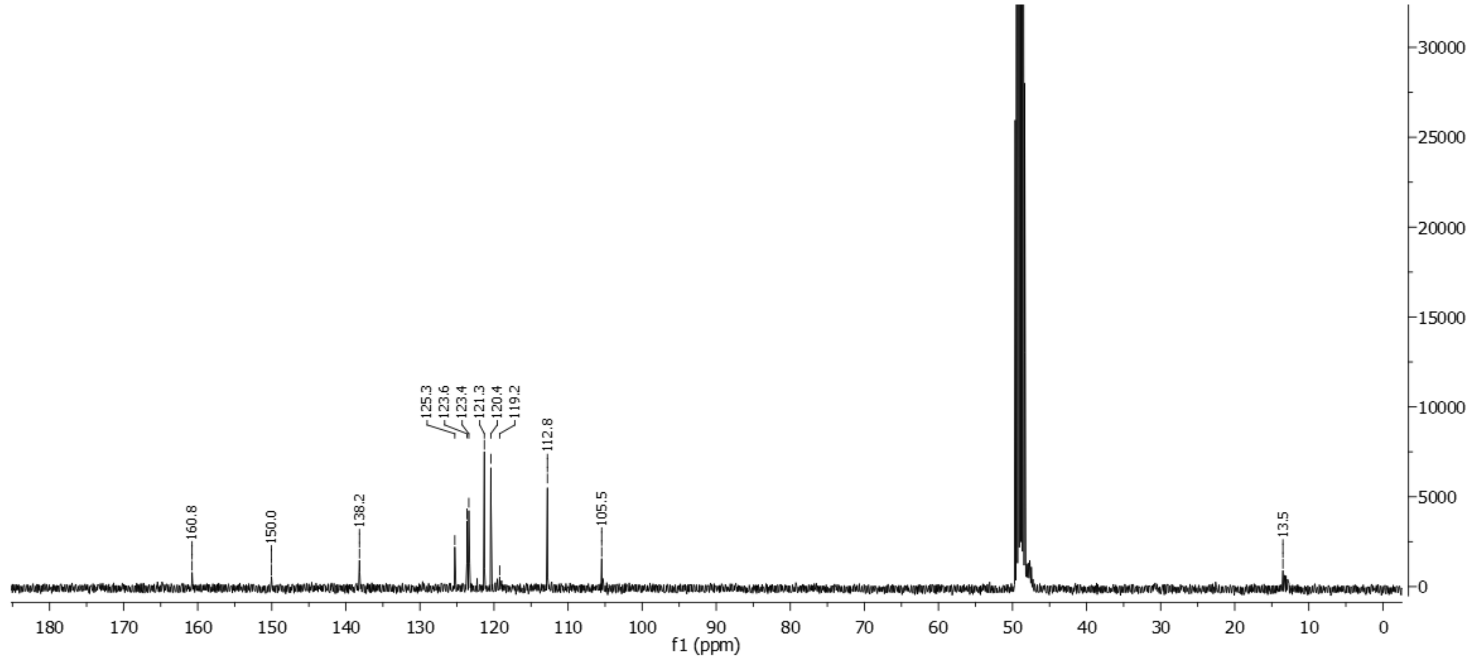

^{13}C-NMR spectrum of **17I**

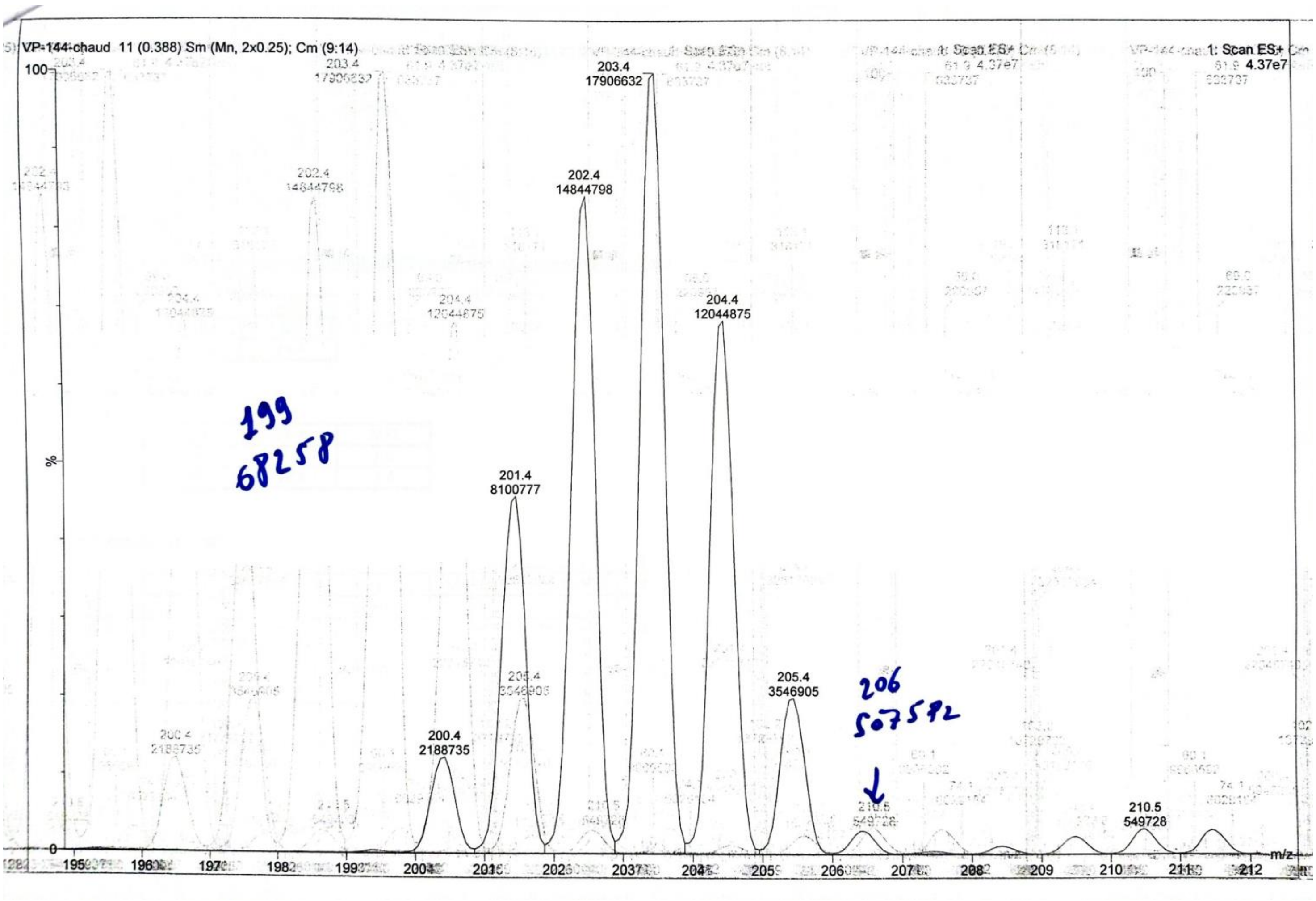

ESI-spectrum of **17I**

Pimprinine **17II** (with base)

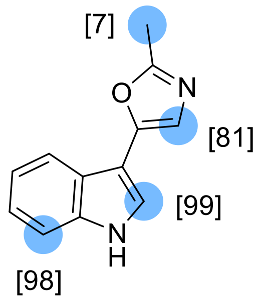

Chemical Formula: $C_{12}H_{10}N_2O$

Substrate	Cs_2CO_3	Solvent (Volume)	RuNp@PVP cat.
20.0mg, 0.1mmol	32.6mg, 0.1mmol	Methanol-*d*$_4$ (2mL)	28.9mg, 20mol%

Workup and purification:

After cooling down to room temperature the reaction mixture was poured on a 5mM solution of acetic acid in H_2O dist. (150mL). The aqueous phase was extracted three times with EtOAc (3 x 50mL) in a separation funnel. The solvent was removed under vacuum and the crude product was purified over SiO_2. Deuterium labelled pimprinine could be eluted at Cy/EtOAc (1:4).

Yield: 3.0mg, 15%, white solid

^{1}H NMR (400 MHz, Methanol-*d*$_4$): δ 7.79 – 7.75 (m, 1H), 7.59 (s, 0.01H), 7.45 – 7.41 (m, 0.02H), 7.23 – 7.11 (m, 2.19H), 2.53 – 2.49 (m, 2.80H).

Deuterium incorporation was expected at δ 7.59, δ 7.45 – 7.41, δ 7.23 – 7.11 and at δ 2.53 – 2.49. Isotopic enrichment values were determined against the integral at δ 7.79 – 7.75.

^{2}H-{^{1}H}NMR (92 MHz, Methanol): δ 7.58 (s, 1D), 7.43 (s, 1D), 7.13 (s, 0.81D), 2.49 – 2.41 (m, 0.28D).

^{13}C-{^{1}H}NMR (100 MHz, Methanol-*d*$_4$): δ 160.8, 150.0, 138.1, 125.3, 123.5(m), 123.3, 121.3, 120.4, 119.0 (m), 112.8, 105.3 (m), 13.5 (m).

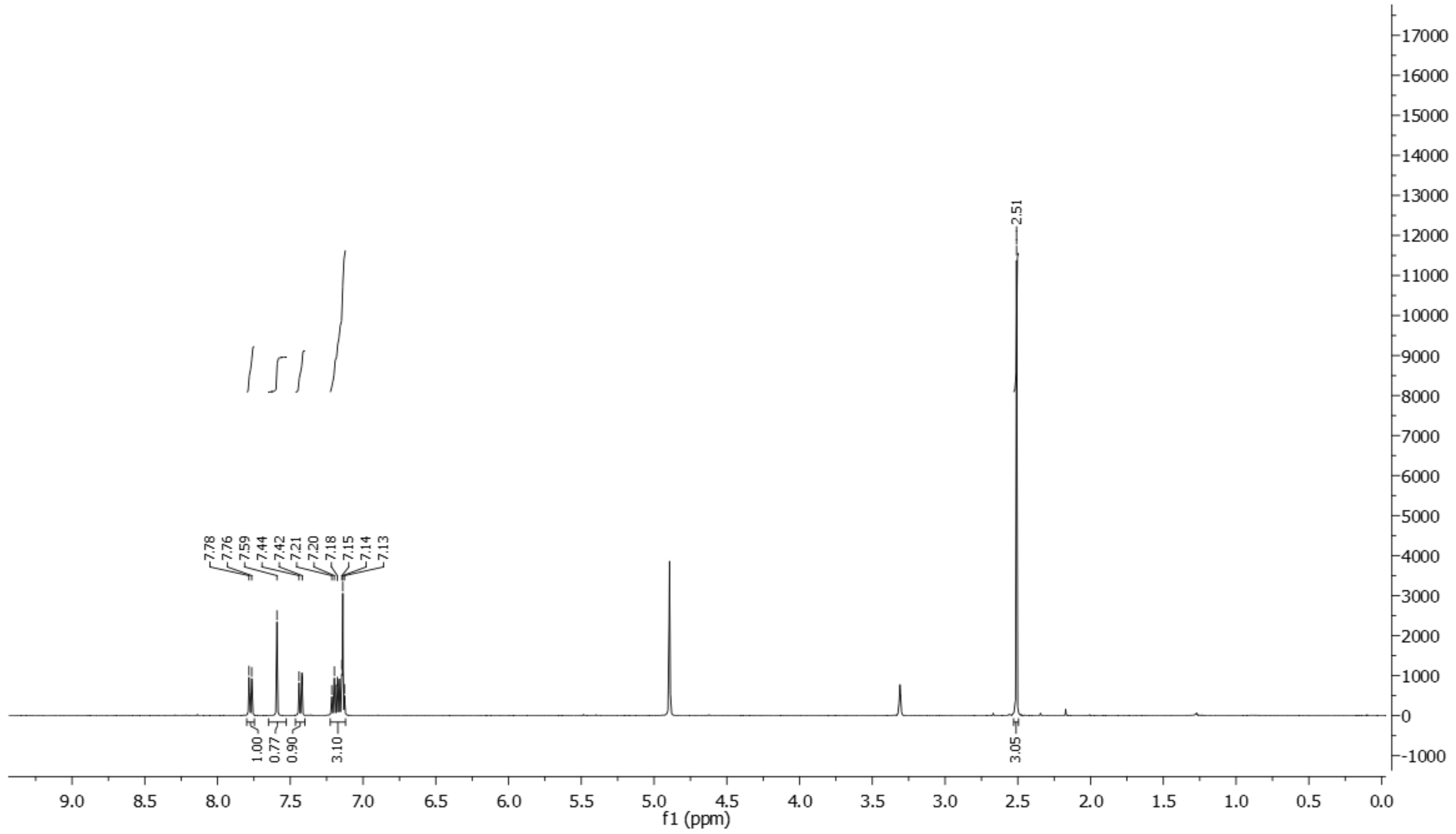

^{1}H-NMR spectrum of the non-deuterated starting material

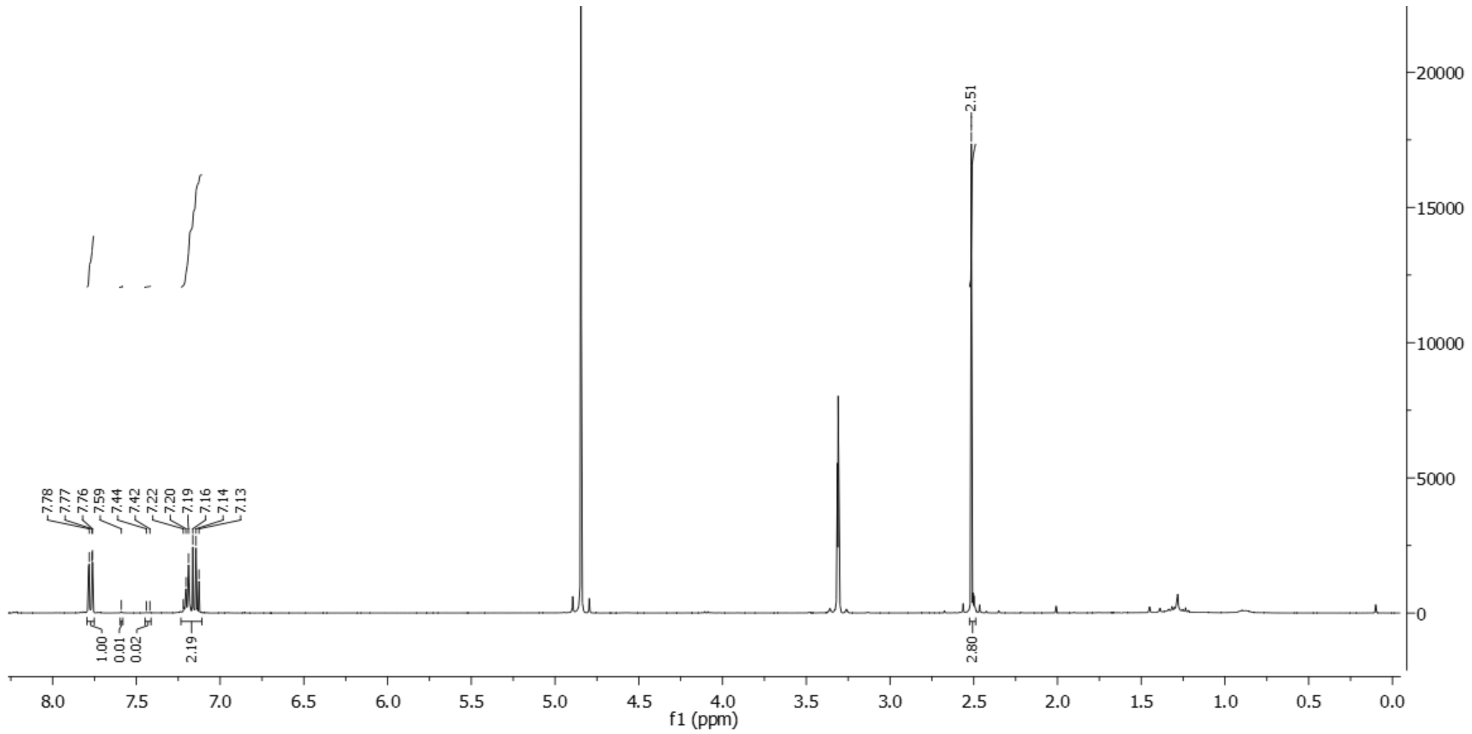

^{1}H-NMR spectrum of **17II**

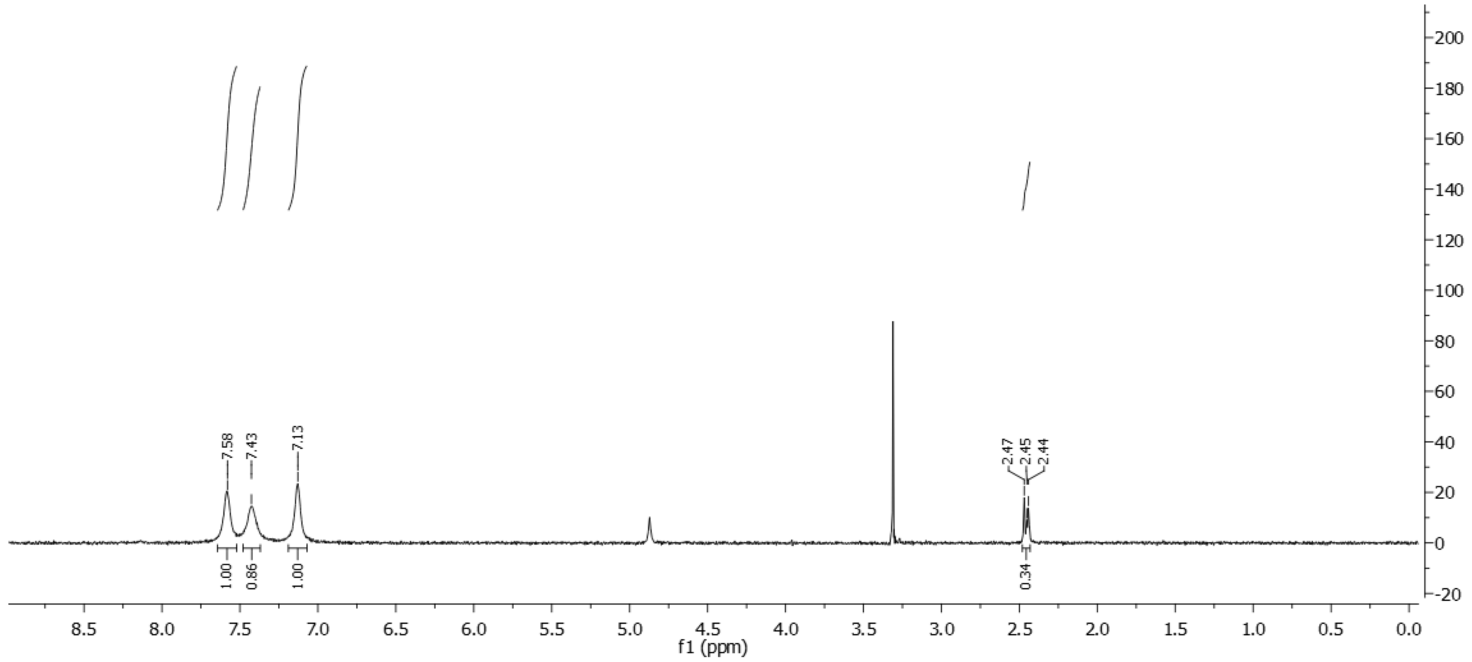

^{2}H-NMR spectrum of **17II**

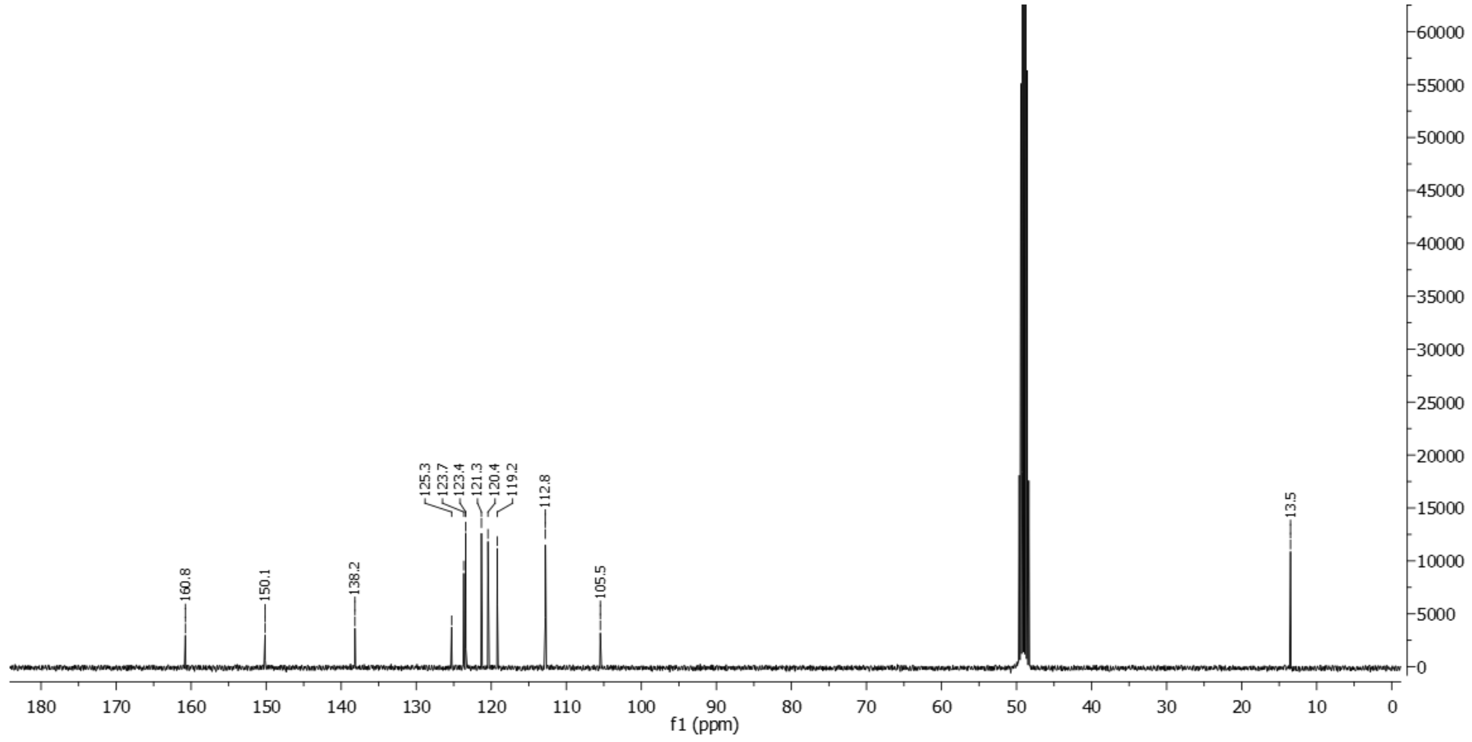

^{13}C-NMR spectrum of the non-deuterated starting material

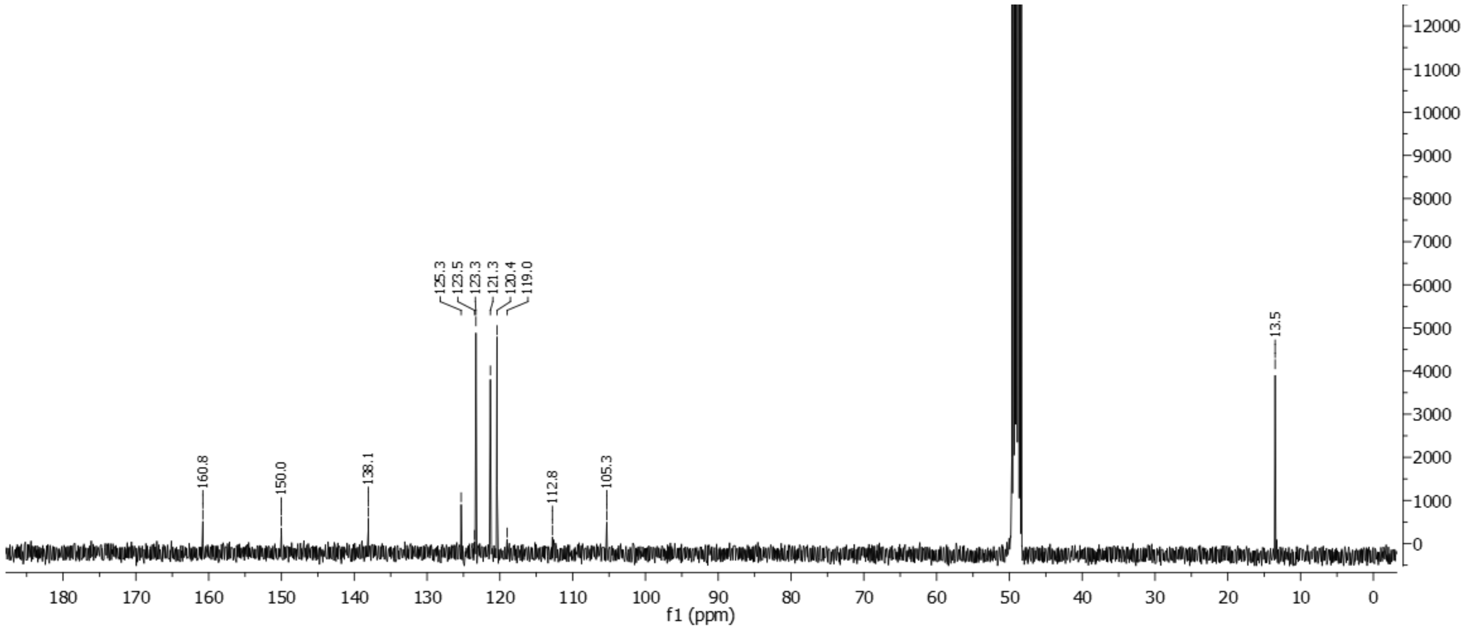

^{13}C-NMR spectrum of **17II**

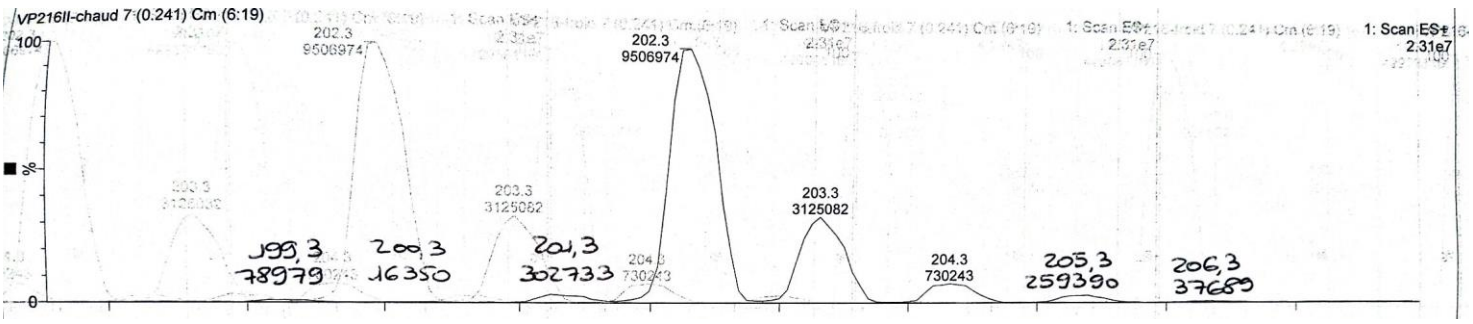

ESI-spectrum of **17II**

Astemizole **18**

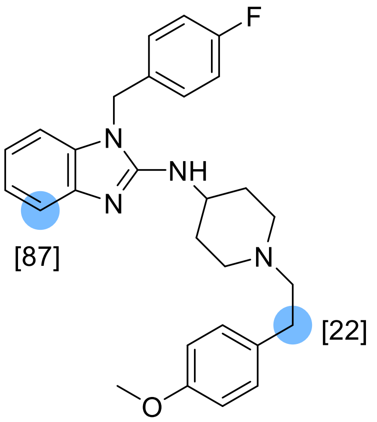

Chemical Formula: $C_{28}H_{31}FN_4O$

Substrate	Solvent (Volume)	RuNp@PVP cat.
45.9mg, 0.1mmol	THF (2mL)	28.9mg, 20mol%

Workup and purification:

After cooling down to room temperature, EtOAc /Cy (1:1, 3mL) was added to the reaction mixture and stirred for 10min to let precipitate RuNp@PVP. The suspension was passed through a Sep-Pak® C18 cartridge and then eluted with EtOAc (5mL). The solvent was removed under vacuum to give 42mg of crude product. 10mg of the crude were purified over basic Al_2O_3. Elution started with Cy/ EtOAc (3:1). Pure product was eluted with THF/MeOH (1:1).

Yield: 6.0mg, 60%, white solid

^{1}H NMR (400 MHz, Acetone-*d$_6$*): δ 7.29 – 7.24 (m, 0.13H), 7.24 – 7.02 (m, 7H), 7.01 – 6.95 (m, 1H), 6.91 – 6.80 (m, 3H), 5.76 (d, J = 7.5 Hz, 1H), 5.28 (s, 2H), 3.97 – 3.85 (m, 1H), 3.75 (s, 3H), 2.97 – 2.90 (m, 2H), 2.73 – 2.66 (m, 1.84H), 2.54 – 2.49 (m, 1.54H), 2.20 – 2.06 (m, 4H), 1.62 – 1.50 (m, 2H).

Deuterium incorporation was expected at *δ* 7.29 – 7.24, *δ* 2.73 – 2.66 and at *δ* 2.54 – 2.49. Isotopic enrichment values were determined against the integral at *δ* 7.01 – 6.95.

^{2}H-{^{1}H}NMR (92 MHz, Acetone): δ 7.28 (s, 0.87D), 2.64 (s, 0.16D), 2.47 (s, 0.46D).

^{13}C-{^{1}H}NMR (100 MHz, Acetone-*d$_6$*): δ 158.9, 154.8, 144.2, 135.6, 134.1, 133.6, 130.4, 129.6, 121.5, 119.5, 116.5 (m), 116.3, 116.1, 114.4, 108.4, 61.4, 55.4, 53.4, 51.4, 44.9, 33.6 (m), 33.3.

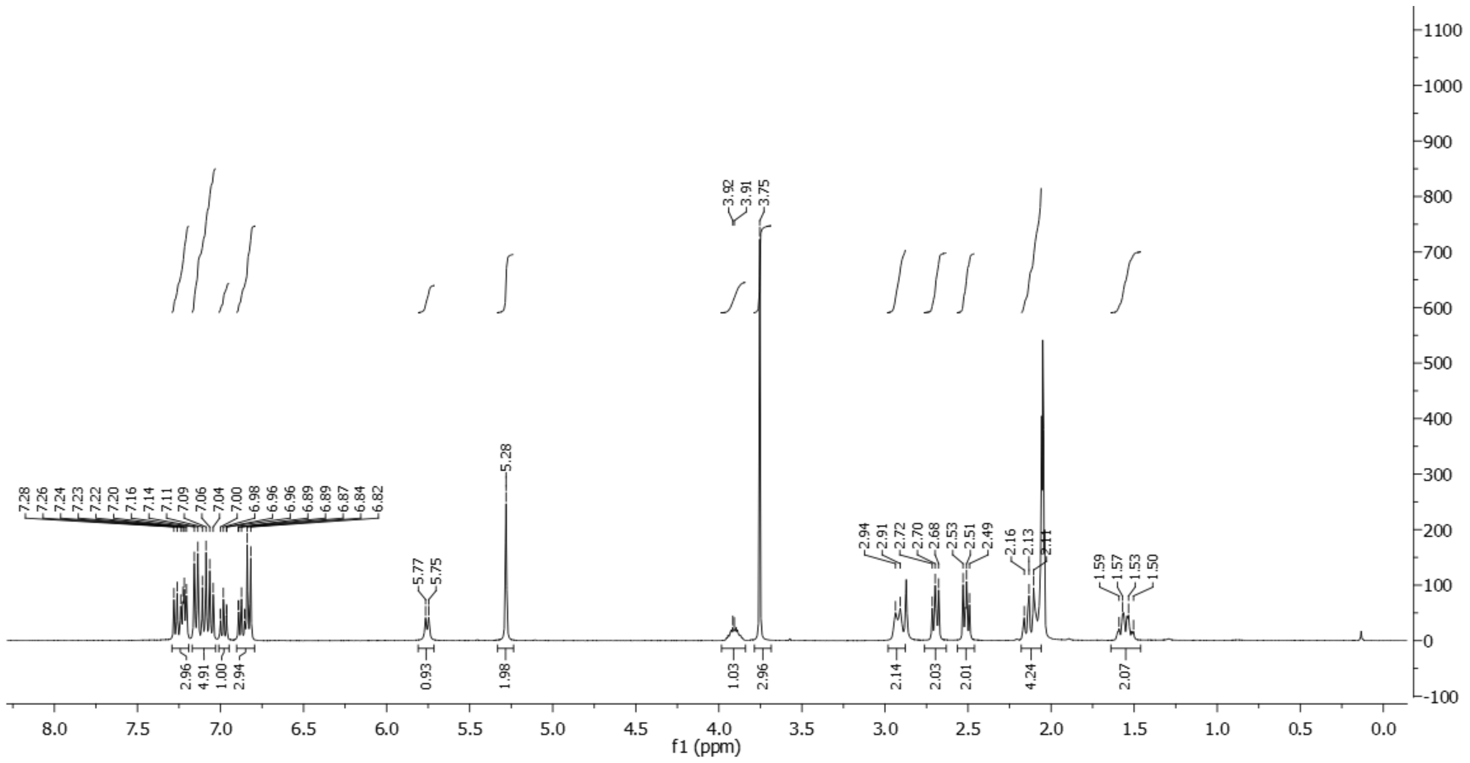

^{1}H-NMR spectrum of the non-deuterated starting material

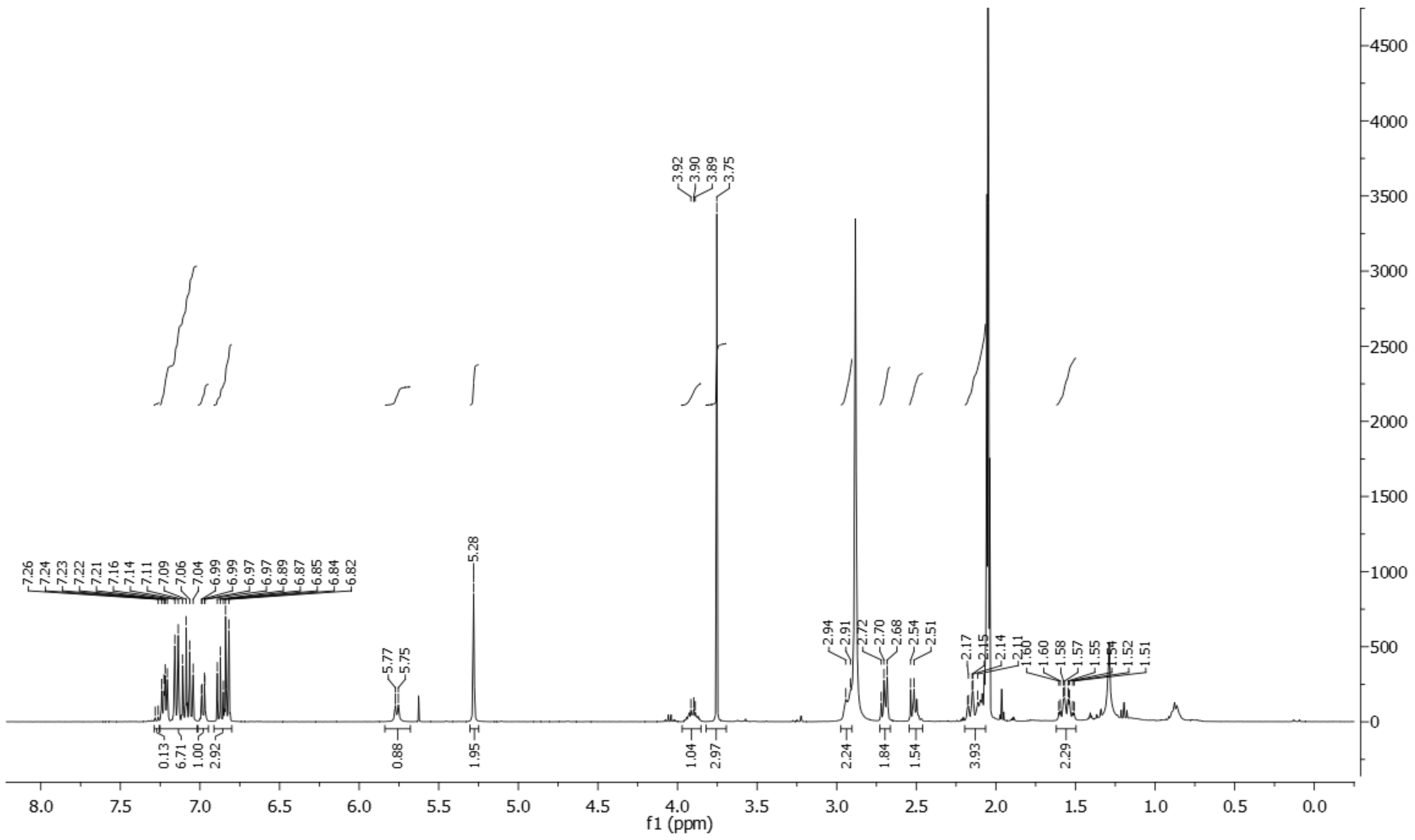

^{1}H-NMR spectrum of **18**

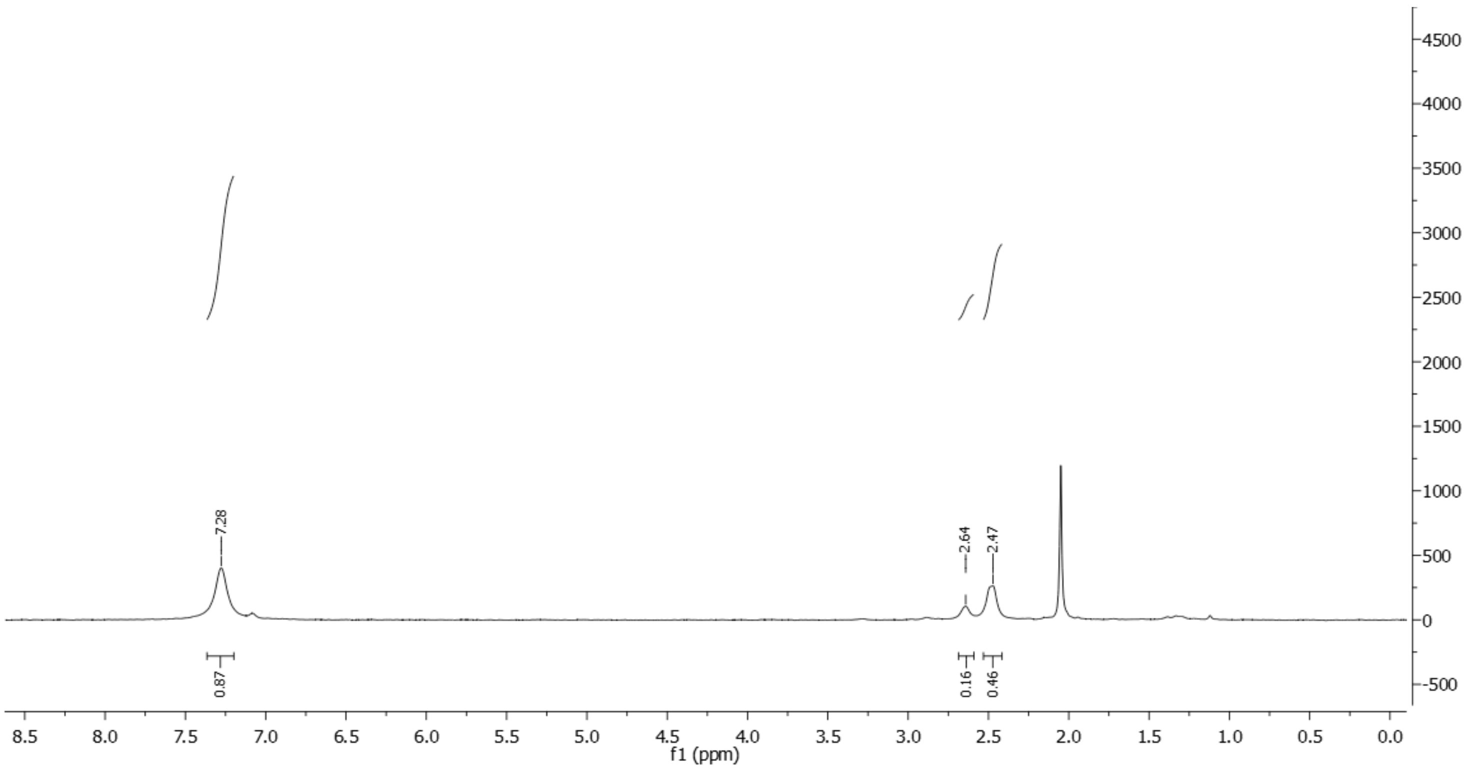

^{2}H-NMR spectrum of **18**

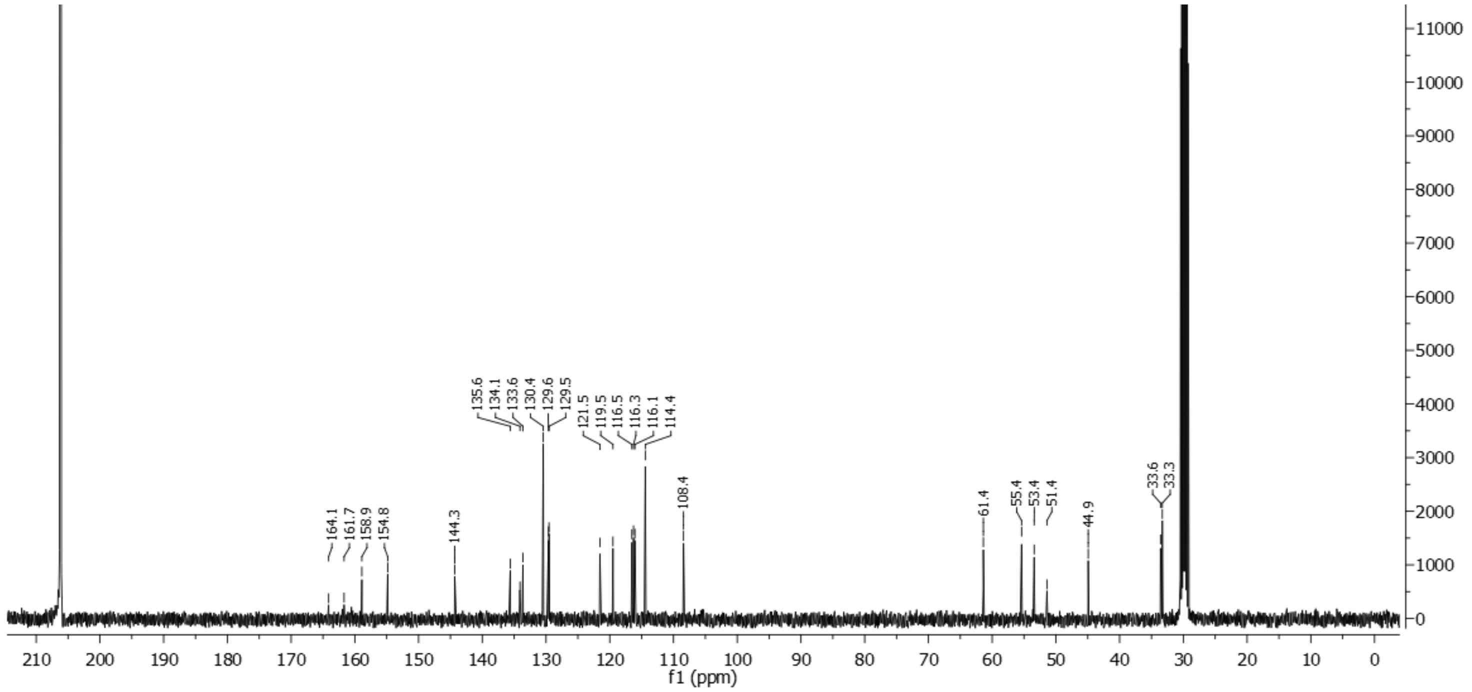

^{13}C-NMR spectrum of the non-deuterated starting material

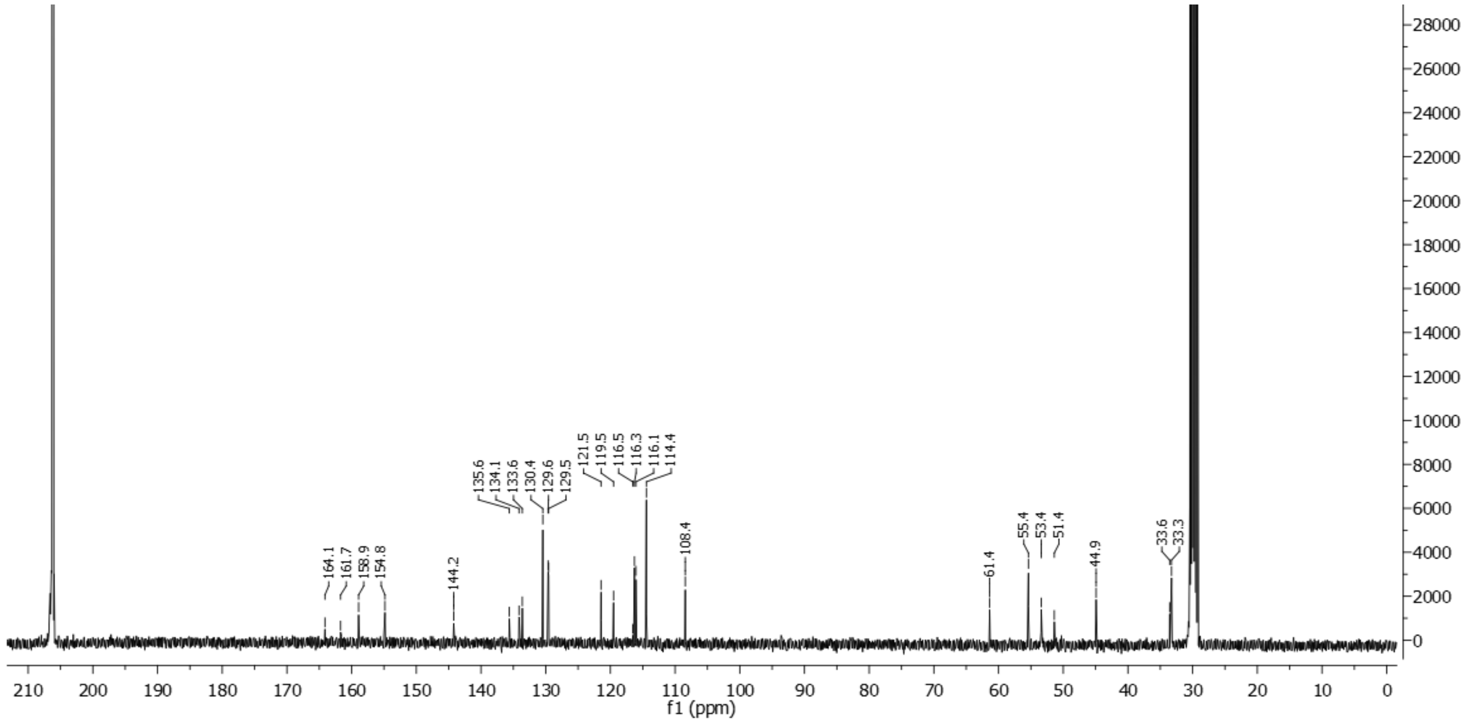

^{13}C-NMR spectrum of **18**

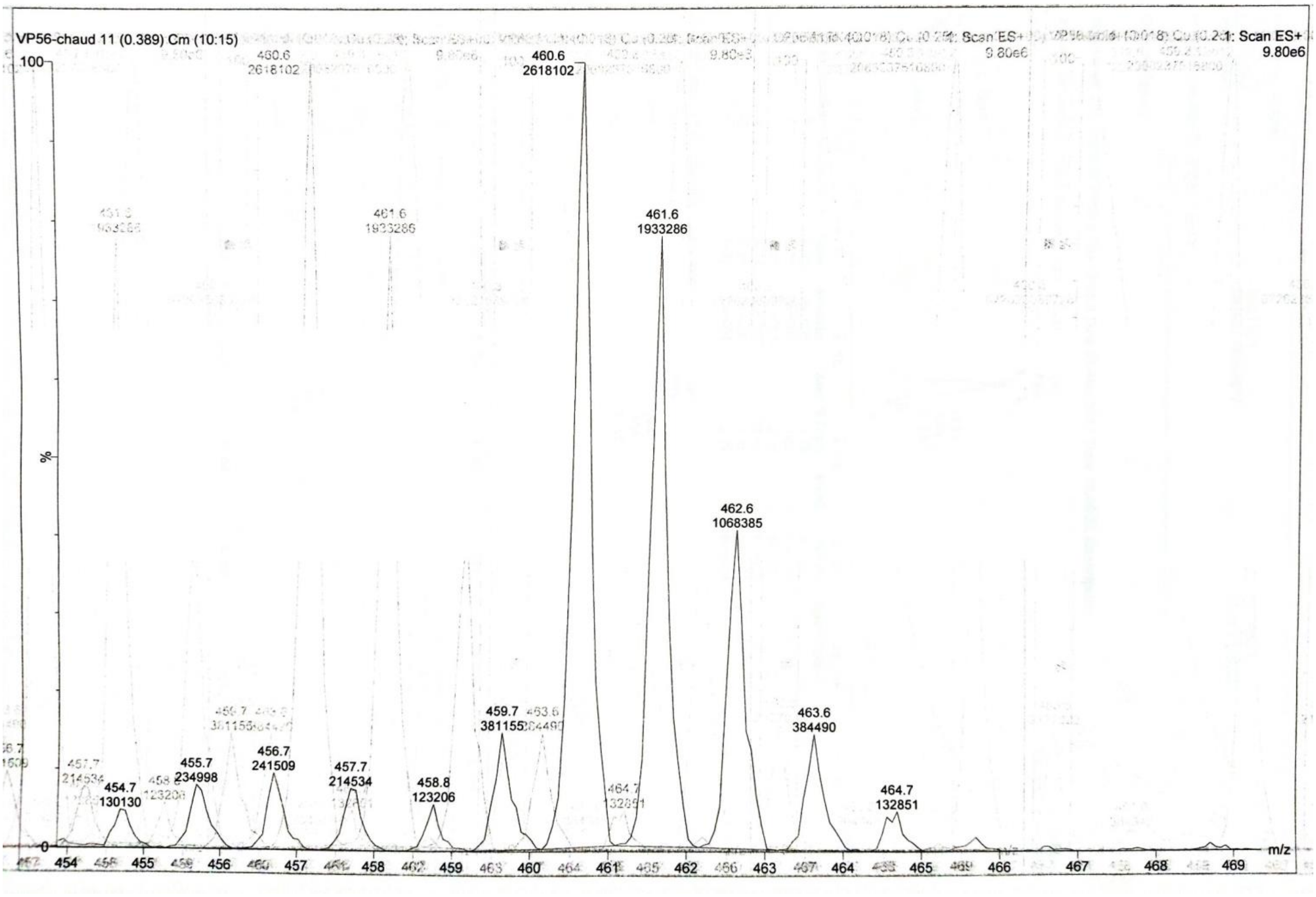

ESI-spectrum of **18**

Imiquimod **19**

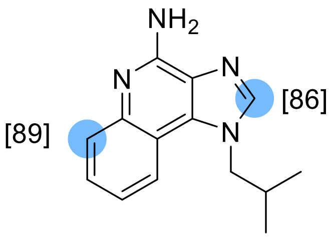

Chemical Formula: $C_{14}H_{16}N_4$

Substrate	Solvent (Volume)	RuNp@PVP cat.
10.0mg, 42µmol	DMA (2mL)	14.4mg, 24mol%

Workup and purification:

After cooling down to room temperature the reaction mixture was poured on H_2O dist. (100mL) in a separation funnel. The aqueous phase was extracted three times with EtOAc (50mL). The solvent was removed under vacuum at room temperature and the crude product was recrystallized from dichloromethane and methanol (DCM : MeOH, 4:1).

Yield: 8.0mg, 80%, white solid

^{1}H NMR (400 MHz, DMSO-*d$_6$*): δ 8.18 (s, 0.18H), 8.03 – 7.97 (m, 1H), 7.63 – 7.59 (m, 0.25H), 7.47 – 7.40 (m, 1H), 7.30 – 7.23 (m, 1H), 6.59 (s, 2H), 4.40 (d, *J* = 7.5 Hz, 2H), 2.21 – 2.13 (m, 1H), 0.91 (d, *J* = 6.6 Hz, 6H).

Deuterium incorporation was expected at *δ* 8.18 and at *δ* 7.63 – 7.59. Isotopic enrichment values were determined against the integral at *δ* 7.30 – 7.23.

^{2}H-{^{1}H}NMR (92 MHz, DMSO): δ 8.17 (s, 0.86D), 7.61 (s, 0.89D).

The low solubility of the compound imiquimod did not allow to record ^{13}C-{^{1}H}NMR spectra.

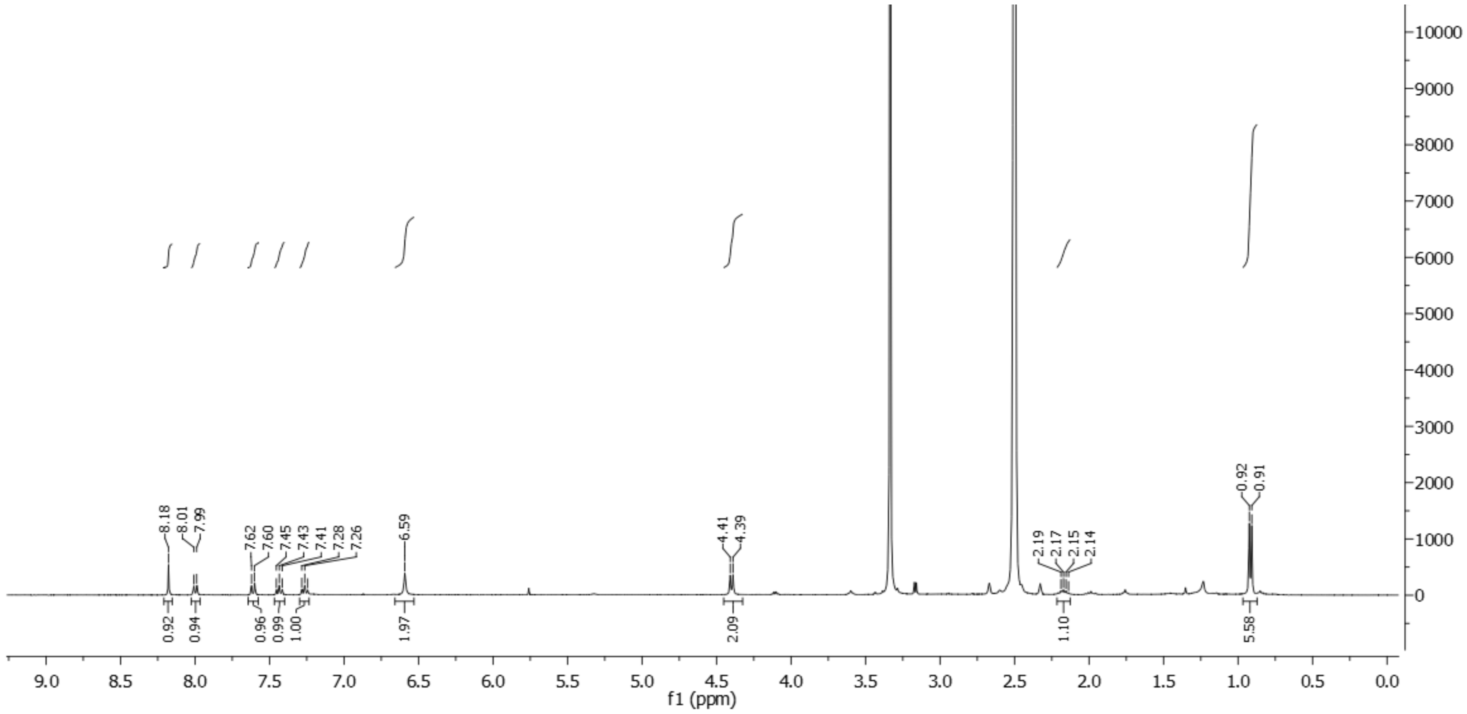

^{1}H-NMR spectrum of the non-deuterated starting material

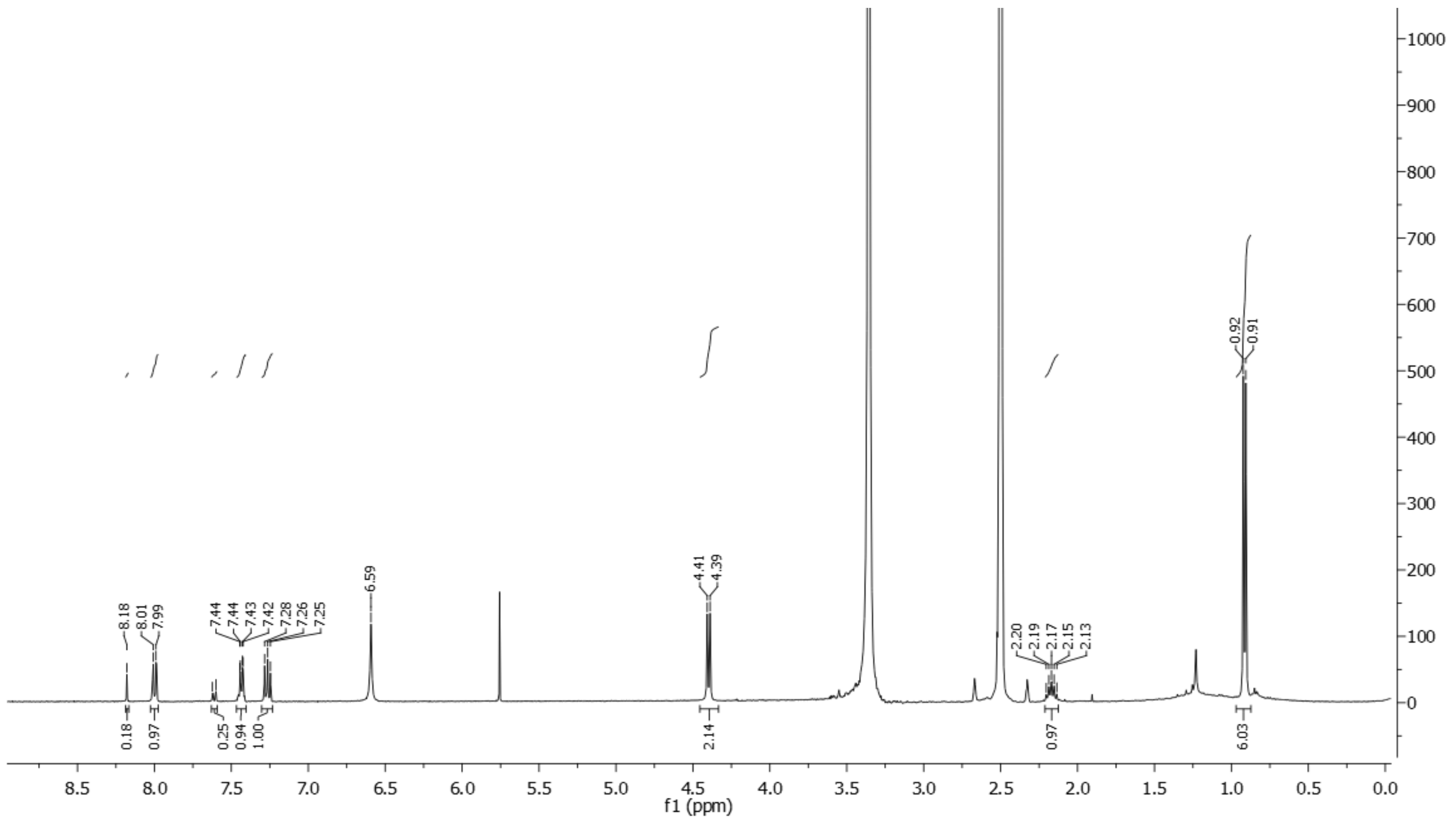

^{1}H-NMR spectrum of **19**

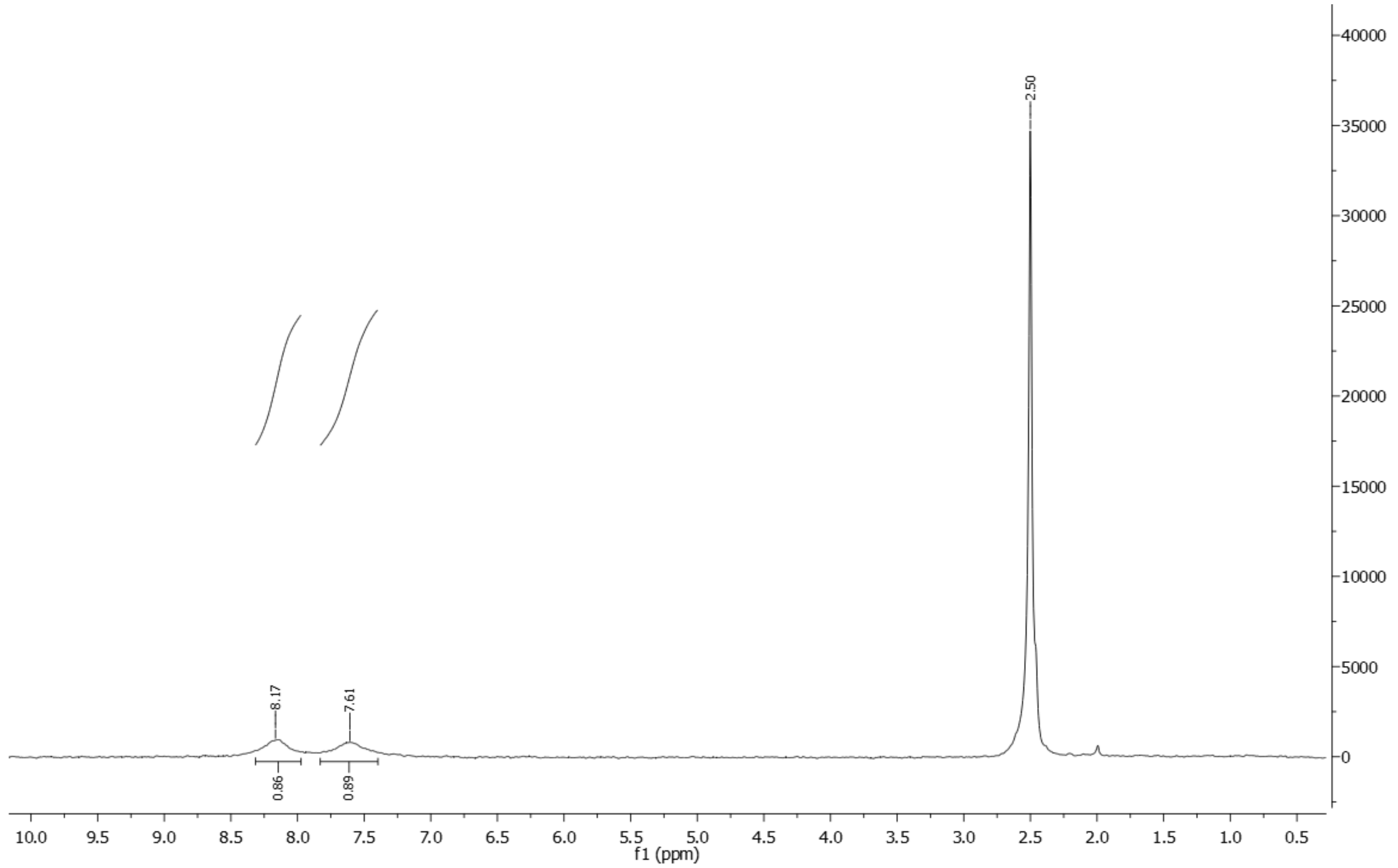

^{2}H-NMR spectrum of **19**

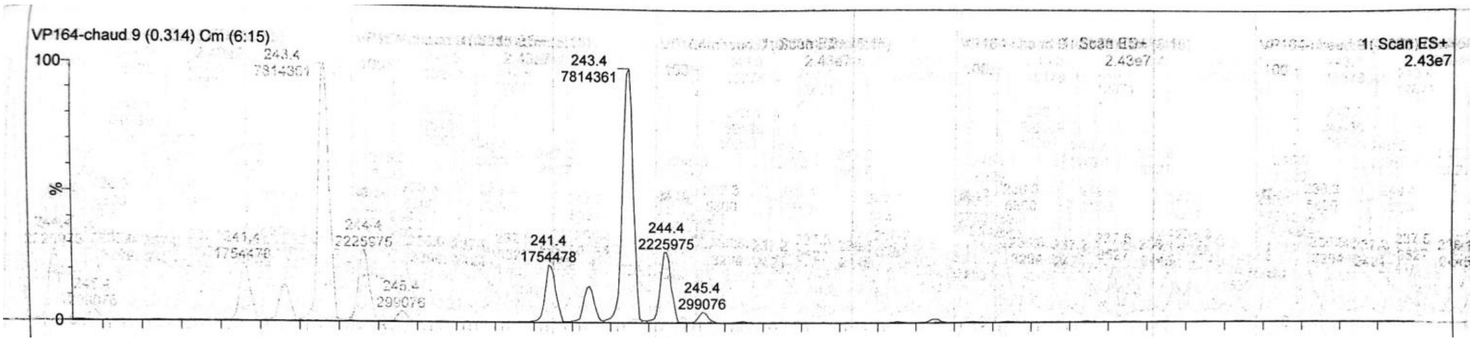

ESI-spectrum of **19**

Fluconazole **20**

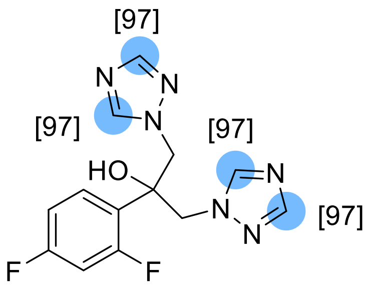

Chemical Formula: $C_{13}H_{12}F_2N_6O$

Substrate	Solvent (Volume)	RuNp@PVP cat.
61.2mg, 0.2mmol	THF (2mL)	28.9mg, 10mol%

Workup and purification:

After cooling down to room temperature, EtOAc/Cy (1:1, 3mL) was added to the reaction mixture and stirred for 10min to let precipitate RuNp@PVP. The suspension was passed through a Sep-Pak® C18 cartridge and then eluted with EtOAc (5mL). The solvent was removed under vacuum.

Yield: 41.0mg, 67%, white solid

^{1}H NMR (400 MHz, Acetone-*d*$_6$): δ 8.28 (s, 0.07H), 7.76 (s, 0.07H), 7.42 – 7.33 (m, 1H), 7.08 – 6.98 (m, 1H), 6.89 – 6.81 (m, 1H), 5.63 (s, 1H), 4.90 (d, *J* = 15.0 Hz, 2H), 4.67 (d, *J* = 14.5 Hz, 2H).

Deuterium incorporation was expected at δ 8.28 and at δ 7.76. Isotopic enrichment values were determined against the integral at δ 6.89 – 6.81.

^{2}H-{^{1}H}NMR (92 MHz, Acetone): δ 8.26 (s, 0.97D), 7.75 (s, 0.97D).

^{13}C-{^{1}H}NMR (100 MHz, Acetone-*d*$_6$): δ 165.2 – 161.2 (m), 162.7 – 158.7 (m), 152.2 (m), 146.0 (m), 131.6 – 130.4 (m), 124.6 – 123.8 (m), 112.6 – 111.4 (m), 105.4 – 104.0 (m), 75.9 – 75.1 (m), 56.2 – 55.1 (m).

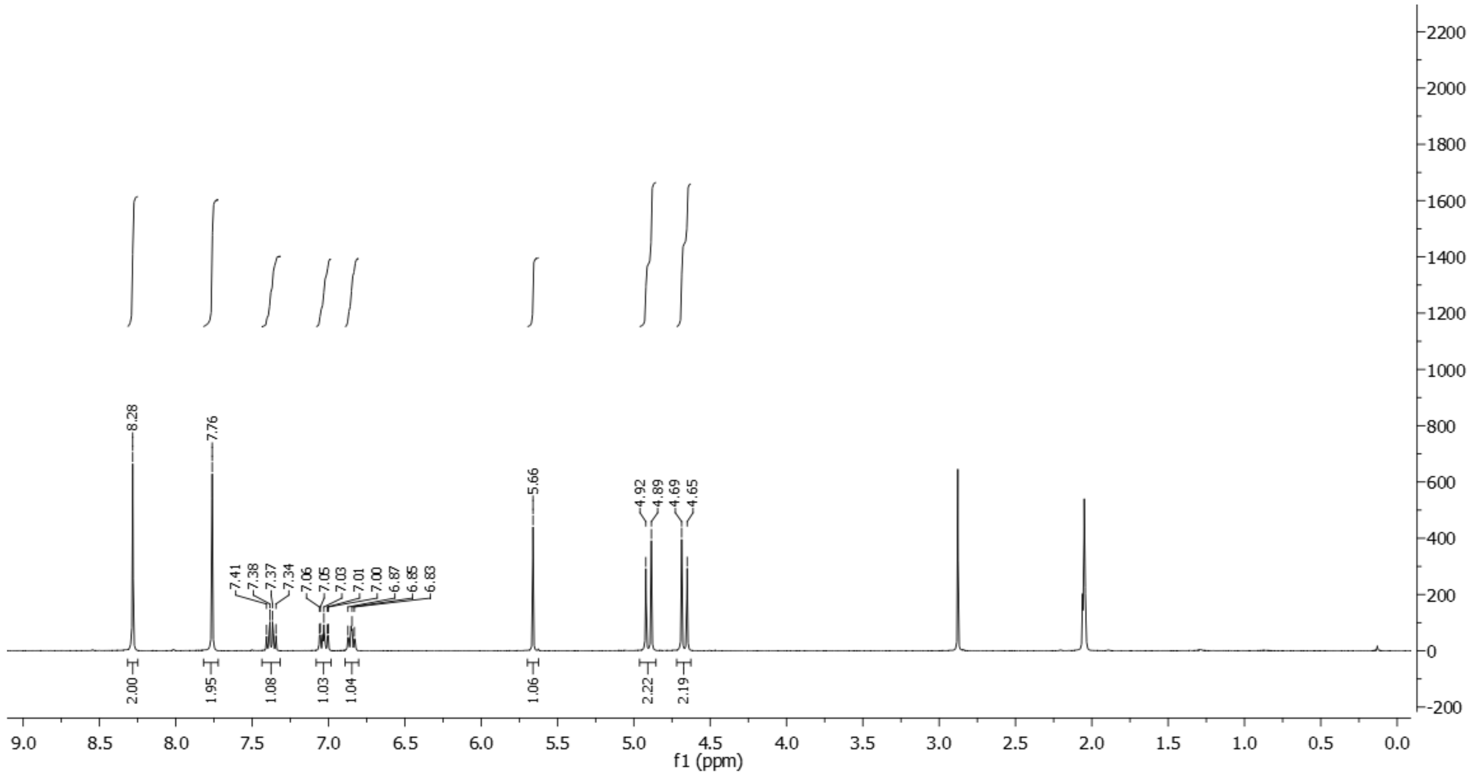

^{1}H-NMR spectrum of the non-deuterated starting material

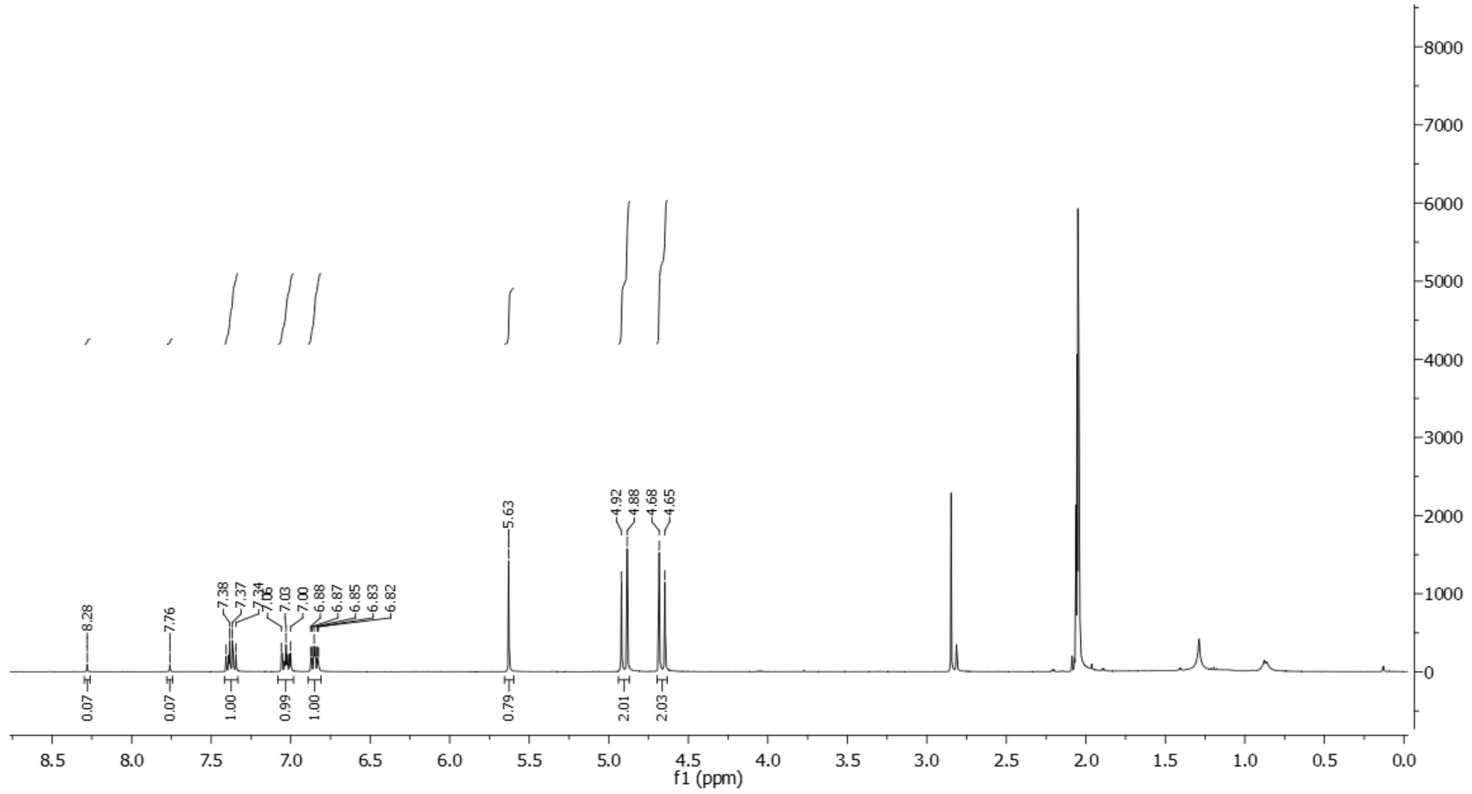

^{1}H-NMR spectrum of **20**

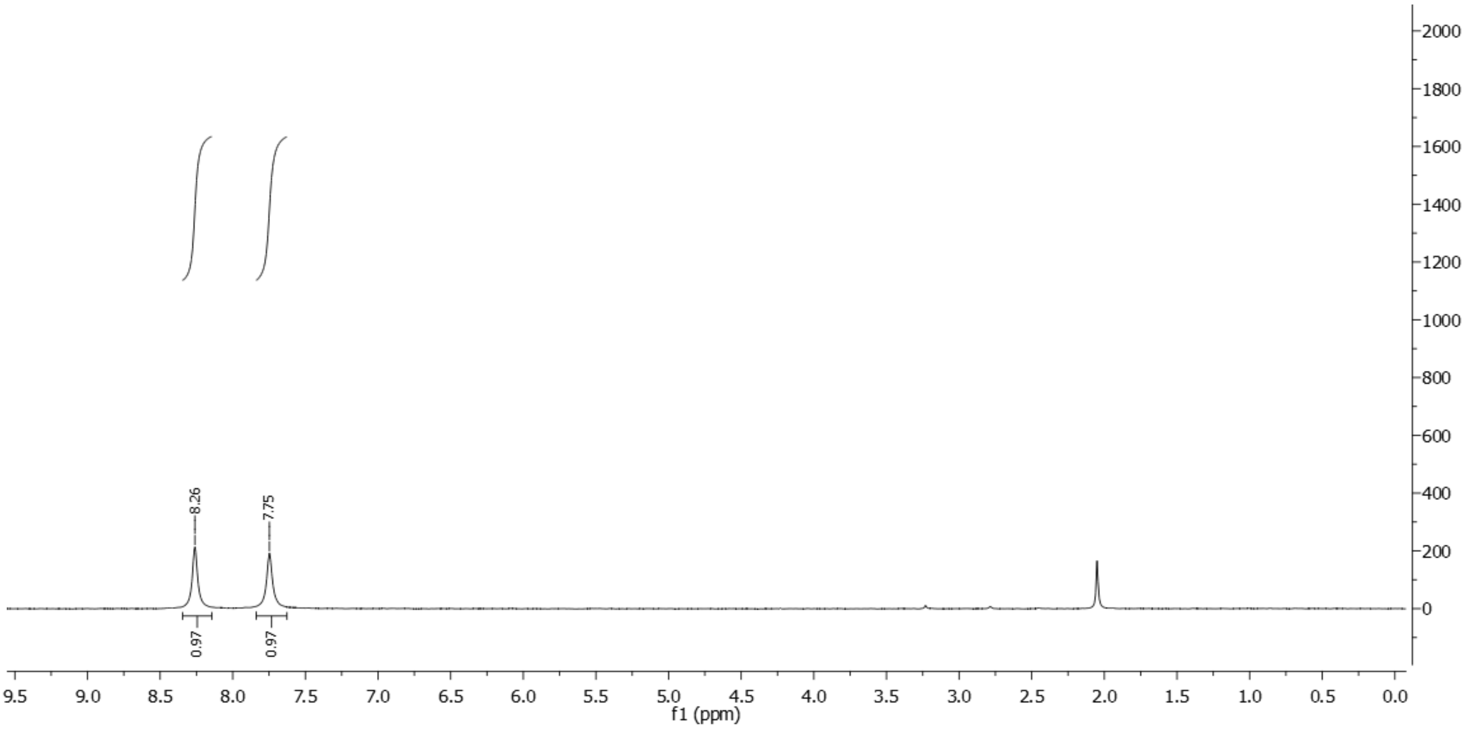

^{2}H-NMR spectrum of **20**

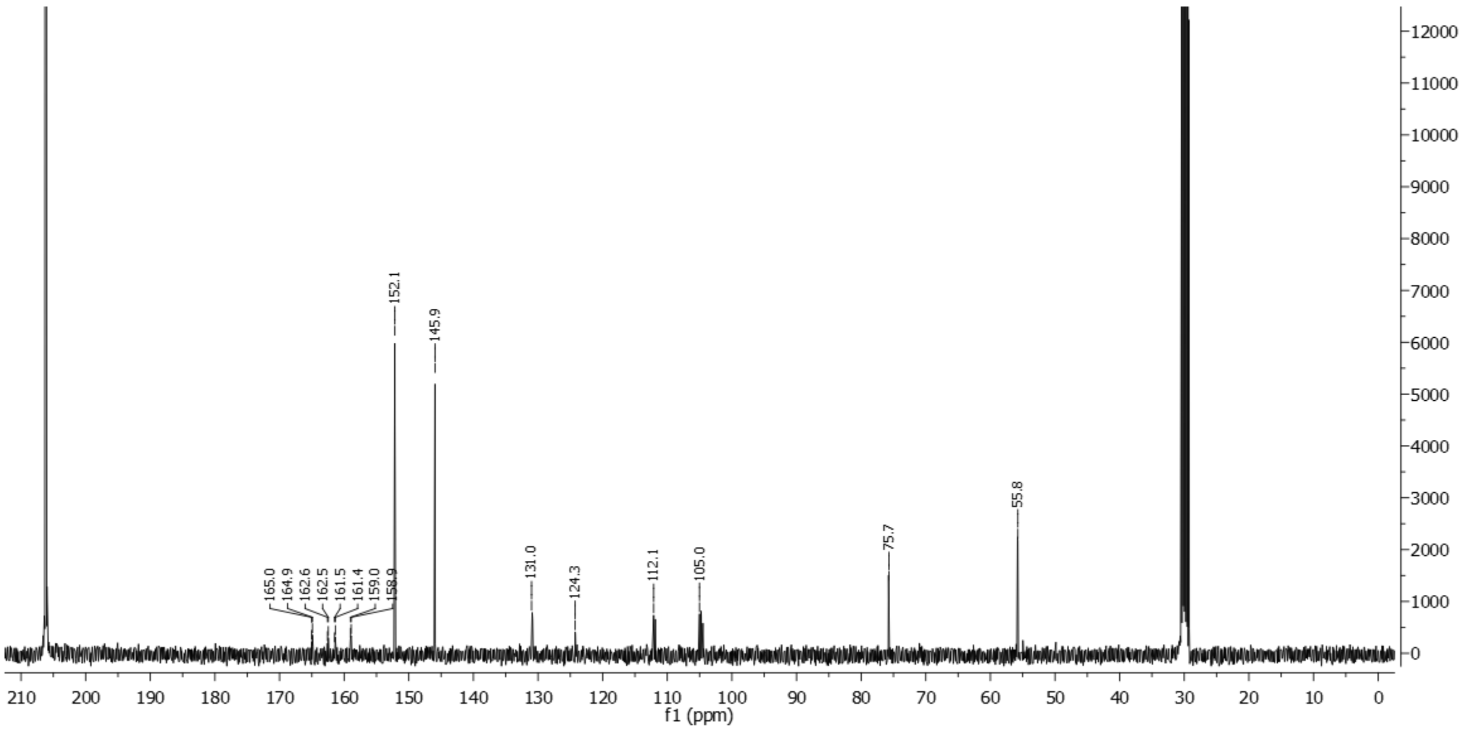

^{13}C-NMR spectrum of the non-deuterated starting material

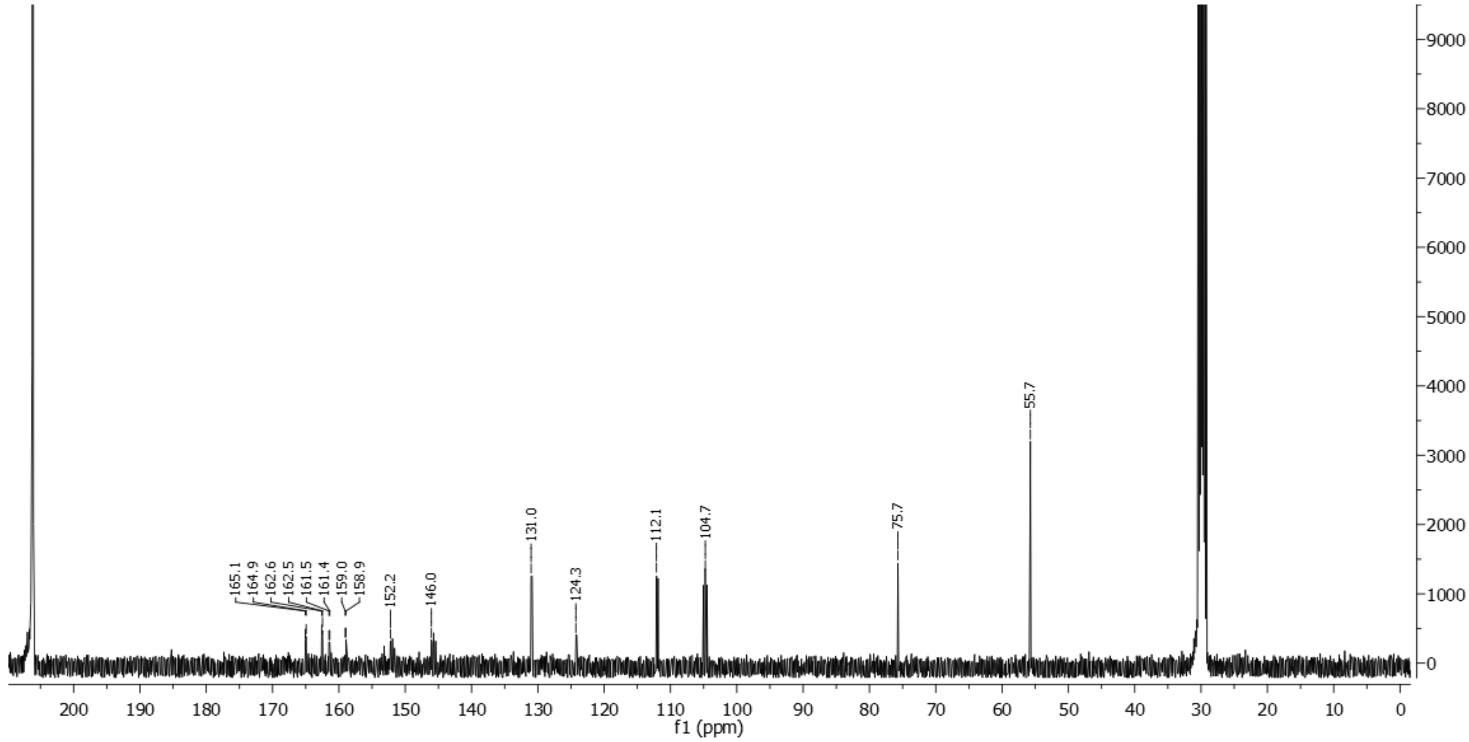

^{13}C-NMR spectrum of **20**

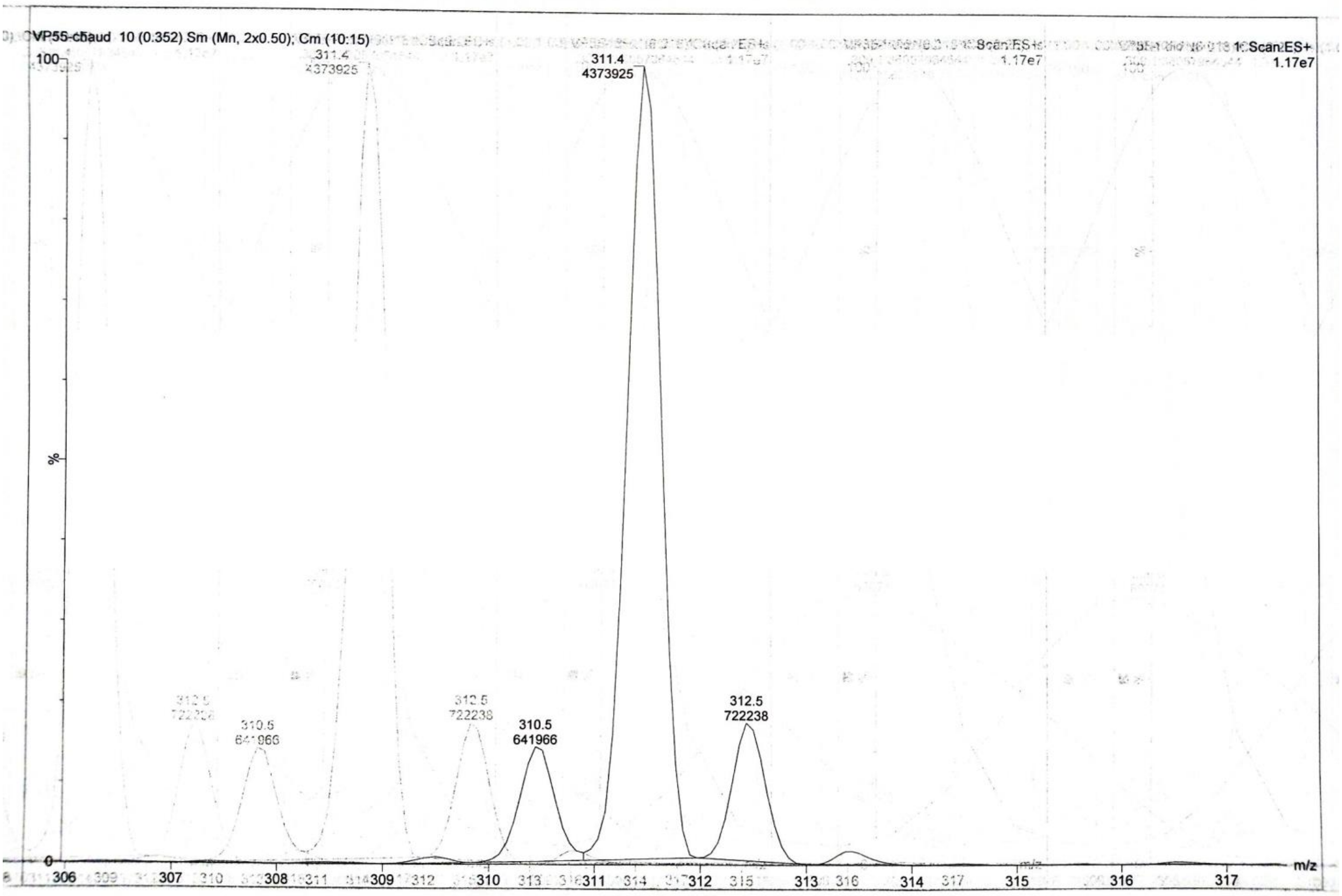

ESI-spectrum of **20**

Fluquinconazole **21**

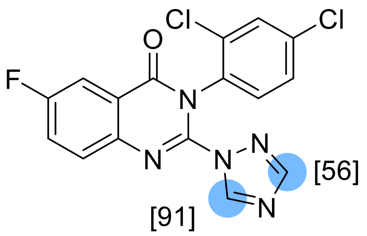

Chemical Formula: $C_{16}H_8Cl_2FN_5O$

Substrate	Solvent (Volume)	RuNp@PVP cat.
10.0mg, 27μmol	THF (2mL)	7.2mg, 19mol%

Workup and purification:

After cooling down to room temperature, EtOAc / Cy (1:1, 3mL) was added to the reaction mixture and stirred for 10min to let precipitate RuNp@PVP. The suspension was passed through a Sep-Pak® C18 cartridge and then eluted with EtOAc (5mL). The solvent was removed under vacuum.

Yield: 7.0mg, 70%, white solid

^{1}H NMR (400 MHz, Acetone-*d$_6$*): δ 9.05 (s, 1H), 7.97 – 7.88 (m, 2.30H), 7.85 – 7.76 (m, 1H), 7.70 – 7.66 (m, 1H), 7.65 – 7.59 (m, 1H), 7.51 – 7.43 (m, 1H).

Deuterium incorporation was expected at *δ* 9.05 and at *δ* 7.97 – 7.88. Isotopic enrichment values were determined against the integral at *δ* 7.51 – 7.43.

^{2}H-{^{1}H}NMR (92 MHz, Acetone): δ 9.03 (s, 0.91D), 7.88 (s, 0.56D).

^{13}C-{^{1}H}NMR (100 MHz, Acetone-*d$_6$*): δ 164.0 – 160.8 (m), 161.3, 153.5, 146.9 (m), 143.6, 142.1, 136.4, 134.9, 133.8, 132.7, 131.7 – 131.3 (m), 130.2, 128.9, 125.2 – 124.5 (m), 123.4 – 123.2 (m), 113.4 – 112.7 (m).

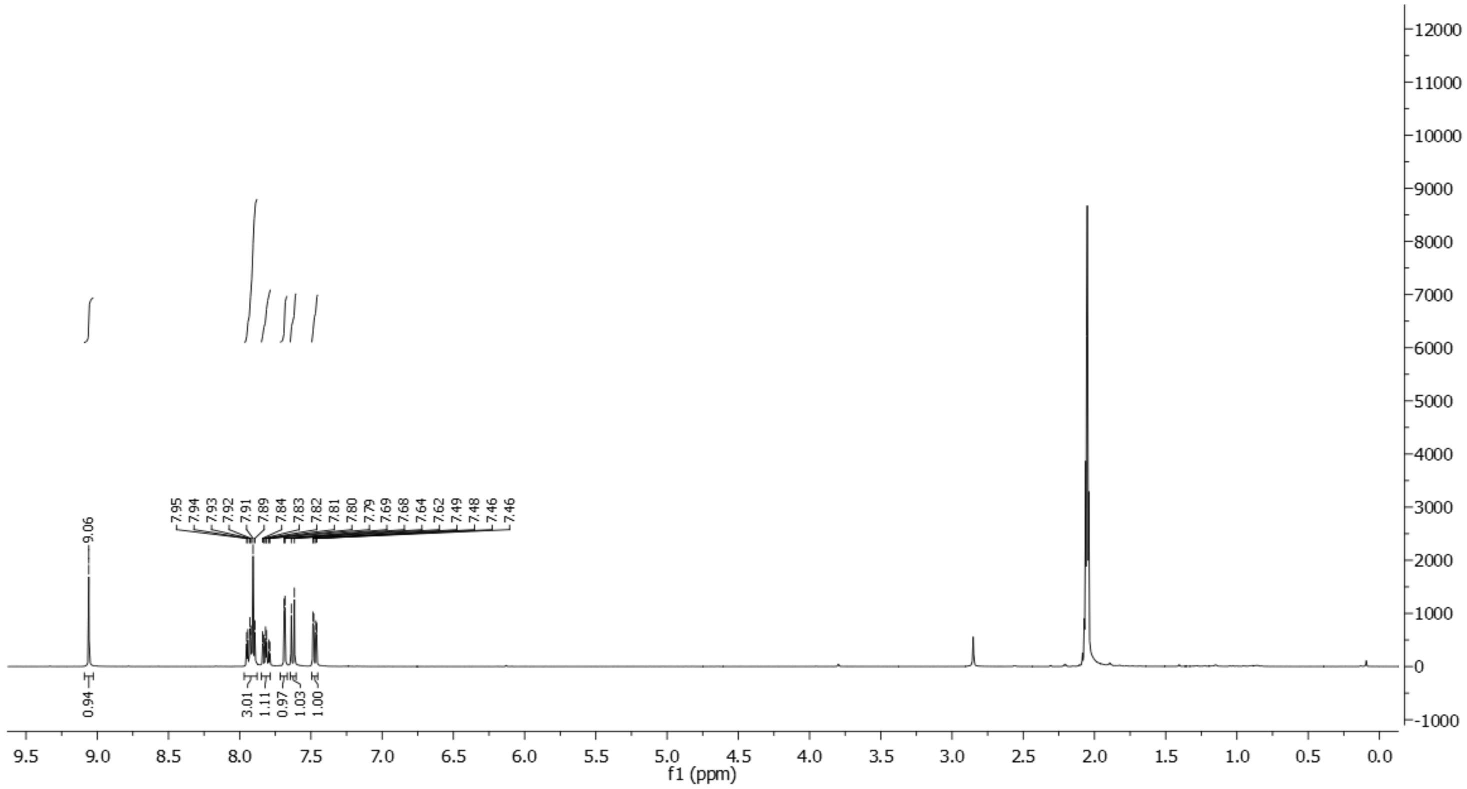

^{1}H-NMR spectrum of the non-deuterated starting material

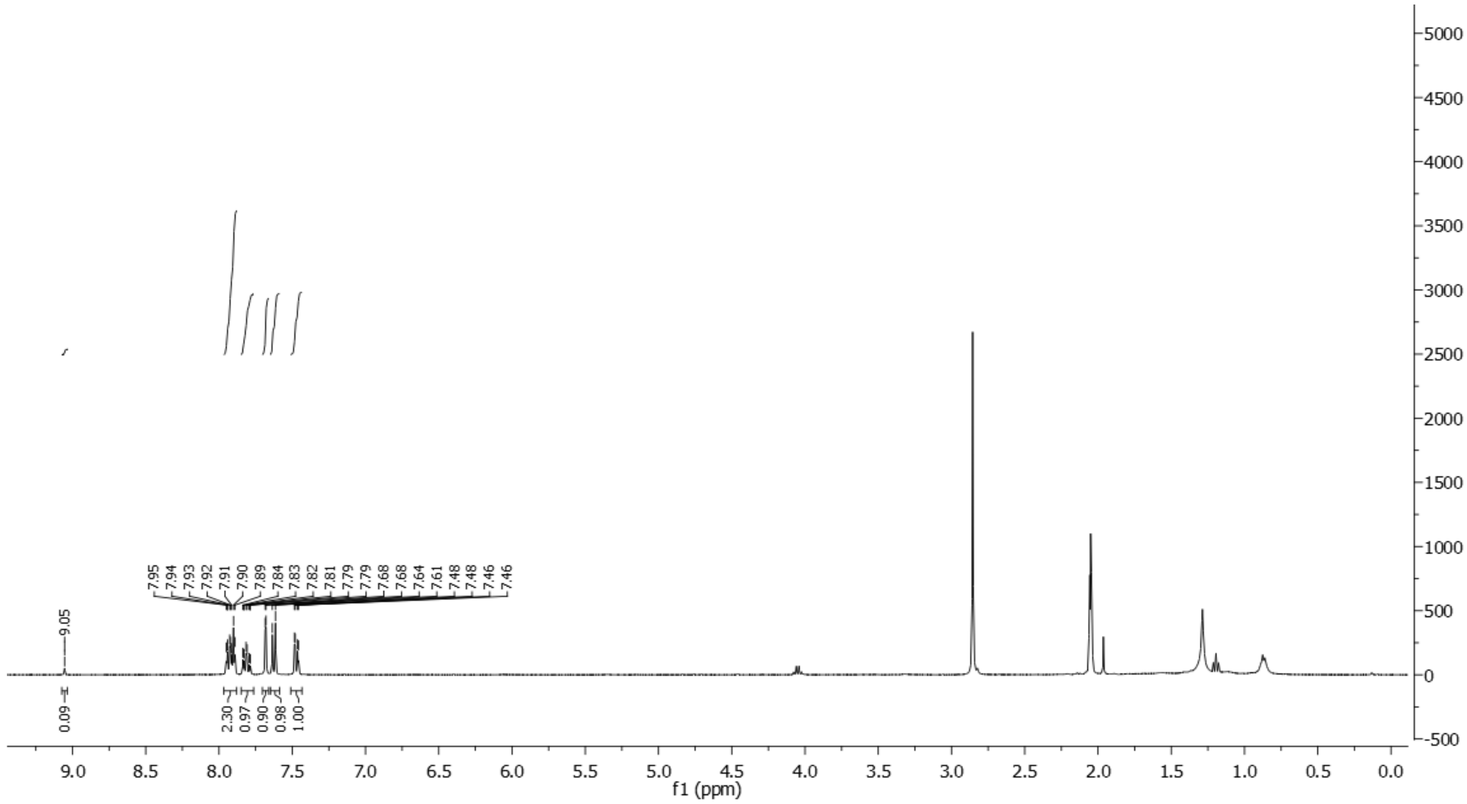

^{1}H-NMR spectrum of **21**

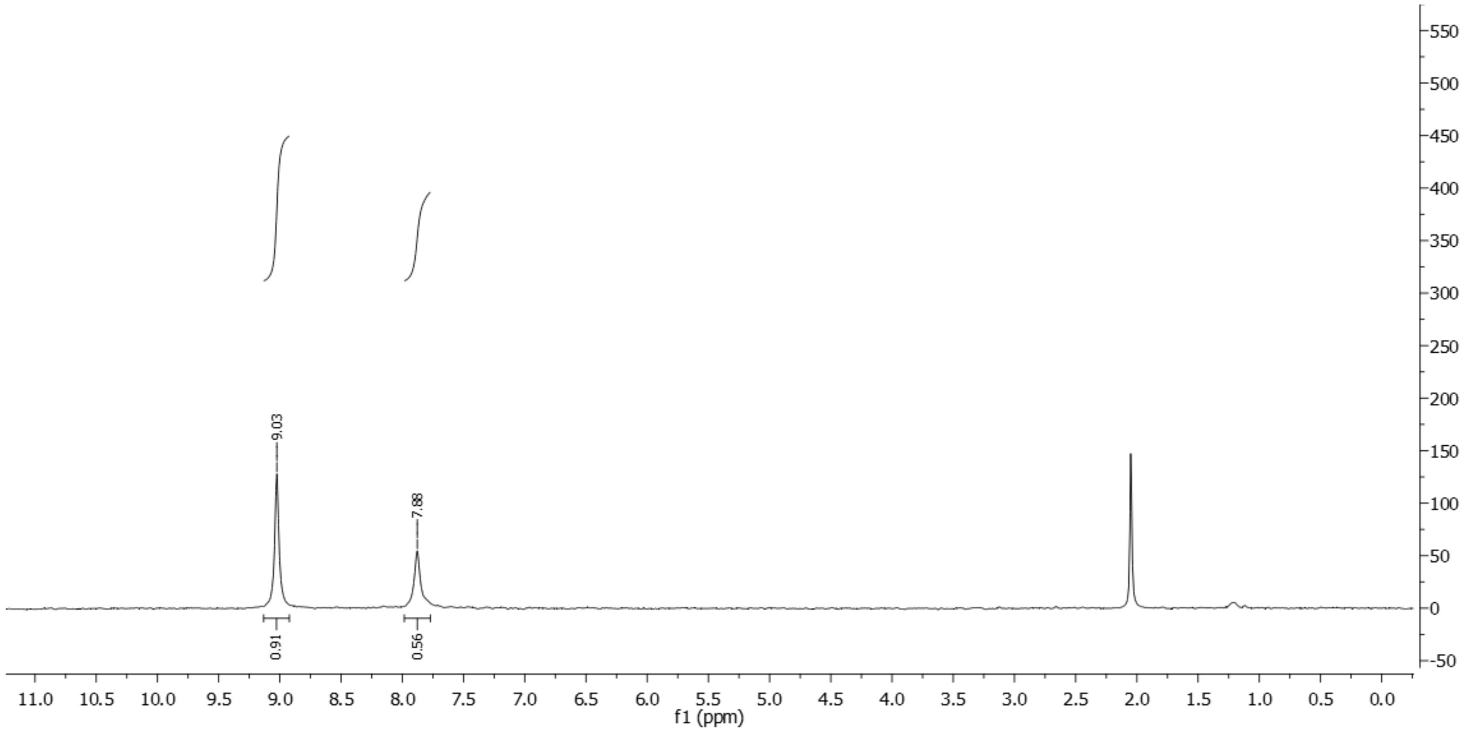

^{2}H-NMR spectrum of **21**

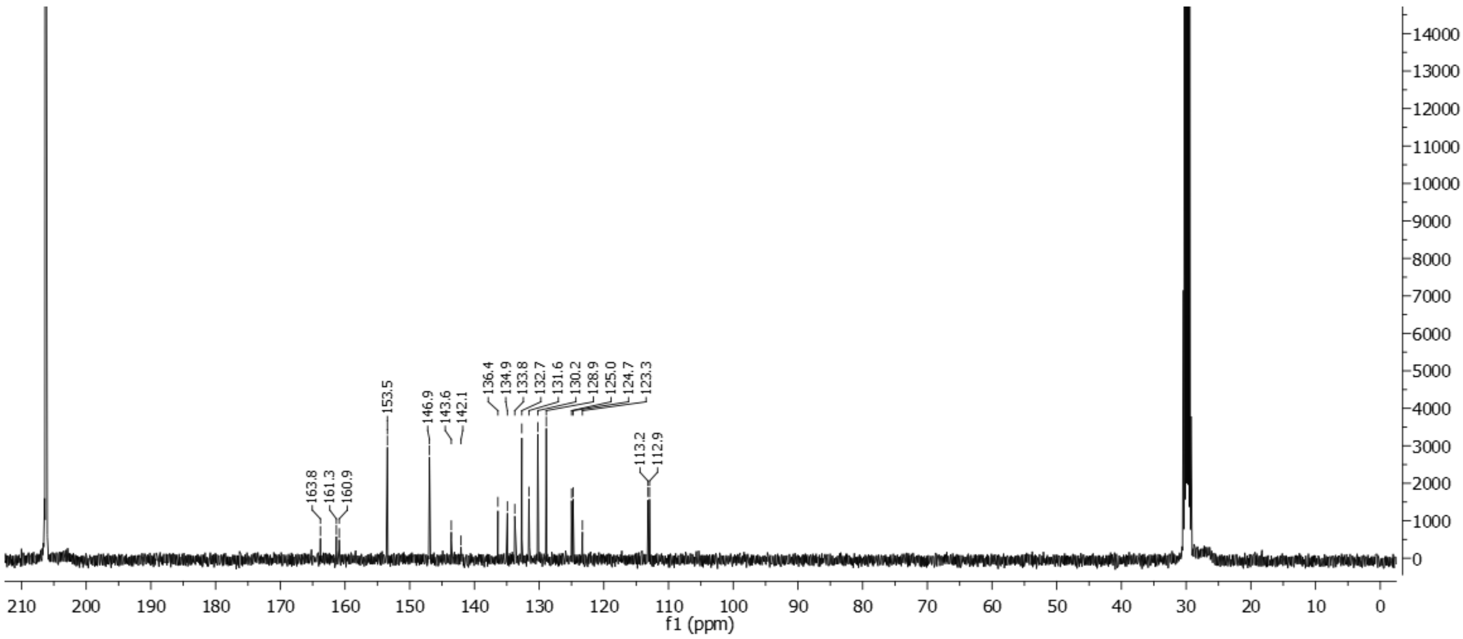

^{13}C-NMR spectrum of the non-deuterated starting material

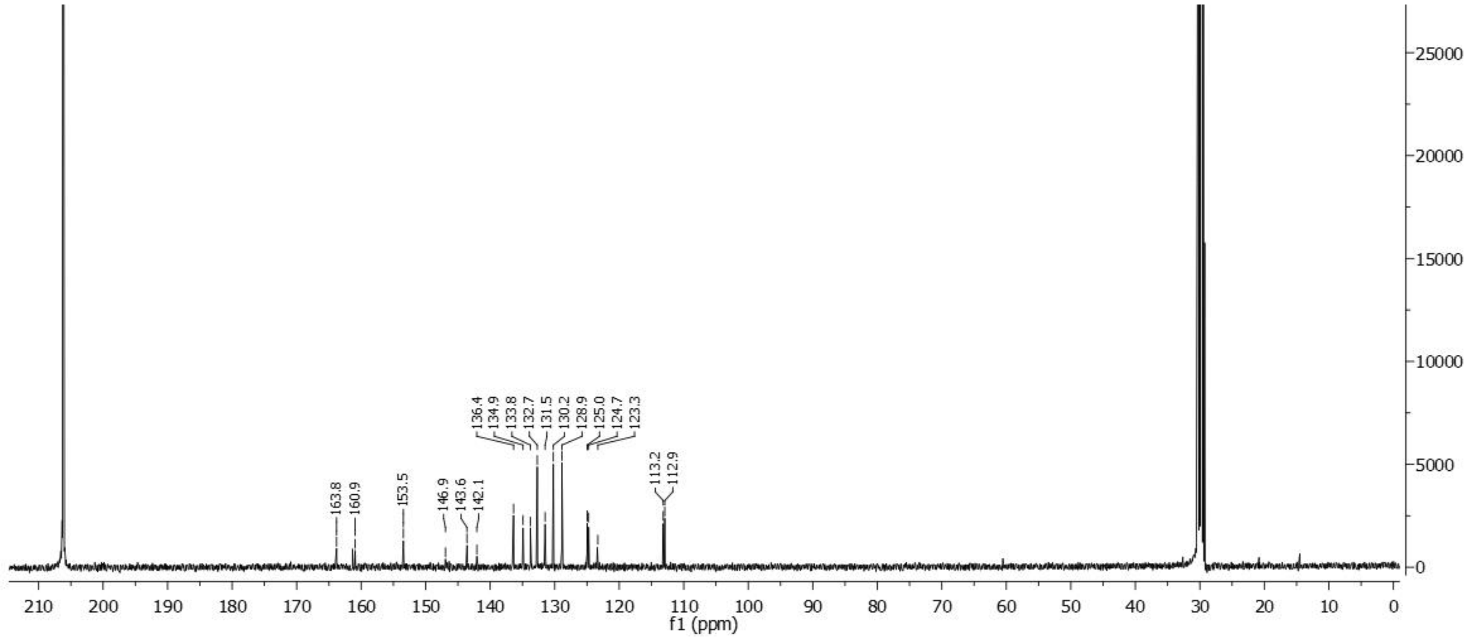

^{13}C-NMR spectrum of **21**

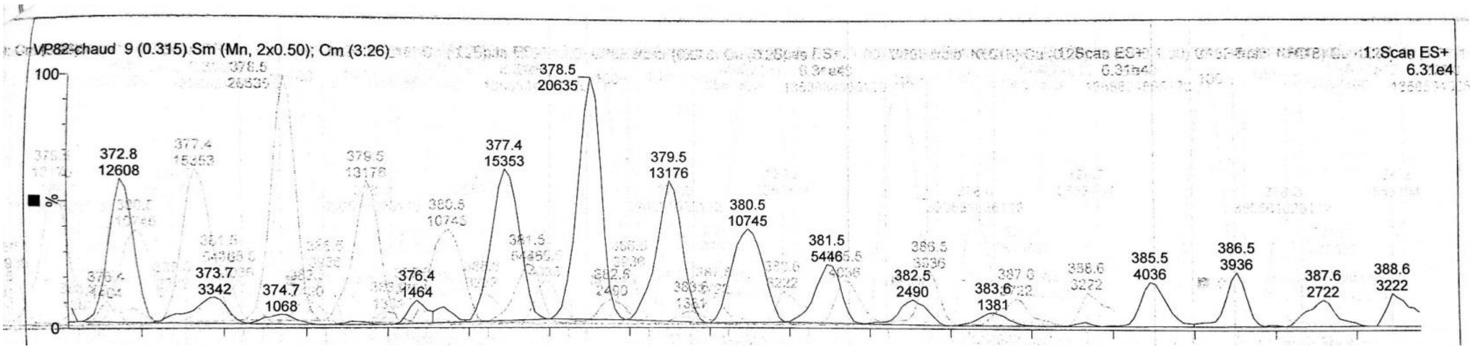

ESI spectrum of **21**

Suvorexant **22**

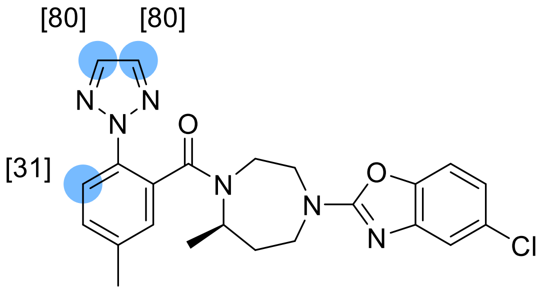

Chemical Formula: $C_{23}H_{23}ClN_6O_2$

Substrate	Solvent (Volume)	RuNp@PVP cat.	Reaction time
22.0mg, 50μmol	THF (0.5mL)	14.44mg, 20mol%	60h

Workup and purification:

After cooling down to room temperature, EtOAc (2mL) was added to the reaction mixture and stirred for 10min to let precipitate RuNp@PVP. The suspension was passed through a basic Al_2O_3 pad and then eluted with EtOAc (3mL). The solvent was removed under vacuum.

Yield: 23.0mg, 99%, white solid

The complexity of the recorded NMR spectra of suvorexant **22** can be explained by the presence of several rotamers in solution (acetone-*d*$_6$) at room temperature.

^{1}H NMR (400 MHz, Acetone-*d*$_6$): δ 7.97 – 7.77 (m, 0.90H), 7.46 – 7.09 (m, 3.66H), 7.07 – 6.90 (m, 1H), 4.94 – 3.18 (m, 8H), 2.83 – 2.37 (m, 3H), 2.26 – 2.12 (m, 1H), 1.26 – 0.87 (m, 3H).

Deuterium incorporation was expected at *δ* 7.97 – 7.77 and at *δ* 7.46 – 7.09. Isotopic enrichment values were determined against the integral at *δ* 7.07 – 6.90.

^{2}H-{^{1}H}NMR (92 MHz, Acetone): δ 7.86 (s, 1.60D), 7.31 (s, 0.23D).

^{13}C-spectra were compared with spectra in literature. Just signals of the major rotamer are given.

^{13}C-{^{1}H}NMR (100 MHz, Acetone-*d*$_6$): δ 169.3, 164.3, 148.7, 146.5, 139.00, 136.6, 135.3, 131.6, 131.0, 129.6, 129.0, 123.4 (m), 120.5, 116.4, 110.3, 53.0, 47.7, 44.5, 41.3, 36.8, 20.9, 19.9.

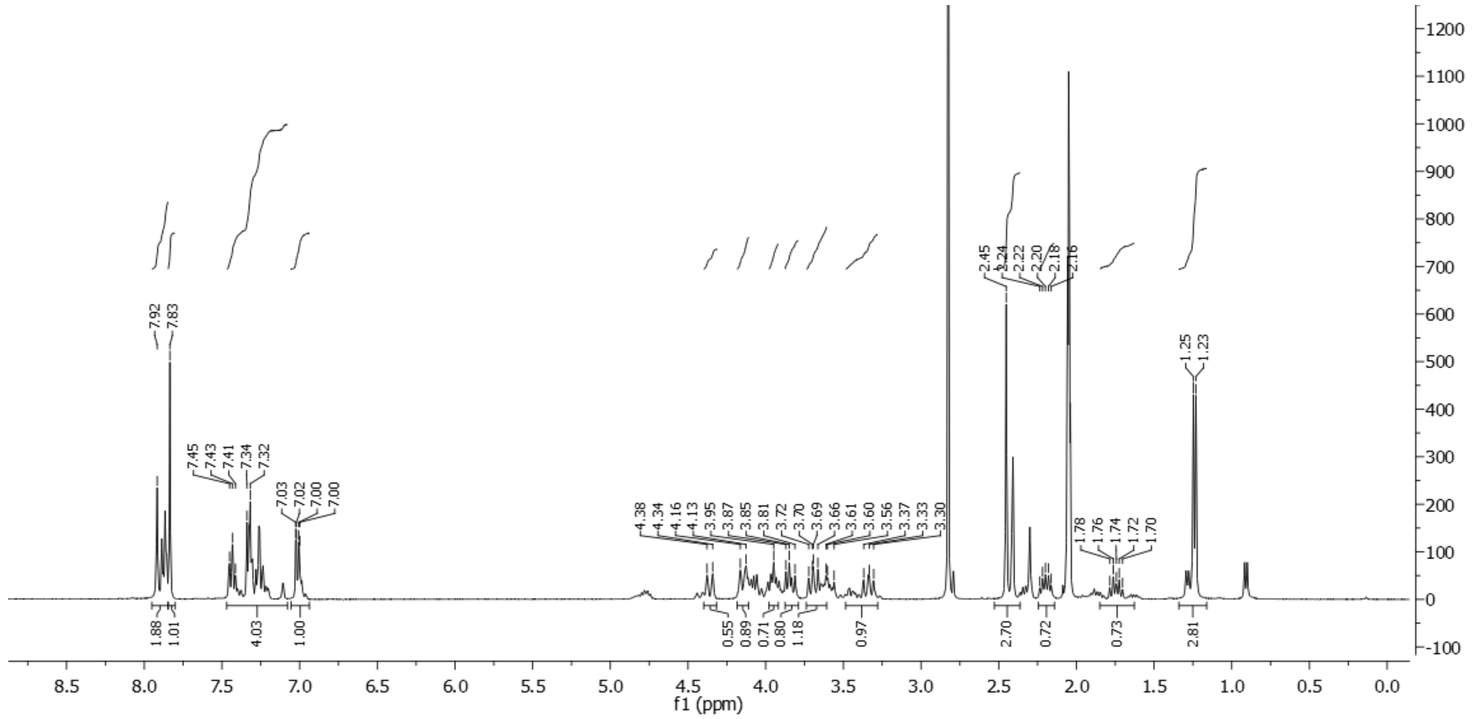

^{1}H-NMR spectrum of the non-deuterated starting material

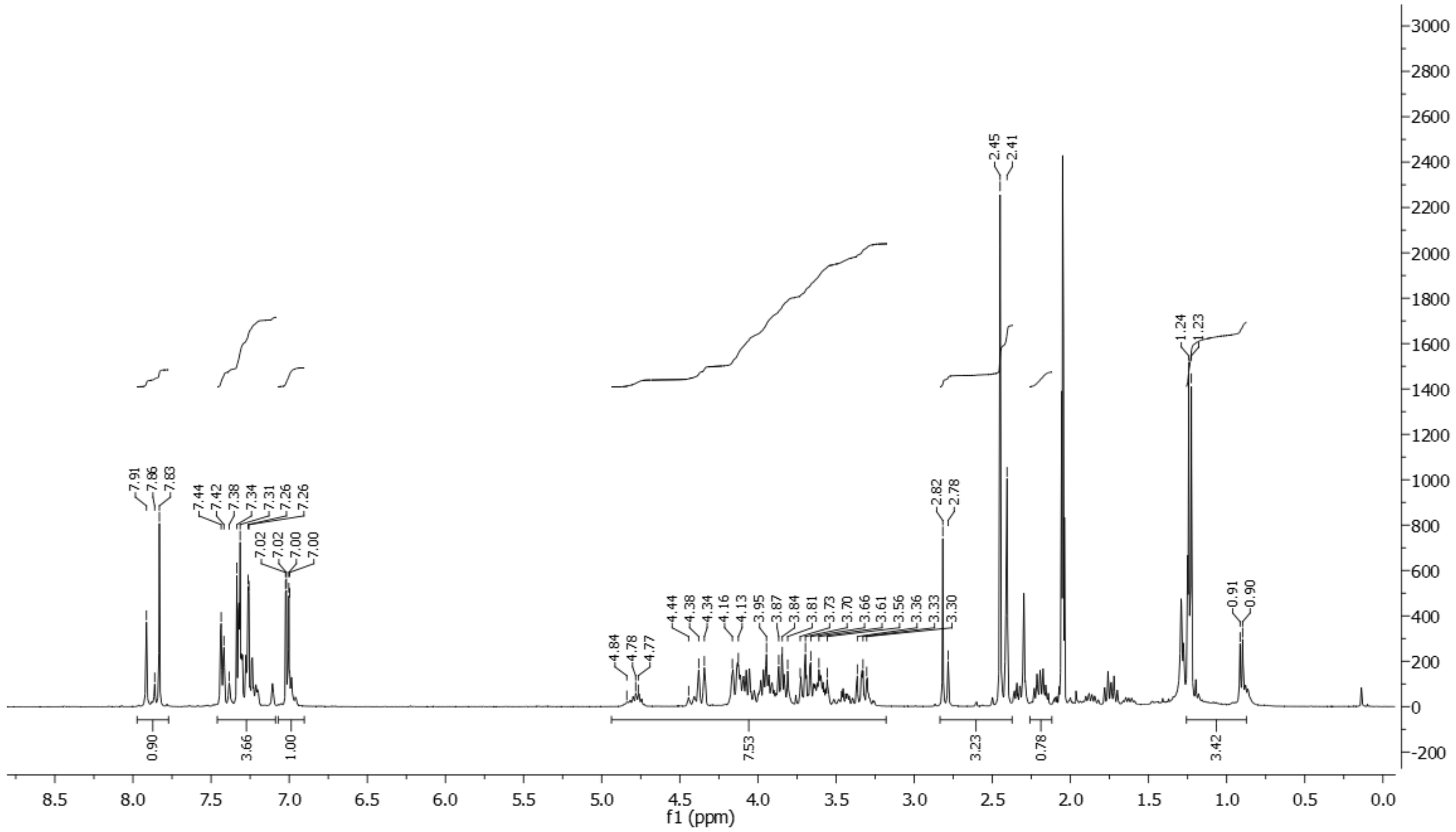

^{1}H-NMR spectrum of **22**

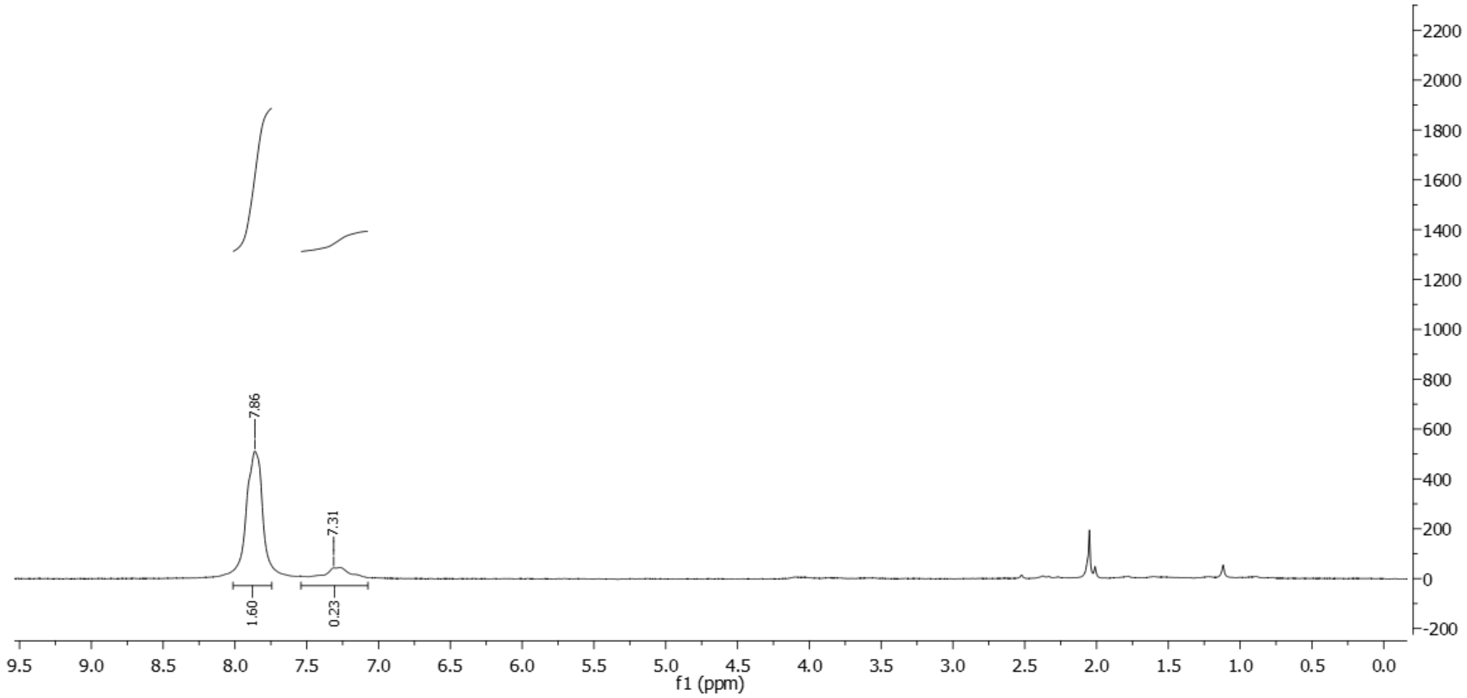

^{2}H-NMR spectrum of **22**

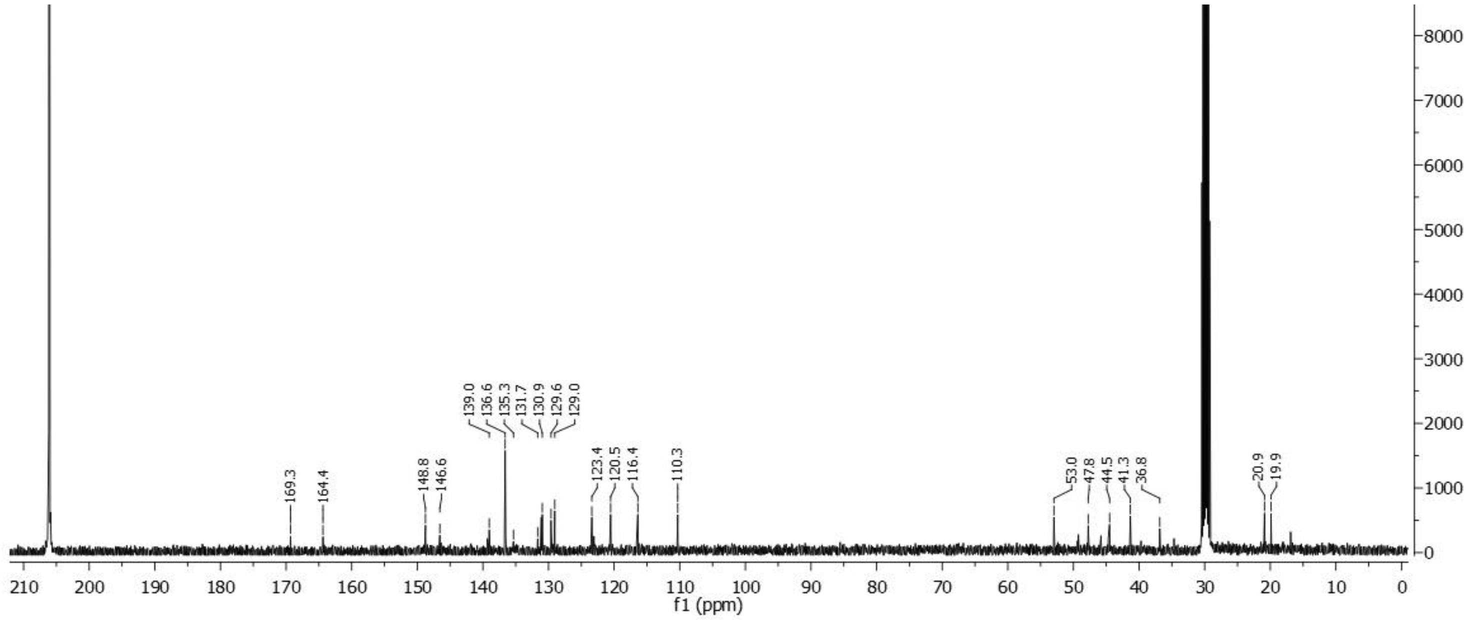

^{13}C-NMR spectrum of the non-deuterated starting material

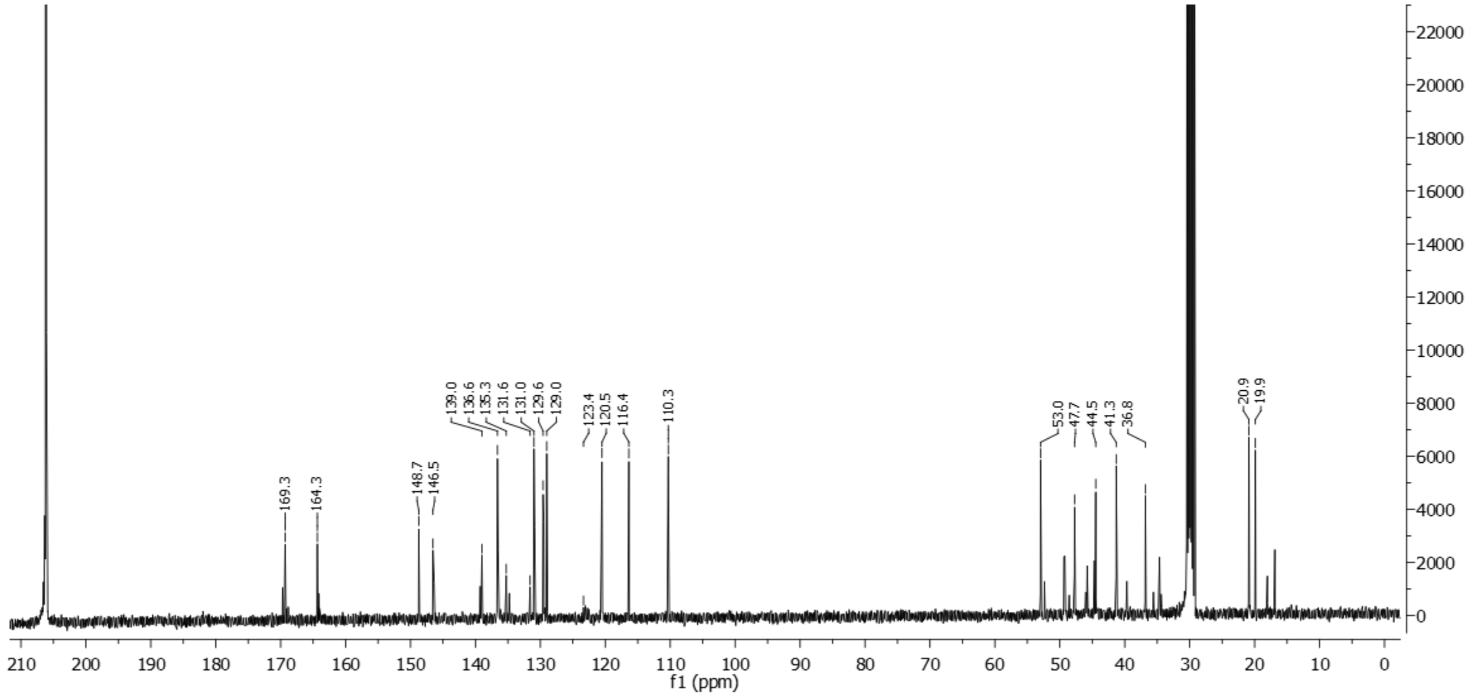

^{13}C-NMR spectrum of **22**

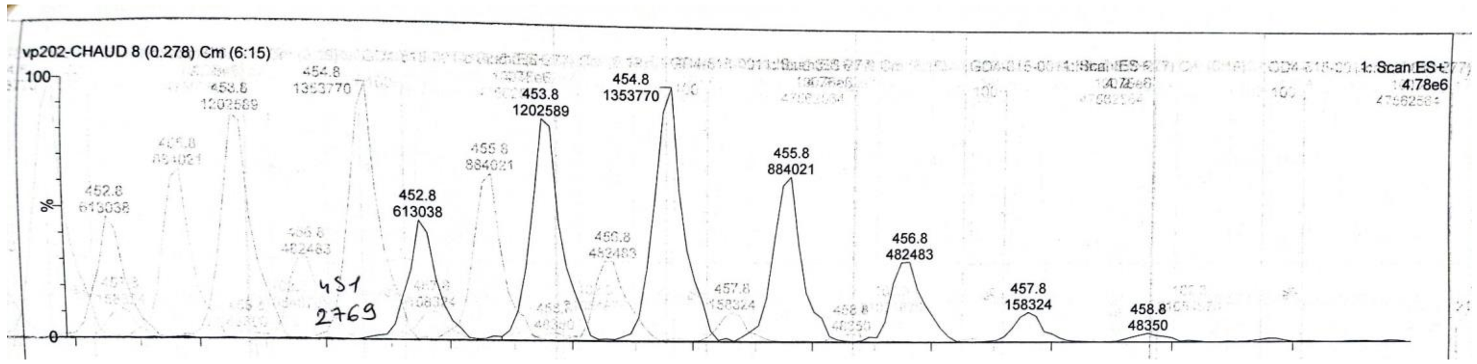

ESI spectrum of **22**

Carvedilol **23**

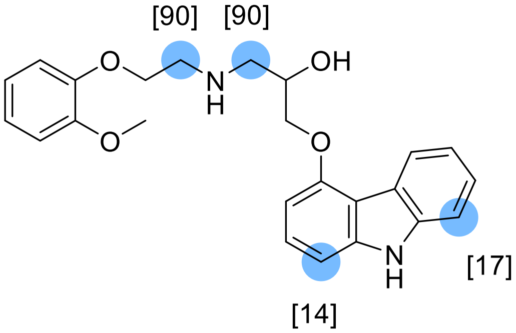

Chemical Formula: $C_{24}H_{26}N_2O_4$

Substrate	Solvent (Volume)	RuNp@PVP cat.
10.0mg, 25µmol	THF (1mL)	7.2mg, 20mol%

Workup and purification:

After cooling down to room temperature, EtOAc /Cy (1:1, 3mL) was added to the reaction mixture and stirred for 10min to let precipitate RuNp@PVP. The suspension was passed through a Sep-Pak® C18 cartridge and then eluted with EtOAc (5mL). The solvent was removed under vacuum.

Yield: 10.0mg, 99%, white solid

Carvedilol spectra were recorded in $CDCl_3$ because signal attribution in the same solvent succeeded already elsewhere.[lit.]

^{1}H NMR (400 MHz, $CDCl_3$): δ 8.29 – 8.23 (m, 1H), 8.18 (bs, NH), 7.44 – 7.28 (m, 3H), 7.24 – 7.16 (m, 1H), 7.09 – 7.01 (m, 0.88H), 6.99 – 6.81 (m, 4H), 6.72 – 6.62 (m, 1H), 4.34 – 4.09 (m, 5H), 3.83 (s, 3H), 3.41 – 3.31 (m, 0.43H).

Deuterium incorporation was expected at δ 9.05 and at δ 7.97 – 7.88. Isotopic enrichment values were determined against the integral at δ 8.29 – 8.23.

^{2}H-{^{1}H}NMR (92 MHz, $CHCl_3$): δ 7.45 (s, 0.17D), 7.09 (s, 0.14D), 3.25 – 2.75 (m, 3.6D).

^{13}C-{^{1}H}NMR (100 MHz, $CDCl_3$): δ 155.3, 149.8, 148.4, 141.1, 138.9, 126.8, 125.1, 123.1, 122.7, 121.8, 121.1, 119.8, 114.2, 112.9, 112.0, 110.1, 104.0, 101.4, 70.4, 68.8, 68.5, 55.9, 52.1 (m), 48.8 (m).

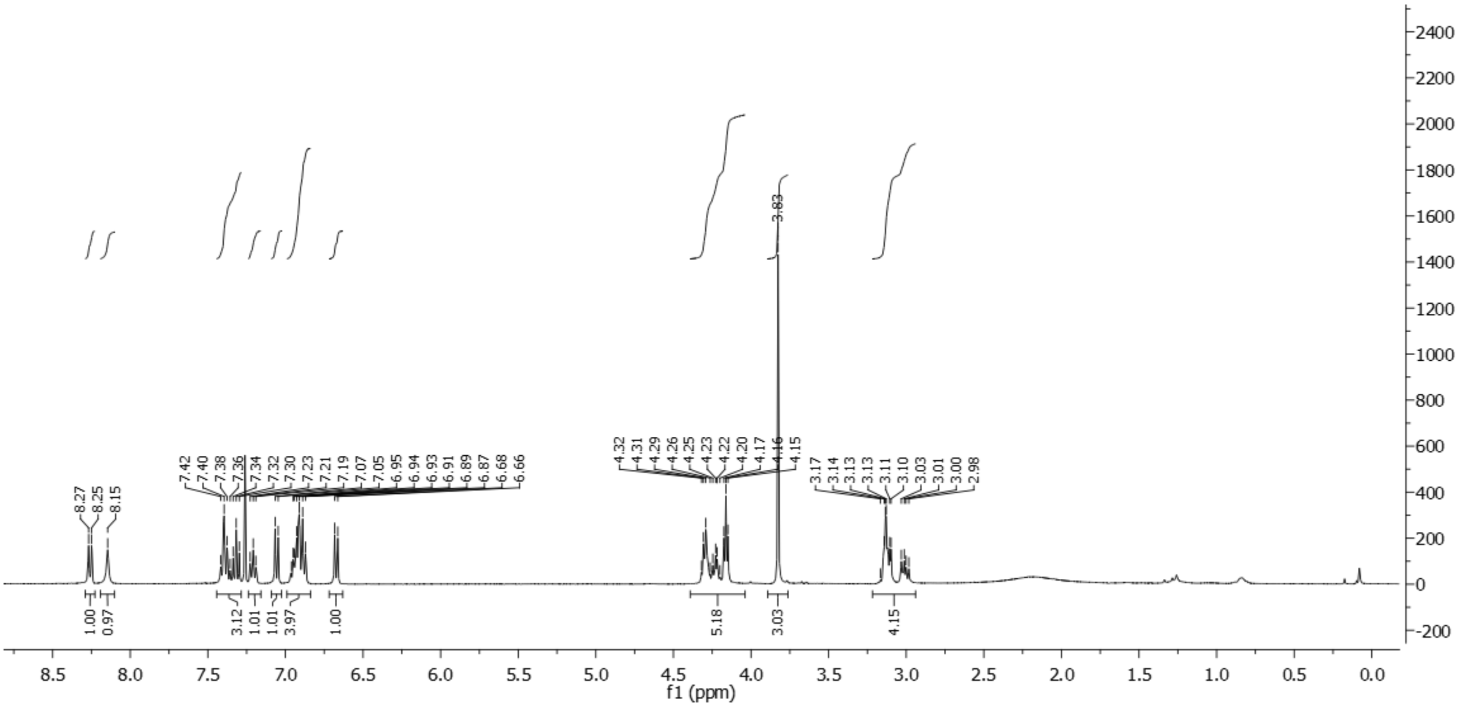

^{1}H-NMR spectrum of the non-deuterated starting material

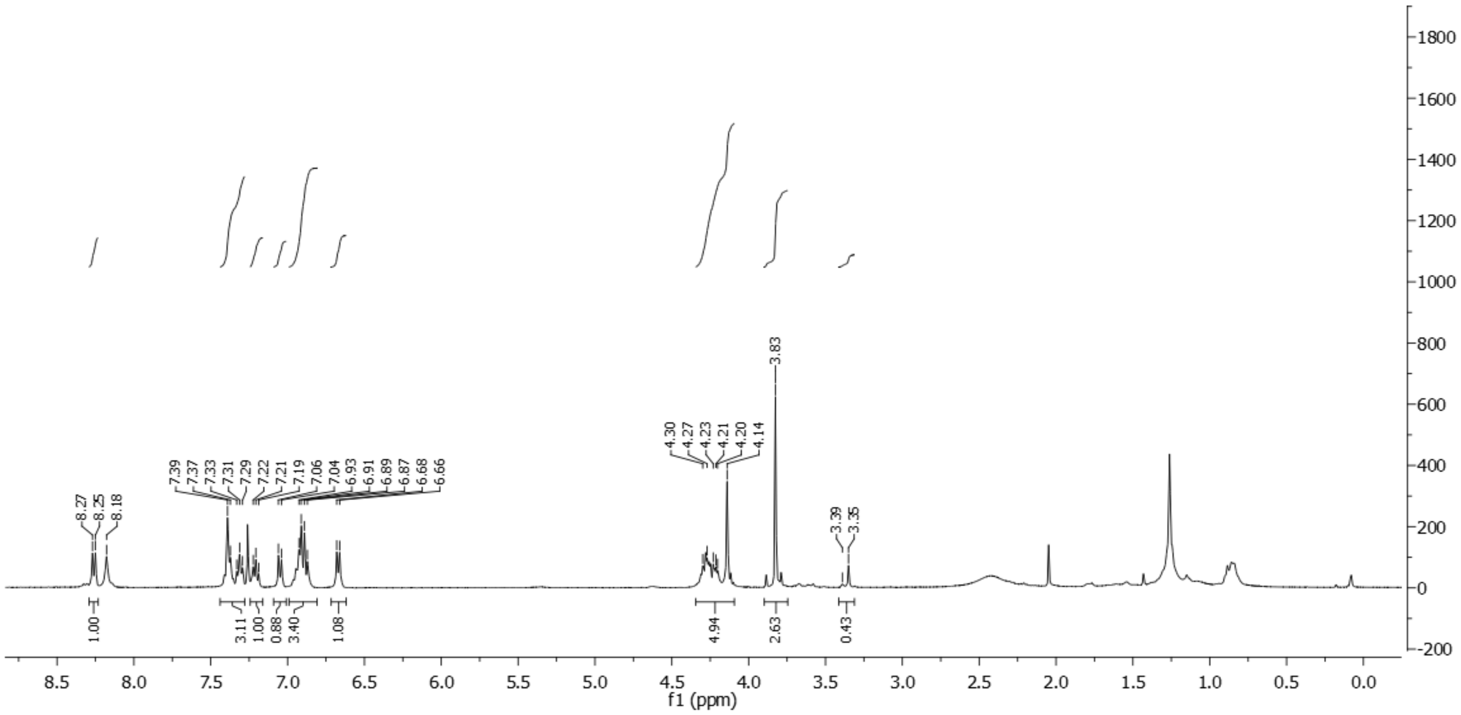

^{1}H-NMR spectrum of **23**

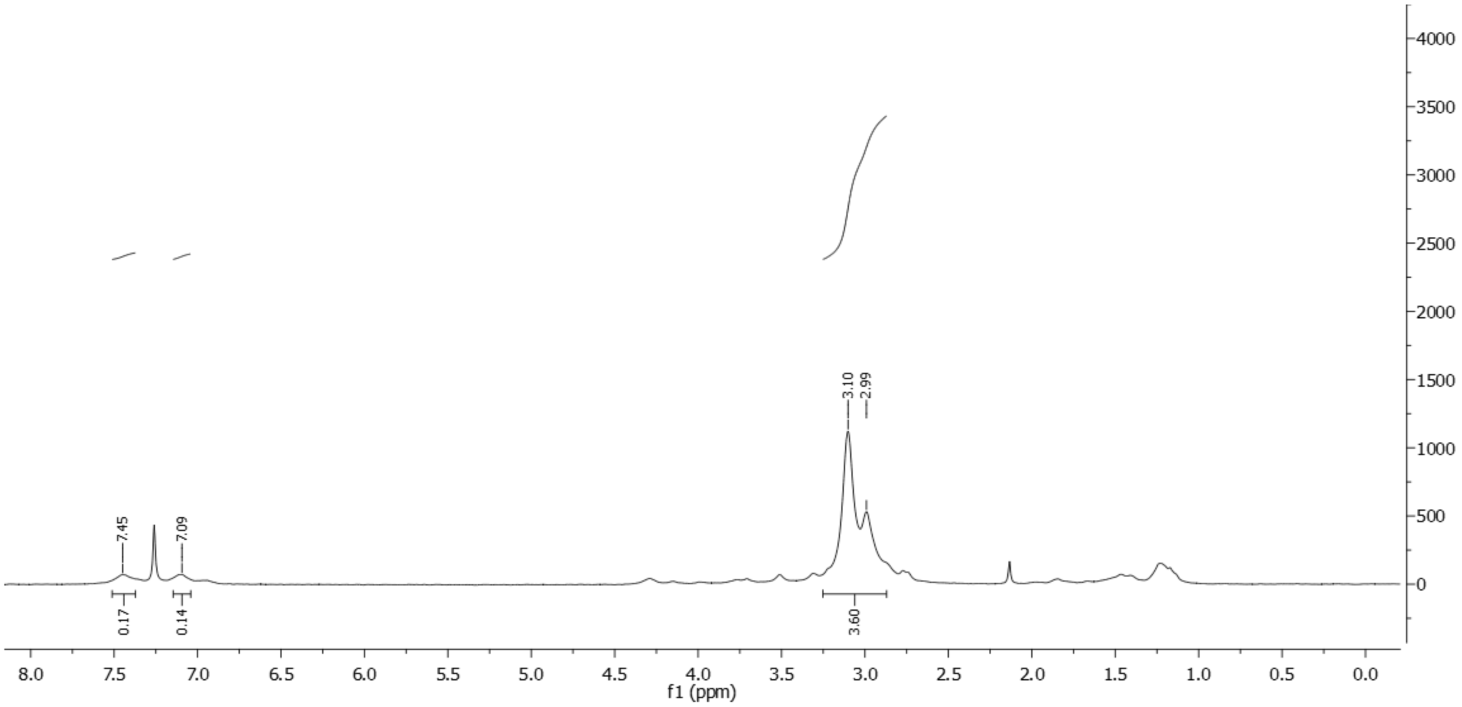

^{2}H-NMR spectrum of **23**

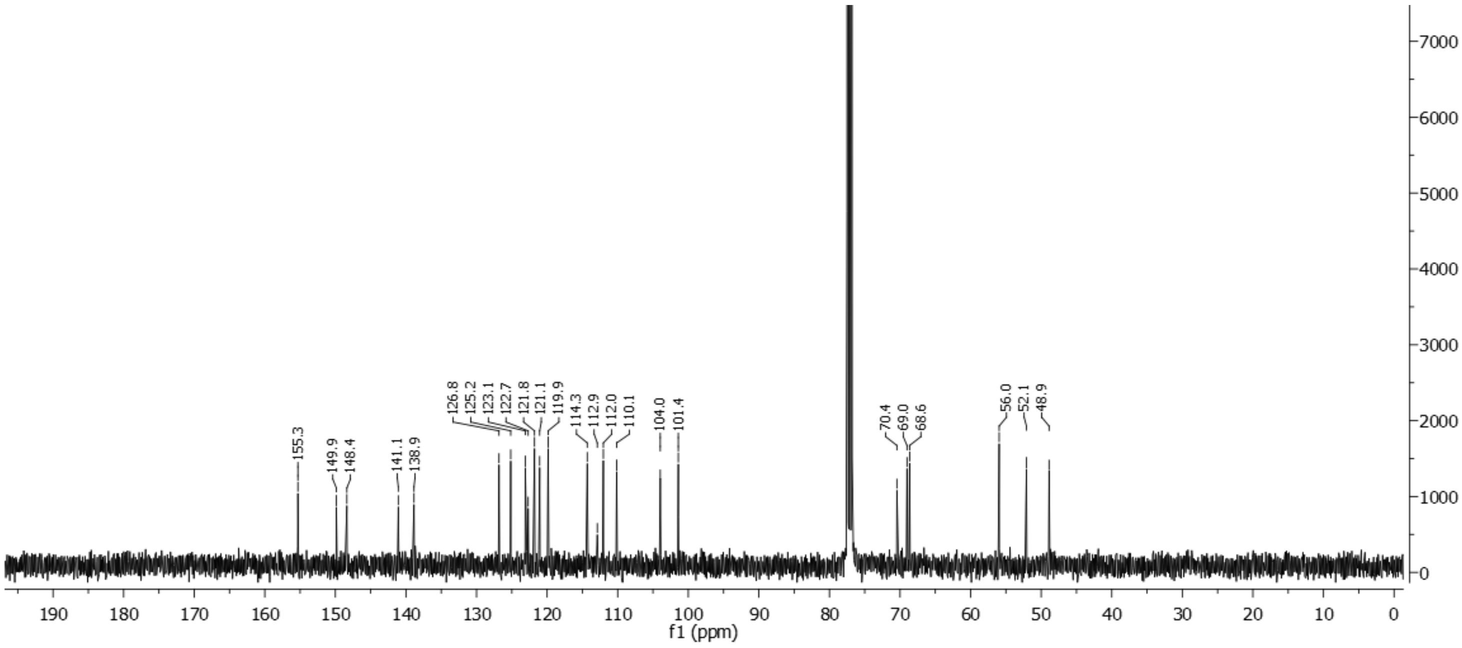

^{13}C-NMR spectrum of the non-deuterated starting material

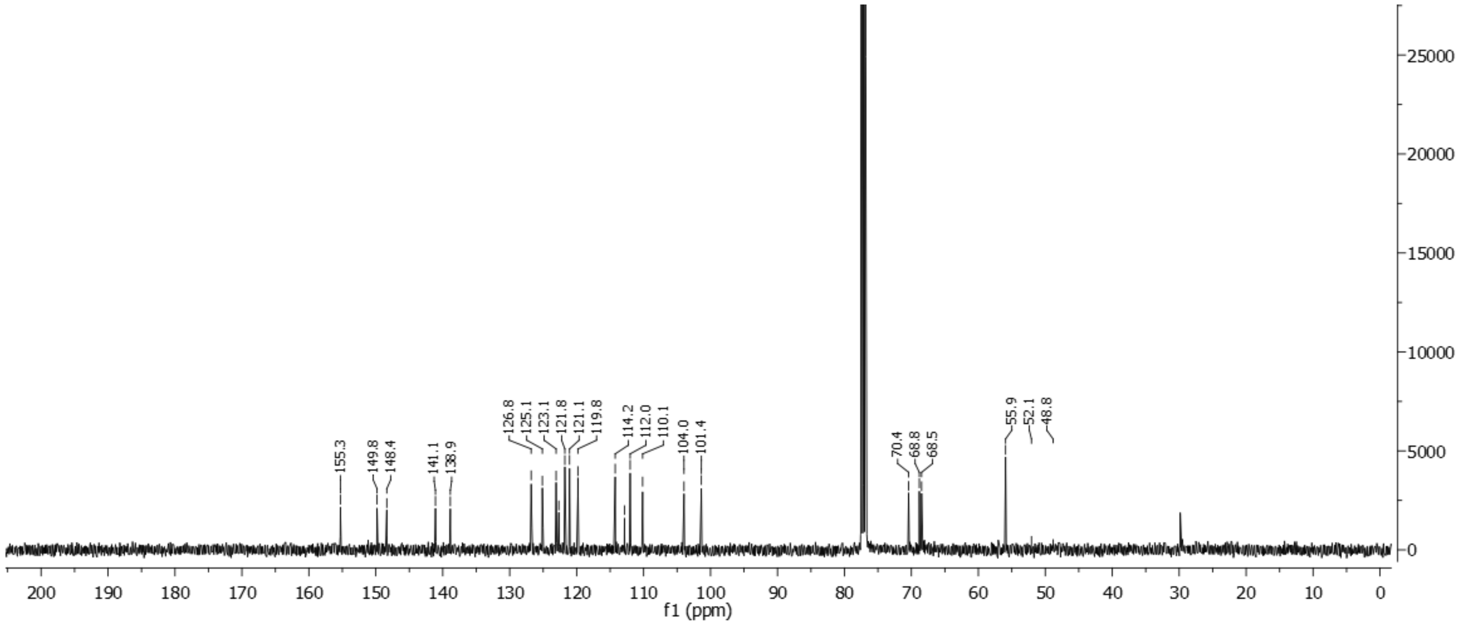

^{13}C-NMR spectrum of **23**

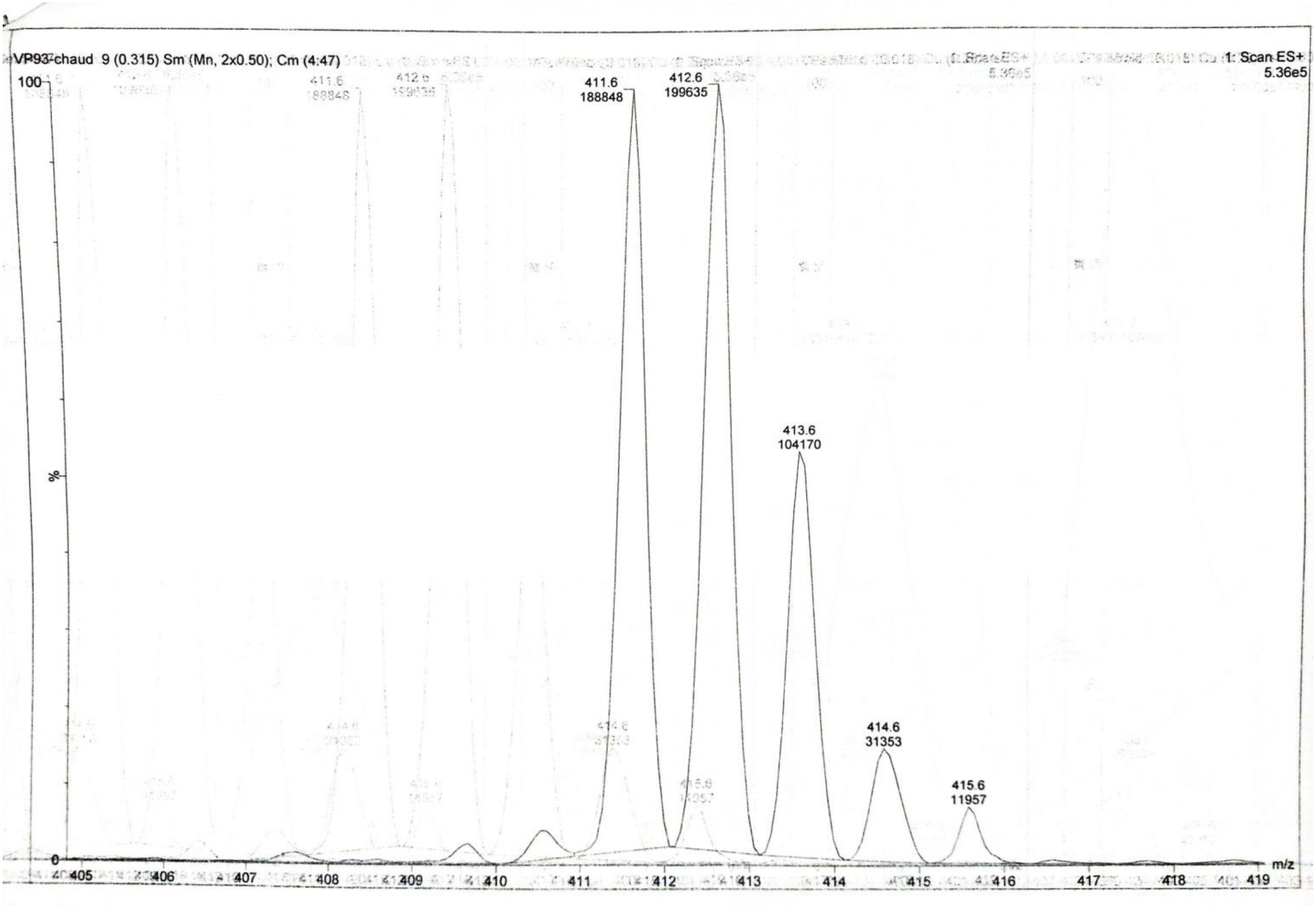

ESI spectrum of **23**

N-Boc-carvedilol **24**

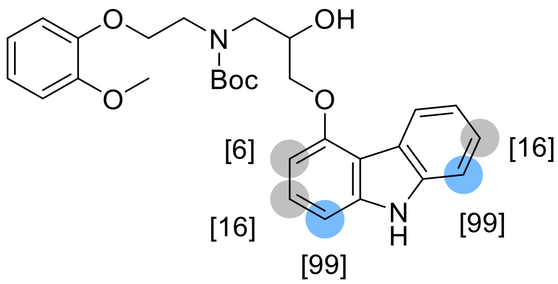

Chemical Formula: $C_{29}H_{34}N_2O_6$

Substrate	Cs_2CO_3	Solvent (Volume)	RuNp@PVP cat.
50.0mg, 0.1mmol	32.5mg, 0.1mmol	THF (1mL)	14.44mg, 10mol%

Workup and purification:

After cooling down to room temperature the reaction mixture was poured on a 5mM solution of acetic acid in H_2O dist. (100mL). The aqueous phase was extracted three times with EtOAc (3 x 50mL) in a separation funnel. The solvent was removed under vacuum and the crude product was purified over SiO_2. Deuterium labelled *N*-Boc-carvedilol could be eluted at Cy/EtOAc (3:1).

Yield: 43.0mg, 86%, white solid

The complexity of the ^{1}H- and ^{13}C-spectrum is increased by the appearance of different rotamers. Signals are designated as multiplets (m) when they could not be nearer specified in the ^{1}H-spectrum. Just signals of the major rotamer are given in the ^{13}C-spectrum.

^{1}H NMR (400 MHz, Acetone-*d*$_6$): δ 10.31 (bs, NH), 8.54 – 8.30 (m, 1H), 7.50 – 7.44 (m, 0.01H), 7.41 – 7.23 (m, 2H), 7.18 – 7.07 (m, 1.01H), 7.02 – 6.80 (m, 4H), 6.76 – 6.68 (m, 1H), 4.72 – 4.43 (m, 2H), 4.32 – 4.15 (m, 4H), 3.98 – 3.88 (m, 1H), 3.83 – 3.78 (m, 2H), 3.75 (s, 3H), 3.74 – 3.64 (m, 1H), 1.52 – 1.41 (m, 9H).

Deuterium incorporation was expected at *δ* 7.50 – 7.44 and at *δ* 7.18 – 7.07. Isotopic enrichment values were determined against the integral at *δ* 8.54 – 8.30.

^{2}H-{^{1}H}NMR (92 MHz, Acetone): δ 7.45 (s), 7.29 (s), 7.10 (s), 6.87 (s).

^{13}C-{^{1}H}NMR (100 MHz, Acetone-*d*$_6$): δ 157.1, 156.3, 150.8, 149.5, 142.4, 140.1, 127.2, 125.3, 124.2, 123.4, 122.2, 121.6, 119.7, 114.7, 113.3, 110.9 (m), 104.8 (m), 101.4, 80.2, 71.3, 70.5, 68.4, 56.1, 53.2, 49.3, 28.6.

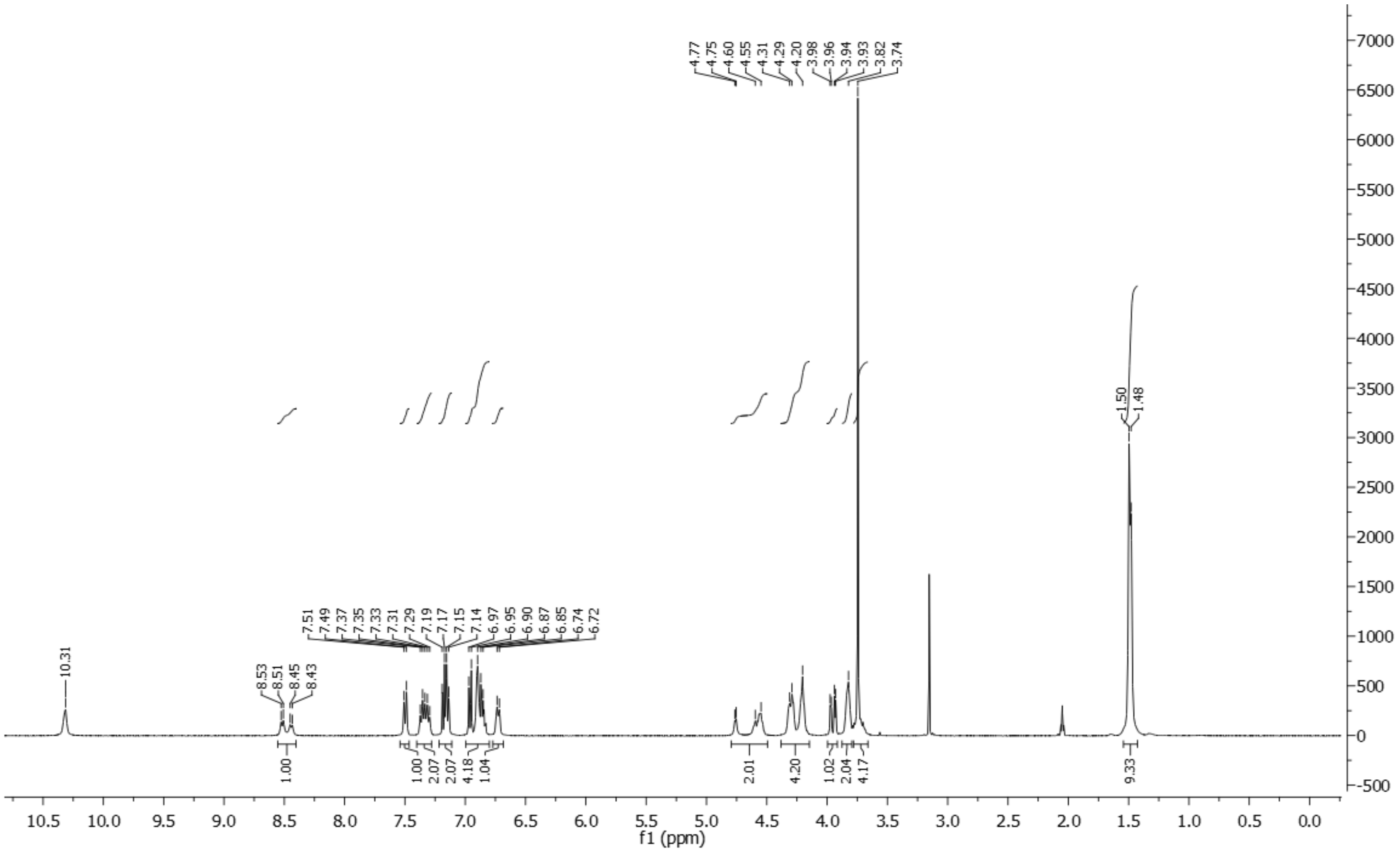

^{1}H-NMR spectrum of the non-deuterated starting material

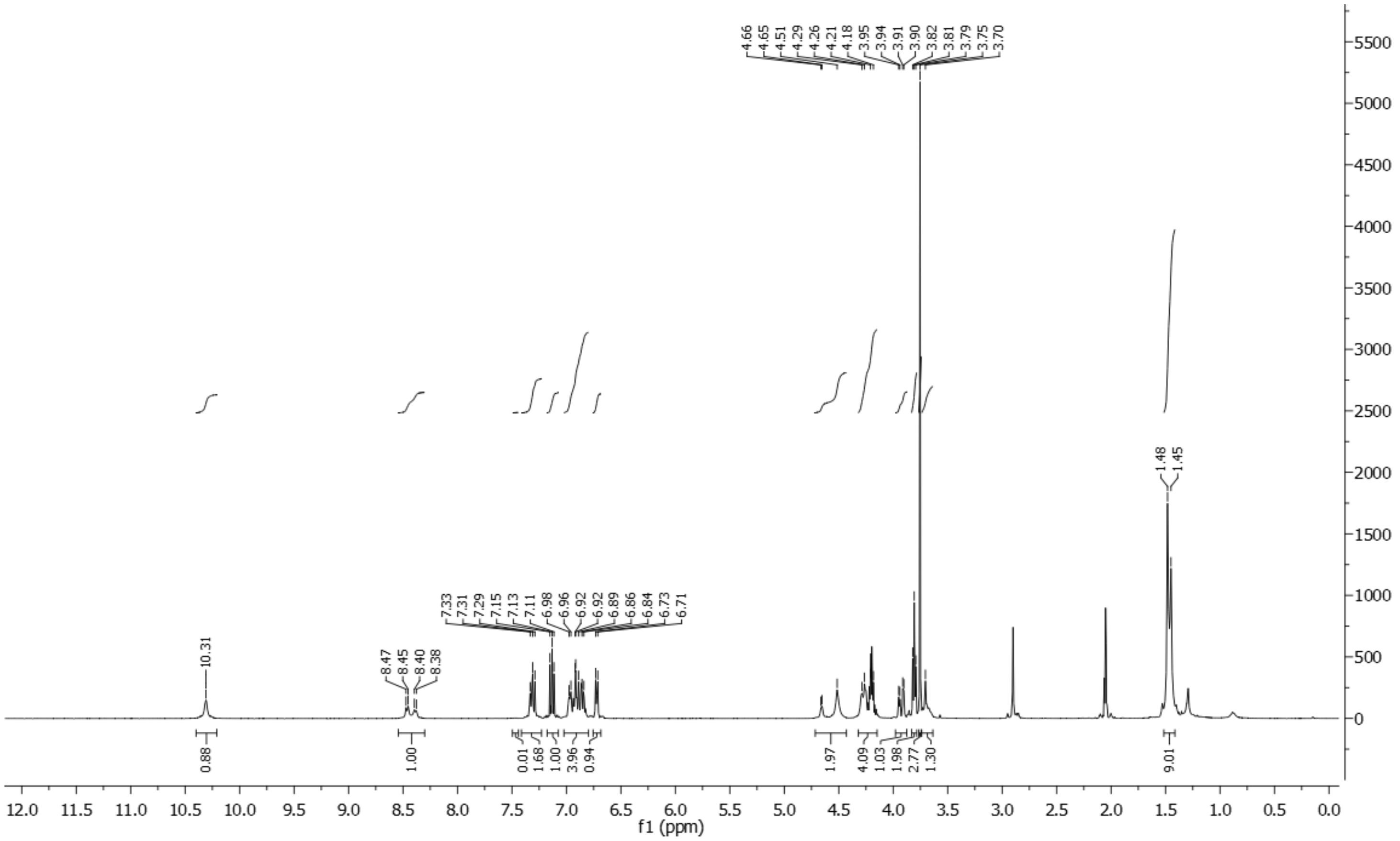

^{1}H-NMR spectrum of **24**

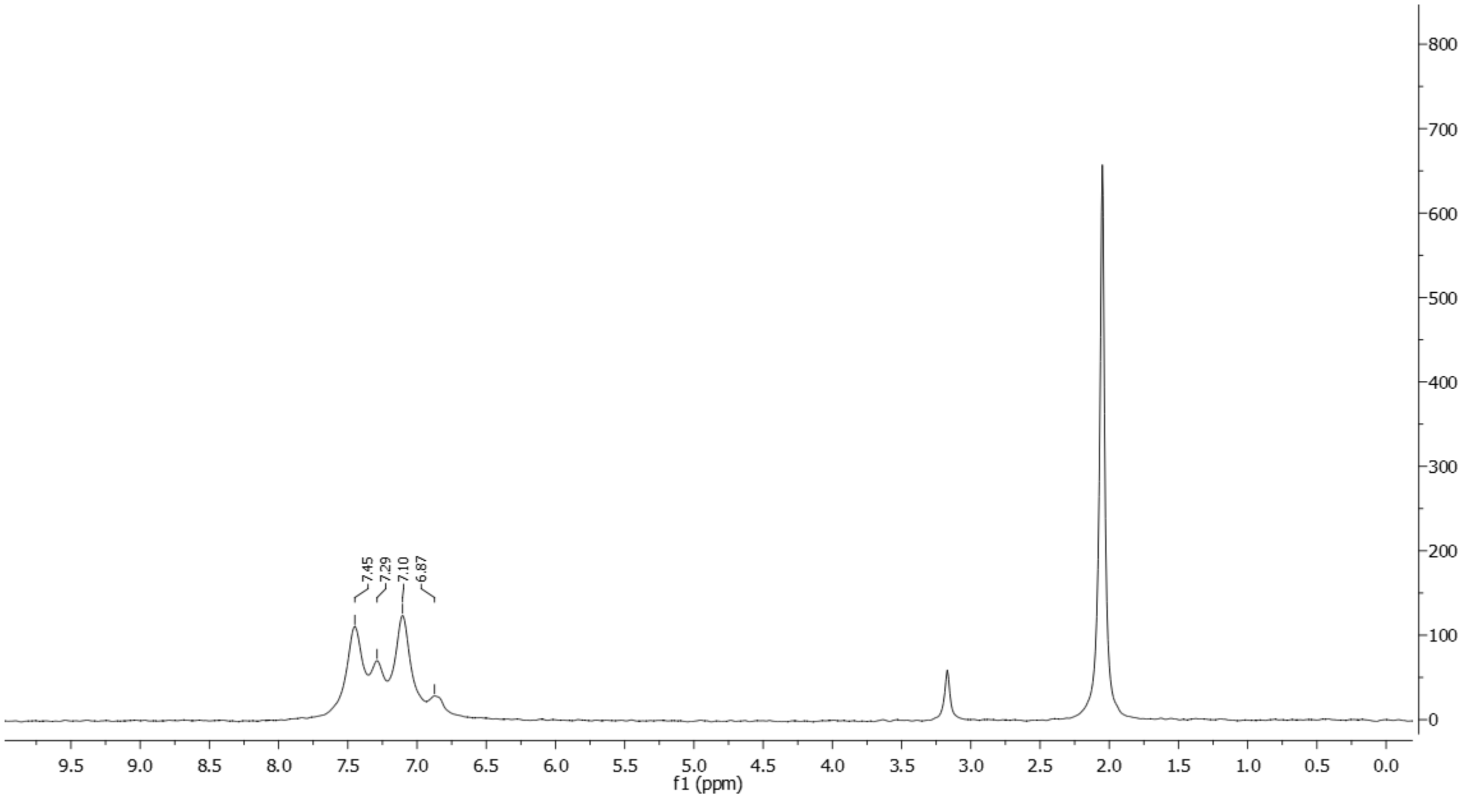

^{2}H-NMR spectrum of **24**

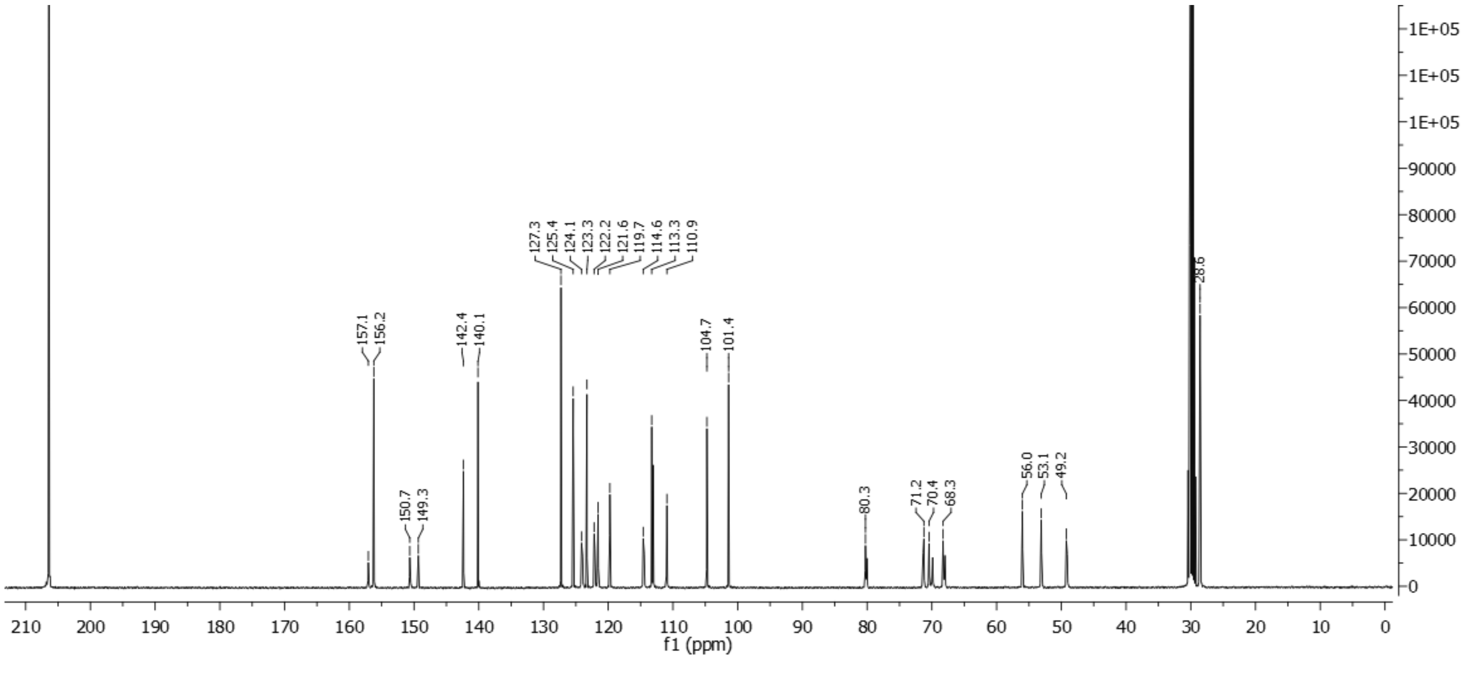

^{13}C-NMR spectrum of the non-deuterated starting material

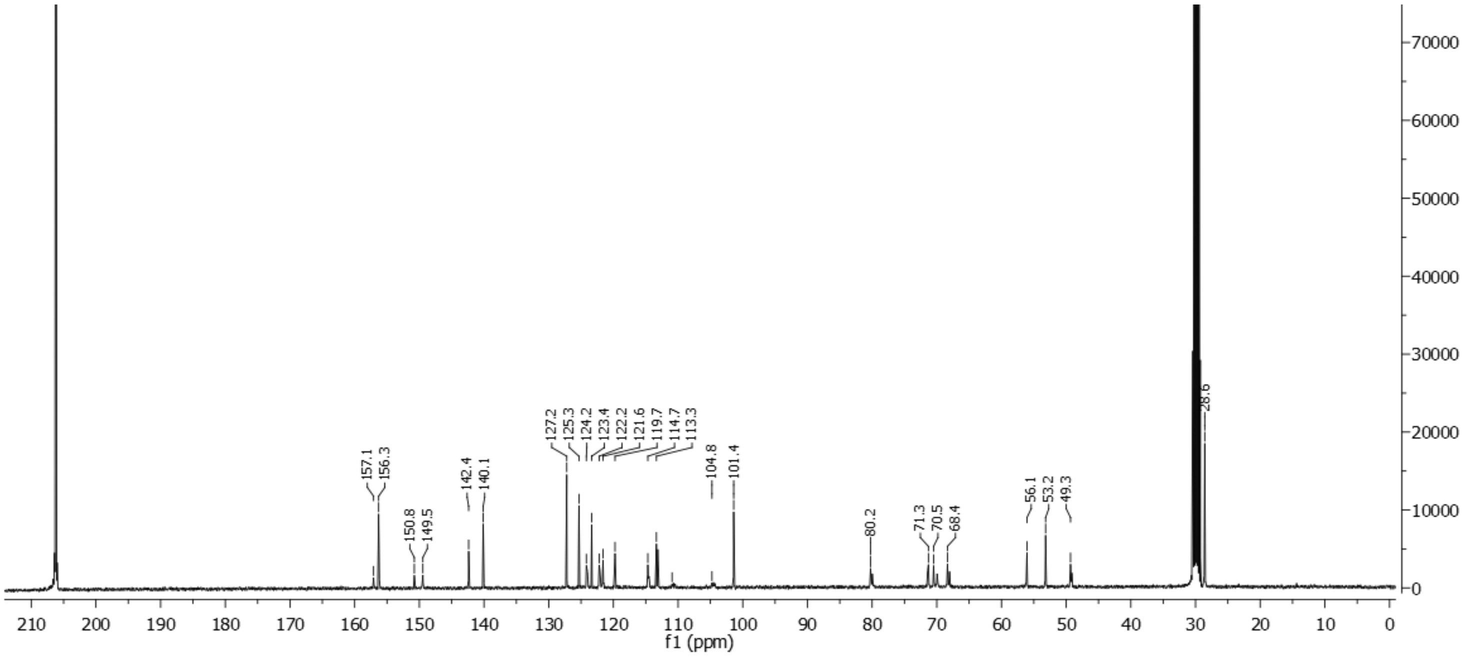

^{13}C-NMR spectrum of **24**

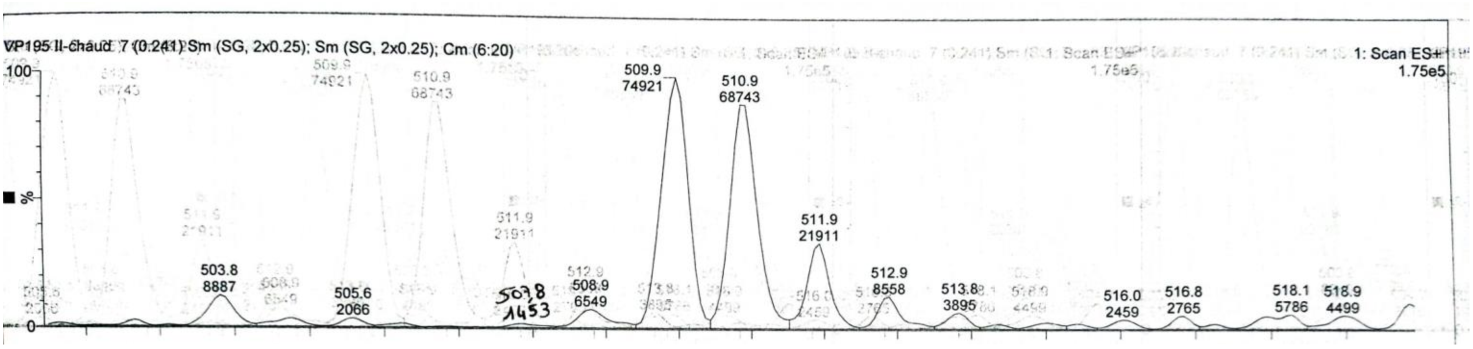

ESI spectrum of **24**

Compound	**M_0**	**M_{+1}**	**M_{+2}**	**M_{+3}**	**M_{+4}**	**M_{+5}**	**M_{+6}**	**M_{+7}**	**M_{+8}**	**Total D**
**1**	0%	6.6%	34.1%	53.8%	-0.4%	0.8%				2.4D
**2**	0%	79.5%	18.6%	0.9%						1.2D
**3**	13.4%	68.3%	17.1%	1.2%						1.1D
**4**	45.4%	54.3%	0.3%							0.6D
**5**	0.1%	2.1%	23.6%	26.5%	42.5%	4.0%	1.0%			3.2D
**6**	1.9%	74.1%	20.9%	3.2%						1.2D
**7**	1.5%	6.0%	32.2%	59.4%	0.9%					2.5D
**8** (1st run)	1.9%	1.0%	19.6%	21.4%	24.6%	32.4%	0.6%			3.7D
**8** (2nd run)	0.0%	0.0%	4.2%	11.1%	25.3%	57.6%	1.8%			4.3D
**9**	0.4%	2.3%	12.3%	27.6%	57.3%					3.4D

**10**	0.9%	0.9%	11.4%	19.0%	67.8%	0.6%				3.5D
**11**	19.9%	5.4%	57.1%	17.5%	18.0%	1.7%				2.4D
**12**	1.8%	0.6%	6.7%	19.7%	71.8%	0.9%				3.7D
**13**	4.8%	21.1%	52.0%	17.9%	4.1%					2.0D
**14**	3.1%	18.1%	77.9%	0.9%	1.1%					1.7D
**15**	8.1%	4.6%	60.4%	26.9%	6.9%	0.9%				2.4D
**16**	1.6%	19.0%	66.3%	13.0%						1.9D
**17I**	0.3%	4.4%	15.7%	27.2%	31.0%	18.2%	3.2%	0.1%		3.5D
**17II**	0.7%	0.0%	2.6%	81.5%	15.2%					3.1D
**18**	8.1%	53.2%	23.5%	12.3%	2.9%					1.4D
**19**	16.1%	7.5%	68.6%	7.9%	0.3%					1.5D
**20**	0.2%	0.2%	0.6%	12.8%	85.9%	0.3%	0.1%			3.8D
**21**	6.8%	41.9%	46.5%	3.5%	-3.8%	1.3%	0.9%	2.8%		1.4D
**22**	0.1%	22.5%	37.8%	30.9%	7.9%	0.7%				1.9D
**23**	0.7%	0.1%	1.1%	1.4%	44.4%	35.2%	13.1%	2.3%	1.6%	4.7D
**24**	1.1%	4.6%	55.5%	33.4%	1.7%	3.7%				2.2D

Tritiations of Drugs

Tritiation of astemizole **18***

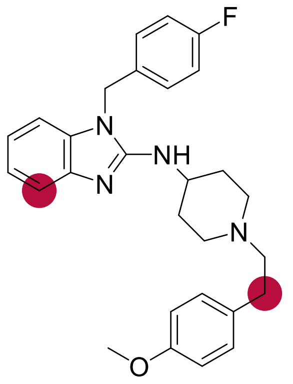

23.7 Ci/mmol

Chemical Formula: $C_{28}H_{31}FN_4O$

Substrate	Solvent (Volume)	RuNp@PVP cat.	T_2 [21°C]
5.0mg, 11µmol	THF (0.4mL)	14.0mg, 92mol%	970mbar, 12.4Ci

Workup and purification:

After cooling down to room temperature, EtOAc /Cy (1:1, 1mL) was added to the reaction mixture and stirred for 3min to let precipitate RuNp@PVP. The suspension was passed through a C18-SiO_2 pad. The product was eluted with EtOAc (4.5mL). The solvent was removed under vacuum to give a white solid.

Analytical HPLC was performed on a Waters XBridge Prep Phenyl 150mm x 10mm, 5µm, column. Condition: 4.0mL/min, UV & mass detection, 25°C, Solvents & gradients: Solvent A : H_2O + 0.1% HCOOH; Solvent B : ACN + 0.1% HCOOH

t (0min)	95%A	5%B
t(25min)	0%A	100%B
t(30min)	0%A	100%B

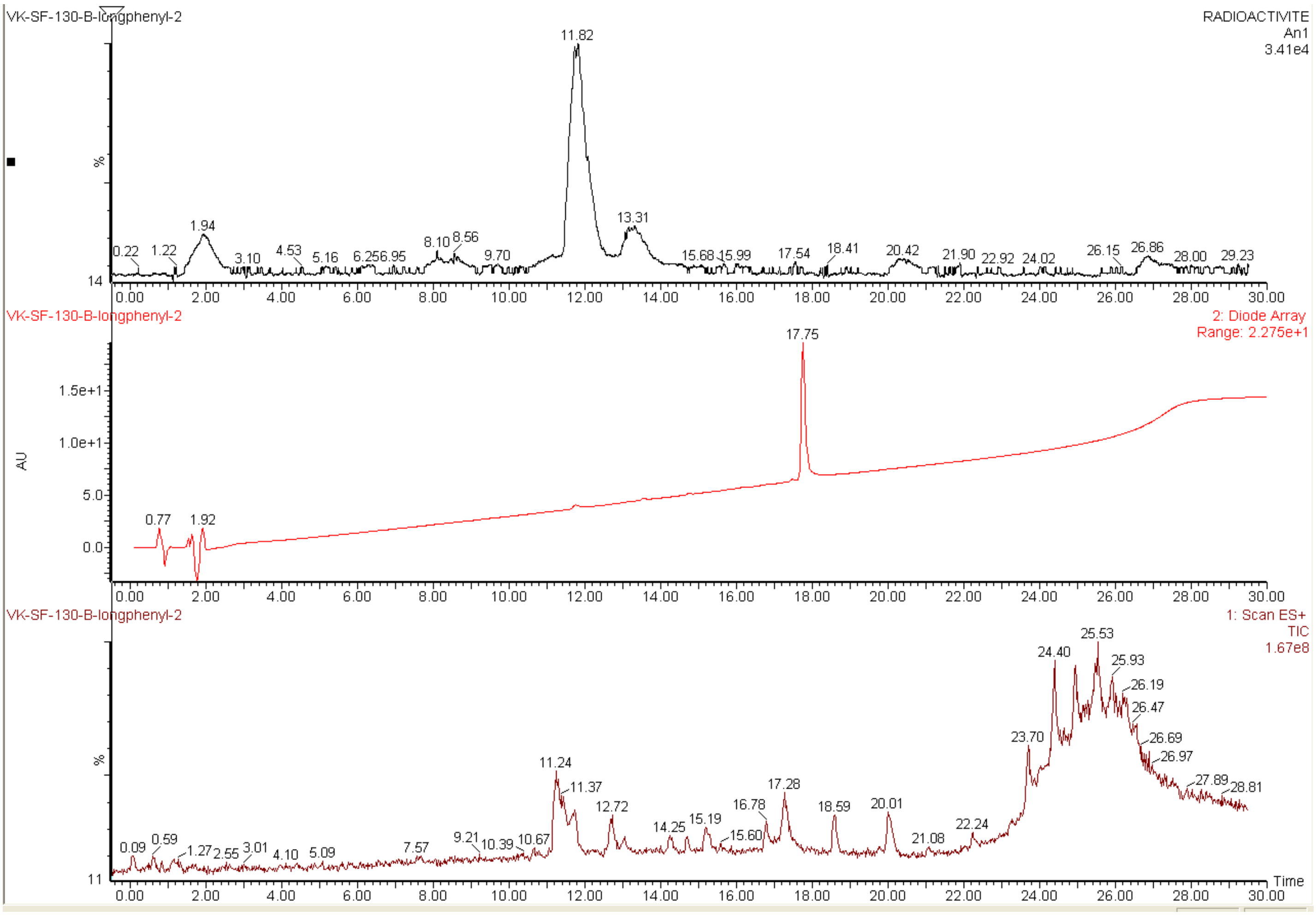

HPLC chromatogram

^{3}H NMR (427 MHz, Acetone-*d*$_6$): δ 7.31 (s, 0.42T), 2.60 – 2.45 (m, 0.33T).

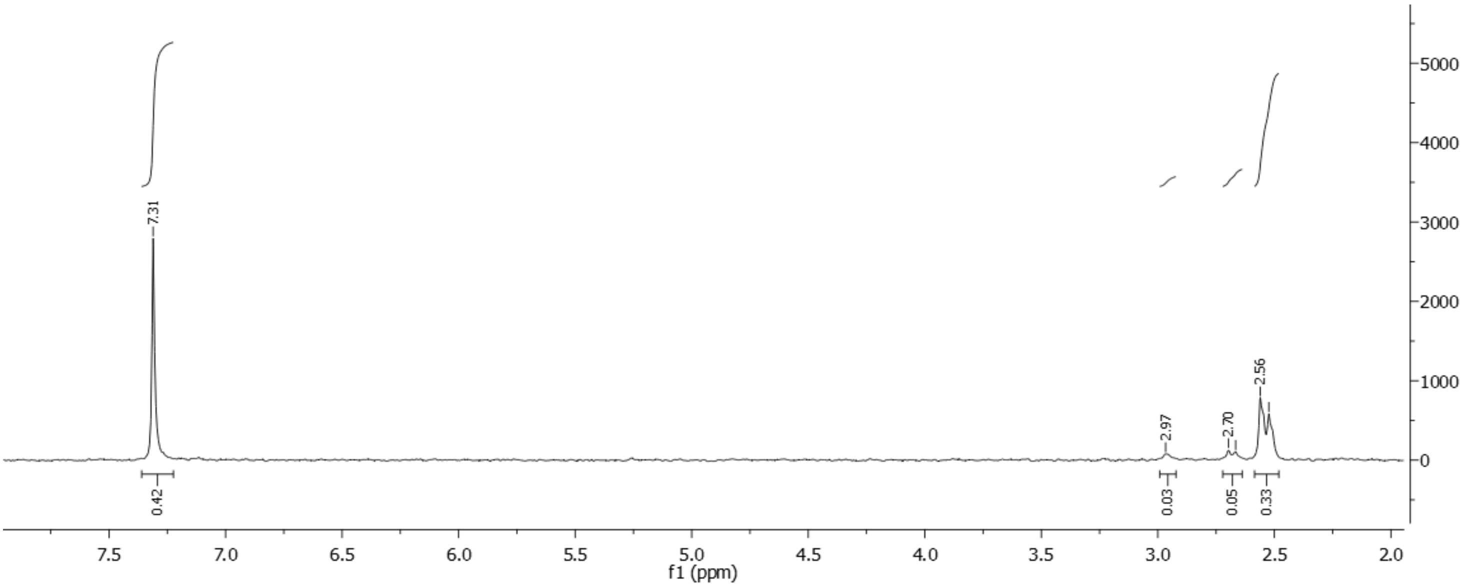

^{3}H-NMR spectrum of **18***

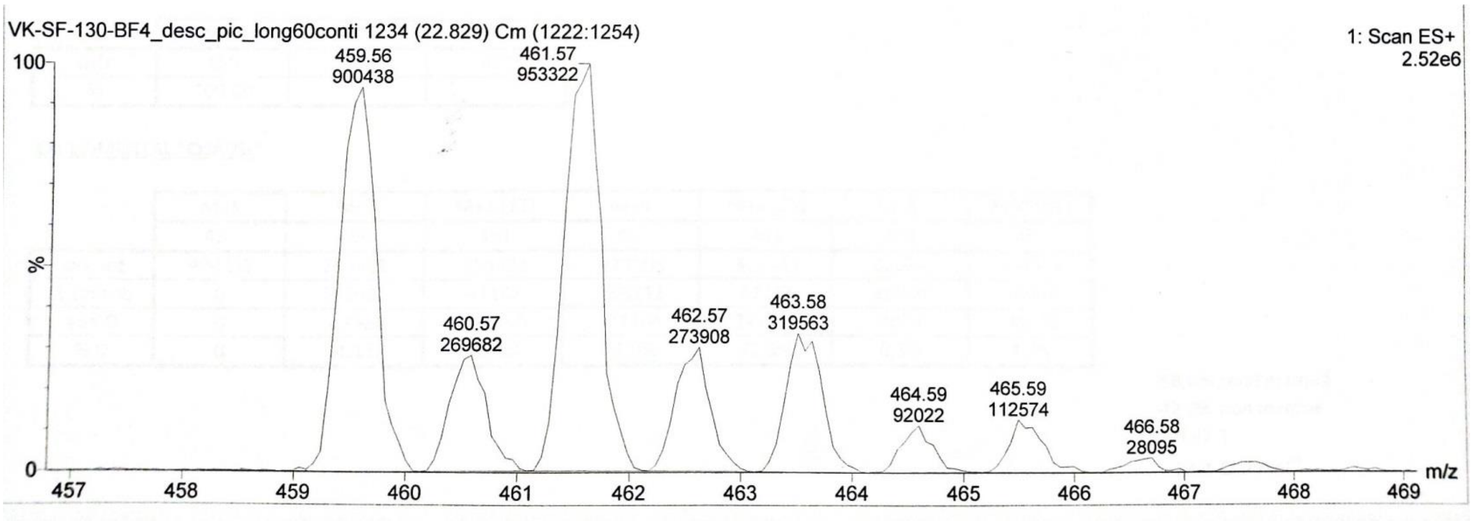

ESI-spectrum of **18***

Tritiation of fluconazole **20***

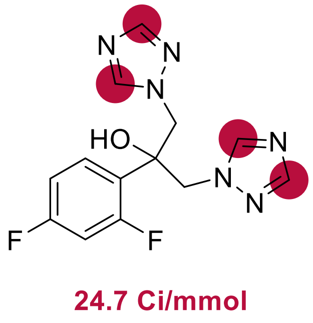

Chemical Formula: $C_{13}H_{12}F_{2}N_{6}O$

Substrate	Solvent (Volume)	RuNp@PVP cat.	T_2 gas [21°C]
5.0mg, 17µmol	THF (0.5mL)	3mg, 13mol%	869mbar, 11.1Ci

Workup and purification:

After cooling down to room temperature, EtOAc/Cy (1:1, 1mL) was added to the reaction mixture and stirred for 3 minutes to let precipitate RuNp@PVP. The suspension was passed through a SiO_2 pad and then eluted with distilled THF (5mL). The solvent was removed under vacuum to give a white solid.

Analytical HPLC was performed on a Waters XBridge C18 250mm x 4.6mm, 3.5µm, column. Condition: 1.0mL/min, UV & mass detection, 25°C, Solvents & gradients: Solvent A: H_2O; Solvent B: MeOH

t (0min)	95%A	5%B
t(25min)	0%A	100%B
t(30min)	0%A	100%B

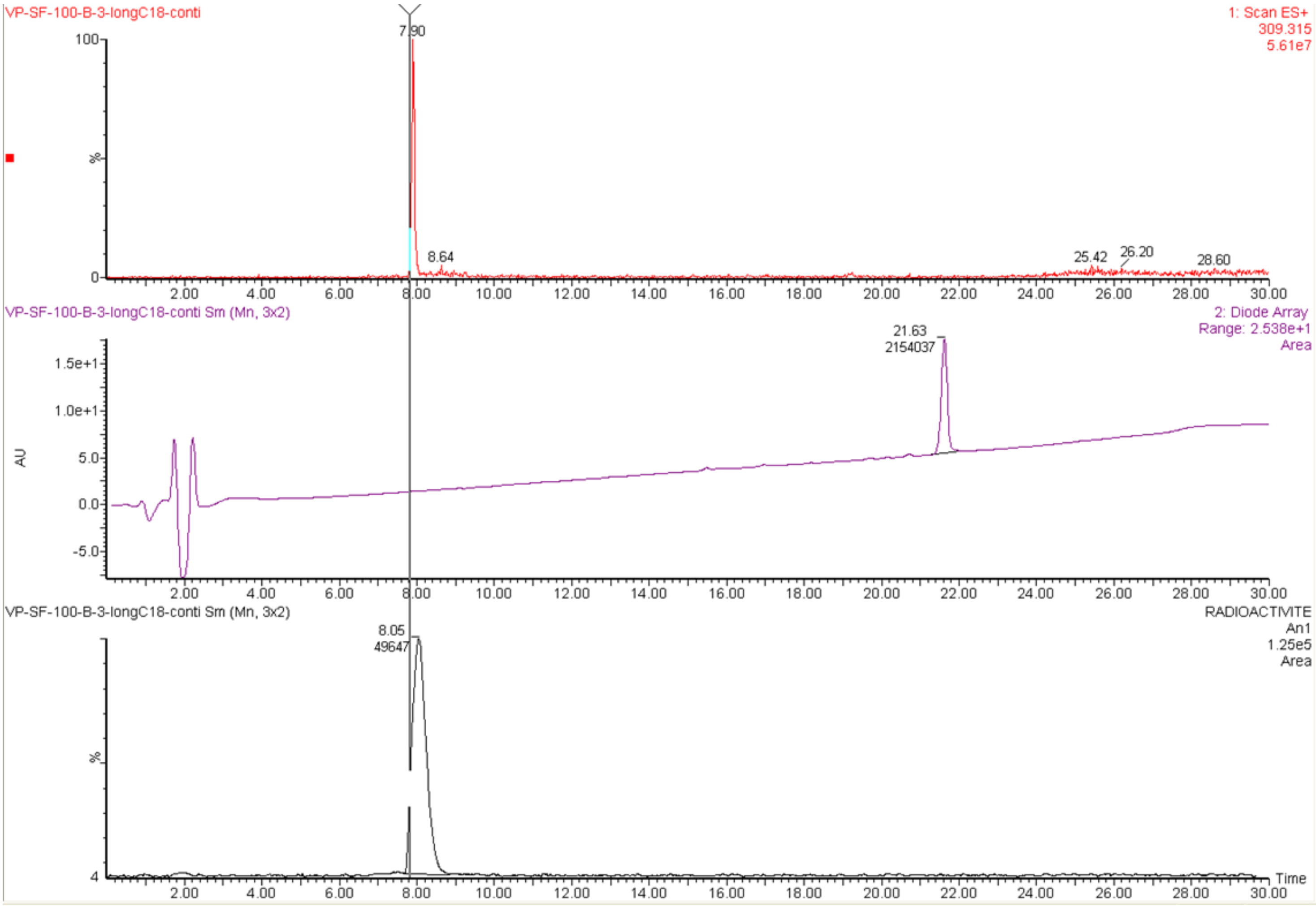

HPLC chromatogram

^{3}H NMR (427 MHz, Acetone-*d_6*): δ 8.33 (s, 0.66T), 7.79 (s, 0.24T).

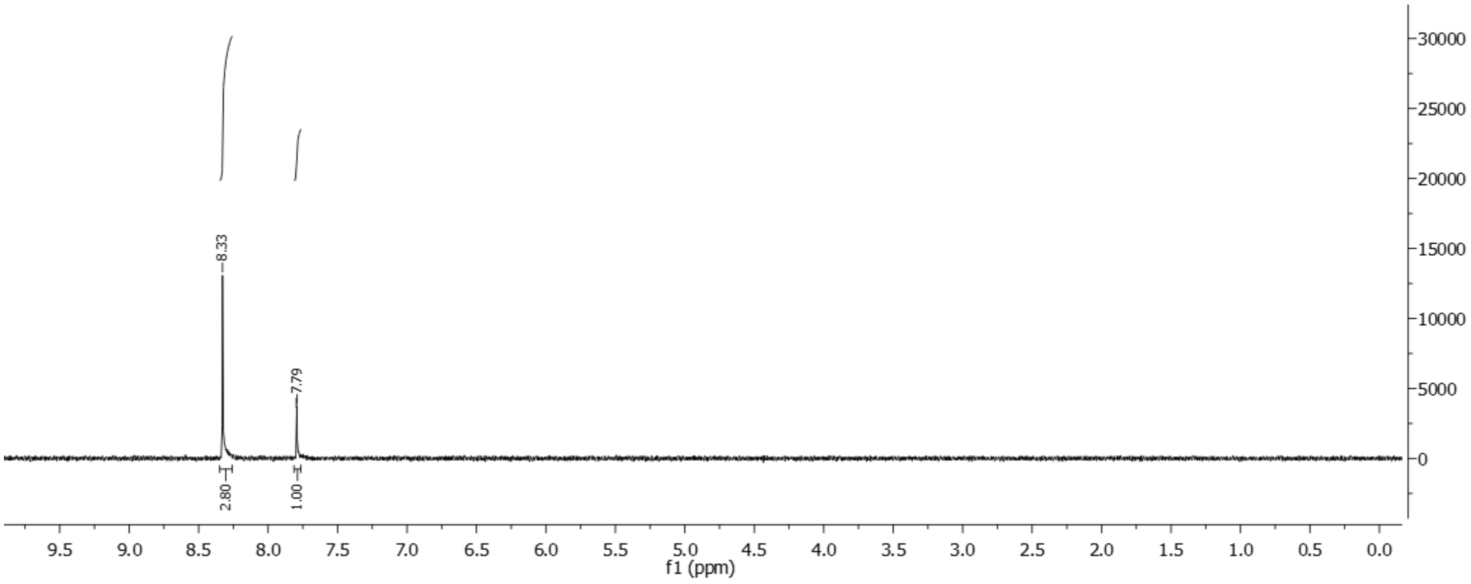

^{3}H-NMR spectrum of **20***

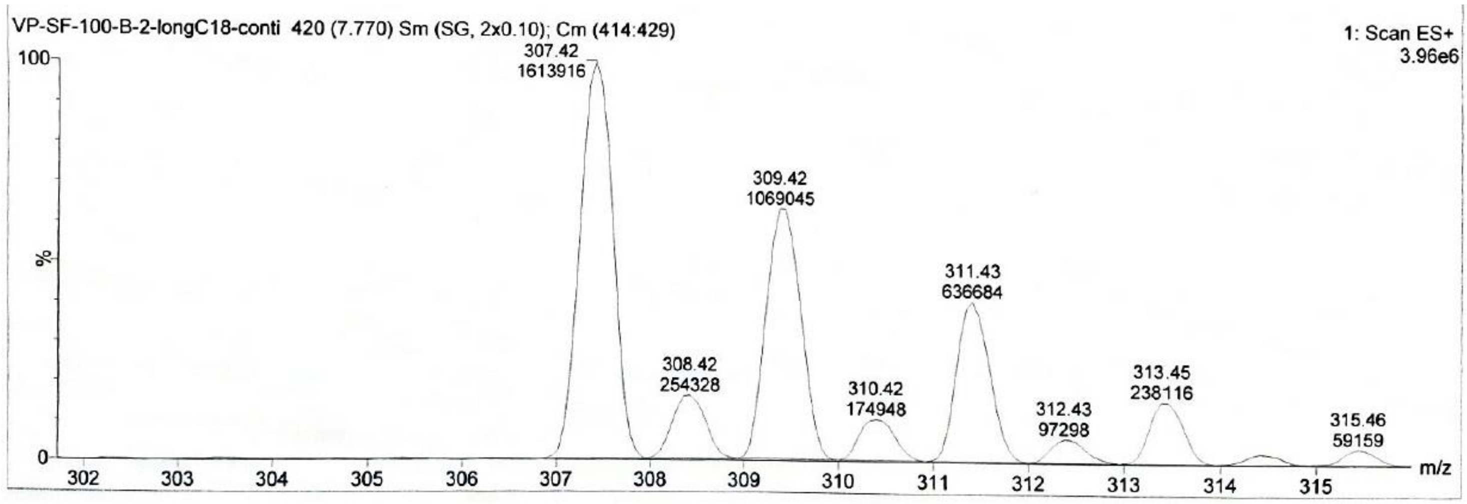

ESI-spectrum of **20***

Tritiation of *N*-boc-carvedilol **24***

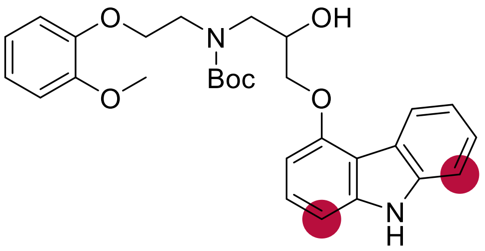

8.7 Ci/mmol

Chemical Formula: $C_{29}H_{34}N_2O_6$

Substrate	Cs_2CO_3	Solvent (Volume)	RuNp@PVP cat.	T_2 [21°C]
5.0mg, 10µmol	3.3mg, 10µmol	THF (0.3mL)	3mg, 20mol%	519mbar, 6.6Ci

Workup and purification:

After cooling down to room temperature, an acetic acid solution (1% in EtOAc, 0.5mL) was added to the reaction mixture and stirred for 3 minutes to let precipitate RuNp@PVP. The suspension was passed through a SiO_2 pad and then eluted with EtOAc (1.5mL). The solvent was removed under vacuum to give a white solid.

Analytical HPLC performed on an Waters XBridge C18 100mm x 4.6mm, 3.5µm, column. Condition: 1.0mL/min, UV & mass detection, 25°C, Solvents & gradients: Solvent A : H_2O + 0.1% HCOOH; Solvent B : ACN + 0.1% HCOOH

t (0min)	95%A	5%B
t(25min)	0%A	100%B
t(30min)	0%A	100%B

^{3}H NMR (427 MHz, Acetone-*d*$_6$): δ 7.59 (s, 0.15T), 7.25 (s, 0.15T).

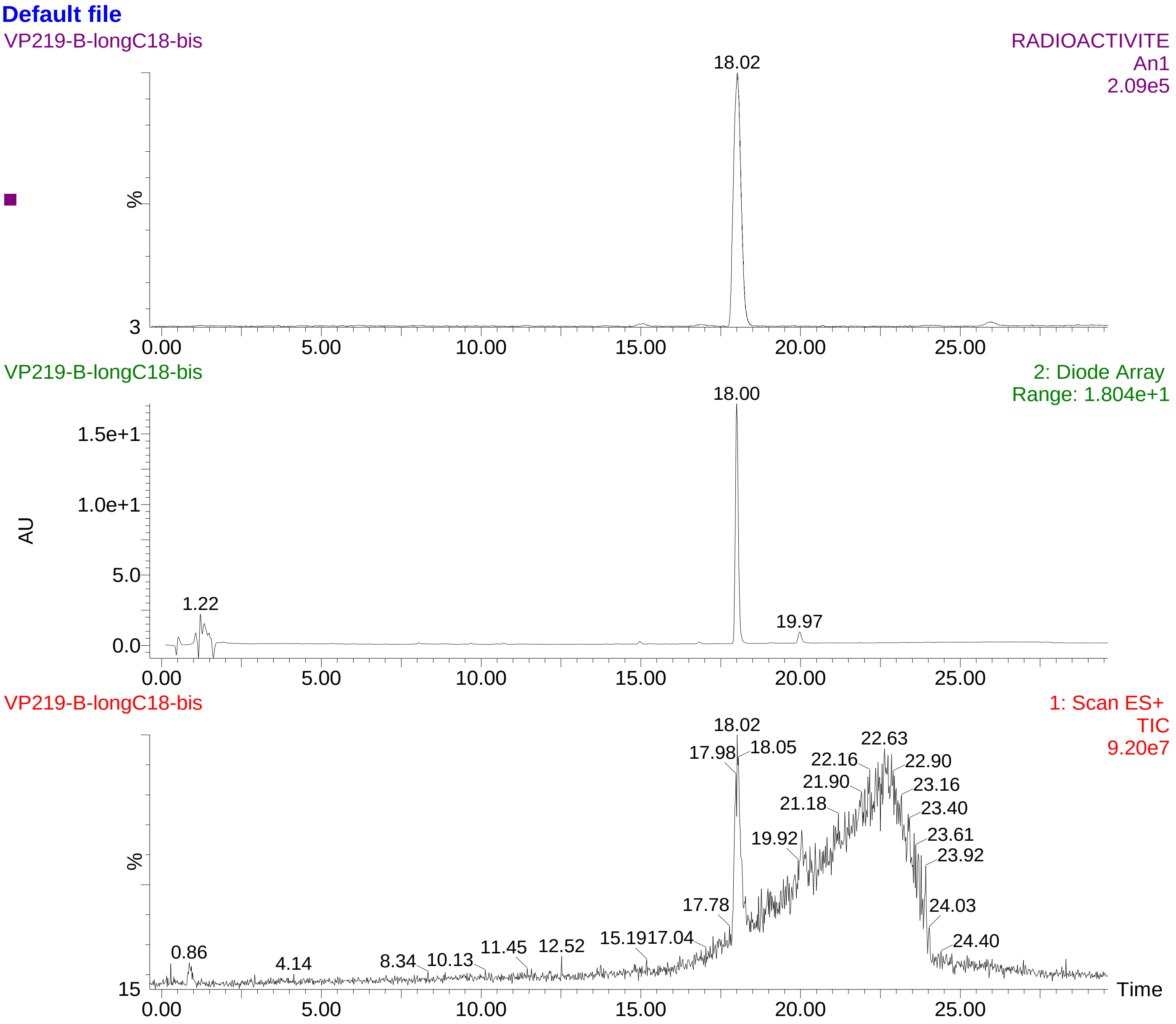

Chromatogram of **24***

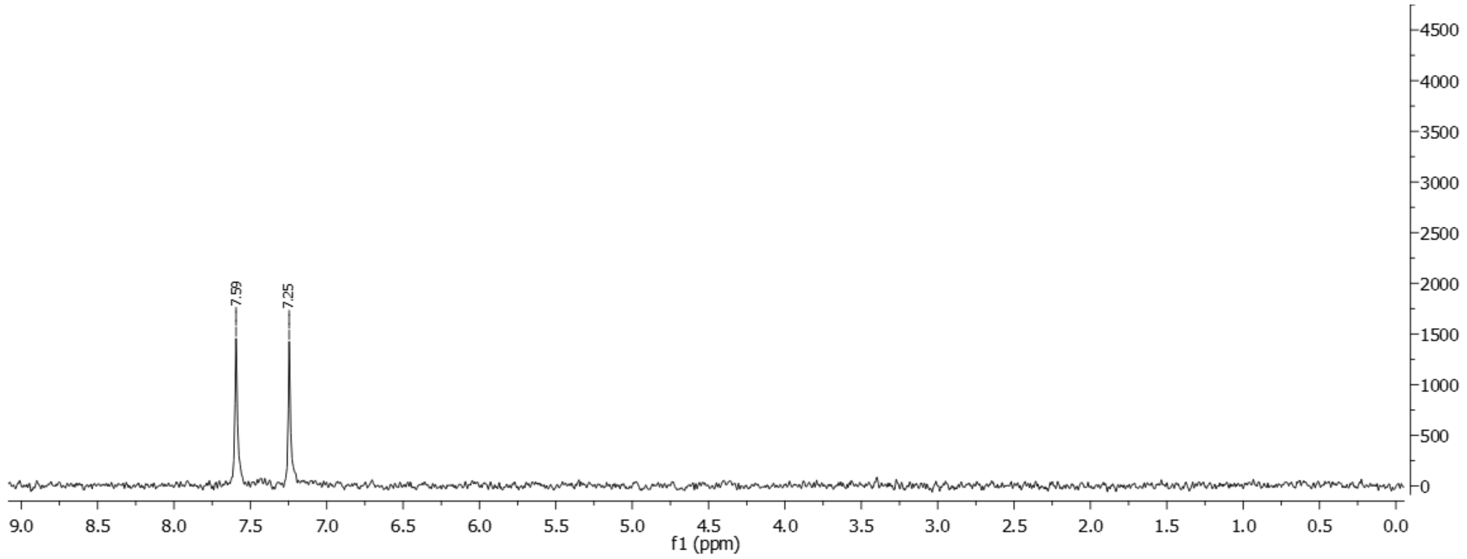

^{3}H-NMR spectrum of **24**

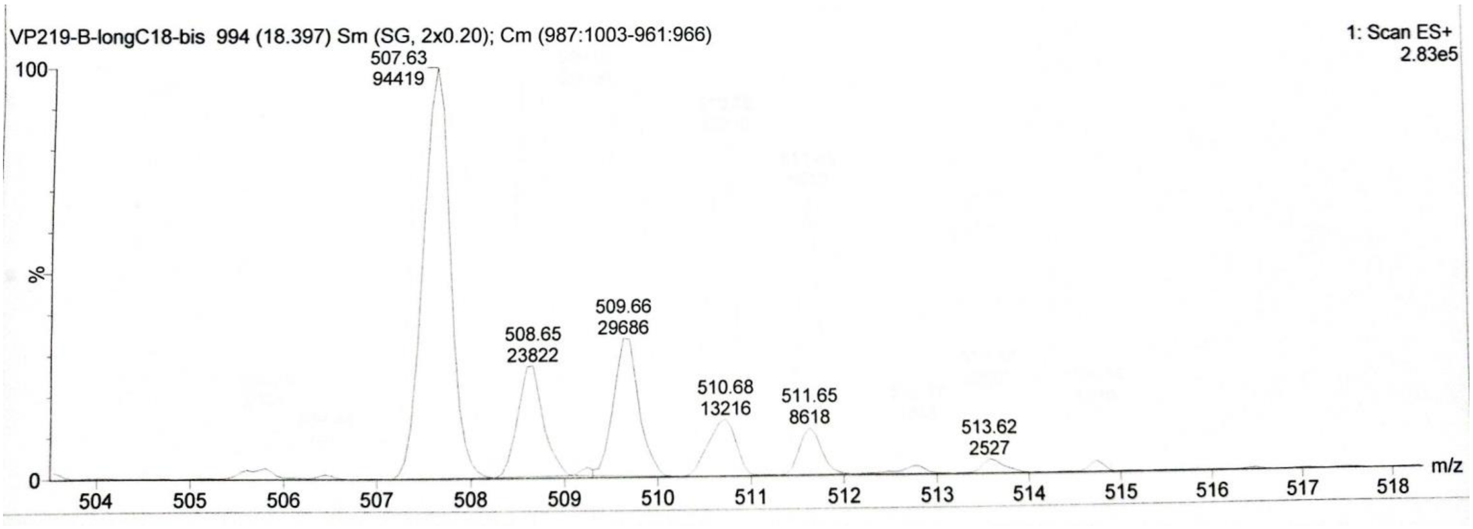

ESI spectrum of **24***

Compound	M_0	M_{+1}	M_{+2} (1T)	M_{+3}	M_{+4} (2T)	M_{+5}	M_{+6} (3T)	M_{+7}	M_{+8} (4T)	Total T
**18***	42.0%	-1.1%	42.5%	-1.0%	12.9%	0.1%	4.5%			0.8T
**20***	46.1%	-0.3%	29.9%	0.1%	17.7%	-0.1%	6.6%			0.9T
**24***	77.1%	-6.6%	21.3%	4.0%	4.2%					0.3T

Reaction pathways and syntax of the labels

Syntax of the labels $N^{X[H]*[,Y[H]*][‡]}$

- the number, **N**, corresponds to a given substrate
- X and Y refer to the atoms of the substrate (C or N) coordinated to a metal site, *, of a NP. For example, a substrate coordinated to a surface as a dimetallacycle, prior to any C-H or N-H activation, is designated as $\mathbf{N}^{XH*,YH*}$, whereas a monodentate ligand is designated as $\mathbf{N}^{XH*}$. A label $\mathbf{N}^{(XH,YH)*}$ is used when two atoms of the substrate are coordinated to the same metal site.
- H, optional: after X-H activation by a metal site *, a substrate **N** becomes $\mathbf{N}^{X*}$
- D, optional: after D insertion in a X-* bond, a substrate **N** becomes $\mathbf{N}^{XD*}$
- ‡, optional, is used for transition states.

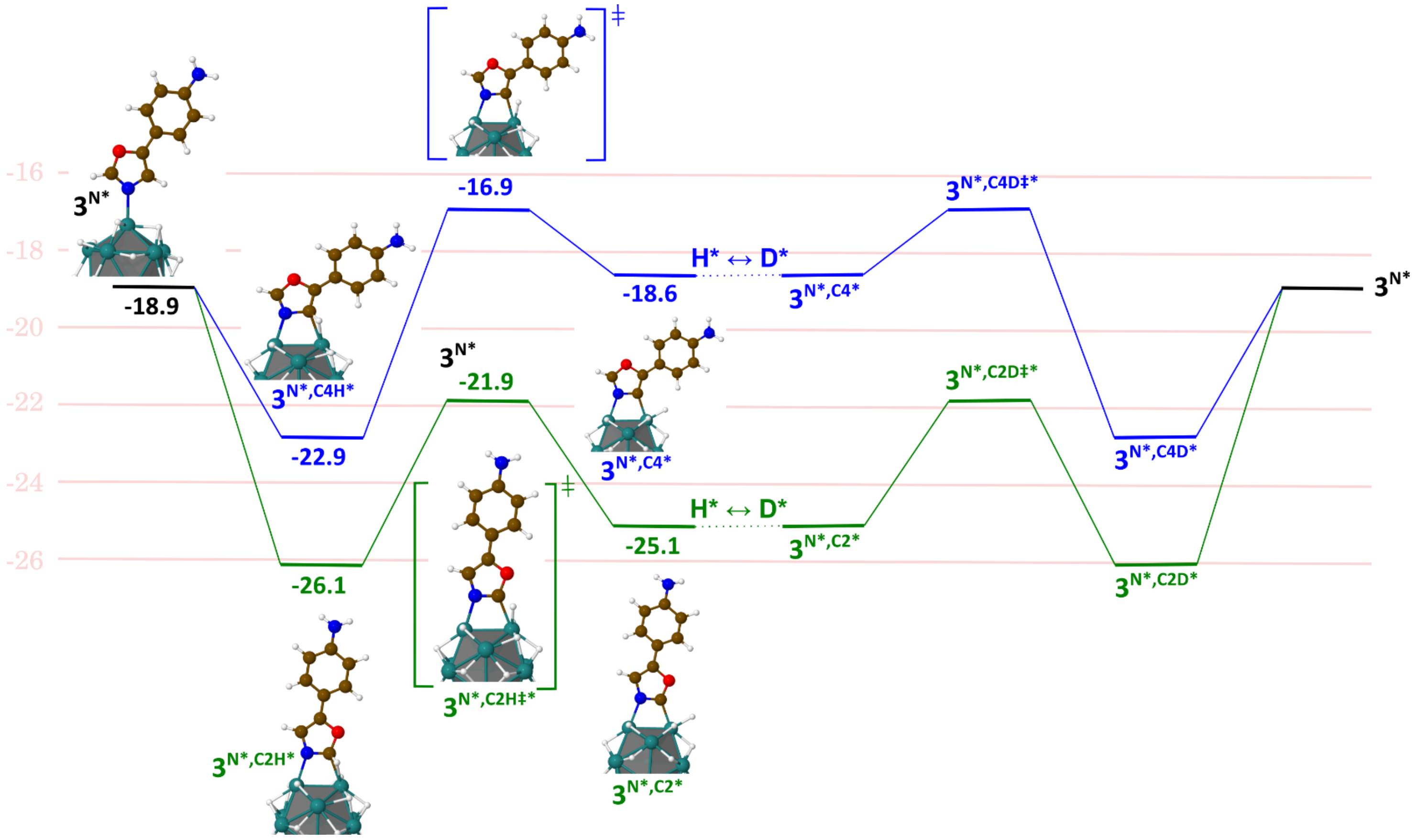

Figure S1**:** Energy diagram for the Langmuir–Hinshelwood-type H/D exchange on the C_2 (green pathway) and C_4 (blue pathway) position of the oxazole ring of compound **3**; energies are given in kcal.mol^{-1}. For the sake of clarity, geometries are not given on the way back to $\mathbf{3}^{N*}$, i.e. after D insertion. The H* ⟷ D* step stands for the easy reorganization of hydrides and deuterides at the surface of RuNps

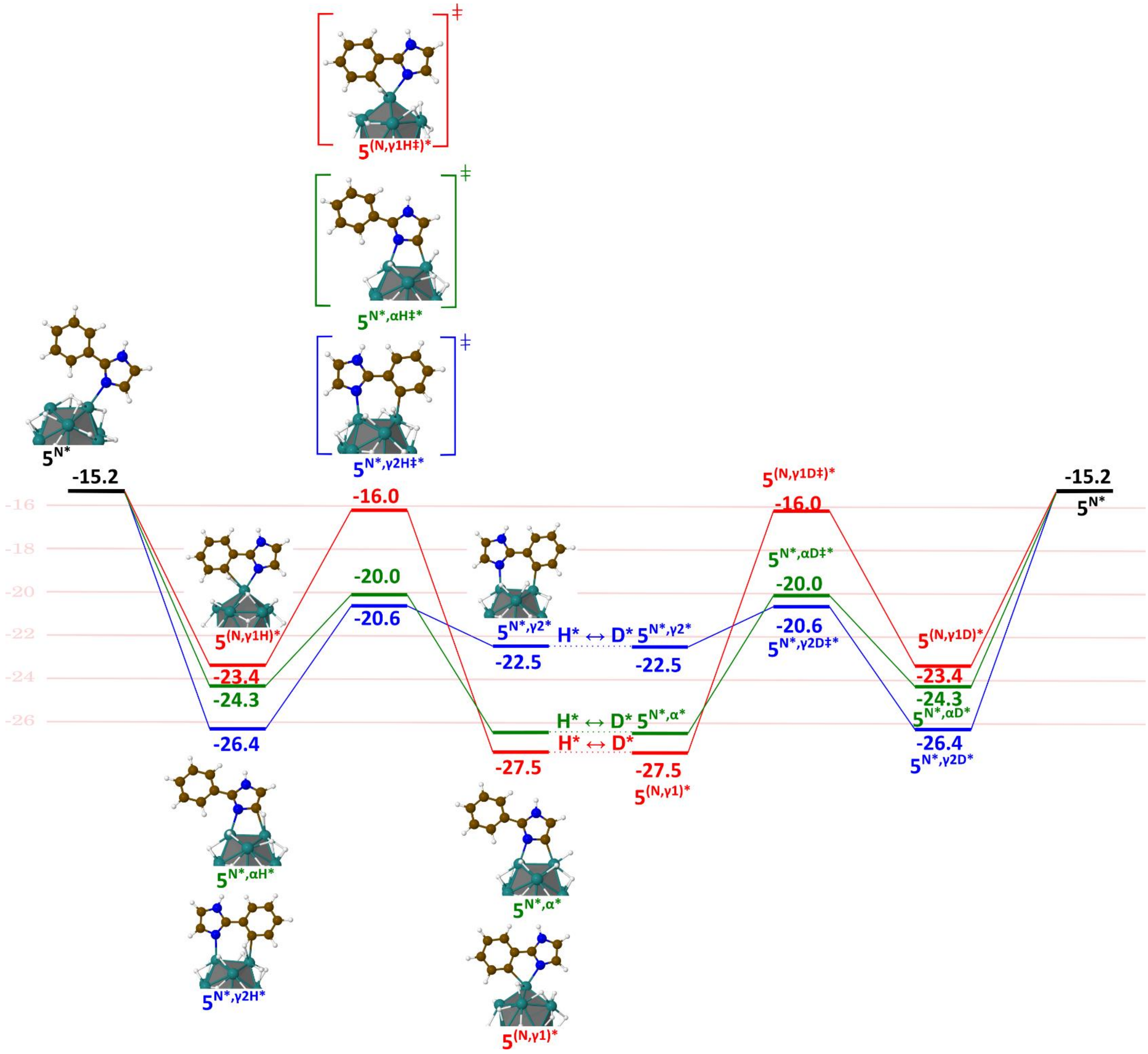

Figure S2**:** Energy diagram for the Langmuir–Hinshelwood-type H/D exchange on 5 in the ortho-position of the phenyl (blue and red pathways) and at α-positions relative to the imidazole nitrogen atoms (green pathway); energies are given in kcal.mol^{-1}. For the sake of clarity, geometries are not given on the way back to $\mathbf{5^{N*}}$, i.e. after D insertion. The H* ⟷ D* step stands for the easy reorganization of hydrides and deuterides at the surface of RuNps

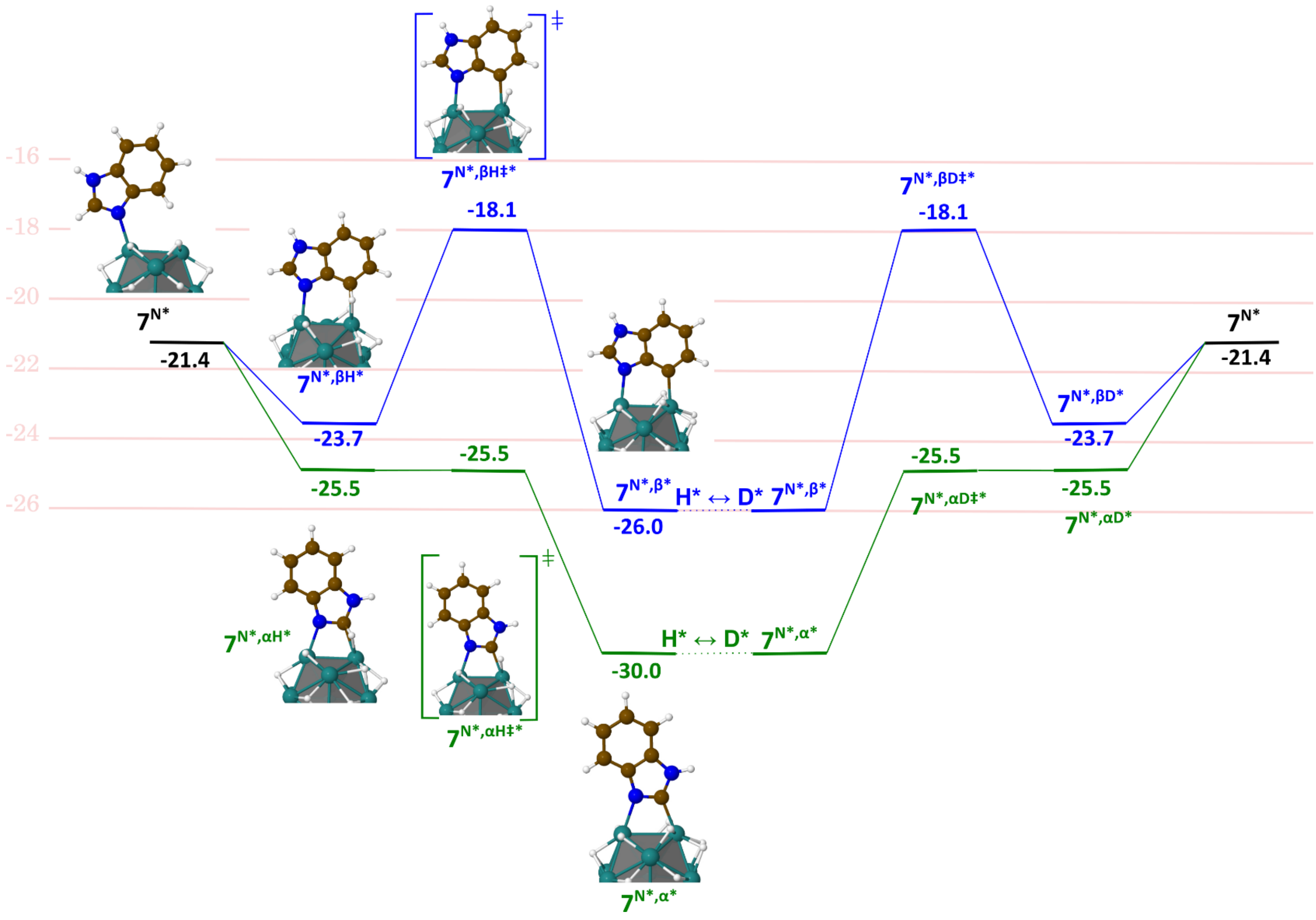

Figure S3**:** Energy diagram for the Langmuir–Hinshelwood-type H/D exchange on **7** in α (green pathway) and β (blue pathway) positions of the nitrogen atoms; energies are given in kcal.mol^{-1}. For the sake of clarity, geometries are not given on the way back to **7**^{N*}, i.e. after D insertion. The H* ↔ D* step stands for the easy reorganization of hydrides and deuterides at the surface of RuNps

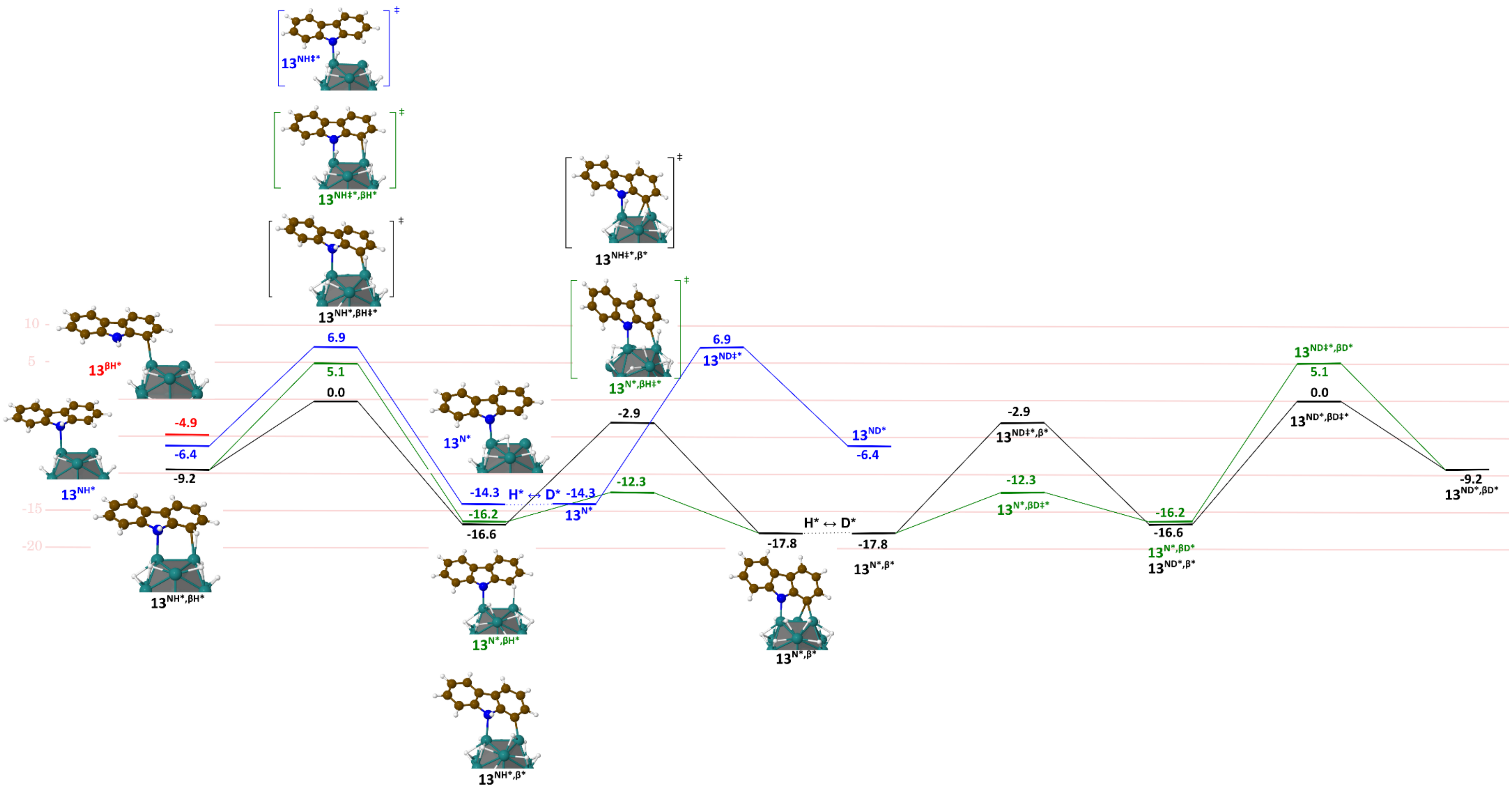

Figure S4: Energy diagram for the Langmuir–Hinshelwood-type H/D exchange on **13**: on N and then in β (green pathway); in β and then on N (black pathway); directly on N (blue pathway). The π adsorption energy in β is also given (red energy level). Energies are given in kcal.mol^{-1}. For the sake of clarity, geometries are not given on the way back to **13**^{ND*,βD*}, i.e. after D insertion. The H* ⟷ D* step stands for the easy reorganization of hydrides and deuterides at the surface of RuNps
